# miR-26a and miR-384-5p are required for LTP maintenance and spine enlargement

**DOI:** 10.1038/ncomms7789

**Published:** 2015-04-10

**Authors:** Qin-Hua Gu, Danni Yu, Zhonghua Hu, Xing Liu, Yanqin Yang, Yan Luo, Jun Zhu, Zheng Li

**Affiliations:** 1 Unit on Synapse Development and Plasticity, National Institute of Mental Health, National Institutes of Health, Bethesda, Maryland 20892, USA; 2 Department of Statistics, Purdue University, West Lafayette, Indiana 47907, USA; 3 Systems Biology Center, National Heart Lung and Blood Institute, National Institutes of Health, Bethesda, Maryland 20892, USA

## Abstract

Long-term potentiation (LTP) is a form of synaptic plasticity that results in enhanced synaptic strength. It is associated with the formation and enlargement of dendritic spines—tiny protrusions accommodating excitatory synapses. Both LTP and spine remodelling are crucial for brain development, cognition and the pathophysiology of neurological disorders. The role of microRNAs (miRNAs) in the maintenance of LTP, however, is not well understood. Using next-generation sequencing to profile miRNA transcriptomes, we demonstrate that miR-26a and miR-384-5p specifically affect the maintenance, but not induction, of LTP and different stages of spine enlargement by regulating the expression of RSK3. Using bioinformatics, we also examine the global effects of miRNA transcriptome changes during LTP on gene expression and cellular activities. This study reveals a novel miRNA-mediated mechanism for gene-specific regulation of translation in LTP, identifies two miRNAs required for long-lasting synaptic and spine plasticity and presents a catalogue of candidate ‘LTP miRNAs'.

## Abstract (web summary)

Long-term potentiation (LTP) is a form of synaptic plasticity that results in enhanced synaptic strength. Here, the authors demonstrate that miR-26a and miR-384-5p affect the maintenance, but not induction, of LTP as well as spine enlargement by regulating the expression of RSK3.

## Graphical abstract

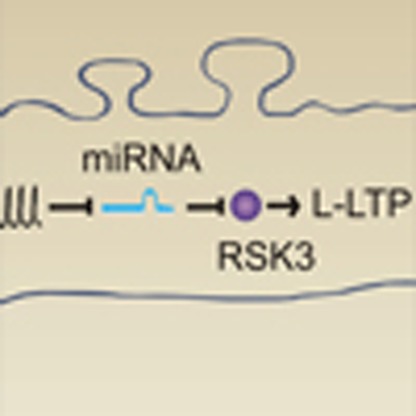

Long-term potentiation (LTP) of synaptic transmission is a form of synaptic plasticity that leads to long-lasting enhancement of synaptic strength. LTP is a prominent cellular model for encoding and storing information in the brain^1^,^2^,^3^,^4^. On the basis of temporal characteristics, LTP is classified into two forms: short- and long-lasting LTP. Short-lasting LTP is induced by weak stimulation of synaptic inputs, persists for no more than 2 h and involves no protein synthesis. Long-lasting LTP, which is induced by strong stimulation, lasts longer than 2 h, requires *de novo* protein synthesis and is essential for memory formation^5^,^6^.

Protein synthesis in long-lasting LTP is promoted at both the translational and transcriptional levels, with translation required throughout and transcription roughly 2 h after LTP is induced in the hippocampal CA1 region^6^. Extracellular signal-regulated kinase and mammalian target of rapamycin play important roles in translational regulation in LTP. They promote phosphorylation of proteins in the translational machinery to facilitate translation initiation, thereby enhancing protein synthesis^5^.

Protein synthesis is also required for other forms of synaptic plasticity, such as long-term synaptic depression (LTD), which leads to decreases in synaptic strength. The unique changes in synapses that occur during each type of synaptic plasticity cannot be accounted for by global upregulation of translation. Indeed, translation of selective messenger RNAs (mRNAs) has been implicated in synaptic plasticity^5^. For instance, after LTP is induced, Ca^2+^/calmodulin-dependent protein kinases II (CaMKII) promotes phosphorylation of cytoplasmic polyadenylation element (CPE)-binding protein, which in turn stimulates the polyadenylation and translation initiation of mRNAs containing CPE^7^,^8^. However, little is yet known about the mechanisms of gene-specific regulation of translation and about which genes are translated during LTP.

MicroRNAs (miRNAs), short non-coding RNAs, regulate translation by binding to the 3′ untranslated region (UTR) of mRNAs^9^. Of the >1,000 mammalian miRNAs identified to date, hundreds are expressed in the brain^10^,^11^. Several miRNAs (for example, miR-134, miR-125, miR-138, miR-132 and miR-29) have been shown to regulate dendritic spine morphology and synaptic transmission^12^. The role of miRNAs in LTP, however, has yet to be determined.

Here we combine next-generation sequencing, bioinformatics, electrophysiology and time-lapse imaging to investigate the role of miRNAs in LTP. These studies lead to the identification of miR-26a, miR-384-5p and let-7a as essential for LTP maintenance and enlargement of dendritic spines. Moreover, we show that ribosomal S6 kinase 3 (RSK3) mediates the function of miR-26a and miR-384-5p in LTP and present computational evidence that miRNAs exert both diverse and concerted effects in LTP.

## Results

### miRNAs differentially expressed in LTP

To investigate the role of miRNAs in LTP, we first examined changes in miRNA expression during LTP using next-generation deep sequencing. LTP was induced in hippocampal slices prepared from mice (2∼3 weeks old) by tetanic stimulation of the Schaffer collateral pathway (4 trains of 100 Hz, 1 s stimulation separated by 5 min)^13^,^14^,^15^,^16^. Long-lasting LTP was induced, as evidenced by the persistent increase in field excitatory postsynaptic potentials (fEPSPs) (247.9±24.7% of pre-induction baseline at 10 min post stimulation, 234.8±31.5% at 1 h and 191.3±35.3% at 4 h; Supplementary Fig. 1a). The LTP was dependent on protein synthesis as fEPSPs increased only transiently and returned to the baseline level by 2 h after stimulation in the presence of the translational inhibitor anisomycin (20 μM) (Supplementary Fig. 1a).

**Figure 1:**
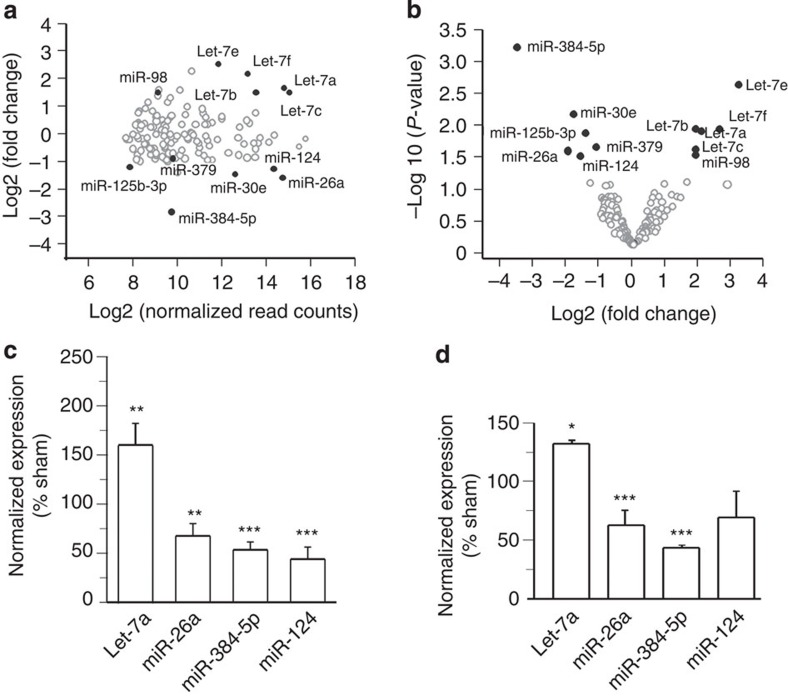
miRNAs differentially expressed in LTP. (**a**) The fold change (on log2 scale) of each miRNA is plotted against its normalized read number (on log2 scale). (**b**) The *P* value (on a log10 scale) of each miRNA is plotted against its fold change (on a log2 scale). In **a** and **b**, each circle represents one miRNA; miRNAs with *P* values less than 0.05 are illustrated by solid circles. (**c**) Normalized miRNA expression (*n*=5 slices from three rats for each condition) in rat hippocampal slices stimulated with high-frequency stimulation. (**d**) Normalized miRNA expression (*n*=4–7 experiments for each condition) in cultured rat hippocampal neurons stimulated with TEA. In **c** and **d**, data are presented as mean±s.e.m. Student's *t*-test or Mann–Whitney *U*-test (determined by the distribution and variance of the data) was used for comparison between control and stimulated samples in **c** and **d**; *P<0.05, **P<0.01, ***P<0.001.

Since short-lasting LTP persists for no more than 2 h, and long-lasting LTP lasts longer than 2 h (refs 5, 6), the processes regulating protein synthesis are likely engaged within 2 h after LTP induction. Hence, at 90 min after LTP induction, we analysed miRNAs isolated from the CA1 region by deep sequencing. Control miRNAs were isolated from the hippocampal CA1 area of sham-treated slices (from the same mice). On average 0.47 million quality-filtered reads were obtained from each sample, and 64∼77% of them were mapped to mature miRNAs. Of the 1,941,171 mapped reads from all 6 samples (Supplementary Table 1), we identified 372 miRNAs. The number of detected miRNAs is in line with earlier reports that ∼300 miRNAs are expressed in the rodent brain^10^,^17^. To avoid statistical bias introduced by large inter-sample variations in the abundance of rare miRNAs, we included only miRNAs with ≥20 reads in each sample in our differential expression analysis (Fig. 1; Supplementary Fig. 2).

All miRNAs with *P* values <0.05 were changed more than twofold (Fig. 1b), suggesting consistency in the statistical and biological significance of the sequencing result. Of the 12 miRNAs changed in LTP, 6 were down- and 6 were upregulated (Fig. 1a,b). All upregulated miRNAs belong to the let-7 family, and two of the downregulated miRNAs (miR-384-5p and miR-30e) belong to the same miRNA family.

To validate the deep-sequencing results, we analysed miRNA expression using quantitative reverse transcription (qRT)–PCR in an independent set of slices (from 2- to 3–week-old rats) that were sham-treated or stimulated by high-frequency stimulation. Rat slices were selected for validation because rat brains are larger than mouse brains and therefore commonly used in slice physiology. Consistent with deep-sequencing results, our qRT–PCR analysis showed that in the CA1 region of stimulated slices, let-7a increased, while miR-26a, miR-384-5p and miR-124 decreased (Fig. 1c).

To test whether other LTP induction protocols also cause miRNA changes, we treated cultured rat hippocampal neurons with the potassium channel blocker tetraethylammonium chloride TEA (25 mM, 15 min), which induces translation-dependent long-lasting LTP (Supplementary Fig. 1b) that shares expression mechanisms with electrical LTP^18^,^19^. miRNAs were assessed with qRT–PCR at 90 min post TEA treatment. TEA-induced changes in let-7a, miR-26a and miR-384-5p were comparable to those detected in electrically stimulated hippocampal slices (Fig. 1d). miR-124 was also decreased by TEA treatment, although not to a statistically significant level (Fig. 1d). Hence, the miRNA expression changes induced by either electrical or chemical induction of LTP in rat hippocampal neurons are largely consistent with those in the mouse hippocampus.

Taken together, these results indicate that a subset of miRNAs is changed in expression after LTP induction.

### Gene-GO analysis of differentially expressed miRNAs

To systematically evaluate how changes in the miRNA transcriptome during LTP affect gene expression and cellular activities, we conducted a miR-gene-Gene Ontology (GO) enrichment analysis (as reported previously^20^) for miRNAs differentially expressed in LTP. Target genes with *P* values <5 × 10^−4^ and GO terms with *P* values <0.05 following this test were considered significantly enriched (Supplementary Tables 2 and 3). The miR-gene-GO enrichment analysis shows that following LTP induction, the bidirectional changes in the miRNA transcriptome affect a broad range of cellular activities. The enriched GO terms related to the structural and functional plasticity of synapses are shown in Supplementary Fig. 3.

### LTP maintenance requires decreases in miR-26a and miR-384-5p

Since the effect size of miRNAs on LTP are likely to be positively correlated with their fold changes, we examined the roles of miR-384-5p (with the largest fold change and smallest *P* value) and miR-26a (with the second-largest fold change and the highest abundance among downregulated miRNAs) in LTP. As both miRNAs' expression was reduced in LTP, to perturb their changes caused by LTP induction, we transduced cultured hippocampal slices with lentivirus expressing these two miRNAs. Our lentivirus primarily infected excitatory neurons (Supplementary Fig. 4a). To determine the level of miRNA overexpression, we isolated small RNAs from the CA1 region of slices transduced with lentivirus for qRT–PCR analysis of miRNAs, and found that miR-26a and miR-384-5p were increased by 30∼40% in miR-26a and miR-384-5p virus-transduced slices (Supplementary Fig. 4b).

**Figure 2:**
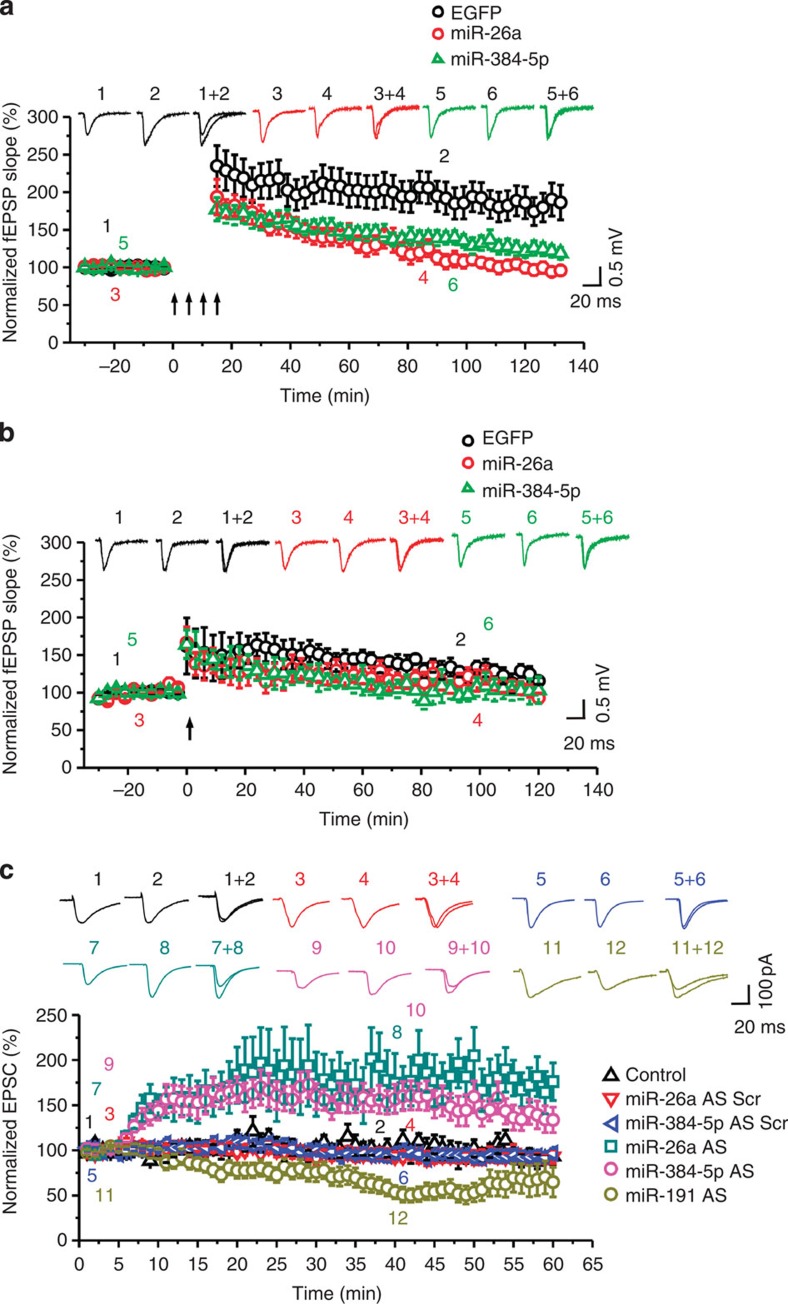
The downregulation of miR-26a and miR-384-5p is required for the maintenance of protein synthesis-dependent LTP. (**a**) Protein synthesis-dependent LTP induced by four trains of tetanic stimulation. (**b**) Protein synthesis-independent LTP induced by a single train of tetanic stimulation. In **a** and **b**, the fEPSP slope normalized to the baseline prior to stimulation was plotted as mean±s.e.m.; *n*=5–12 slices for each condition. (**c**) The effect of infusing antisense oligonucleotides against miR-26a, miR-384-5p or miR-191, and mutated miR-26a and miR-384-5p anti-miRs (all at 2 μM) on EPSCs; *n*=7–9 slices for each condition. Data are presented as mean±s.e.m.

fEPSPs were recorded in the CA1 region by stimulating two independent pathways on either the CA3 or the subicular side of the recording electrode. Only the CA3–CA1 pathway was stimulated for LTP induction. Ten minutes after stimulation, the enhancement of fEPSPs in the CA3–CA1 pathway was comparable in miR-26a (175.6±15.0% of baseline, *P*=0.61 versus control), miR-384-5p (171.6±12.7% of baseline, *P*=0.39 versus control) and control virus-transduced slices (218.9±25.3% of baseline)(Fig. 2a; Supplementary Table 4). By contrast, at 120 min post tetanization, LTP (control: 186.4±23.2% of baseline) was greatly reduced by transduction of the miR-26a (95.7±6.7% of baseline, *P*=0.0001 versus control) or miR-384-5p (118.7±7.9% of baseline, *P*=0.005 versus control) virus (Fig. 2a; Supplementary Table 4). fEPSPs elicited by stimulating the control pathway were stable throughout the recording period in all conditions (Supplementary Fig. 4c).

By contrast, one tetanic stimulation-induced LTP, which is not dependent on protein synthesis^21^,^22^, was comparable in control slices and those transduced with the miR-26a or miR-384-5p virus (Fig. 2b; Supplementary Table 5). Basal synaptic transmission and the ratio of α-amino-3-hydroxy-5-methyl-4-isoxazolepropionic acid (AMPA) and N-methyl-D-aspartate (NMDA) receptor-mediated currents (EPSC_AMPA_/EPSC_NMDA_) were also intact in slices transduced with the miR-26a or miR-384-5p virus (Supplementary Fig. 4d,e). Hence, the reduction of miR-26a and miR-384-5p in LTP is required specifically for the maintenance, but not induction, of protein synthesis-dependent LTP.

We also tested whether reducing miR-26a and miR-384-5p expression is sufficient to enhance synaptic strength. Antisense oligonucleotides against miR-26a or miR-384-5p (anti-miRs) were loaded directly into CA1 neurons through the whole-cell-recording pipettes. EPSCs were increased by anti-miR infusion (Fig. 2c; Supplementary Table 6). By contrast, infusion of anti-miR-26a or anti-miR-384-5p oligonucleotides mutated in the seed region had no effect on EPSCs (Fig. 2c; Supplementary Table 6), while infusion of antisense oligonucleotides against miR-191 (downregulated in LTD^20^) caused a decrease in EPSCs (Fig. 2c; Supplementary Table 6). Hence, the downregulation of miR-26a and miR-384-5p is sufficient to strengthen synaptic transmission.

Taken together, these findings indicate that long-lasting LTP requires downregulation of miR-26a and miR-384-5p.

### RSK3 is a target gene of miR-26a and miR-384-5p

To determine the mechanism by which miR-26a and miR-384-5p regulate LTP, we searched the enriched target genes identified by our miR-gene-GO enrichment analysis for genes that may mediate these two miRNAs' function. We found that 90 kDa *RSK3*, a computationally predicted target gene of both miR-26a and miR-384-5p, is enriched by miRNAs downregulated in LTP (Supplementary Table 2). The RSK family of protein kinase regulates translation by phosphorylating various components, such as rpS6, of translational control pathways^23^. Since translation is involved in LTP, we tested whether RSK3 mediates the effect of miR-26a and miR-384-5p on LTP.

**Figure 3:**
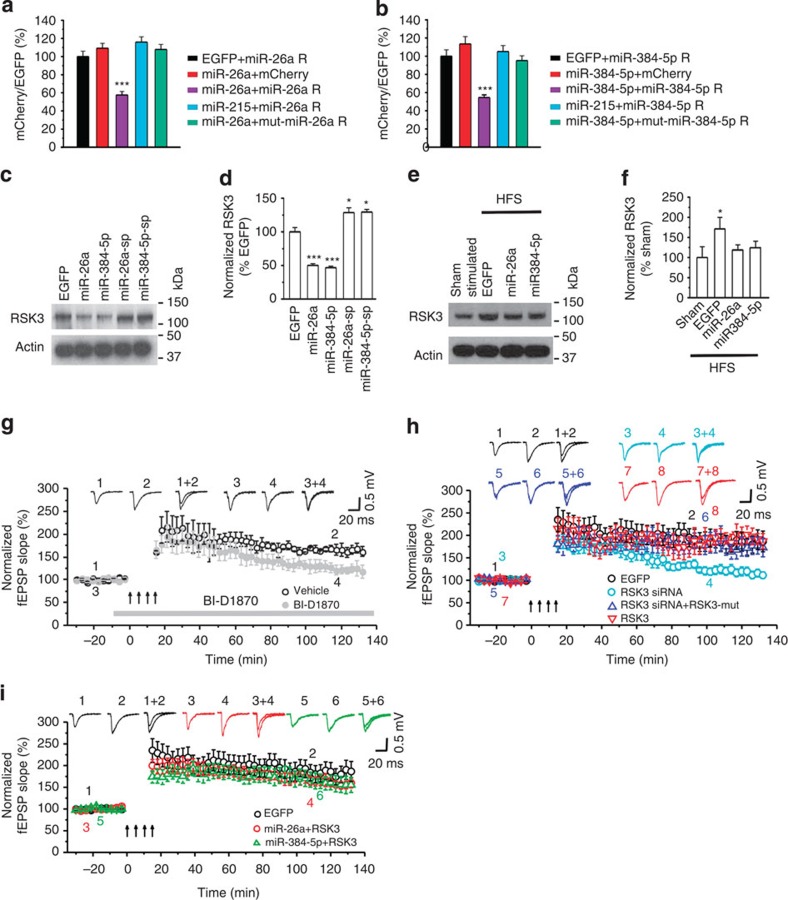
RSK3 mediates the effect of miR-26a and miR-384-5p on LTP. (**a**,**b**) Cultured hippocampal neurons were transfected with designated reporter constructs at DIV14 and fixed for image acquisition at DIV17; *n*=13–15 neurons for each group; data are presented as mean±s.e.m.; Kruskal–Wallis and Mann–Whitney *U*-tests are used for statistical analysis; ***P<0.001; scale bar, 20 μm. (**c**–**f**) Cultured hippocampal slices were transduced with a designated lentivirus, unstimulated (**c**,**d**), sham-stimulated (**e**,**f**) or stimulated with high-frequency stimulation (HFS; **e**,**f**) and immunoblotted for RSK3; data are presented as mean±s.e.m.; *n*=4–5 slices for each condition; Kruskal–Wallis and Mann–Whitney *U*-tests are used for statistical analysis; *P<0.05, ***P<0.001. (**g**–**i**) Cultured hippocampal slices were treated with vehicle or BI-D1870 or transduced with designated lentivirus, and stimulated for LTP induction; the fEPSP slope normalized to the baseline prior to stimulation was plotted as mean±s.e.m.; *n*=5–7 slices for each condition; one-way analysis of variance and Student's *t*-test are used for normally distributed data with equal variance, and Kruskal–Wallis and Mann–Whitney *U*-tests are used for non-normally distributed data with unequal variance for statistical analysis.

We first tested whether RSK3 is a true target of miR-26a and miR-384-5p. We generated miR-26a and miR-384-5p reporter constructs by inserting the sequences surrounding their predicted binding sites in the 3′ UTR of RSK3 behind the destabilized *mCherry* gene. These reporter constructs were co-transfected with plasmids expressing miRNAs (along with enhanced green fluorescent protein (EGFP)) into cultured hippocampal neurons (DIV14). At 2∼3 days after transfection, in neurons co-transfected with the miR-26a or miR-384-5p constructs and their corresponding reporters, mCherry expression was significantly lower than in cells transfected with the EGFP and the miR-26a or miR-384-5p reporter (Fig. 3a,b; Supplementary Fig. 5). Deleting the miR-26a- and miR-384-5p-binding sites or mutating the sequence base-pairing with the seed region of miR-26a and miR-384-5p in the reporter construct abolished this effect (Fig. 3a,b; Supplementary Fig. 5). By contrast, miR-215, which is not predicted to bind to RSK3 mRNAs (TargetScan and miRanda) did not affect expression of the miR-26a and miR-384-5p reporters (Fig. 3a,b; Supplementary Fig. 5).

To test whether miR-26a and miR-384-5p bind to RSK3 mRNAs under physiological conditions, we analysed RNAs associated with the RNA-induced silencing complex (RISC) by using the crosslinking and immunoprecipitation assay. RNAs and proteins of primary cortical neurons (10 days after transduction with lentivirus-expressing Flag-tagged argonoute 2, Ago 2, an miRNA-binding protein of the RISC) were crosslinked by ultraviolet irradiation, and pulled down with an anti-Flag antibody. Using RT–PCR, we detected miR-26a, miR-384-5p and RSK3 mRNAs in the immunoprecipitated RISC (cycle threshold or Ct: miR-26a=29.93±0.26, miR-384-5*P*=29.91±0.29, RSK3=29.76±0.13; *n*=6 samples from two pull-down experiments for each condition). Hence, miR-26a and miR-384-5p interact with RSK3 mRNAs in neurons.

To test whether miR-26a and miR-384-5p regulate endogenous RSK3 protein expression, we transduced hippocampal slices with lentivirus-expressing miR-26a, miR-384-5p or sponges that bind to and sequester them. RSK3 protein expression in the CA1 region was reduced by the miR-26a and miR-384-5p virus, and was increased by the sponge virus (Fig. 3c,d; Supplementary Fig. 11), indicating that miR-26a and miR-384-5p repress the expression of endogenous RSK3.

Taken together, these findings indicate that RSK3 is a true, physiological target gene of miR-26a and miR-384-5p.

### RSK3 mediates the effect of miR-26a and miR-384-5p on LTP

We next examined whether RSK3 regulates LTP. First we tested whether RSK3 expression is altered in LTP. We induced LTP in hippocampal slices by stimulating the Schaffer collateral pathway with four tetanic stimulations, and then removed the CA1 region 90 min later. The level of RSK3 protein was elevated in stimulated slices (Fig. 3e,f; Supplementary Fig. 11). This increase can be obliterated by transduction of the miR-26a or miR-384-5p virus (Fig. 3e,f; Supplementary Fig. 11). The expression of the miR-26a and the miR-384-5p reporters, moreover, were increased by TEA treatment in cultured hippocampal neurons (Supplementary Fig. 6). Hence, the downregulation of miR-26a and miR-384-5p during LTP leads to an increase in RSK3.

To test whether RSK3 plays a role in LTP, we inhibited RSK with BI-D1870 (200 nM) during and after LTP induction in cultured hippocampal slices. The enhancement of fEPSPs in the CA1 region remained unaffected at 10 min, but was reduced at 120 min after BI-D1870 treatment (control: 161.7±10.5% of baseline; BI-D1870: 111.9±9.1%, *P*=0.013; Fig. 3g; Supplementary Table 7). Long-lasting LTP, however, was recovered after BI-D1870 was washed out (Supplementary Fig. 7a; Supplementary Table 8), indicating that BI-D1870 specifically blocks LTP maintenance rather than impairing synaptic transmission. Hence, RSK3 activity is required for long-lasting LTP.

To confirm the involvement of RSK3 in LTP, we transduced hippocampal slices with lentivirus-expressing short interfering RNAs (siRNAs) that effectively and specifically knock down RSK3 expression (Supplementary Fig. 8). In slices transduced with the RSK3 siRNA virus, four tetanization-induced LTP remained unaffected at 10 min, but was reduced at 120 min after stimulation (control: 186.4±23.2% of baseline; RSK3 siRNA: 111.3±7.4%, *P*=0.008 versus control; Fig. 3h; Supplementary Fig. 7b; Supplementary Table 9). To test whether the effect of RSK3 siRNA on LTP is caused by RSK3 knockdown, we transduced hippocampal slices with the RSK3 siRNA virus along with lentivirus-expressing RSK3 resistant to the RSK3 siRNA (harbouring silent mutations at the siRNA-binding site). While RSK3 overexpression alone had no effect on LTP, co-transduction of the RSK3 virus abolished the effect of RSK3 siRNA on LTP (at 120 min post stimulation, RSK3: 187.0±16.9% of baseline; RSK3 siRNA plus RSK3: 172.8±20.6% of baseline; *P*=0.73 for RSK3 siRNA plus RSK3 versus control; Fig. 3h; Supplementary Fig. 7b; Supplementary Table 9). These results confirm that RSK3 knockdown blocks LTP maintenance. Hence, RSK3 is required for the maintenance, but not for the induction, of LTP.

To determine whether RSK3 mediates the effect of miR-26a and miR-384-5p on LTP, we transduced cultured hippocampal slices with the miR-26a or miR-384-5p lentivirus along with the RSK3 expressing lentivirus to compensate for the repression of RSK3 expression by miR-26a and miR-384-5p. Unlike the inhibition of long-term LTP by miR-26a and miR-384-5p (Fig. 2a), co-transduction of the miR-26a and the RSK3 virus (160.0±11.1% of baseline, *P*=0.35 versus 186.4±23.2% % in controls), and co-transduction of the miR-384-5p and the RSK3 virus (155.4±17.3% of baseline, *P*=0.43 versus control) did not change LTP at 120 min after induction (Fig. 3i; Supplementary Fig. 7c; Supplementary Table 10). Hence, miR-26a and miR-384-5p regulate LTP by controlling RSK3 expression.

Taken together, these findings indicate that LTP maintenance requires miR-26a- and miR-384-5p-dependent upregulation of RSK3.

### RSK3 contributes to increased rpS6 phosphorylation

**Figure 4:**
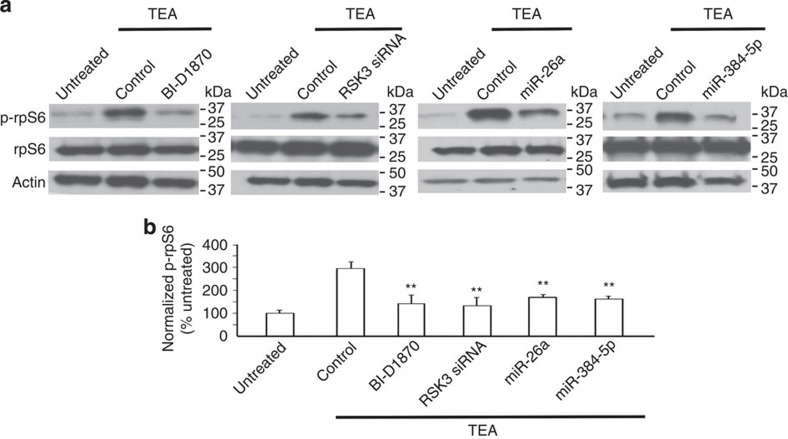
Increased rpS6 phosphorylation caused by the expression change to miR-26a, miR-384-5p and RSK3 in LTP. Primary cortical neurons (DIV4) were transduced with lentivirus-expressing EGFP (as the control), RSK3 siRNA, miR-26a or miR-384-5p. At 2 weeks after transduction, neurons were treated with TEA (25 mM, 15 min) and harvested at 90 min after treatment for immunoblotting. BI-D1870 was added to neural medium at 5 min before TEA treatment. (**a**) Representative blots. (**b**) Quantification of **a**. Data are presented as mean±s.e.m. *n*=4–5 experiments for each condition. Kruskal–Wallis and Mann–Whitney *U*-tests are used for statistical analysis. **P<0.01.

To further delineate the mechanisms by which miR-26a and miR-384-5p regulate LTP, we examined whether RSK3's substrates are affected by changes in its expression. Primary cortical neurons (DIV17) were treated with TEA (25 mM, 15 min), and lysed at 90 min after treatment for immunoblotting against rpS6, a substrate of the RSK family^23^. While the total rpS6 level remained unchanged, phosphorylated rpS6 was increased by 2.9±0.3-fold (Fig. 4; Supplementary Fig. 11).

To test whether RSK3 is responsible for the increase in rpS6 phosphorylation, we transduced neurons with the RSK3 siRNA virus or treated them with BI-D1870. TEA-induced increases in phosphorylated rpS6 were obliterated by the RSK3 siRNA and the RSK inhibitor (Fig. 4; Supplementary Fig. 11). To determine whether miR-26a and miR-384-5p contribute to TEA-induced rpS6 phosphorylation, we transduced neurons with the miR-26a or the miR-384-5p lentivirus. Expression of both miRNAs abolished TEA's effects on rpS6 phosphorylation (Fig. 4; Supplementary Fig. 11).

Taken together, these results indicate that elevated RSK3 expression subsequent to the downregulation of miR-26a and miR-384-5p leads to increased phosphorylation of rpS6, and therefore presumably affects translation.

### miR-26a and miR-384-5p are essential for spine enlargement

LTP is accompanied by spine formation and enlargement^24^. To determine whether miR-26a and miR-384-5p are also involved in spine plasticity, we analysed dendritic spines prior to and after TEA treatment in primary hippocampal neurons. To visualize spines, we transfected DIV14 neurons with the venus construct along with a plasmid expressing miR-26a, miR-384-5p or miR-215 (as a control).

**Figure 5:**
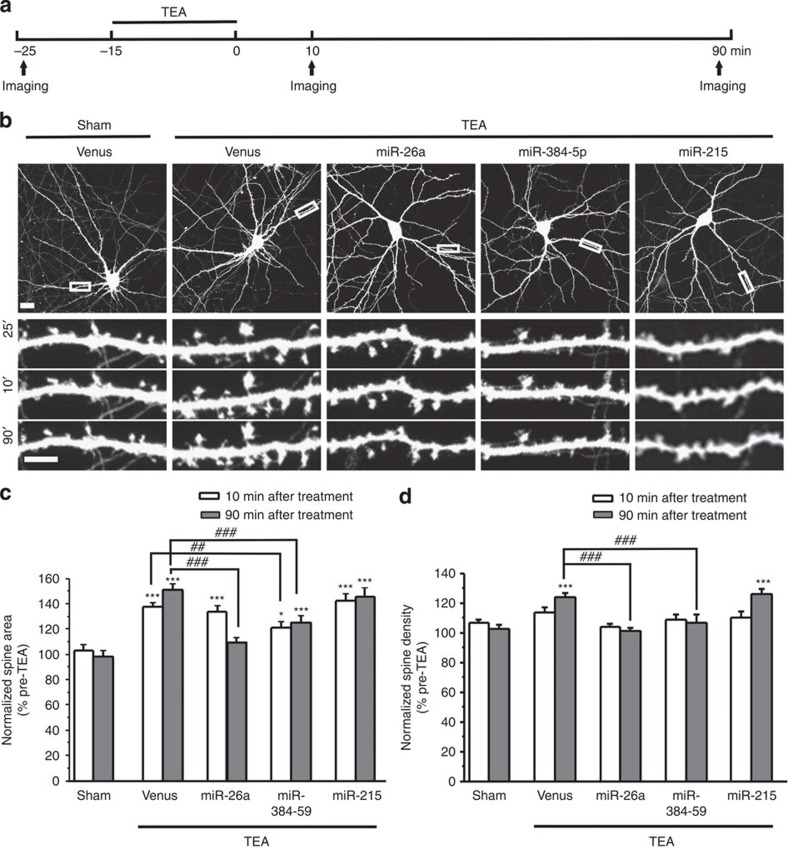
The downregulation of miR-26a and miR-384-5p is required for spine plasticity associated with LTP. (**a**) Experimental design. (**b**) Representative images. (**c**,**d**) Quantification of **b**. *n*=10–18 neurons for each condition. Data are presented as mean±s.e.m. Kruskal–Wallis and Mann–Whitney *U*-tests are used for statistical analysis. *P<0.05, *** and ^##^*P*<0.01, ^###^*P*<0.001. Scale bar, 20 μm for low-magnification images and 5 μm for high-magnification images.

At 2∼3 days after transfection, neurons were treated with TEA (25 mM, 15 min) and imaged before and after treatment. TEA treatment caused a rapid and sustained expansion of dendritic spines (increased by 37.4±3.6% (*P*=2.14 × 10^−5^) at 10 min and 51.4±4.5% (*P*=5.8 × 10^−8^) at 90 min after treatment; Fig. 8). Spine number was also increased by TEA treatment (up by 14.0±3.2% (*P*=0.12) at 10 min and 24.1±3.0% (*P*=0.03) at 90 min after treatment; Fig. 5). Transfection of the miR-215 construct did not affect TEA-induced changes in spines (Fig. 5).

TEA-induced spine formation was obliterated in both miR-26a- and miR-384-5p-transfected neurons (Fig. 5). In miR-26a-transfected neurons, spine size was increased to a magnitude comparable to that found in control neurons at 10 min after TEA treatment, but by 90 min post stimulation, spines returned to their pre-stimulation sizes (Fig. 5). In neurons transfected with miR-384-5p, by contrast, TEA-induced spine enlargement was reduced at both 10 and 90 min after TEA treatment (Fig. 5). These results show that during LTP, miR-384-5p inhibits the induction and miR-26a restricts the duration of spine enlargement. Without TEA treatment, the density and size of dendritic spines were indistinguishable in miR-26a-, miR-384-5p-, miR-215- and control plasmid-transfected cells (Supplementary Fig. 9). Moreover, knockdown of miR-26a or miR-384-5p had no effect on spine area or density (Supplementary Fig. 9). Hence, both miR-26a and miR-348-5p regulate activity-induced (but not baseline) morphogenesis of dendritic spines.

### RSK3 mediates the effects of miR-26a on spine plasticity

**Figure 6:**
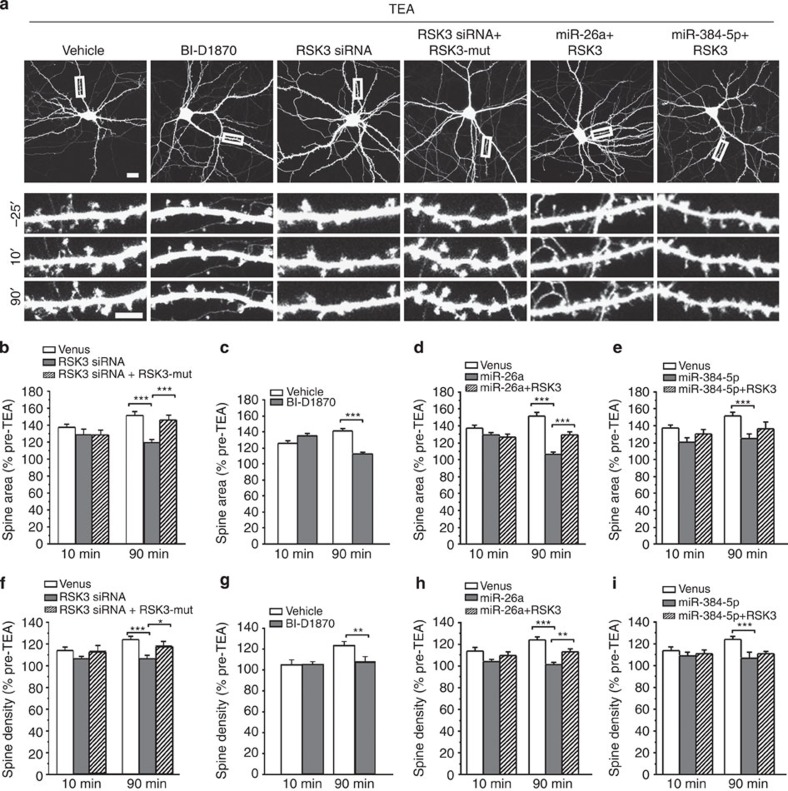
RSK3 mediates the effect of miR-26a on spine plasticity. (**a**) Representative images. (**b**–**i**) Quantification of spine area and spine density. For BI-D1870 treatment, BI-D1870 was added to the medium at 5 min before TEA treatment. *n*=13–16 neurons for each condition. Data are presented as mean±s.e.m. Kruskal–Wallis and Mann–Whitney *U*-tests are used for statistical analysis. *P<0.05, **P<0.01, ***P<0.001. Scale bar, 20 μm for low-magnification images and 5 μm for high-magnification images.

Having found that miR-26a and miR-384-5p both target RSK3 to regulate LTP, we next tested whether they also target RSK3 for spine remodelling. First, we tested whether RSK3 is involved in spine plasticity. We transfected cultured hippocampal neurons (DIV14) with the RSK3 siRNA construct, and treated them with TEA (25 mM, 15 min) at 2∼3 days after transfection. Transfection of the RSK3 siRNA construct obliterated TEA-induced spine enlargement at 90 min, but not at 10 min post stimulation (Fig. 6a,b). TEA-induced spine formation was also blocked by transfection of the RSK3 siRNA construct (Fig. 6a,f). The blockade of TEA-induced spine formation and enlargement by RSK3 siRNA, however, was inhibited by co-transfection with a construct expressing siRNA-resistant RSK3, confirming that the effects of RSK siRNA are specific (Fig. 6a,b,f). Likewise, BI-D1870 treatment abolished TEA-induced spine formation and long-lasting spine enlargement (Fig. 6a,c,g). These results indicate that RSK3 is required for spine formation and for the maintenance (but not the induction) of spine enlargement induced by TEA treatment.

Next, to test whether RSK3 mediates the function of miR-26a and miR-384-5p in spine plasticity, we transfected cultured hippocampal neurons (DIV14) with the miR-26a or miR-384-5p construct along with a construct expressing RSK3. Neurons were treated with TEA (25 mM, 15 min) at 2∼3 days after transfection. Transfection of the RSK3 construct attenuated miR-26a's inhibition of TEA-induced spine formation and long-lasting spine enlargement, but left miR-384-5p's effects on spine formation and enlargement unchanged (Fig. 6a,d,e,h,i). These results indicate that miR-26a constrains spine remodelling by inhibiting the expression of RSK3.

Taken together, these findings indicate that RSK3 is a target gene that mediates the effect of miR-26a downregulation on TEA-induced spine formation and prolonged spine enlargement.

### GluN2A-dependent regulation of miR-26a and miR-384-5p in LTP

**Figure 7:**
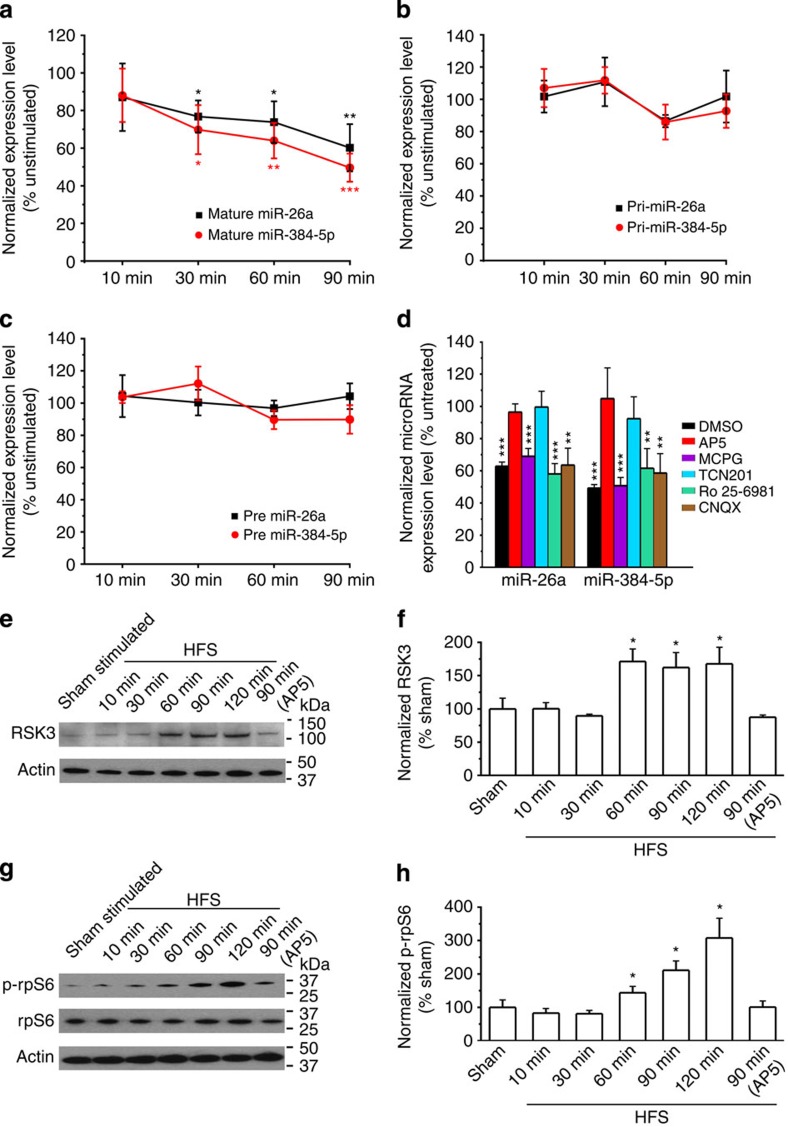
miR-26a and miR-384-5p are regulated at post-transcriptional levels by GluN2A in LTP. LTP was induced in hippocampal slices by stimulating the Schaffer collateral pathway with high-frequency stimulation. The CA1 region was removed at indicated time points after stimulation for miRNA (**a**,**d**), pri-miRNA (**b**), pre-miRNA (**c**) and protein analyses (**e**–**h**). *n*=8–9 slices (qRT–PCR) or 4–5 slices (immunoblot) for each condition. Data are presented as mean±s.e.m. Kruskal–Wallis and Mann–Whitney *U*-tests are used for statistical analysis between unstimulated (**a**–**d**) or sham-stimulated (**e**–**h**) slices and stimulated slices harvested at different post-stimulation time points. *P<0.05, **P<0.01, ***P<0.001. In **a**–**c**, black asterisks indicate statistically significant differences for mature, pri- and pre-miR-26a, and orange asterisks indicate those for mature, pri- and pre-miR-384-5p.

To explore how miR-26a and miR-384-5p are regulated in LTP, we first assessed the temporal dynamics and biogenesis of miR-26a and miR-384-5p in LTP. Acute hippocampal slices were stimulated at the Schaffer collateral pathway with four tetanizations for LTP induction. The CA1 region was removed after stimulation for qRT–PCR. Both miR-26a and miR-384-5p exhibited a downward trend, and were significantly reduced by 30 min post stimulation (Fig. 7a). Pri- and pre-miR-26a and -miR-384-5p, by contrast, were not changed significantly by LTP induction (Fig. 7b,c). Conversely, RSK3 protein and phosphorylated rpS6 increased after LTP induction (Fig. 7e–h; Supplementary Fig. 11). These results indicate that the downregulation of miR-26a and miR-384-5p in LTP is post-transcriptional.

Since NMDA, metabotropic glutamate (mGlu) and AMPA receptors play important roles in the induction and expression of synaptic plasticity in the hippocampus^25^,^26^,^27^, we tested whether these receptors are involved in the regulation of miR-26a and miR-384-5p in LTP. We added AP5 ((2R)-amino-5-phosphonovaleric acid, 100 μM, NMDA receptor antagonist), MCPG (500 μM, mGluR antagonist) or CNQX (10 μM, AMPA receptor blocker) to the perfusion solution during and after LTP induction, and removed the CA1 region at 90 min after stimulation for qRT–PCR. The reduction of miR-26a and miR-384-5p in LTP was blocked by AP5, but not by MCPG or CNQX (Fig. 7d). Hence, miR-26a and miR-384-5p are regulated by NMDA receptors in LTP.

In hippocampal neurons, NMDA receptors are composed of GluN1 and GluN2 (GluN2A and GluN2B) subunits^28^. To test which GluN2 subunit regulates miRNAs in LTP, we treated hippocampal slices with the GluN2A antagonist TCN 201 (10 μM) or the GluN2B antagonists Ro 25-6891 (3 μM) during and after LTP induction, and measured miRNAs in the CA1 region at 90 min post induction. The GluN2A, but not the GluN2B, antagonist abolished the decreases in miR-26a and miR-384-5p (Fig. 7d).

### Local regulation of dendritic miR-26a and miR-384-5p in LTP

**Figure 8:**
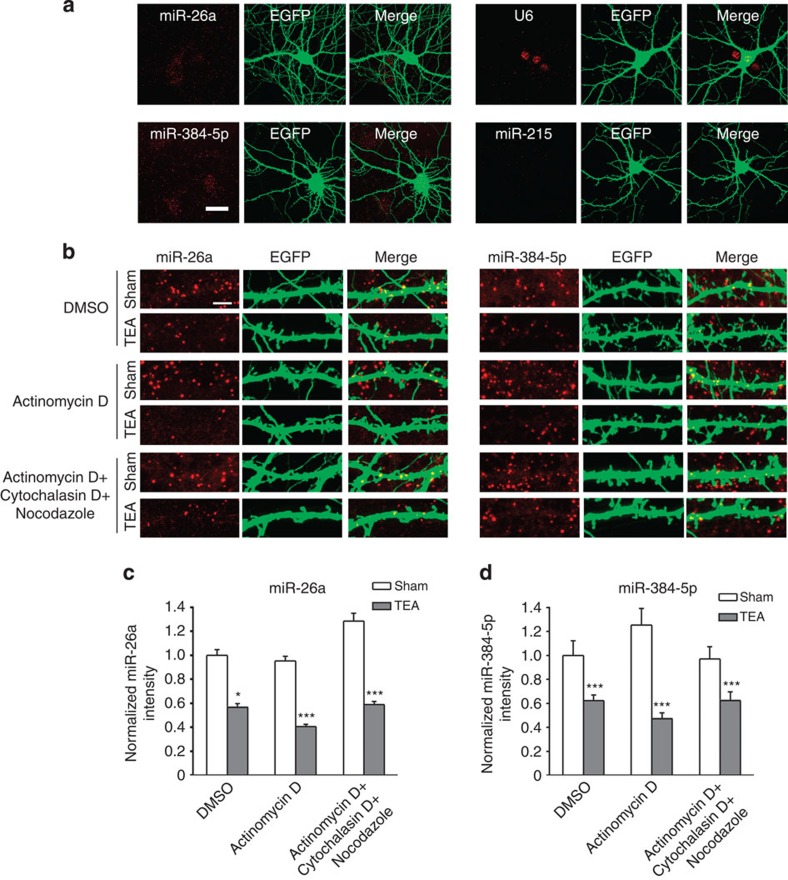
miR-26a and miR-384-5p are regulated locally in dendrites in LTP. Cultured hippocampal neurons were transfected with the EGFP construct (for visualization of transfected neurons), treated with TEA (25 mM, 15 min) at 3 days after transfection and fixed at 90 min after stimulation for *in situ* hybridization. (**a**) The subcellular distribution of miR-26a and miR-384-5p. (**b**–**d**) The effect of TEA treatment on dendritic miR-26a and miR-384-5p; representative images are in **b**, quantification of **b** is in **c** and **d**; *n*=18–24 neurons for each condition. Data are presented as mean±s.e.m. Mann–Whitney *U*-test is used for statistical analysis. *P<0.05, ***P<0.001.

Since miRNAs can be regulated locally in dendrites during LTD^20^, we next tested whether miR-26a and miR-384-5p are regulated locally in LTP. We first examined the subcellular distribution of miR-26a and miR-384-5p using *in situ* hybridization (ISH). Hippocampal neurons (DIV14) were transfected with the EGFP construct for visualization of dendrites. At 3 days after transfection, locked nucleic acid probes were used for miRNA ISH. miR-26a and miR-384-5p were detected in both soma and dendrites (Fig. 8a). By contrast, the U6 small nuclear RNA was found primarily in the soma, and miR-215 was barely detected (Fig. 8a), confirming the specificity of our ISH assay. These results indicate that miR-26a and miR-384-5p are indeed localized in dendrites.

To test whether dendritic miR-26a and miR-384-5p are changed in LTP, we treated hippocampal neurons (DIV17, 3 days after transfection with the EGFP construct) with TEA (25 mM, 15 min), and fixed them at 90 min post stimulation for ISH. Both miRNAs were downregulated in dendrites by TEA treatment (Fig. 8b–d). This decrease was not affected by inhibiting transcription and active transport of miRNAs with the transcription inhibitor actinomycin D, the actin polymerization inhibitor cytochalasin D and the microtubule polymerization inhibitor nocodazole (all at 10 μM; Fig. 8b–d). Hence, miR-26a and miR-384-5p can be regulated locally in dendrites.

### Downregulation of miR-26a and miR-384-5p in learning

**Figure 9:**
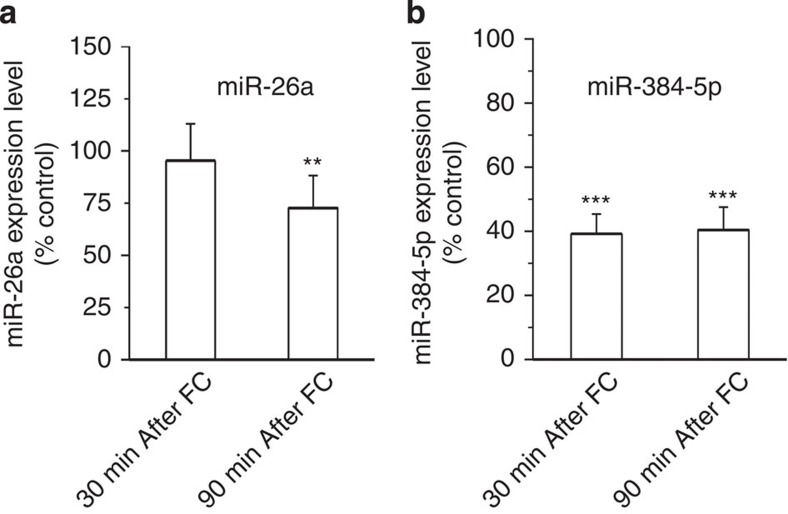
miR-26a and miR-384-5p in the hippocampus decrease after fear conditioning. Mice (8–9 weeks of age) were subjected to fear conditioning. The hippocampus was removed at 30 and 90 min after fear conditioning for miRNA analysis by qRT–PCR. The expression levels of miR-26a (**a**) and miR-384-5p (**b**) were normalized to those in untreated control mice. Data are presented as mean±s.e.m., *n*=4 mice for each condition. Mann–Whitney *U*-test is used for statistical analysis. **P<0.01, ***P<0.001.

LTP is a cellular mechanism that can be engaged by cognitive processing such as learning^29^,^30^,^31^. To test whether miR-26a and miR-384-5p are involved in learning, we analysed the expression of miR-26a and miR-384-5p in the hippocampus after fear conditioning, a form of hippocampus-dependent associative learning that induces hippocampal LTP^32^,^33^,^34^,^35^. Mice (8–9 weeks of age) were placed in a chamber to receive tones paired with electrical foot shock. At 30 and 90 min after fear conditioning, mice were euthanized and the hippocampus was removed for miRNA analysis. miR-26a was reduced at 90 min, while miR-384-5p was reduced at both 30 and 90 min after fear conditioning (Fig. 9). Hence, fear conditioning induces decreases in miR-26a and miR-384-5p, presumably facilitating LTP and fear memory formation.

### Let-7a is required for long-lasting LTP and spine plasticity

**Figure 10:**
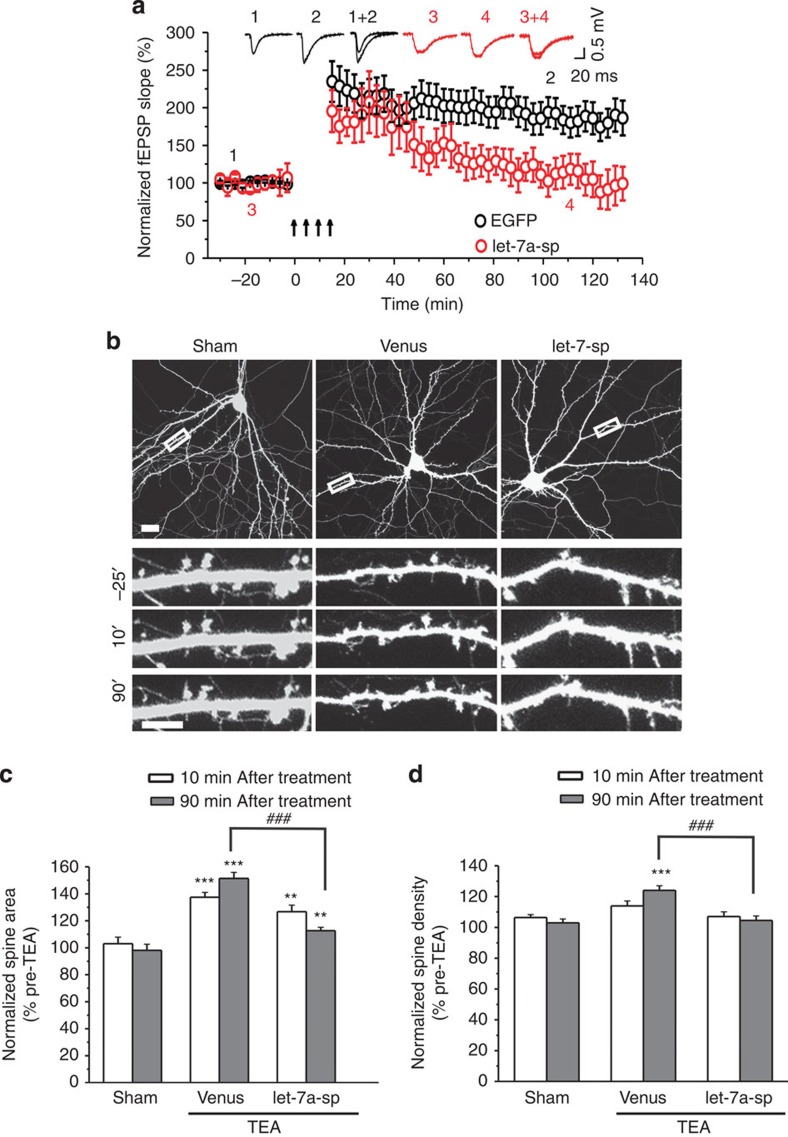
Let-7a is required for spine plasticity associated with LTP. (**a**) Cultured hippocampal slices were transduced with lentivirus-expressing let-7a sponge and stimulated with high-frequency stimulation for LTP induction; *n*=5 slices. The fEPSP slope normalized to the baseline prior to stimulation is plotted as mean±s.e.m. In **b**–**d**, cultured hippocampal neurons were co-transfected with the let-7a sponge and the venus (for visualization of transfected neurons) construct at DIV14 and stimulated with TEA (25 mM, 15 min) at DIV17. (**b**) Representative images. (**c**,**d**) Quantification of **b**; *n*=12–18 neurons for each condition; data are presented as mean±s.e.m. Kruskal–Wallis and Mann–Whitney *U*-tests are used for statistical analysis. *** and ^###^*P*<0.001, **P<0.01. Scale bar, 20 μm for low-magnification images and 5 μm for high-magnification images.

To investigate the significance of miRNAs upregulated in LTP, we transduced cultured hippocampal slices with lentivirus-expressing sponges binding to let-7a (a member of the let-7 family that is increased in LTP). At 5–7 days after transduction, LTP was induced in the CA1 region by stimulating the Schaffer collateral pathway with four tetanizations, and the subicular-CA1 pathway was used as the control pathway. At 10 min after stimulation, the enhancement of fEPSCs in the CA3–CA1 pathway was comparable in control virus- (218.9±25.3% of baseline) and let-7a-sponge-transduced slices (181.1±27.7% of baseline; *P*=0.65 versus control; Fig. 10a; Supplementary Table 11). Long-lasting LTP, however, was blocked by let-7a knockdown (fEPSCs at 120 min post stimulation, let-7a sponge: 99.1±22.5% of baseline; control: 186.4±23.2% of baseline; *P*=0.045; Fig. 10a; Supplementary Table 11). fEPSPs elicited in the control pathway were stable throughout the recording period in all conditions (Supplementary Fig. 10). These results indicate that let-7a is required for the maintenance, but not for the induction and early phase, of LTP.

To test whether let-7a is also involved in spine plasticity, we transfected cultured hippocampal neurons (DIV14) with the let-7a sponge construct along with the venus construct. The size and density of dendritic spines at 2∼3 days after transfection were comparable in control and let-7a knockdown cells (Supplementary Fig. 9). Transfected neurons were treated with TEA (25 mM, 15 min) to induce spine remodelling. In cells transfected with the let-7a sponge construct, TEA-induced spine enlargement was intact at 10 min, but was obliterated at 90 min post stimulation (Fig. 10b,c). TEA-induced spine formation was also abolished by let-7a knockdown (Fig. 10b,d). Hence, let-7a is necessary for TEA-induced spine formation and long-lasting spine enlargement.

Taken together, these findings indicate that let-7a is essential for long-lasting LTP and structural modification of dendritic spines.

## Discussion

In this study, we identified miRNAs that contribute to the protein synthesis-dependent phase of LTP. Our deep-sequencing and computational analysis systematically assessed the diverse as well as convergent effects of miRNA expression changes during LTP on cell physiology. Using electrophysiology and live imaging techniques, we characterized the functions of select miRNAs in LTP and spine enlargement.

Several lines of evidence from this study show that miRNAs are essential for protein synthesis-dependent LTP and spine enlargement. Electrical stimulations inducing LTP produced robust changes in the miRNA transcriptome, and 12 of the 140 miRNAs we analysed changed more than onefold in expression. Our miR-gene-Go enrichment analysis revealed that these differentially expressed miRNAs target many cellular processes (including synaptic transmission, actin modification, protein phosphorylation, mRNA transport and processing, transcription, translation and gene silencing) that are well recognized to be involved in the modification of synapses and dendritic spines. Moreover, since there are more downregulated than upregulated miRNAs in LTP and miRNAs repress translation, it is predicted that miRNAs contribute to protein synthesis in LTP. This scenario is confirmed by our results that obliteration of the decreases in miR-26a or miR-384-5p expression impaired protein synthesis-dependent LTP.

Our finding of bidirectional changes in miRNA expression is consistent with an earlier study in the dentate gyrus^36^, but contrasts with the result from the study of Park *et al.*^37^ that most miRNAs are upregulated following induction of chemical LTP by TEA. This discrepancy might be due to differences between the two studies in the age of mice and the method of miRNA detection. It is also worth noting that Park *et al.* did not test whether their detected changes in miRNA expression are statistically significant. Our findings that miRNAs regulate long-lasting LTP are consistent with miRNAs' functions in memory formation^38^,^39^, as LTP is an important cellular mechanism for cognition^29^,^30^,^31^. Indeed, our study shows that miR-26a and miR-384-5p are downregulated by hippocampal-dependent fear conditioning^40^,^41^.

Our finding that the *RSK3* gene is the primary target responsible for the effects of both miR-26a and miR-384-5p on LTP suggest that in neurons miRNAs can act cooperatively to regulate target expression. We found that although RSK3 was responsible for miR-26a's effects on, it did not reverse miR-384-5p's inhibition of TEA-induced spine formation. This is likely because miR-26a and miR-384-5p use different mechanisms to regulate spine formation. While miR-26a did not affect the induction of spine enlargement, it did inhibit the maintenance of enlarged spines in a RSK3-dependent manner. miR-384-5p, by contrast, inhibited the initiation of spine enlargement independent of RSK3.

We found that although mature miR-26a and miR-384-5p are reduced in LTP, their pre- and pri-forms are unchanged. These findings suggest that the reduction of miR-26a and miR-384-5p in LTP is likely caused by decay of mature miRNAs (but not by inhibition of miRNA gene transcription or pre-miRNA processing). Furthermore, we found that both miRNAs are localized in dendrites, and blockers of active intracellular transport have no effect on their upregulation by LTP induction in dendrites, suggesting that they can be regulated locally in dendrites in LTP. A reduction of mature miRNAs in the absence of the changes to pri- and pre-miRNAs has also been observed in the dentate gyrus following *in vivo* LTP induction^36^.

In addition to miR-26a and miR-384-5p, our miRNA transcriptome analysis shows that several members of the let-7 family are upregulated. Our functional analyses show that let-7a is also essential for long-lasting LTP and spine plasticity. It is noted that let-7 expression is regulated by brain-derived neurotrophic factor (BDNF), which contributes to enduring modification of synaptic function^42^. Hence, long-lasting LTP is enabled by both up- and downregulation of specific miRNAs.

In summary, we identified miRNAs exhibiting expression changes during LTP and demonstrated that among them, miR-26a, miR-384-5p and let-7a are essential for long-term maintenance of LTP.

## Methods

### Animals

All animal procedures followed the US National Institutes of Health guidelines. Animal using were approved by the National Institute of Mental Health Animal Care and Use Committee.

### DNA constructs and reagents

Using RT–PCR, pre-miRNAs (miR-26a primers: 5′-GAAGATCTCTTCCGTAGCCCCTTCTCTT-3′ and 5′-CCCAAGCTTAGATTGACGGG GGACTCTG-3′; miR-384-5p primers: 5′-GAAGATCTCTTCAAAGTGAACAGCCCAGT-3′ and 5′-CCCAAGCTTAGCTTCTTGAAGGCTTCCTATG-3′ and miR-215 primers: 5′-CGGGATCCCCTCCAAGTGCTCTGTCACCAG-3′ and 5′-CCCAAGCTTAAAAAACAGACTCCCATGGTCTGCT-3′) were amplified from rat mRNAs and then cloned into the pGsuper vector and the pRRLsin.CMV.GFPpre vector (for lentivirus production). Rat RSK3 complementary DNA (cDNA) was obtained by RT–PCR and cloned into the pGW1 vector. The RSK3 siRNA construct was made by inserting annealed oligonucleotides containing a siRNA sequence against RSK3 (5′-GCAGTTACATGGGAACAAT-3′) into the pSuper vector. The reporter constructs were generated by inserting the ∼500-bp fragment surrounding the miRNA-binding sites in the 3′UTR of RSK3 into the pCMV-mCherry-Ds vector behind DS-mCherry. The miRNA-binding sites of miR-26a, miR-384-5p and let-7a sponges were designed as below: miR-26a: 5′-AGCCTATCCTCTATACTTGAA-3′; miR-384-5p: 5′-ACATTGCCTAGTTATGTTTACA-3′ and let-7a: 5′-AACTATACAAGATCTACCTCA-3′. The following reagents were obtained commercially: the COS-7 cell line (ATCC); miR-26a, miR-384-5p and miR-191 antisense oligonucleotide (IDT, the sequence of the seed region was changed to AUUCUAG for mutated miR-26a anti-miR, and to UUCAUGU for mutated miR-384-5p anti-miR); TEA, anisomysin, actinomycin D, cytochalasin, nocodazole, AP5 and Ro 25-6891 from Sigma-Aldrich; TCN 201, CNQX and MCPG from Tocris; BI-D 1870 (Enzo Life Sciences); anti-RSK3 antibody (1:200 for immunoblotting, Abcam, Cat # ab40997), anti-phospho-Ser235/236-S6 ribosomal protein antibody (1:1,000 for immunoblotting, Cell signaling, Cat # 4858), anti-S6 ribosomal protein antibody (1:1,000 for immunoblotting, Cell Signaling, Cat # 2217), anti-Tmod2 (1:3,000 for immunoblotting, Abcam, Cat # ab67407), anti-complexin-1 antibody (1:3,000 for immunoblotting, Synaptic Systems, Cat # 122002), anti-HA antibody (1:1,000 for immunoblotting, Covance, Cat # MMS-101P), anti-Myc antibody (1:1,000 for immunoblotting, CalBiochem, Cat # OP10) and anti-actin antibody (1:5,000 for immunoblotting, Sigma-Aldrich, Cat # A4700), anti-CaMKIIα antibody (1:200 for immunocytochemistry, Life technologies, Cat # 13-7300), anti-PV antibody (1:10,000 for immunocytochemistry, Swant, Cat # PV25), anti-GFAP antibody (1:200 for immunocytochemistry, Millipore, Cat # MAB360), anti-OX42 antibody (1:200 for immunocytochemistry, Abcam, Cat # ab1211).

### Deep sequencing

The hippocampal CA1 region of mice (C57BL/6, 17–19 days of age) was removed and homogenized with a Polytron homogenizer (Kinematica). The mirVana miRNA Isolation Kit (Ambion, Austin, TX, USA) was used to isolate small RNAs. RNA quality was assessed by measuring the RNA integrity number with the Agilent 2100 Bioanalyzer. Denaturing polyacrylamide gel electrophoresis (15%) was used to purify miRNAs (17∼27 nt long) from the small RNA fraction. The Small RNA Sample Prep kit (Illumina) was used to construct deep-sequencing libraries. Briefly, 3′ and 5′ adaptors were sequentially ligated to purified miRNAs, then adaptor-tagged miRNAs were reverse-transcribed and PCR amplified. PCR products were purified by polyacrylamide gel electrophoresis (8%) and sequenced using an Illumina Genome Analyzer II.

### Deep-sequencing data analysis

Sequence data were analysed as described previously^17^. Briefly, raw sequence reads were consolidated by clustering identical sequence reads. Only those containing complete 3′- and 5′-adaptor sequences were used in downstream analysis. Using Bowtie v0.2.1, the remaining sequences were aligned to miRNA hairpin sequences downloaded from miRBase database release ( http://www.mirbase.org/)^43^. Mappable reads were further filtered to remove those that did not map to mature miRNAs. The expression count for each type of miRNA with at least five read counts in at least 50% samples was normalized to the total number of mapped reads for the corresponding sample^44^. The read count was log2 transformed and quantile normalized across all samples. A modified *t*-test^45^ and false discovery rate (FDR) analysis^46^ were applied to identify differentially expressed miRNAs (*P*<0.05).

### miR-gene-GO enrichment analysis

miR-gene-GO enrichment analysis was performed as described previously^17^. The following equation was used for the hypergeometric statistical test in the gene enrichment analysis for each test group.


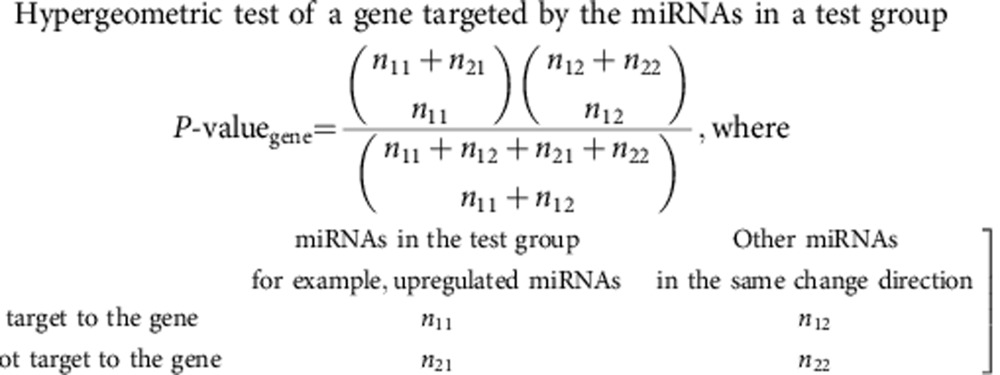


*n*_11_=the number of miRNAs in the test group that target to the gene being tested;

*n*_12_=the number of other detected miRNAs that had the same change direction as those in the test group, and target to the gene being tested, but were not included in the test group because their FDR controlled *P* values were greater than 0.05;

*n*_21_=the number of miRNAs in the group that do NOT target to the gene being tested;

*n*_22_=the number of other miRNAs that had the same change direction as those in the test group, but do NOT target to the gene being tested, and were not included in the test group because their FDR controlled *P* values were greater than 0.05.

The enriched GO terms that annotate the over-represented genes were identified using the conditional hypergeometric test (R package GOStats)^47^. GO terms whose statistical significance was contributed by their significant children terms were removed in the conditional hypergeometric test. GO terms that had *P* values less than 0.05 and were targeted by at least 50% of the total selected miRNAs in the test group were considered statistically enriched. The following equation was used for the test. There were three sets of GO terms: biological process, molecular function and cellular component.


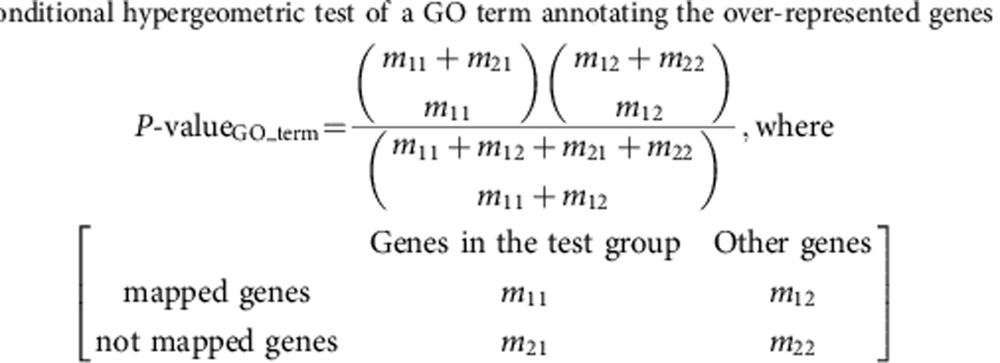


*m*_11_=the number of over-represented genes in the test group that were mapped to the GO term being tested;

*m*_12_=the number of other genes in the GO database that were mapped to the GO term being tested;

*m*_21_=the number of over-represented genes in the test group that were NOT mapped to the GO term being tested;

*m*_22_=the number of other genes in the GO database that were NOT mapped to the GO term being tested.

### Hippocampal slice culture

Hippocampal slices were prepared from rats (but not from mice) because more slices can be obtained from a rat than from a mouse brain due to its larger size. 6–8-day-old Sprague-Dawley rats were decapitated, and the brain was placed immediately in cold cutting solutions (238 mM sucrose, 2.5 mM KCl, 26 mM NaHCO_3_, 1 mM NaH_2_PO_4_, 5 mM MgCl_2_, 11 mM D-glucose and 1 mM CaCl_2_). Hippocampal slices (400 μm) were cut with a vibratome (Leica) and placed on top of semipermeable membrane inserts (Millipore Corporation) in a six-well plate containing culture medium without antibiotics (78.8% minimum essential medium, 20% heat-inactivated horse serum, 25 mM HEPES, 10 mM D-glucose, 26 mM NaHCO_3_, 2 mM CaCl_2_, 2 mM MgSO_4_, 0.0012% ascorbic acid and 1 μg ml^−1^ insulin; pH 7.3; 320–330 mOsm). Medium was changed every 2 days. Slices were transduced with lentivirus at DIV12 and used for electrophysiology at DIV17.

### Electrophysiology

Using standard methods, cultured hippocampal slices were perfused with artificial cerebrospinal fluid (ACSF, pH 7.4, gassed with 95% O_2_/5% CO_2_, composed of in mM: 124 NaCl, 3 KCl, 26 NaHCO_3_, 1.25 NaH_2_PO_4_, 2.5 CaCl_2_, 1.3 MgSO_4_ and 10 D-glucose bubbled with 95% O_2_/5% CO_2_; 30 °C) at the rate of 3 ml min^−1^. Stimulating electrodes were placed at the Schaffer collateral pathway (for LTP induction) and the subicular side of the recording electrode (for the control pathway). Stimuli were delivered to the electrode at 15-s intervals. For field recordings, recording pipettes (1–2 Mω) were filled with the bath solution and placed in the CA1 region. One or four tetanic stimuli (100 pulses at 100 Hz, 5-min intervals between trains) were delivered to induce LTP.

### Neuronal culture and transfection

Hippocampal and cortical neuron cultures were prepared from embryonic day (E) 18–19 rat embryos as previously described^48^. Rats were used because more neurons can be obtained from a rat than from a mouse brain due to its larger size. Neurons were seeded on poly-D-lysine- (30 mg ml^−1^) and laminin- (2 mg ml^−1^) coated coverslips or plates at a density of 750 cells mm^−2^ (for spine morphology analysis) or 2,100 cells mm^−2^ (for immunoblotting). Cultures were grown in Neurobasal medium supplemented with 2% B27, 2 mM glutamax. Hippocampal neurons were transfected with Lipofectamine 2000. All reagents for neuronal cultures were purchased from Invitrogen.

### Live imaging and induction of chemical LTP

Neurons grown on coverslips were transferred to the imaging chamber perfused with heated (31 °C) ACSF (pH 7.4, gassed with 95% O_2_/5% CO_2_, composed of in mM: 124 NaCl, 3 KCl, 26 NaHCO_3_, 1.25 NaH_2_PO_4_, 2.5 CaCl_2_, 1.3 MgSO_4_ and 10 D-glucose). Images of transfected neurons were acquired with an Olympus laser scanning confocal microscope ( × 63 objective, numerical aperture 1.35). After taking the first image, chemical LTP was induced by perfusion with ACSF containing 25 mM TEA, 5 mM calcium and without magnesium for 15 min. The RSK inhibitor BI-D1870 (200 nM), added to ACSF 5 min before TEA treatment, was present throughout the imaging period. The same neurons were imaged again at 10 and 90 min after TEA treatment.

### Image analysis

The z-stack confocal images were collapsed to make two-dimensional projections for image analysis. Fluorescence intensity and spines were measured using the MetaMorph software. Spines on secondary dendrites were traced and analysed by MetaMorph. Dendrites were traced manually and measured using MetaMorph. Spine number was counted manually, and 3–5 dendritic segments were analysed for each neuron.

### Quantitative RT–PCR

The mirVanaTM miRNA Isolation Kit (Ambion) was used to extract small RNAs from cell lysates in accordance with the manufacturer's manual. miRNAs were transcribed into cDNAs and amplified by PCR with the Taqman primers (miR-26a, ID: 405; miR-384-5p, ID: 2602; miR-124, ID: 1182; let-7a, ID: 377; endogenous control gene U6, ID:1973). Pre- and pri-miRNAs were transcribed into cDNAs and amplified by PCR (pre-miR-26a primer: 5′-CCGTGGCCTTGTTCAAGTAA-3′ and 5′-AACCAAGAATAGGCCCCTTG-3′; pri-miR-26a primer: 5′-CTTCCGTAGCCCCTTCTCTT-3′ and 5′-AACCAAGAATAGGCCCCTTG-3′; pre-miR-384-5p primer: 5′-TTCCTAGGCAATGTGTATAATGTTGG-3′ and 5′-TGAACAATTTCTAGGAATGACTTACC-3′; pri-miR-384-5p primer: 5′-ACGTAGCAGGCTGCAGAAAT-3′ and 5′-TGAACAATTTCTAGGAATGACTTACC-3′; U6 primer: 5′-GCTTCGGCAGCACATATACTAA-3′ and 5′-AAAATATGGAACGCTTCACGA-3′). The RNA was eluted in 50 μl nuclease-free water and assessed for quality using the Agilent 2100 bioanalyzer. The Applied Biosystems 7900 Fast Real-Time PCR System was used for qRT–PCR.

### *In situ* hybridization

ISH of miRNA was performed as described^20^. Briefly, hippocampal neurons were fixed with 4% formaldehyde (in PBS containing 4% sucrose). After permeabilization with 0.5% Triton X-100, neurons were hybridized with 5′ and 3′ digoxigenin-labelled, locked nucleic acid-modified oligonucleotide probes (Exiqon). Hybridized probes were detected by incubation with horseradish peroxidase-conjugated anti-digoxigenin antibody (Roche) followed by amplification with Cy5-conjugated tyramide (Perkin Elmer).

### Crosslinking and immunoprecipitation assay

Cultured cortical neurons (DIV7) were transduced with lentivirus-expressing Flag-tagged Ago2. At 10 days after transduction, neurons were crosslinked with ultraviolet irradiation (480,000 μJ c^−1^ m^−2^) and then lysed in RIPA buffer containing 0.1 U μl^−1^ RNase inhibitor followed by sonication. After centrifugation at 13,000 r.p.m. for 30 min at 4 °C, the supernatant was incubated with anti-Flag antibody-conjugated beads (Sigma-Aldrich) overnight with rotation at 4 °C. The beads were washed five times with immunoprecipitation buffer (25 mM HEPES (pH 7.5), 150 mM NaCl, 0.5 mM EDTA, 1 mM EGTA, 10% glycerol, 0.1% NP40, 1 mM NaF, 1 mM 2-glycerophosphate and 1 mM Na_3_VO_4_). Bound RNAs were extracted with phenol/chloroform, precipitated with ethanol and analysed by qRT–PCR.

### Statistical analysis

The electrophysiology, imaging and immunoblotting data were first tested for normality using Shapiro–Wilk test and equal variance using Levene's test. Data that were normally distributed with equal variance were analysed using one-way analysis of variance and then the Student's *t*-test for *post hoc* analysis. Data that did not pass the normality and equal variance test were analysed using the Kruskal–Wallis test and then the Mann–Whitney *U*-test for *post hoc* analysis. *P*<0.05 was considered significant.

## Author contributions

Q.-H.G., X.L., J.Z. and Z.L. designed the experiments. Q.-H.G. conducted electrophysiology, imaging, qRT–PCR and immunoblotting experiments and analysed the data of these experiments. X.L. was involved in the live imaging experiment. D.Y. conducted the miRNA-gene-GO enrichment analysis, and Y.Y. and Y.L. analysed the deep-sequencing data. Z.H. constructed the deep-sequencing libraries, performed the ISH and the crosslinking and immunoprecipitation (CLIP) experiment and analysed the ISH and the CLIP data. J.Z. conducted deep sequencing. Q.-H.G., J.Z. and Z.L. wrote the manuscript.

## Additional information

**Accession codes:** The miRNA deep-sequencing data were deposited in the NIH Sequence Read Archive database under the accession code SRP051826.

**How to cite this article:** Gu, Q.-H. *et al.* miR-26a and miR-384-5p are required for LTP maintenance and spine enlargement. *Nat. Commun.* 6:6789 doi: 10.1038/ncomms7789 (2015).

## Supplementary Material

Supplementary Information Supplementary Figures 1-11 and Supplementary Tables 1-11
