# Supplementary material for: miR-26a and miR-384-5p are required for LTP maintenance and spine enlargement
Source: Nat Commun. 2015 Apr 10;6:6789. doi: 10.1038/ncomms7789 (PMC4403380; doi:10.1038/ncomms7789)
Supplement: Supplementary Information — Supplementary Figures 1-11 and Supplementary Tables 1-11 [file ncomms7789-s1.pdf]

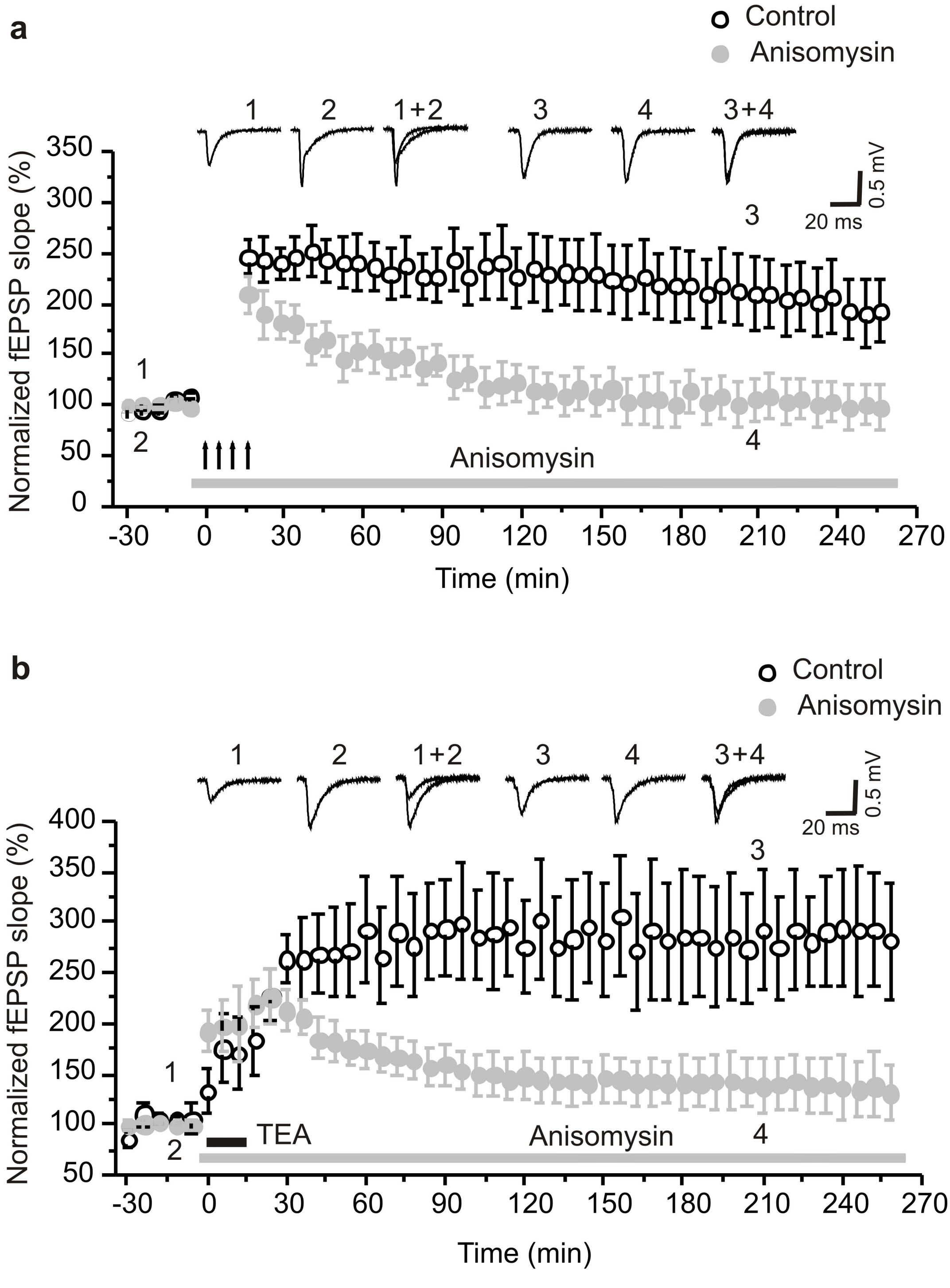

Supplementary Figure 1. Induction of translation-dependent LTP in the hippocampus. Hippocampal slices prepared from mice (2~3-week old) were stimulated at the Schaffer collateral pathway with 4 trains of 100 Hz pulses (a), or treated with TEA (b) for LTP induction. Anisomysin (20 μM) was added at 5 min before LTP induction. The fEPSP slope normalized to the baseline prior to stimulation was plotted as mean ± SEM. n = 5-6 slices for each group.

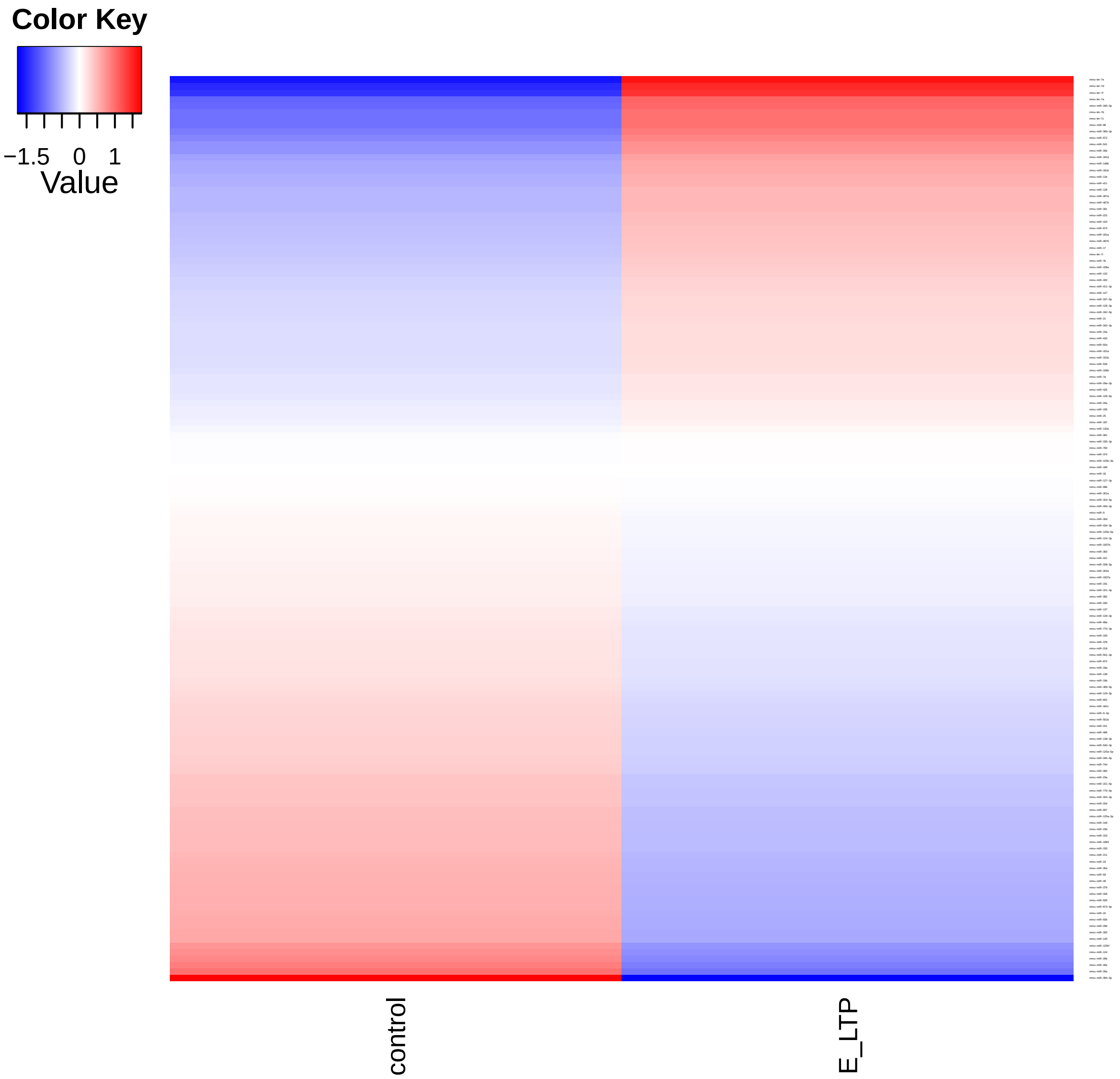

Supplementary Figure 2. Heat map of miRNAs analyzed for differential expression. Mean read counts of miRNAs following normalization are ordered by their p-values from small to large.

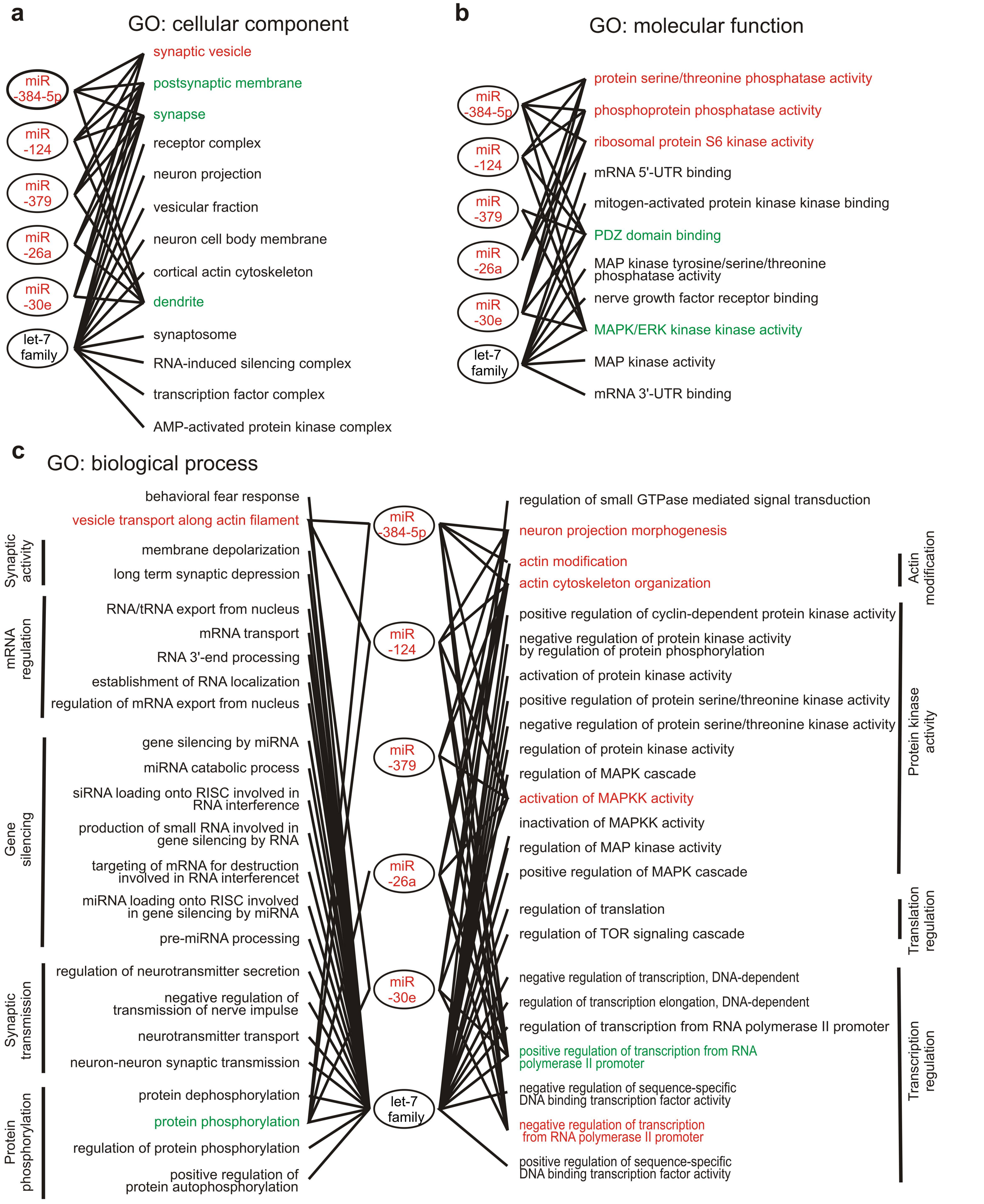

Supplementary Figure 3. Gene-ontology (GO) terms enriched by miRNAs differentially expressed in LTP. The enriched cellular component terms are shown in a, the enriched molecular function terms in b, the enriched biological process terms in c. GO terms enriched by down-regulated miRNAs are in red, by up-regulated miRNAs in black, and by both up- and down- regulated miRNAs in green.

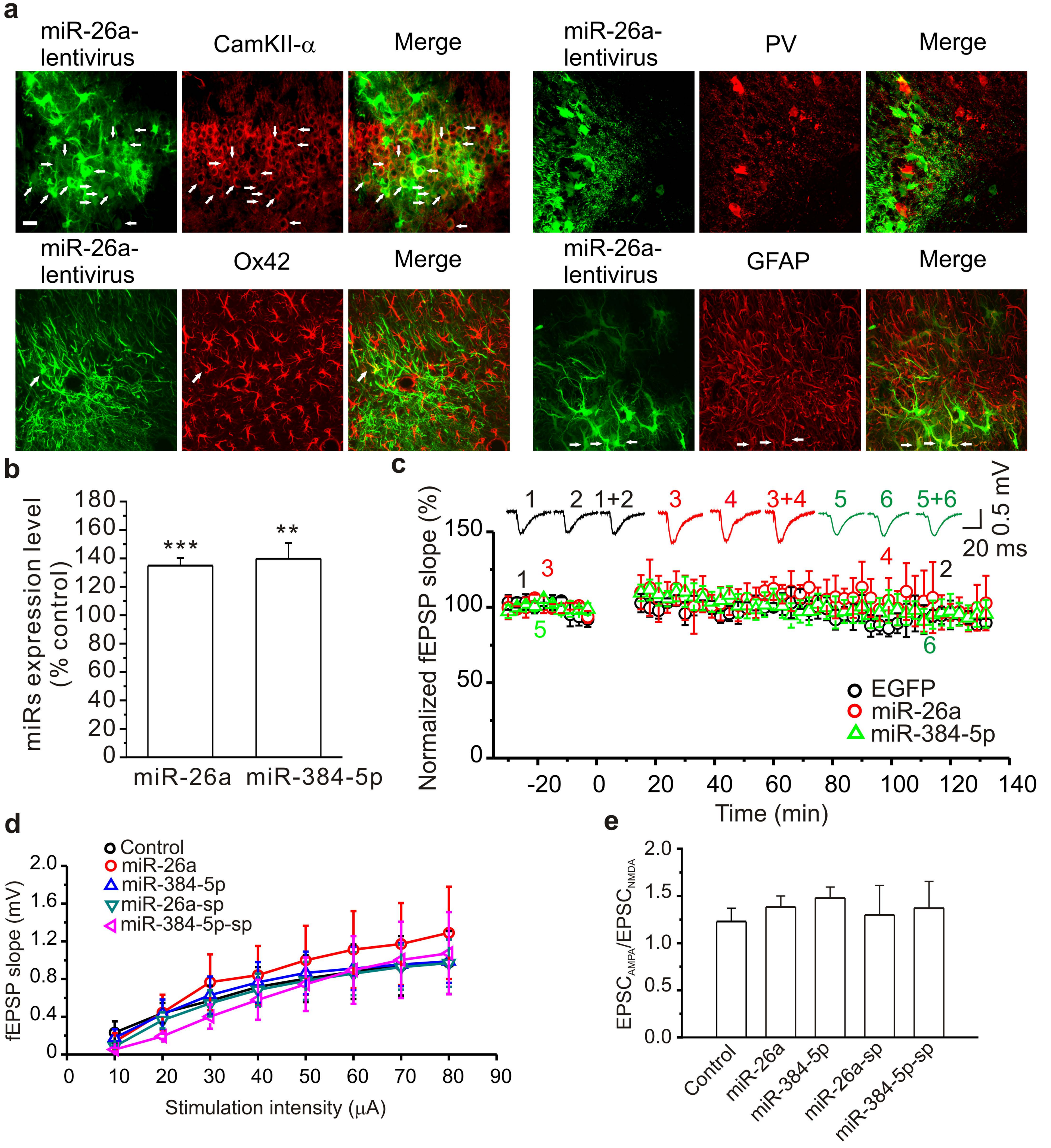

Supplementary Figure 4. miR-26a and miR-384-5p have no effect on the control pathway. (a) Representative images of the hippocampal CA1 region transduced with control virus or lentivirus expressing miR-26a or miR-384-5p, and stained with antibodies against Ca2+/calmodulin-dependent protein kinase II (CamkII), parvalbumin , glial fibrillary acidic protein (GFAP) or Ox42; arrows point to transduced cells colocalized with designated cell type markers; scale bar: 20 μm. (b) Cultured hippocampal slices were transduced with control virus (expressing EGFP) or lentivirus expressing miR-26a or miR-384-5p for qRT-PCR analysis of miR-26a and miR-384-5p. (c) Electrophysiological recording at both the CA3-CA1 (stimulated with high-frequency stimulation) and the subicular-CA1(control) pathway. Data in c show fEPSPs recorded in the control pathway (presented as mean ± SEM); n = 5-6 slices for each condition. (d) The input-output relationship in cultured hippocampal slices transduced with designated lentivirus (n = 5-10 slices in each group). (e) The ratio of AMPA and NMDA receptor-mediated currents in cultured hippocampal slices transduced with designated lentivirus (n = 6-10 neurons in each group).

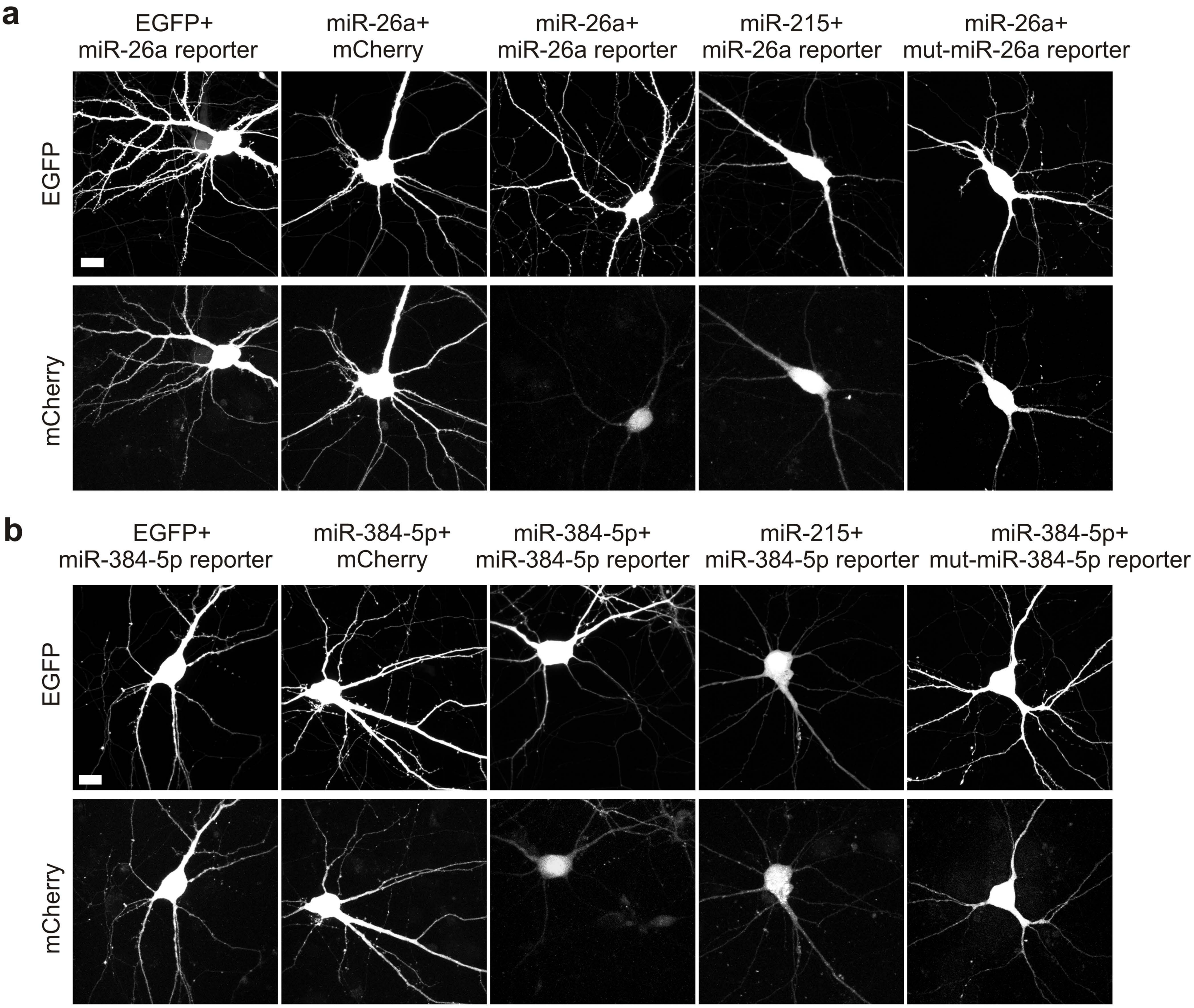

Supplementary Figure 5. RSK3 is a validated target of miR-26a and miR-384-5p.Cultured hippocampal neurons were transfected with designated constructs at DIV14 and fixed at DIV17. (a, b) Representative images of transfected neurons.

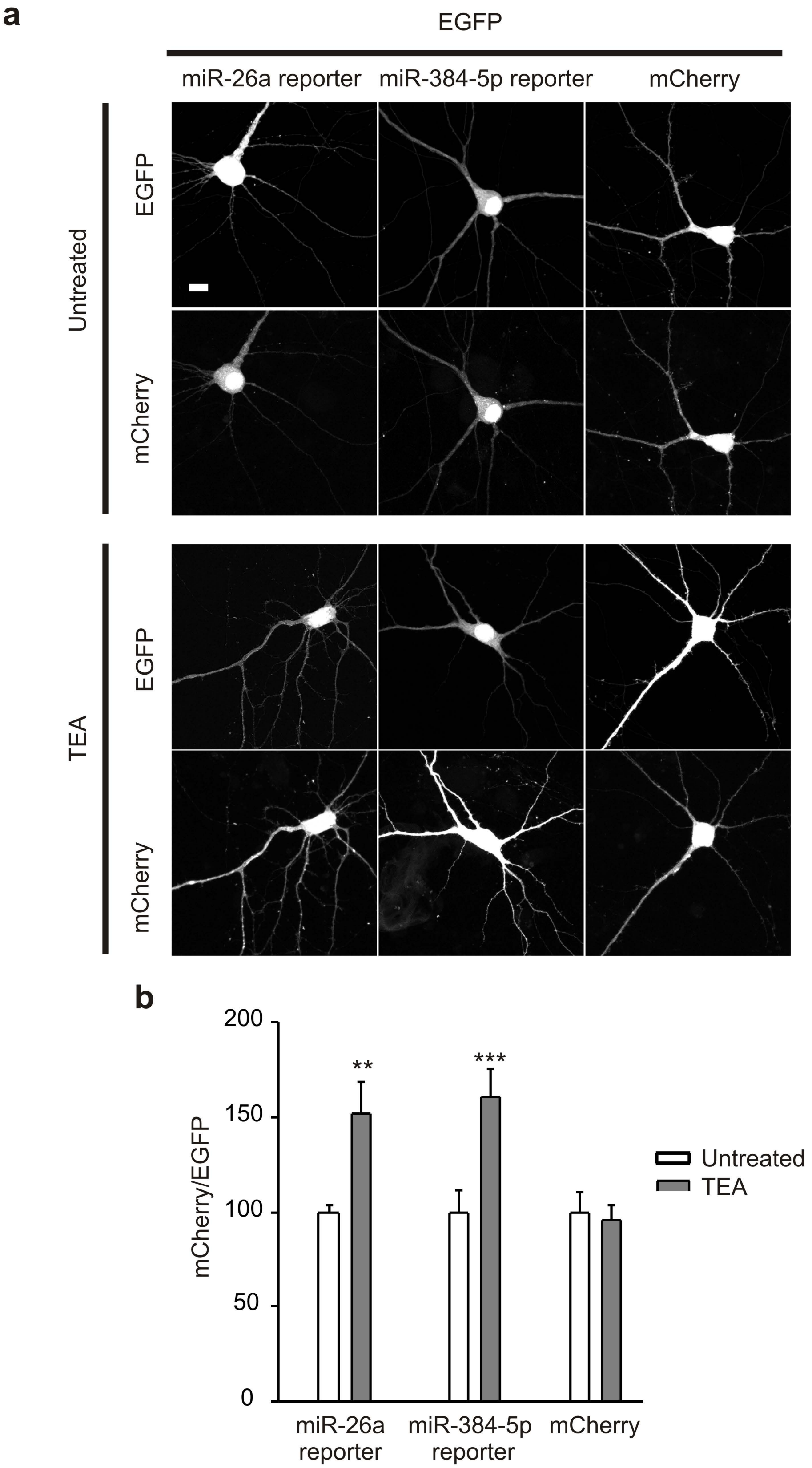

Supplementary Figure 6. TEA treatment alters the expression of reporters containing RSK3's miR-26a and miR-384-5p binding sites. Cultured hippocampal neurons (DIV 14) were co-transfected with the EGFP plasmid and the mCherry reporter constructs containing the miR-26a or miR-384-5p binding sites found in RSK3's 3'UTR. At 2-3 days after transfection, neurons were treated with TEA for 15 min, and fixed for image acquisition, neurons were treated with TEA for 15 min, and fixed for image acquisition at 90 min after treatment. (a) Representative images of transfected neurons. (b) Quantification of a. Scale bar, 20 μm. n = 20-40 neurons for each condition. Data are presented as mean ± SEM. Mann-Whitney U test is used for statistical analysis. ** $p<0.01$, *** $p<0.001$.

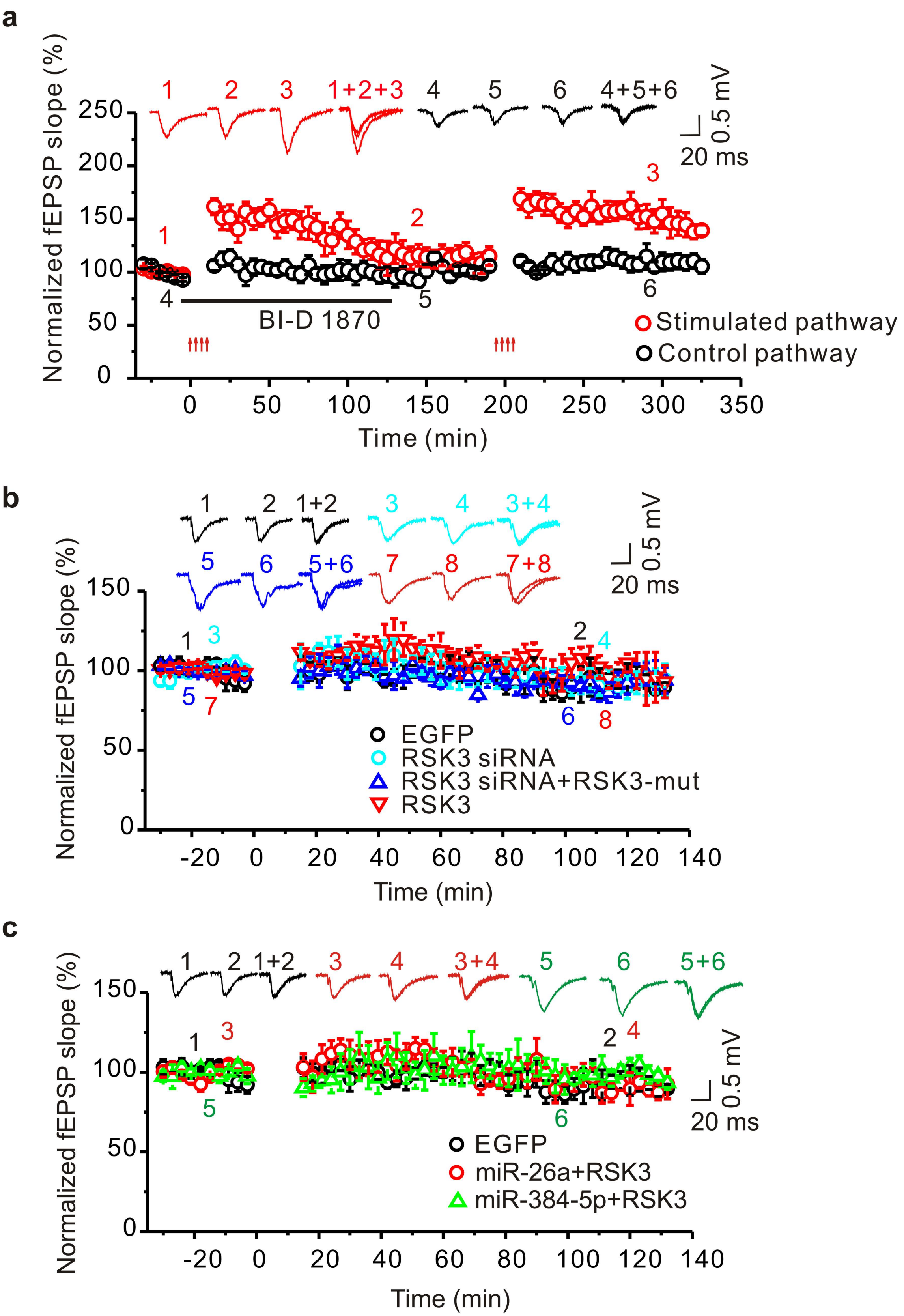

Supplementary Figure 7. The blockade of LTP maintenance by BI-D1870 is reversible. (a) Cultured hippocampal slices were treated with the RSK inhibitor BI-D1870 (200 nM) and stimulated with 4 tetanizations to induce LTP. At 2 hr after LTP induction, BI-D1870 was washed out, and slices were stimulated with tetanic stimulation again. (b, c) Cultured hippocampal slices were transduced with designated lentivirus and recorded at the CA1 region for responses evoked by stimulating the CA3-CA1 (for LTP induction) and the subicular-CA1 pathway (control pathway); data in b and c show the fEPSP (recorded from the control pathway) slope normalized to the baseline prior to stimulation (plotted as mean ± SEM). n = 5 slices for each condition.

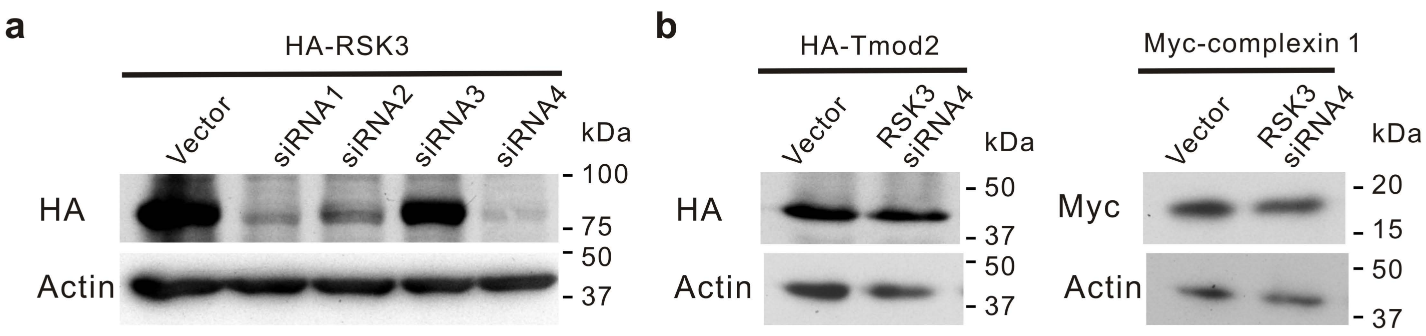

Supplementary Figure 8. The efficacy and specificity of RSK3 siRNA. Cos-7 cells were transfected with the RSK3 siRNA constructs along with HA tagged RSK3, TMOD2 or myc-tagged complexin 1. Cells were harvested at 2 days after transfection for immunoblotting against HA or myc.

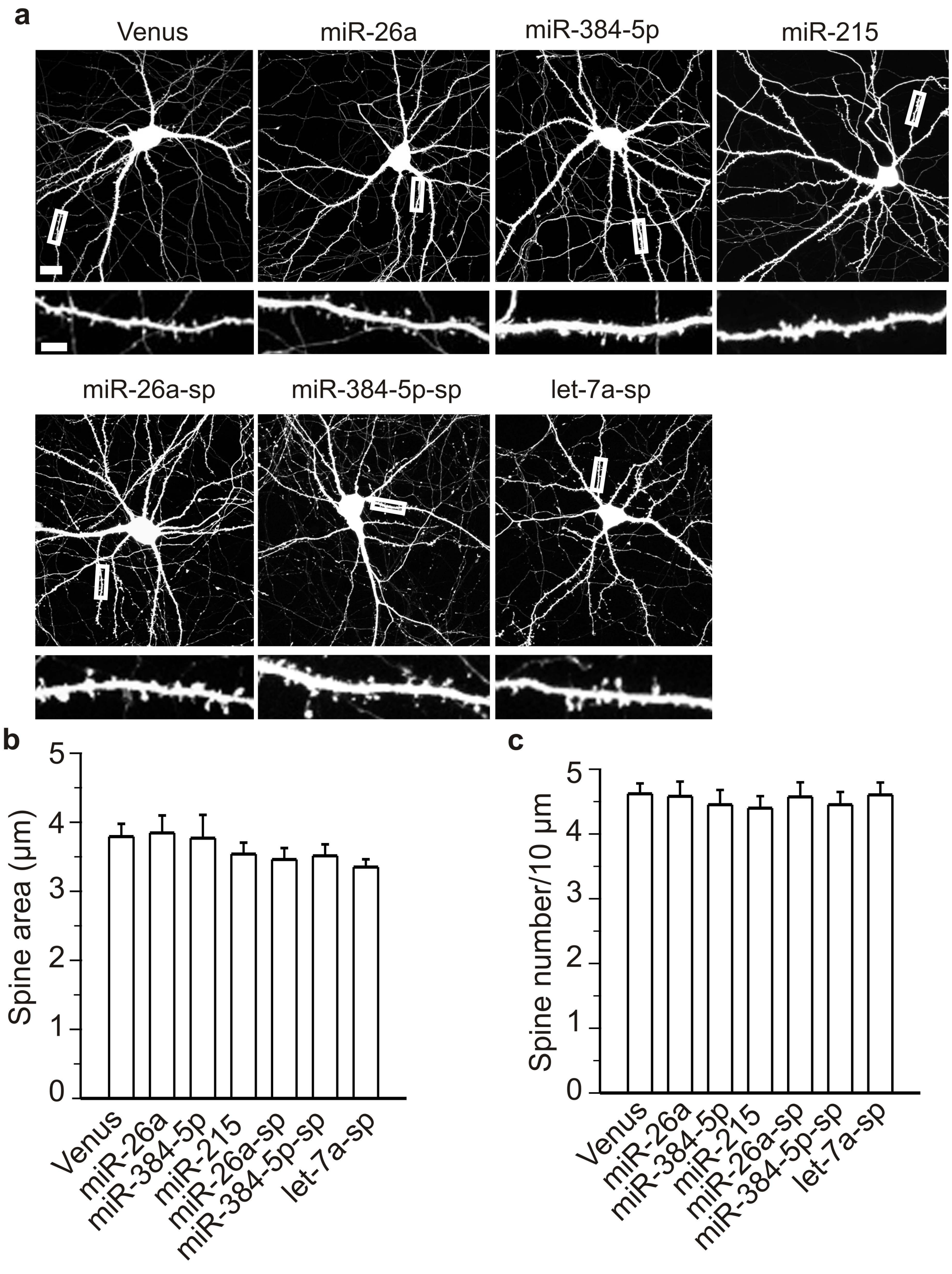

Supplementary Figure 9. miR-26a, miR-384-5p and let-7a do not affect the basal level of spine size and density. Cultured hippocampal neurons (DIV14) were transfected with the Venus constructs along with constructs expressing designated miRNAs or miRNA sponges, and imaged at 2-3 days after transfection. (a) Represented images. (b-c) Quantification of a. Data are presented as mean ± SEM. Scale bar, 20 μm for low- and 5 μm for high-magnification images.

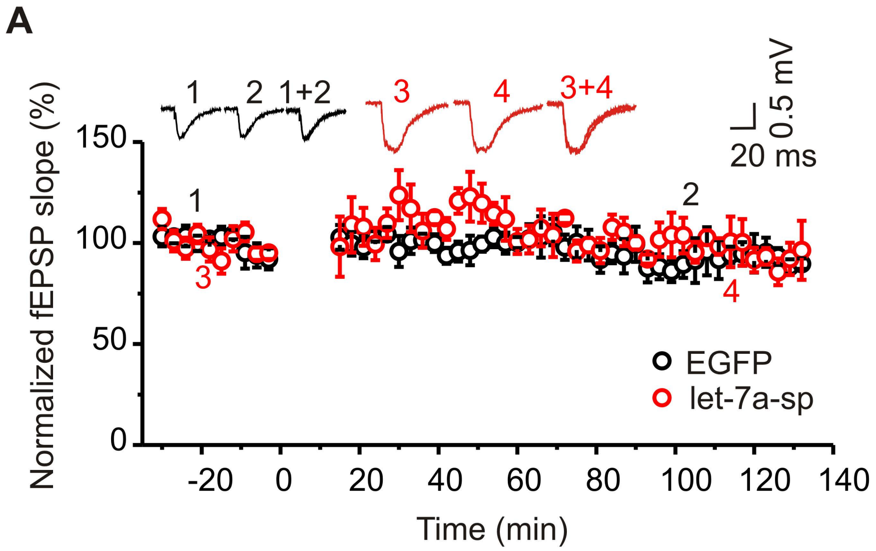

Supplementary Figure 10. Let-7a knockdown has no effect on the control pathway. Cultured hippocampal slices were transduced with the EGFP virus or lentivirus expressing let-7a sponge, and recorded at both the CA3-CA1 (stimulated with high-frequency stimulation) and the subicular-CA1 (control) pathway. Data show fEPSPs recorded in the control pathway (presented as mean ± SEM). n = 5-6 slices for each condition.

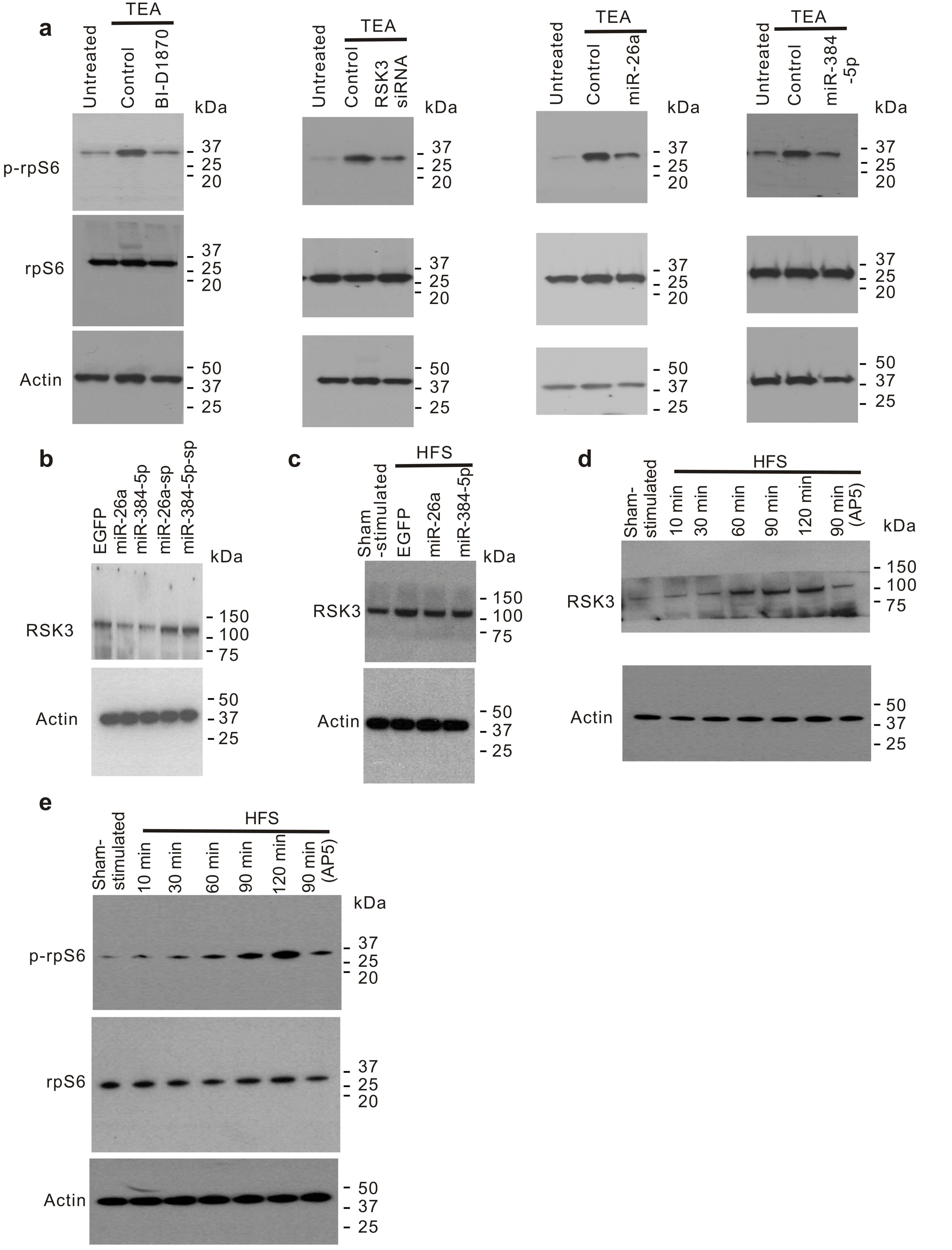

Supplementary Figure 11. Full-blot images of immunoblots. (a) Full-blot images of the cropped representative blots for p-rpS6, rpS6 and actin in Figure 4. (b, c) Full-blot images of the cropped representative blots for RSK and actin in Figure 3c,e.(d) Full-blot images of the cropped representative blots for RSK and actin in Figure 7e. (e) Full-blot images of the cropped representative blots for p-rpS6, rpS6 and actin in Figure 7g.

Supplementary Table 1. Summary of sequencing results.

Sample ID	Read count	Mapped read	% Mapped
Control-1	591741	434847	73
Control-2	225731	173992	77
Control-3	531646	363928	68.5
LTP-1	76633	57790	75
LTP-2	673645	446227	66.2
LTP-3	728130	464387	63.8

Supplementary Table 2. Genes enriched by miRNAs differentially expressed in LTP.

**Down-regulated miRNAs**	
miRNA ID	*Enriched target gene*
mmu-miR-124	Adamts9, Dmd, Ednrb, Fxr1, Gfpt2, Gnai2, Kif3a, Myo5a, P4ha2, Sypl, Rarg, Kctd8, Vat1l, Usp2, B4galt6, Sec61a2, Polr3g, Zfp706, Elovl5, Kctd5, 3110043O21Rik, Faf2, 2310046O06Rik, Bcl2l13, Sall4, Oaf, Osbpl11, Epha2, Gna13, 2700050L05Rik, Ccdc28a, Cldnd1, Slc9a2, Frmd4b, Slc2a13, Wipf3, Dapk1, Dram1, Sema6d, Reep3, Atad2b, Brwd3, Celsr3, Gpt2, Bcl6, Cbx2, Pip4k2a, Rwdd4a, Surf4, Usp49, Cadps, Zbtb11, Lrrc58, Dll4, Rffl, Ube2v2, Gria2, Mat2a, Map3k1, Zfp275, Stk39, Dlg5, Larp1, Ranbp10, Kif21b, Fbxo42, Slco5a1, Baz2b, Tet1, Nap1l5, Mtdh, Eaf1, Ovol2, Acp2, Calu, Eya2, P4ha1, Pnn, Snai2, Parp16, Ube2i, Sdad1, Lrrc3, Cpne5, Lrrfip2, Pgm2, Pus10, Usp48, Kalrn, Atl3, Pcdh17, Zfp608, Sh3rf1, Lnp, Zdhhc20, Fa2h, Tmed10, Abcc4, Fam174b, Fam150b, Slc15a4, Usp30, Hepacam2, Numa1, Gcom1, 10-Sep, Qsox1, Lmf2, Fbxo38, Rapgef1, Cstf2, Rnpepl1, Ccdc86, Pttg1ip, Sft2d2, Aif1l, Cyb5, Pecr, Abca2, Acadvl, Ache, Aspa, Adcy9, Acan, Ak2, Ampd3, Anxa4, Anxa5, Prdx6, Ap1m2, Arpc1b, Rab27a, Atp1a1, Barx2, Bckdha, Bmp6, Cacnb2, Capn1, Casq2, Cav1, Col12a1, Cpt1a, Cycs, Slc25a1, Dlx5, Dnajc1, Dsg2, Efnb1, Rhbdf1, Epha3, Eya1, Ube4a, Cacng5, Flot1, Fpgs, Fzd4, Xrcc6, Galc, Gas1, Gas2, Gdap2, Gnai1, Gnal, Gng10, Gpam, Lpcat3, Aes, Hadh, Nr4a1, Hoxb1, Itga7, Itgae, Stt3a, Itpr3, Kcnk2, Kif2a, Hivep3, Lhx2, Lipe, BC017612, Tmed1, Galnt10, Mdk, Cox4nb, Nthl1, Nxn, Pcdh8, Peg3, Pemt, Phex, Pip5k1a, Lcp1, Ppfibp2, Ppl, Casc3, Luc7l2, Wdr81, Ptprz1, BC046404, Rab3d, Rgs9, Rras, Rxra, Ryr1, Scd1, Scd2, Scn7a, Slc22a5, Slit1, Snta1, Sos2, Sox8, Sphk1, Pde2a, Yeats2, Rsrc2, Suclg2, Clic6, Sdc4, AF529169, Thsd7b, Mkx, Cspp1, Aldh4a1, Lman2l, Arrdc1, Aldh1l2, Pik3ip1, Rufy1, Pqlc3, L2hgdh, Tjp2, Zfp503, Spata13, Top3a, Tpst2, Ttc3, Eny2, Zbed4, Pim3, Lemd2, Enpp4, Zfp239, Arhgef4, Ctdsp1, Gsn, Smox, Trib3, Gdap1l1, Cbln4, Galnt12, Zfp189, E130308A19Rik, Usp1, Zmpste24, Serinc2, B930041F14Rik, 9330182L06Rik, Galnt9, Tmem150a, Shisa7, Zscan22, Fbxo27, Nipa1, Thumpd1, Tpcn2, Tmco3, Ftsjd1, Sc5d, Slc17a5, Gk5, Lrfn5, 6430527G18Rik, Mtr, Fxr2, Tmem20, Tmem8b, Dmrta1, Tsku, Ssh3, Chodl, Ttc26, Plod3, Slc27a1, Homer2, Ror2, Zscan20, Lpcat2, Yme1l1, Marveld1, Nynrin, Eif3b, Nsun2, Apln, Tor3a, Casc4, Tmem117, Sdk1, Ctxn1, Slc9a9, Slc2a12, 5930434B04Rik, Xkr8, Gpr114, Poc1b, Itprip, Zfp213, Slc25a13, Agpat5, Dhrs1, Vamp4, Htatip2, Cd164, 9-Sep, Zkscan4, Sfrp5, Rcan1, Brp44l, Slc1a4, Hebp2, Rhog, Srpk3, Nme4, Aldh9a1, Apba3, Slc29a1, Sdf2l1, Suv39h2, Vps35, Twsg1, Tmem111, 1110031I02Rik, Tpd52l2, Farsa, Lix1, Nhlrc2, Pcyox1, Prpf38b, Cand2, 2410127L17Rik, Fundc2, Frmd8, Snap29, Akt1s1, Dctn4, Fam174a, Ccdc130, Dgat2, Sugt1, Trabd, Dcakd, AI846148, Lmbrd1, Steap3, Ppm1f, Acss1, 1110059M19Rik, 2310044H10Rik, Ttl, Zfp219, 2610018G03Rik, Tspan15, Inf2, Arhgap17, Hars2, Ccdc93, Osbpl7, Myo1e, Ankle2, Rnf135, Snx6, Kcnk10, Plekhf1, Pan3, Zkscan3, Spock3, 1110012D08Rik, Slc35f5, Paqr8, Chic2, Dhcr24, Mmd2, Rasd2, Fgfr1op, Ascc2, Pcdh1, Rilpl1, Morc4, Gstk1, Glt8d1, Clip3, Tbc1d9b, Fam81a, Lass2, Agl, Myh10, Zfp687, Mvp, Tsc22d4, Sp2, Gas2l1, Ern1, Trim39, Parp9, Nckipsd, Tcfap4, Ctns, Slc12a9, Tbx19, Narg2, C1galt1, Hadha, Efhd1, Rdh10, Stard7
mmu-miR-26a	Mex3b, Pawr, Adam9, Ctnnd2, Twf1, Erlin1, Fkbp3, Jakmip2, Mfsd6, Oaf, Osbpl11, Epha2, Gna13, 2700050L05Rik, Ccdc28a, Cldnd1, Slc9a2, Frmd4b, Slc2a13, Dapk1, Dram1, Sema6d, Reep3, Atad2b, Brwd3, Epb4.1l3, Mab21l1, Smad1, Nid1, Ocln, Ppp3cb, Ptpn13, Rps6ka2, Tcp11l2, Mkrn3, Cdh20, Abi2, Pdcd10, Arl6ip6, Brap, Ube2ql1, Mat2a, Zfp275, Stk39, Dlg5, Larp1, Ranbp10, Kif21b, Fbxo42, Slco5a1, Baz2b, Tet1, Nap1l5, Mtdh, Eaf1, Usp48, Kalrn, Atl3, Zfp608, Sh3rf1, Lnp, Zdhhc20, Fa2h, Tmed10, Abcc4
mmu-miR-30e	Adamts9, Mex3b, Pawr, Adam9, Dmd, Ednrb, Fxr1, Gfpt2, Gnai2, Kif3a, Myo5a, Ctnnd2, P4ha2, Sypl, Twf1, Rarg, Sema3a, Erlin1, Kctd8, Vat1l, Fkbp3, Usp2, B4galt6, Sec61a2, Polr3g, Fbxo32, Zfp706, Elovl5, Kctd5, 3110043O21Rik, Jakmip2, Faf2, 2310046O06Rik, Bcl2l13, Mfsd6, Sall4, Wipf3, Sema6d, Reep3, Atad2b, Brwd3, Celsr3, Gpt2, Bcl6, Cbx2, Epb4.1l3, Fst, Mab21l1, Smad1, Nid1, Ocln, Pip4k2a, Ppp3cb, Rwdd4a, Ptpn13, Rps6ka2, Surf4, Tcp11l2, Usp49, Mkrn3, Cdh20, Cadps, Zbtb11, Lrrc58, Abi2, Dll4, Pdcd10, Arl6ip6, Edem3, Rffl, Ube2v2, Brap, Ube2ql1, Gria2, Mat2a, Map3k1, Zfp275, Stk39, Dlg5, Larp1, Ranbp10, Kif21b, Fbxo42, Slco5a1, Baz2b, Tet1, Nap1l5, Mtdh, Eaf1, Ovol2, Acp2, Calu, Eya2, P4ha1, Pnn, Snai2, Parp16, Ube2i, Sdad1, Lrrc3, Cpne5, Lrrfip2, Pgm2, Pus10, Usp48, Kalrn, Atl3, Pcdh17, Zfp608, Sh3rf1, Lnp, Zdhhc20, Fa2h, Tmed10, Abcc4
mmu-miR-379	Sema3a, Fbxo32, Fst, Edem3, Gria2, Map3k1, Pcdh17, Abcc4, Edn1, Htr1d, Pde2a, Ube2e3, Homer2, 5930434B04Rik

mmu-miR-384-5p	Adamts9, Mex3b, Pawr, Adam9, Dmd, Ednrb, Fxr1, Gfpt2, Gnai2, Kif3a, Myo5a, Ctnnd2, P4ha2, Sypl, Twf1, Rarg, Sema3a, Erlin1, Kctd8, Vat1l, Fkbp3, Usp2, B4galt6, Sec61a2, Polr3g, Fbxo32, Zfp706, Elovl5, Kctd5, 3110043O21Rik, Jakmip2, Faf2, 2310046O06Rik, Bcl2l13, Mfsd6, Sall4, Sema6d, Reep3, Atad2b, Brwd3, Celsr3, Gpt2, Bcl6, Cbx2, Epb4.1l3, Fst, Mab21l1, Smad1, Nid1, Ocln, Pip4k2a, Ppp3cb, Rwdd4a, Ptpn13, Rps6ka2, Surf4, Tcp11l2, Usp49, Mkrn3, Cdh20, Cadps, Zbtb11, Lrrc58, Abi2, Dll4, Pdcd10, Arl6ip6, Edem3, Rffl, Ube2v2, Brap, Ube2ql1, Gria2, Mat2a, Map3k1, Zfp275, Stk39, Dlg5, Larp1, Ranbp10, Kif21b, Fbxo42, Slco5a1, Baz2b, Tet1, Nap1l5, Mtdh, Eaf1, Ovol2, Acp2, Calu, Eya2, P4ha1, Pnn, Snai2, Parp16, Ube2i, Sdad1, Lrrc3, Cpne5, Lrrfip2, Pgm2, Pus10, Usp48, Kalrn, Atl3, Pcdh17, Zfp608, Sh3rf1, Lnp, Zdhhc20, Fa2h, Tmed10, Abcc4
**Up-regulated miRNAs**	
miRNA ID	*Enriched target gene*

mmu-miR-7a	Nhsl2, Nfxl1, Zfp282, Rpusd3, B630005N14Rik, A130022J15Rik, Fbxl14, Spred3, Agpat6, Dcun1d2, Cd276, Cdh22, Sbk1, Aldh6a1, Tecpr2, Rab15, Iars, Rnf44, Dusp22, AW549877, Dhx57, 2010002N04Rik, Gpr137, Unc5a, Lgr4, Chd4, Olr1, Prkaa2, Prkab2, Galnt2, B3gnt1, Uhrf2, Chd9, Rnf20, Txlna, Braf, Hdlbp, Lmx1a, Slc8a2, Hmga1-rs1, Egln2, Abcg1, Foxp2, Acta1, Acvr1b, Acvr2a, Adrb2, Adrb3, Ankfy1, Ap1s1, Speg, Aqp4, Bach1, Bcap29, Bcat1, Bcl2l1, Prdm1, Bsn, Cacna1d, Calm1, Cap1, Cask, Casp3, Cbx2, Cbx5, Ccnd1, Ccnd2, Cd86, Cdc25a, Chrd, Socs1, Tpp1, Cmah, Ccr7, Coil, Col14a1, Col15a1, Col3a1, Col4a1, Col4a2, Col4a5, Col5a2, Col1a2, Cpd, Crk, Cry2, Cyp46a1, Ddn, Dmd, Dmp1, Dnajc1, Arid3a, Dyrk1a, E2f5, Eda, Edn1, Ddx19a, Eif4g2, Epha3, Epha4, Esr2, Smarcad1, Igf2bp3, Ezh2, Fasl, Fgf4, Fgf5, Frk, Gabpa, Gabra6, Galnt1, Gas7, Mtpn, Nr6a1, Gdap2, Ghr, Gnal, Gng5, Grik2, Grin2b, Gtf2i, Hand1, Has2, Hic1, Hk2, Hmga1, Hmga2, Hoxd1, Htr4, Igf1r, Il10, Il13, Il6, Lrig1, Insr, Kcnc3, Khsrp, Kif21b, Klkb1, Kpna1, Lalba, Limk2, Sh2b3, Lor, Peg10, Slc24a6, Ppargc1b, Grid2ip, Mxd1, Masp1, Mdfi, Mllt10, Ndst2, Meis2, Meis3, Grpel2, Myo1f, Nefm, Ngf, Nid2, Nlk, Mycn, Nras, Nrtn, Ntn1, Osmr, P2rx1, P4ha2, Pldn, Pak1, Pappa, Pbx1, Pbx2, Pbx3, Pcx, Pdgfb, Piga, Pld3, Prrx1, Ppara, Ppargc1a, Mobkl3, Mapk11, Prlr, Socs7, Edem1, Dusp1, Pla2g15, Ptprd, Ptpru, Igdcc3, Rasgrp1, Tet3, Rb1, Rbpj, Rcn1, Rdx, Dpf2, Rgs16, Rnf7, Rorc, Sub1, Rrm2, Scd2, Scn5a, Scn8a, Msr1, Sema4c, Sema4f, Ostf1, St6gal1, St8sia1, Clpb, Skil, Slc31a1, Smarcc1, Snap23, Snn, Sox13, Sall3, Strbp, Dtx4, Wdr37, Gramd1c, Srebf2, Dot1l, Slk, Prrg3, Dock3, Cpeb3, Zc3hav1l, Stx3, Snx30, Zfp710, Igsf1, Lcorl, Tmc7, Syt1, Syt2, Tmem194, Trim41, Fam123c, Trib1, Plekhh1, Pde12, Zfyve26, Dcaf15, Lcor, Pqlc2, Mfsd4, Plekhg6, Tarbp2, Tbx5, Zfp354a, Nipal4, Tet2, Tcf7l1, Etnk2, Hip1, C330019G07Rik, Lrrc20, Cdc34, Eea1, 9-Mar, Pik3ip1, Fnip1, Tead3, Tead4, Wnt9a, Taok1, Hlf, Trib2, Pacs2, Tgfbr1, Tgfbr3, Thbs1, Thrsp, Tia1, Gcnt4, Marveld2, Timm17b, Slc4a7, Sec24c, D14Abb1e, Pcdh20, Tnfaip3, Tnfrsf1b, Usp12, Ugcg, Utrn, Farp1, Zc3h3, 5031439G07Rik, Gxylt1, Wnt1, Fam43a, Wnt9b, Impg2, Xk, Rab40c, Uhrf1bp1, Btbd9, Abcc10, Dlgap1, Map4k3, Thoc1, Ammecr1l, Apbb3, Cep120, Rnf165, Lrfn4, Trpm6, Fam178a, Yod1, Ahctf1, Zfp2, Vash2, B3gnt7, Ikzf2, Slc30a1, Slc35c1, Frmd5, Btbd3, Dlgap4, Ppp1r16b, Npepl1, Zbtb10, Ythdf3, Syt11, Pogz, Slc25a24, B230312A22Rik, Zbtb5, Ikbkap, Acer2, Slc5a9, Zfp362, Fras1, Frmd4b, Slc6a1, Dcun1d3, Psd3, Ddx19b, Tmem231, Adamts15, Zbtb16, Lingo1, Rab8b, Prtg, Ddx26b, Pcyt1b, Nxt2, Adamts14, Trhde, Ankrd52, Pla2g3, Ankrd43, Usp32, Adamts5, Akap6, Fam160b2, Adamts12, Rspo2, Mgll, Nek3, Smcr7l, Klhl6, Liph, Lmln, Gpr156, Arid1b, Scn11a, Ccnj, Slco5a1, Tor1aip2, Scyl3, Crb2, Tspan18, Slc7a14, Frem2, Zfp697, Gdf6, Zfp462, Klhl9, Gpatch3, E2f2, Napepld, Tmprss11f, Sbno1, Stard13, Styk1, Arhgap20, Tnfaip8l3, C230081A13Rik, Klhl31, Klf8, Trappc1, Onecut3, Srgap3, Gga3, Esrrb, Spata2, Map3k1, Mapk8, Sema4g, Homer2, Rab11fip4, Greb1, Slc38a9, 9930012K11Rik, B3galt1, Arhgap28, Dagla, Mgat4a, Cep110, Map4k4, Acvr1c, Znf512b, Trpc7, Lrig2, Vsnl1, Gpr157, Nat8l, AU019823, Rab6b, Mbtps2, Zfp275, Rpusd2, X99384, Dnajb9, Abcc5, Rasl10b, Nynrin, Klhl23, Efhd2, Ubfd1, D11Wsu99e, Limd1, Ulk2, Adamts8, Fbxl12, Fndc3a, Pld5, Dusp4, Hif1an, Igf2bp2, Apba1, Ercc6, Uggt1, Nceh1, Zbtb39, Zmat4, Lrrc58, Senp5, Iqcb1, Lrig3, Trank1, Ubn2, Phf8, Zbtb26, Pptc7, Slc26a9, Rfx6, Cdv3, Frs2, Pitpnm3, Gfod1, Zfp516, 8430427H17Rik, Fnip2, AI464131, Usp24, Acot11, Slc5a6, Sdk1, D630045J12Rik, Trim67, Thoc2, Kcnk18, Slc2a12, Col27a1, Serf2, Lin28b, Obscn, Begain, Mll2, Greb1l, 5930434B04Rik, Xkr8, Cep135, Pdp2, Scn4b, Fam84b, Taf9b, Gnptab, Nlrc5, Arhgef15, E2f6, Ero1l, Tmprss2, Dlc1, Mapk6, Dkk3, Tmod2, Rragd, Reep1, Atxn1l, Zfp644, Rufy3, Tmx4, D1Bwg0212e, Hif3a, Nap1l1, Cpsf4, Nme6, Slc4a4, Plagl2, Plxnc1, Arid3c, Rbm38, Tspan5, Styx, Mycbp, Abcb9, Arid3b, 1500003O03Rik, Syncrip, Ikbke, Ankrd49, Rbms2, Nme4, Habp4, Crtap, Igdcc4, Vav3, Tob2, Psors1c2, Golga7, Cd200r1, Bin3, Hic2, Fam184b, Arpp19, Fign, Kctd21, Trim71, Ralb, Zfp280b, Suv39h2, Tsc1, Cldn12, Tmem167, Arrdc4, Pcp4l1, Nova1, Tnik, Gdpd1, Dzip1, Abi3, Atg4b, Tmem41a, Dcbld1, Spryd4, Slc16a9, 1200014J11Rik, Riok3, Bzw1, Bzw2, Prpf38b, Golt1b, Edem3, Nkap, Polr3d, Gatm, Lipt2, Cap2, Brd3, Ergic1, 1810074P20Rik, Ttll4, Stx17, Fbxo32, Gpatch2, Plxnd1, Limd2, Ubxn4, Gipc1, Zcchc3, Ccny, Trabd, Aen, Cgnl1, Fam135a, Leprotl1, AI846148, 0610030E20Rik, Adipor2, Nsmce2, Ppm1f, Letmd1, Tab2, Zfp740, Ddi2, Vps26b, 2200002K05Rik, Snx5, Tmem110, Pcgf3, Fam83g, Pxdn, Ints2, Slc35d2, Naa30, Dusp16, Tspan2, Prss22, Pgm2l1, Stox2, Zfp689, Golga7b, Col24a1, Ing3, Slc16a14, Cpa4, Slc25a18, Fbxo30, Ppapdc1b, Nkiras2, Ppp2r2a, Fndc3b, Cyb561d1, Tsc22d2, Cnot2, Lsm11, 2410042D21Rik, Ppme1, 2810030E01Rik, B3gat3, Kctd17, Slc22a23, Tmed5, Tbkbp1, Wasl, Xpot, Fam118a, 1700052N19Rik, Vgll3, Ppp2r1b, 1110012D08Rik, Slc25a27, Nphp3, Armc8, Sec14l1, Crct1, Stk40, Dtx2, 1700017B05Rik, Gale, 4931428F04Rik, Lonrf3, Ppapdc2, Zswim5, Snx16, Usp38, Tmem65, Usp47, Rab11fip2, Dpp3, Etnk1, Arl5a, Gns, Slc10a6, Klhl24, Senp2, 6230409E13Rik, Stard3nl, Tgds, Clasp2, Slc10a7, Eif2c4, Atg16l1, Bcl7a, Rnf170, Lbh, Hook1, Klhdc8b, Rictor, Dlst, Chsy3, Spnb4, Tusc2, Med8, Mesdc1, Zfp202, Arl4d, Ndst3, Ctns, Lin28a, Elovl4, Slc12a9, Tmem2, Kremen1, Sec16b, Entpd7, Gpr45, Brwd1, Cecr6, Trim33, Cnnm4, Qars, Lbr, Rdh10, Clp1, Cercam, Sall4, Kcnc4

mmu-miR-7b	Nhsl2, Nfxl1, Zfp282, Rpusd3, B630005N14Rik, A130022J15Rik, Fbxl14, Spred3, Agpat6, Dcun1d2, Cd276, Cdh22, Sbk1, Aldh6a1, Tecpr2, Rab15, Iars, Rnf44, Dusp22, AW549877, Dhx57, 2010002N04Rik, Gpr137, Unc5a, Lgr4, Chd4, Olr1, Prkaa2, Prkab2, Galnt2, B3gnt1, Uhrf2, Chd9, Rnf20, Txlna, Braf, Hdlbp, Lmx1a, Slc8a2, Hmga1-rs1, Egln2, Abcg1, Foxp2, Acta1, Acvr1b, Acvr2a, Adrb2, Adrb3, Ankfy1, Ap1s1, Speg, Aqp4, Bach1, Bcap29, Bcat1, Bcl2l1, Prdm1, Bsn, Cacna1d, Calm1, Cap1, Cask, Casp3, Cbx2, Cbx5, Ccnd1, Ccnd2, Cd86, Cdc25a, Chrd, Socs1, Tpp1, Cmah, Ccr7, Coil, Col14a1, Col15a1, Col3a1, Col4a1, Col4a2, Col4a5, Col5a2, Col1a2, Cpd, Crk, Cry2, Cyp46a1, Ddn, Dmd, Dmp1, Dnajc1, Arid3a, Dyrk1a, E2f5, Eda, Edn1, Ddx19a, Eif4g2, Epha3, Epha4, Esr2, Smarcad1, Igf2bp3, Ezh2, Fasl, Fgf4, Fgf5, Frk, Gabpa, Gabra6, Galnt1, Gas7, Mtpn, Nr6a1, Gdap2, Ghr, Gnal, Gng5, Grik2, Grin2b, Gtf2i, Hand1, Has2, Hic1, Hk2, Hmga1, Hmga2, Hoxd1, Htr4, Igf1r, Il10, Il13, Il6, Lrig1, Insr, Kcnc3, Khsrp, Kif21b, Klkb1, Kpna1, Lalba, Limk2, Sh2b3, Lor, Peg10, Slc24a6, Ppargc1b, Grid2ip, Mxd1, Masp1, Mdfi, Mllt10, Mmp11, Ndst2, Meis2, Meis3, Grpel2, Myo1f, Nefm, Ngf, Nid2, Nlk, Mycn, Nras, Nrtn, Ntn1, Osmr, P2rx1, P4ha2, Pldn, Pak1, Pappa, Pbx1, Pbx2, Pbx3, Pcx, Pdgfb, Piga, Pld3, Prrx1, Ppara, Ppargc1a, Mobkl3, Mapk11, Prlr, Socs7, Edem1, Dusp1, Pla2g15, Ptprd, Ptpru, Igdcc3, Rasgrp1, Tet3, Rb1, Rbpj, Rcn1, Rdx, Dpf2, Rgs16, Rnf7, Rorc, Sub1, Rrm2, Scd2, Scn5a, Scn8a, Msr1, Sema4c, Sema4f, Ostf1, St6gal1, St8sia1, Clpb, Skil, Slc31a1, Smarcc1, Snap23, Snn, Sox13, Sall3, Strbp, Dtx4, Wdr37, Gramd1c, Srebf2, Dot1l, Slk, Prrg3, Dock3, Cpeb3, Zc3hav1l, Stx3, Snx30, Zfp710, Igsf1, Lcorl, Tmc7, Syt1, Syt2, Tmem194, Trim41, Fam123c, Trib1, Plekhh1, Pde12, Zfyve26, Dcaf15, Lcor, Pqlc2, Mfsd4, Plekhg6, Tarbp2, Tbx5, Zfp354a, Nipal4, Tet2, Tcf7l1, Etnk2, Hip1, C330019G07Rik, Lrrc20, Cdc34, Eea1, 9-Mar, Pik3ip1, Fnip1, Tead3, Tead4, Wnt9a, Taok1, Hlf, Trib2, Pacs2, Tgfbr1, Tgfbr3, Thbs1, Thrsp, Tia1, Gcnt4, Marveld2, Timm17b, Slc4a7, Sec24c, D14Abb1e, Pcdh20, Tnfaip3, Tnfrsf1b, Usp12, Ugcg, Utrn, Farp1, Zc3h3, 5031439G07Rik, Gxylt1, Wnt1, Fam43a, Wnt9b, Impg2, Xk, Rab40c, Uhrf1bp1, Btbd9, Abcc10, Dlgap1, Map4k3, Thoc1, Ammecr1l, Apbb3, Cep120, Rnf165, Lrfn4, Trpm6, Fam178a, Yod1, Ahctf1, Zfp2, Vash2, B3gnt7, Ikzf2, Slc30a1, Slc35c1, Frmd5, Btbd3, Dlgap4, Ppp1r16b, Npepl1, Zbtb10, Ythdf3, Syt11, Pogz, Slc25a24, B230312A22Rik, Zbtb5, Ikbkap, Acer2, Slc5a9, Zfp362, Fras1, Frmd4b, Slc6a1, Dcun1d3, Psd3, Ddx19b, Tmem231, Adamts15, Zbtb16, Lingo1, Rab8b, Prtg, Ddx26b, Pcyt1b, Nxt2, Adamts14, Trhde, Ankrd52, Pla2g3, Ankrd43, Usp32, Adamts5, Akap6, Fam160b2, Adamts12, Rspo2, Mgll, Nek3, Smcr7l, Klhl6, Liph, Lmln, Gpr156, Arid1b, Scn11a, Ccnj, Slco5a1, Tor1aip2, Scyl3, Crb2, Tspan18, Slc7a14, Frem2, Zfp697, Gdf6, Zfp462, Klhl9, Gpatch3, E2f2, Napepld, Tmprss11f, Sbno1, Stard13, Styk1, Arhgap20, Tnfaip8l3, C230081A13Rik, Klhl31, Klf8, Trappc1, Onecut3, Srgap3, Gga3, Esrrb, Spata2, Map3k1, Mapk8, Sema4g, Homer2, Rab11fip4, Greb1, Slc38a9, 9930012K11Rik, B3galt1, Arhgap28, Dagla, Mgat4a, Cep110, Map4k4, Acvr1c, Znf512b, Trpc7, Lrig2, Vsnl1, Gpr157, Nat8l, AU019823, Rab6b, Mbtps2, Zfp275, Rpusd2, X99384, Dnajb9, Abcc5, Rasl10b, Nynrin, Klhl23, Efhd2, Ubfd1, D11Wsu99e, Limd1, Ulk2, Adamts8, Fbxl12, Fndc3a, Pld5, Dusp4, Hif1an, Igf2bp2, Apba1, Ercc6, Uggt1, Nceh1, Zbtb39, Zmat4, Lrrc58, Senp5, Iqcb1, Lrig3, Trank1, Ubn2, Phf8, Zbtb26, Pptc7, Slc26a9, Rfx6, Cdv3, Frs2, Pitpnm3, Gfod1, Zfp516, 8430427H17Rik, Fnip2, AI464131, Usp24, Acot11, Slc5a6, Sdk1, D630045J12Rik, Trim67, Thoc2, Kcnk18, Slc2a12, Col27a1, Serf2, Lin28b, Obscn, Begain, Mll2, Greb1l, 5930434B04Rik, Xkr8, Cep135, Pdp2, Scn4b, Fam84b, Taf9b, Gnptab, Nlrc5, Arhgef15, E2f6, Ero1l, Tmprss2, Dlc1, Mapk6, Dkk3, Tmod2, Rragd, Reep1, Atxn1l, Zfp644, Rufy3, Tmx4, D1Bwg0212e, Hif3a, Nap1l1, Cpsf4, Nme6, Slc4a4, Plagl2, Plxnc1, Arid3c, Rbm38, Tspan5, Styx, Mycbp, Abcb9, Arid3b, 1500003O03Rik, Syncrip, Ikbke, Elf4, Ankrd49, Rbms2, Nme4, Habp4, Crtap, Igdcc4, Vav3, Tob2, Psors1c2, Golga7, Cd200r1, Bin3, Hic2, Fam184b, Arpp19, Fign, Kctd21, Trim71, Ralb, Zfp280b, Suv39h2, Tsc1, Cldn12, Tmem167, Arrdc4, Pcp4l1, Nova1, Tnik, Gdpd1, Dzip1, Abi3, Atg4b, Tmem41a, Dcbld1, Spryd4, Slc16a9, 1200014J11Rik, Riok3, Bzw1, Bzw2, Prpf38b, Golt1b, Edem3, Nkap, Polr3d, Gatm, Lipt2, Cap2, Brd3, Ergic1, 1810074P20Rik, Ttll4, Stx17, Fbxo32, Gpatch2, Plxnd1, Limd2, Ubxn4, Gipc1, Zcchc3, Ccny, Trabd, Aen, Cgnl1, Fam135a, Leprotl1, AI846148, 0610030E20Rik, Adipor2, Nsmce2, Ppm1f, Letmd1, Tab2, Zfp740, Ddi2, Vps26b, 2200002K05Rik, Snx5, Tmem110, Pcgf3, Fam83g, Pxdn, Ints2, Slc35d2, Naa30, Dusp16, Tspan2, Prss22, Pgm2l1, Stox2, Zfp689, Golga7b, Col24a1, Ing3, Slc16a14, Cpa4, Slc25a18, Fbxo30, Ppapdc1b, Nkiras2, Ppp2r2a, Fndc3b, Cyb561d1, Tsc22d2, Cnot2, Lsm11, 2410042D21Rik, Ppme1, 2810030E01Rik, B3gat3, Kctd17, Slc22a23, Tmed5, Tbkbp1, Wasl, Xpot, Fam118a, 1700052N19Rik, Vgll3, Ppp2r1b, 1110012D08Rik, Slc25a27, Nphp3, Armc8, Sec14l1, Crct1, Stk40, Dtx2, 1700017B05Rik, Gale, 4931428F04Rik, Lonrf3, Ppapdc2, Zswim5, Snx16, Usp38, Tmem65, Usp47, Rab11fip2, Dpp3, Etnk1, Arl5a, Gns, Slc10a6, Klhl24, Senp2, 6230409E13Rik, Stard3nl, Tgds, Clasp2, Slc10a7, Eif2c4, Atg16l1, Bcl7a, Rnf170, Lbh, Hook1, Klhdc8b, Rictor, Dlst, Chsy3, Spnb4, Tusc2, Med8, Mesdc1, Zfp202, Arl4d, Ndst3, Ctns, Lin28a, Elovl4, Slc12a9, Tmem2, Kremen1, Sec16b, Entpd7, Gpr45, Brwd1, Cecr6, Trim33, Cnnm4, Qars, Lbr, Rdh10, Clp1, Cercam, Sall4, Kcnc4

mmu-miR-7c	Nhsl2, Nfxl1, Zfp282, Rpusd3, B630005N14Rik, A130022J15Rik, Fbxl14, Spred3, Agpat6, Dcun1d2, Cd276, Cdh22, Sbk1, Aldh6a1, Tecpr2, Rab15, Iars, Rnf44, Dusp22, AW549877, Dhx57, 2010002N04Rik, Gpr137, Unc5a, Lgr4, Chd4, Olr1, Prkaa2, Prkab2, Galnt2, B3gnt1, Uhrf2, Chd9, Rnf20, Txlna, Braf, Hdlbp, Lmx1a, Slc8a2, Hmga1-rs1, Egln2, Abcg1, Foxp2, Acta1, Acvr1b, Acvr2a, Adrb2, Adrb3, Ankfy1, Ap1s1, Speg, Aqp4, Bach1, Bcap29, Bcat1, Bcl2l1, Prdm1, Bsn, Cacna1d, Calm1, Cap1, Cask, Casp3, Cbx2, Cbx5, Ccnd1, Ccnd2, Cd86, Cdc25a, Chrd, Socs1, Tpp1, Cmah, Ccr7, Coil, Col14a1, Col15a1, Col3a1, Col4a1, Col4a2, Col4a5, Col5a2, Col1a2, Cpd, Crk, Cry2, Cyp46a1, Ddn, Dmd, Dmp1, Dnajc1, Arid3a, Dyrk1a, E2f5, Eda, Edn1, Ddx19a, Eif4g2, Epha3, Epha4, Esr2, Smarcad1, Igf2bp3, Ezh2, Fasl, Fgf4, Fgf5, Frk, Gabpa, Gabra6, Galnt1, Gas7, Mtpn, Nr6a1, Gdap2, Ghr, Gnal, Gng5, Grik2, Grin2b, Gtf2i, Hand1, Has2, Hic1, Hk2, Hmga1, Hmga2, Hoxd1, Htr4, Igf1r, Il10, Il13, Il6, Lrig1, Insr, Kcnc3, Khsrp, Kif21b, Klkb1, Kpna1, Lalba, Limk2, Sh2b3, Lor, Peg10, Slc24a6, Ppargc1b, Grid2ip, Mxd1, Masp1, Mdfi, Mllt10, Mmp11, Ndst2, Meis2, Meis3, Grpel2, Myo1f, Nefm, Ngf, Nid2, Nlk, Mycn, Nras, Nrtn, Ntn1, Osmr, P2rx1, P4ha2, Pldn, Pak1, Pappa, Pbx1, Pbx2, Pbx3, Pcx, Pdgfb, Piga, Pld3, Prrx1, Ppara, Ppargc1a, Mobkl3, Mapk11, Prlr, Socs7, Edem1, Dusp1, Pla2g15, Ptprd, Ptpru, Igdcc3, Rasgrp1, Tet3, Rb1, Rbpj, Rcn1, Rdx, Dpf2, Rgs16, Rnf7, Rorc, Sub1, Rrm2, Scd2, Scn5a, Scn8a, Msr1, Sema4c, Sema4f, Ostf1, St6gal1, St8sia1, Clpb, Skil, Slc31a1, Smarcc1, Snap23, Snn, Sox13, Sall3, Strbp, Dtx4, Wdr37, Gramd1c, Srebf2, Dot1l, Slk, Prrg3, Dock3, Cpeb3, Zc3hav1l, Stx3, Snx30, Zfp710, Igsf1, Lcorl, Tmc7, Syt1, Syt2, Tmem194, Trim41, Fam123c, Trib1, Plekhh1, Pde12, Zfyve26, Dcaf15, Lcor, Pqlc2, Mfsd4, Plekhg6, Tarbp2, Tbx5, Zfp354a, Nipal4, Tet2, Tcf7l1, Etnk2, Hip1, C330019G07Rik, Lrrc20, Cdc34, Eea1, 9-Mar, Pik3ip1, Fnip1, Tead3, Tead4, Wnt9a, Taok1, Hlf, Trib2, Pacs2, Tgfbr1, Tgfbr3, Thbs1, Thrsp, Tia1, Gcnt4, Marveld2, Timm17b, Slc4a7, Sec24c, D14Abb1e, Pcdh20, Tnfaip3, Tnfrsf1b, Usp12, Ugcg, Utrn, Farp1, Zc3h3, 5031439G07Rik, Gxylt1, Wnt1, Fam43a, Wnt9b, Impg2, Xk, Rab40c, Uhrf1bp1, Btbd9, Abcc10, Dlgap1, Map4k3, Thoc1, Ammecr1l, Apbb3, Cep120, Rnf165, Lrfn4, Trpm6, Fam178a, Yod1, Ahctf1, Zfp2, Vash2, B3gnt7, Ikzf2, Slc30a1, Slc35c1, Frmd5, Btbd3, Dlgap4, Ppp1r16b, Npepl1, Zbtb10, Ythdf3, Syt11, Pogz, Slc25a24, B230312A22Rik, Zbtb5, Ikbkap, Acer2, Slc5a9, Zfp362, Fras1, Frmd4b, Slc6a1, Dcun1d3, Psd3, Ddx19b, Tmem231, Adamts15, Zbtb16, Lingo1, Rab8b, Prtg, Ddx26b, Pcyt1b, Nxt2, Adamts14, Trhde, Ankrd52, Pla2g3, Ankrd43, Usp32, Adamts5, Akap6, Fam160b2, Adamts12, Rspo2, Mgll, Nek3, Smcr7l, Klhl6, Liph, Lmln, Gpr156, Arid1b, Scn11a, Ccnj, Slco5a1, Tor1aip2, Scyl3, Crb2, Tspan18, Slc7a14, Frem2, Zfp697, Gdf6, Zfp462, Klhl9, Gpatch3, E2f2, Napepld, Tmprss11f, Sbno1, Stard13, Styk1, Arhgap20, Tnfaip8l3, C230081A13Rik, Klhl31, Klf8, Trappc1, Onecut3, Srgap3, Gga3, Esrrb, Spata2, Map3k1, Mapk8, Sema4g, Homer2, Rab11fip4, Greb1, Slc38a9, 9930012K11Rik, B3galt1, Arhgap28, Dagla, Mgat4a, Cep110, Map4k4, Acvr1c, Znf512b, Trpc7, Lrig2, Vsnl1, Gpr157, Nat8l, AU019823, Rab6b, Mbtps2, Zfp275, Rpusd2, X99384, Dnajb9, Abcc5, Rasl10b, Nynrin, Klhl23, Efhd2, Ubfd1, D11Wsu99e, Limd1, Ulk2, Adamts8, Fbxl12, Fndc3a, Pld5, Dusp4, Hif1an, Igf2bp2, Apba1, Ercc6, Uggt1, Nceh1, Zbtb39, Zmat4, Lrrc58, Senp5, Iqcb1, Lrig3, Trank1, Ubn2, Phf8, Zbtb26, Pptc7, Slc26a9, Rfx6, Cdv3, Frs2, Pitpnm3, Gfod1, Zfp516, 8430427H17Rik, Fnip2, AI464131, Usp24, Acot11, Slc5a6, Sdk1, D630045J12Rik, Trim67, Thoc2, Kcnk18, Slc2a12, Col27a1, Serf2, Lin28b, Obscn, Begain, Mll2, Greb1l, 5930434B04Rik, Xkr8, Cep135, Pdp2, Scn4b, Fam84b, Taf9b, Gnptab, Nlrc5, Arhgef15, E2f6, Ero1l, Tmprss2, Dlc1, Mapk6, Dkk3, Tmod2, Rragd, Reep1, Atxn1l, Zfp644, Rufy3, Tmx4, D1Bwg0212e, Hif3a, Nap1l1, Cpsf4, Nme6, Slc4a4, Plagl2, Plxnc1, Arid3c, Rbm38, Tspan5, Styx, Mycbp, Abcb9, Arid3b, 1500003O03Rik, Syncrip, Ikbke, Elf4, Ankrd49, Rbms2, Nme4, Habp4, Crtap, Igdcc4, Vav3, Tob2, Psors1c2, Golga7, Cd200r1, Bin3, Hic2, Fam184b, Arpp19, Fign, Kctd21, Trim71, Ralb, Zfp280b, Suv39h2, Tsc1, Cldn12, Tmem167, Arrdc4, Pcp4l1, Nova1, Tnik, Gdpd1, Dzip1, Abi3, Atg4b, Tmem41a, Dcbld1, Spryd4, Slc16a9, 1200014J11Rik, Riok3, Bzw1, Bzw2, Prpf38b, Golt1b, Edem3, Nkap, Polr3d, Gatm, Lipt2, Cap2, Brd3, Ergic1, 1810074P20Rik, Ttll4, Stx17, Fbxo32, Gpatch2, Plxnd1, Limd2, Ubxn4, Gipc1, Zcchc3, Ccny, Trabd, Aen, Cgnl1, Fam135a, Leprotl1, AI846148, 0610030E20Rik, Adipor2, Nsmce2, Ppm1f, Letmd1, Tab2, Zfp740, Ddi2, Vps26b, 2200002K05Rik, Snx5, Tmem110, Pcgf3, Fam83g, Pxdn, Ints2, Slc35d2, Naa30, Dusp16, Tspan2, Prss22, Pgm2l1, Stox2, Zfp689, Golga7b, Col24a1, Ing3, Slc16a14, Cpa4, Slc25a18, Fbxo30, Ppapdc1b, Nkiras2, Ppp2r2a, Fndc3b, Cyb561d1, Tsc22d2, Cnot2, Lsm11, 2410042D21Rik, Ppme1, 2810030E01Rik, B3gat3, Kctd17, Slc22a23, Tmed5, Tbkbp1, Wasl, Xpot, Fam118a, 1700052N19Rik, Vgll3, Ppp2r1b, 1110012D08Rik, Slc25a27, Nphp3, Armc8, Sec14l1, Crct1, Stk40, Dtx2, 1700017B05Rik, Gale, 4931428F04Rik, Lonrf3, Ppapdc2, Zswim5, Snx16, Usp38, Tmem65, Usp47, Rab11fip2, Dpp3, Etnk1, Arl5a, Gns, Slc10a6, Klhl24, Senp2, 6230409E13Rik, Stard3nl, Tgds, Clasp2, Slc10a7, Eif2c4, Atg16l1, Bcl7a, Rnf170, Lbh, Hook1, Klhdc8b, Rictor, Dlst, Chsy3, Spnb4, Tusc2, Med8, Mesdc1, Zfp202, Arl4d, Ndst3, Ctns, Lin28a, Elovl4, Slc12a9, Tmem2, Kremen1, Sec16b, Entpd7, Gpr45, Brwd1, Cecr6, Trim33, Cnnm4, Qars, Lbr, Rdh10, Clp1, Cercam, Sall4, Kcnc4

mmu-miR-7e	Nhsl2, Nfxl1, Zfp282, Rpusd3, B630005N14Rik, A130022J15Rik, Fbxl14, Spred3, Agpat6, Dcun1d2, Cd276, Cdh22, Sbk1, Aldh6a1, Tecpr2, Rab15, Iars, Rnf44, Dusp22, AW549877, Dhx57, 2010002N04Rik, Gpr137, Unc5a, Lgr4, Chd4, Olr1, Prkaa2, Prkab2, Galnt2, B3gnt1, Uhrf2, Chd9, Rnf20, Txlna, Braf, Hdlbp, Lmx1a, Slc8a2, Hmga1-rs1, Egln2, Abcg1, Foxp2, Acta1, Acvr1b, Acvr2a, Adrb2, Adrb3, Ankfy1, Ap1s1, Speg, Aqp4, Bach1, Bcap29, Bcat1, Bcl2l1, Prdm1, Bsn, Cacna1d, Calm1, Cap1, Cask, Casp3, Cbx2, Cbx5, Ccnd1, Ccnd2, Cd86, Cdc25a, Chrd, Socs1, Tpp1, Cmah, Ccr7, Coil, Col14a1, Col15a1, Col3a1, Col4a1, Col4a2, Col4a5, Col5a2, Col1a2, Cpd, Crk, Cry2, Cyp46a1, Ddn, Dmd, Dmp1, Dnajc1, Arid3a, Dyrk1a, E2f5, Eda, Edn1, Ddx19a, Eif4g2, Epha3, Epha4, Esr2, Smarcad1, Igf2bp3, Ezh2, Fasl, Fgf4, Fgf5, Frk, Gabpa, Gabra6, Galnt1, Gas7, Mtpn, Nr6a1, Gdap2, Ghr, Gnal, Gng5, Grik2, Grin2b, Gtf2i, Hand1, Has2, Hic1, Hk2, Hmga1, Hmga2, Hoxd1, Htr4, Igf1r, Il10, Il13, Il6, Lrig1, Insr, Kcnc3, Khsrp, Kif21b, Klkb1, Kpna1, Lalba, Limk2, Sh2b3, Lor, Peg10, Slc24a6, Ppargc1b, Grid2ip, Mxd1, Masp1, Mdfi, Mllt10, Ndst2, Meis2, Meis3, Grpel2, Myo1f, Nefm, Ngf, Nid2, Nlk, Mycn, Nras, Nrtn, Ntn1, Osmr, P2rx1, P4ha2, Pldn, Pak1, Pappa, Pbx1, Pbx2, Pbx3, Pcx, Pdgfb, Piga, Pld3, Prrx1, Ppargc1a, Mobkl3, Mapk11, Prlr, Socs7, Edem1, Dusp1, Pla2g15, Ptprd, Ptpru, Igdcc3, Rasgrp1, Tet3, Rb1, Rbpj, Rcn1, Rdx, Dpf2, Rgs16, Rnf7, Rorc, Sub1, Rrm2, Scd2, Scn5a, Scn8a, Msr1, Sema4c, Sema4f, Ostf1, St6gal1, St8sia1, Clpb, Skil, Slc31a1, Smarcc1, Snap23, Snn, Sox13, Sall3, Strbp, Dtx4, Wdr37, Gramd1c, Srebf2, Dot1l, Slk, Prrg3, Dock3, Cpeb3, Zc3hav1l, Stx3, Snx30, Zfp710, Igsf1, Lcorl, Tmc7, Syt1, Syt2, Tmem194, Trim41, Fam123c, Trib1, Plekhh1, Pde12, Zfyve26, Dcaf15, Lcor, Pqlc2, Mfsd4, Plekhg6, Tarbp2, Tbx5, Zfp354a, Nipal4, Tet2, Tcf7l1, Etnk2, Hip1, C330019G07Rik, Lrrc20, Cdc34, Eea1, 9-Mar, Pik3ip1, Fnip1, Tead3, Tead4, Wnt9a, Taok1, Hlf, Trib2, Pacs2, Tgfbr1, Tgfbr3, Thbs1, Thrsp, Tia1, Gcnt4, Marveld2, Timm17b, Slc4a7, Sec24c, D14Abb1e, Pcdh20, Tnfaip3, Tnfrsf1b, Usp12, Ugcg, Utrn, Farp1, Zc3h3, 5031439G07Rik, Gxylt1, Wnt1, Fam43a, Wnt9b, Impg2, Xk, Rab40c, Uhrf1bp1, Btbd9, Abcc10, Dlgap1, Map4k3, Thoc1, Ammecr1l, Apbb3, Cep120, Rnf165, Lrfn4, Trpm6, Fam178a, Yod1, Ahctf1, Zfp2, Vash2, B3gnt7, Ikzf2, Slc30a1, Slc35c1, Frmd5, Btbd3, Dlgap4, Ppp1r16b, Npepl1, Zbtb10, Ythdf3, Syt11, Pogz, Slc25a24, B230312A22Rik, Zbtb5, Ikbkap, Acer2, Slc5a9, Zfp362, Fras1, Frmd4b, Slc6a1, Dcun1d3, Psd3, Ddx19b, Tmem231, Adamts15, Zbtb16, Lingo1, Rab8b, Prtg, Ddx26b, Pcyt1b, Nxt2, Adamts14, Trhde, Ankrd52, Pla2g3, Ankrd43, Usp32, Adamts5, Akap6, Fam160b2, Adamts12, Rspo2, Mgll, Nek3, Smcr7l, Klhl6, Liph, Lmln, Gpr156, Arid1b, Scn11a, Ccnj, Slco5a1, Tor1aip2, Scyl3, Crb2, Tspan18, Slc7a14, Frem2, Zfp697, Gdf6, Zfp462, Klhl9, Gpatch3, E2f2, Napepld, Tmprss11f, Sbno1, Stard13, Styk1, Arhgap20, Tnfaip8l3, C230081A13Rik, Klhl31, Klf8, Trappc1, Onecut3, Srgap3, Gga3, Esrrb, Spata2, Map3k1, Mapk8, Sema4g, Homer2, Rab11fip4, Greb1, Slc38a9, 9930012K11Rik, B3galt1, Arhgap28, Dagla, Mgat4a, Cep110, Map4k4, Acvr1c, Znf512b, Trpc7, Lrig2, Vsnl1, Gpr157, Nat8l, AU019823, Rab6b, Mbtps2, Zfp275, Rpusd2, X99384, Dnajb9, Abcc5, Rasl10b, Nynrin, Klhl23, Efhd2, Ubfd1, D11Wsu99e, Limd1, Ulk2, Adamts8, Fbxl12, Fndc3a, Pld5, Dusp4, Hif1an, Igf2bp2, Apba1, Ercc6, Uggt1, Nceh1, Zbtb39, Zmat4, Lrrc58, Senp5, Iqcb1, Lrig3, Trank1, Ubn2, Phf8, Zbtb26, Pptc7, Slc26a9, Rfx6, Cdv3, Frs2, Pitpnm3, Gfod1, Zfp516, 8430427H17Rik, Fnip2, AI464131, Usp24, Acot11, Slc5a6, Sdk1, D630045J12Rik, Trim67, Thoc2, Kcnk18, Slc2a12, Col27a1, Serf2, Lin28b, Obscn, Begain, Mll2, Greb1l, 5930434B04Rik, Xkr8, Cep135, Pdp2, Scn4b, Fam84b, Taf9b, Gnptab, Nlrc5, Arhgef15, E2f6, Ero1l, Tmprss2, Dlc1, Mapk6, Dkk3, Tmod2, Rragd, Reep1, Atxn1l, Zfp644, Rufy3, Tmx4, D1Bwg0212e, Hif3a, Nap1l1, Cpsf4, Nme6, Slc4a4, Plagl2, Plxnc1, Arid3c, Rbm38, Tspan5, Styx, Mycbp, Abcb9, Arid3b, 1500003O03Rik, Syncrip, Ikbke, Elf4, Ankrd49, Rbms2, Nme4, Habp4, Crtap, Igdcc4, Vav3, Tob2, Psors1c2, Golga7, Cd200r1, Bin3, Hic2, Fam184b, Arpp19, Fign, Kctd21, Trim71, Ralb, Zfp280b, Suv39h2, Tsc1, Cldn12, Tmem167, Arrdc4, Pcp4l1, Nova1, Tnik, Gdpd1, Dzip1, Abi3, Atg4b, Tmem41a, Spryd4, Slc16a9, 1200014J11Rik, Riok3, Bzw1, Bzw2, Prpf38b, Golt1b, Edem3, Nkap, Polr3d, Gatm, Lipt2, Cap2, Brd3, Ergic1, 1810074P20Rik, Ttll4, Stx17, Fbxo32, Gpatch2, Plxnd1, Limd2, Ubxn4, Gipc1, Zcchc3, Ccny, Trabd, Aen, Cgnl1, Fam135a, Leprotl1, AI846148, 0610030E20Rik, Adipor2, Nsmce2, Ppm1f, Letmd1, Tab2, Zfp740, Ddi2, Vps26b, 2200002K05Rik, Snx5, Tmem110, Pcgf3, Fam83g, Pxdn, Ints2, Slc35d2, Naa30, Dusp16, Tspan2, Prss22, Pgm2l1, Stox2, Zfp689, Golga7b, Col24a1, Ing3, Slc16a14, Cpa4, Slc25a18, Fbxo30, Ppapdc1b, Nkiras2, Ppp2r2a, Fndc3b, Cyb561d1, Tsc22d2, Cnot2, Lsm11, 2410042D21Rik, Ppme1, 2810030E01Rik, B3gat3, Kctd17, Slc22a23, Tmed5, Tbkbp1, Wasl, Xpot, Fam118a, 1700052N19Rik, Vgll3, Ppp2r1b, 1110012D08Rik, Slc25a27, Nphp3, Armc8, Sec14l1, Crct1, Stk40, Dtx2, 1700017B05Rik, Gale, 4931428F04Rik, Lonrf3, Ppapdc2, Zswim5, Snx16, Usp38, Tmem65, Usp47, Rab11fip2, Dpp3, Etnk1, Arl5a, Gns, Slc10a6, Klhl24, Senp2, 6230409E13Rik, Stard3nl, Tgds, Clasp2, Slc10a7, Eif2c4, Atg16l1, Bcl7a, Rnf170, Lbh, Hook1, Klhdc8b, Rictor, Dlst, Chsy3, Spnb4, Tusc2, Med8, Mesdc1, Zfp202, Arl4d, Ndst3, Ctns, Lin28a, Elovl4, Slc12a9, Tmem2, Kremen1, Sec16b, Entpd7, Gpr45, Brwd1, Cecr6, Trim33, Cnnm4, Qars, Lbr, Rdh10, Clp1, Cercam, Sall4, Kcnc4

mmu-miR-7f	Nhsl2, Nfxl1, Zfp282, Rpusd3, B630005N14Rik, A130022J15Rik, Fbxl14, Spred3, Agpat6, Dcun1d2, Cd276, Cdh22, Sbk1, Aldh6a1, Tecpr2, Rab15, Iars, Rnf44, Dusp22, AW549877, Dhx57, 2010002N04Rik, Gpr137, Unc5a, Lgr4, Chd4, Olr1, Prkaa2, Prkab2, Galnt2, B3gnt1, Uhrf2, Chd9, Rnf20, Txlna, Braf, Hdlbp, Lmx1a, Slc8a2, Hmga1-rs1, Egln2, Abcg1, Foxp2, Acta1, Acvr1b, Acvr2a, Adrb2, Adrb3, Ankfy1, Ap1s1, Speg, Aqp4, Bach1, Bcap29, Bcat1, Bcl2l1, Prdm1, Bsn, Cacna1d, Calm1, Cap1, Cask, Casp3, Cbx2, Cbx5, Ccnd1, Ccnd2, Cd86, Cdc25a, Chrd, Socs1, Tpp1, Cmah, Ccr7, Coil, Col14a1, Col15a1, Col3a1, Col4a1, Col4a2, Col4a5, Col5a2, Col1a2, Cpd, Crk, Cry2, Cyp46a1, Ddn, Dmd, Dmp1, Dnajc1, Arid3a, Dyrk1a, E2f5, Eda, Edn1, Ddx19a, Eif4g2, Epha3, Epha4, Esr2, Smarcad1, Igf2bp3, Ezh2, Fasl, Fgf4, Fgf5, Frk, Gabpa, Gabra6, Galnt1, Gas7, Mtpn, Nr6a1, Gdap2, Ghr, Gnal, Gng5, Grik2, Grin2b, Gtf2i, Hand1, Has2, Hic1, Hk2, Hmga1, Hmga2, Hoxd1, Htr4, Igf1r, Il10, Il13, Il6, Lrig1, Insr, Kcnc3, Khsrp, Kif21b, Klkb1, Kpna1, Lalba, Limk2, Sh2b3, Lor, Peg10, Slc24a6, Ppargc1b, Grid2ip, Mxd1, Masp1, Mdfi, Mllt10, Ndst2, Meis2, Meis3, Grpel2, Myo1f, Nefm, Ngf, Nid2, Nlk, Mycn, Nras, Nrtn, Ntn1, Osmr, P2rx1, P4ha2, Pldn, Pak1, Pappa, Pbx1, Pbx2, Pbx3, Pcx, Pdgfb, Piga, Pld3, Prrx1, Ppargc1a, Mobkl3, Mapk11, Prlr, Socs7, Edem1, Dusp1, Pla2g15, Ptprd, Ptpru, Igdcc3, Rasgrp1, Tet3, Rb1, Rbpj, Rcn1, Rdx, Dpf2, Rgs16, Rnf7, Rorc, Sub1, Rrm2, Scd2, Scn5a, Scn8a, Msr1, Sema4c, Sema4f, Ostf1, St6gal1, St8sia1, Clpb, Skil, Slc31a1, Smarcc1, Snap23, Snn, Sox13, Sall3, Strbp, Dtx4, Wdr37, Gramd1c, Srebf2, Dot1l, Slk, Prrg3, Dock3, Cpeb3, Zc3hav1l, Stx3, Snx30, Zfp710, Igsf1, Lcorl, Tmc7, Syt1, Syt2, Tmem194, Trim41, Fam123c, Trib1, Plekhh1, Pde12, Zfyve26, Dcaf15, Lcor, Pqlc2, Mfsd4, Plekhg6, Tarbp2, Tbx5, Zfp354a, Nipal4, Tet2, Tcf7l1, Etnk2, Hip1, C330019G07Rik, Lrrc20, Cdc34, Eea1, 9-Mar, Pik3ip1, Fnip1, Tead3, Tead4, Wnt9a, Taok1, Hlf, Trib2, Pacs2, Tgfbr1, Tgfbr3, Thbs1, Thrsp, Tia1, Gcnt4, Marveld2, Timm17b, Slc4a7, Sec24c, D14Abb1e, Pcdh20, Tnfaip3, Tnfrsf1b, Usp12, Ugcg, Utrn, Farp1, Zc3h3, 5031439G07Rik, Gxylt1, Wnt1, Fam43a, Wnt9b, Impg2, Xk, Rab40c, Uhrf1bp1, Btbd9, Abcc10, Dlgap1, Map4k3, Thoc1, Ammecr1l, Apbb3, Cep120, Rnf165, Lrfn4, Trpm6, Fam178a, Yod1, Ahctf1, Zfp2, Vash2, B3gnt7, Ikzf2, Slc30a1, Slc35c1, Frmd5, Btbd3, Dlgap4, Ppp1r16b, Npepl1, Zbtb10, Ythdf3, Syt11, Pogz, Slc25a24, B230312A22Rik, Zbtb5, Ikbkap, Acer2, Slc5a9, Zfp362, Fras1, Frmd4b, Slc6a1, Dcun1d3, Psd3, Ddx19b, Tmem231, Adamts15, Zbtb16, Lingo1, Rab8b, Prtg, Ddx26b, Pcyt1b, Nxt2, Adamts14, Trhde, Ankrd52, Pla2g3, Ankrd43, Usp32, Adamts5, Akap6, Fam160b2, Adamts12, Rspo2, Mgll, Nek3, Smcr7l, Klhl6, Liph, Lmln, Gpr156, Arid1b, Scn11a, Ccnj, Slco5a1, Tor1aip2, Scyl3, Crb2, Tspan18, Slc7a14, Frem2, Zfp697, Gdf6, Zfp462, Klhl9, Gpatch3, E2f2, Napepld, Tmprss11f, Sbno1, Stard13, Styk1, Arhgap20, Tnfaip8l3, C230081A13Rik, Klhl31, Klf8, Trappc1, Onecut3, Srgap3, Gga3, Esrrb, Spata2, Map3k1, Mapk8, Sema4g, Homer2, Rab11fip4, Greb1, Slc38a9, 9930012K11Rik, B3galt1, Arhgap28, Dagla, Mgat4a, Cep110, Map4k4, Acvr1c, Znf512b, Trpc7, Lrig2, Vsnl1, Gpr157, Nat8l, AU019823, Rab6b, Mbtps2, Zfp275, Rpusd2, X99384, Dnajb9, Abcc5, Rasl10b, Nynrin, Klhl23, Efhd2, Ubfd1, D11Wsu99e, Limd1, Ulk2, Adamts8, Fbxl12, Fndc3a, Pld5, Dusp4, Hif1an, Igf2bp2, Apba1, Ercc6, Uggt1, Nceh1, Zbtb39, Zmat4, Lrrc58, Senp5, Iqcb1, Lrig3, Trank1, Ubn2, Phf8, Zbtb26, Pptc7, Slc26a9, Rfx6, Cdv3, Frs2, Pitpnm3, Gfod1, Zfp516, 8430427H17Rik, Fnip2, AI464131, Usp24, Acot11, Slc5a6, Sdk1, D630045J12Rik, Trim67, Thoc2, Kcnk18, Slc2a12, Col27a1, Serf2, Lin28b, Obscn, Begain, Mll2, Greb1l, 5930434B04Rik, Xkr8, Cep135, Pdp2, Scn4b, Fam84b, Taf9b, Gnptab, Nlrc5, Arhgef15, E2f6, Ero1l, Tmprss2, Dlc1, Mapk6, Dkk3, Tmod2, Rragd, Reep1, Atxn1l, Zfp644, Rufy3, Tmx4, D1Bwg0212e, Hif3a, Nap1l1, Cpsf4, Nme6, Slc4a4, Plagl2, Plxnc1, Arid3c, Rbm38, Tspan5, Styx, Mycbp, Abcb9, Arid3b, 1500003O03Rik, Syncrip, Ikbke, Elf4, Ankrd49, Rbms2, Nme4, Habp4, Crtap, Igdcc4, Vav3, Tob2, Psors1c2, Golga7, Cd200r1, Bin3, Hic2, Fam184b, Arpp19, Fign, Kctd21, Trim71, Ralb, Zfp280b, Suv39h2, Tsc1, Cldn12, Tmem167, Arrdc4, Pcp4l1, Nova1, Tnik, Gdpd1, Dzip1, Abi3, Atg4b, Tmem41a, Spryd4, Slc16a9, 1200014J11Rik, Riok3, Bzw1, Bzw2, Prpf38b, Golt1b, Edem3, Nkap, Polr3d, Gatm, Lipt2, Cap2, Brd3, Ergic1, 1810074P20Rik, Ttll4, Stx17, Fbxo32, Gpatch2, Plxnd1, Limd2, Ubxn4, Gipc1, Zcchc3, Ccny, Trabd, Aen, Cgnl1, Fam135a, Leprotl1, AI846148, 0610030E20Rik, Adipor2, Nsmce2, Ppm1f, Letmd1, Tab2, Zfp740, Ddi2, Vps26b, 2200002K05Rik, Snx5, Tmem110, Pcgf3, Fam83g, Pxdn, Ints2, Slc35d2, Naa30, Dusp16, Tspan2, Prss22, Pgm2l1, Stox2, Zfp689, Golga7b, Col24a1, Ing3, Slc16a14, Cpa4, Slc25a18, Fbxo30, Ppapdc1b, Nkiras2, Ppp2r2a, Fndc3b, Cyb561d1, Tsc22d2, Cnot2, Lsm11, 2410042D21Rik, Ppme1, 2810030E01Rik, B3gat3, Kctd17, Slc22a23, Tmed5, Tbkbp1, Wasl, Xpot, Fam118a, 1700052N19Rik, Vgll3, Ppp2r1b, 1110012D08Rik, Slc25a27, Nphp3, Armc8, Sec14l1, Crct1, Stk40, Dtx2, 1700017B05Rik, Gale, 4931428F04Rik, Lonrf3, Ppapdc2, Zswim5, Snx16, Usp38, Tmem65, Usp47, Rab11fip2, Dpp3, Etnk1, Arl5a, Gns, Slc10a6, Klhl24, Senp2, 6230409E13Rik, Stard3nl, Tgds, Clasp2, Slc10a7, Eif2c4, Atg16l1, Bcl7a, Rnf170, Lbh, Hook1, Klhdc8b, Rictor, Dlst, Chsy3, Spnb4, Tusc2, Med8, Mesdc1, Zfp202, Arl4d, Ndst3, Ctns, Lin28a, Elovl4, Slc12a9, Tmem2, Kremen1, Sec16b, Entpd7, Gpr45, Brwd1, Cecr6, Trim33, Cnnm4, Qars, Lbr, Rdh10, Clp1, Cercam, Sall4, Kcnc4

mmu-miR-98	Nhsl2, Nfxl1, Zfp282, Rpusd3, B630005N14Rik, A130022J15Rik, Fbxl14, Spred3, Agpat6, Dcun1d2, Cd276, Cdh22, Sbk1, Aldh6a1, Tecpr2, Rab15, Iars, Rnf44, Dusp22, AW549877, Dhx57, 2010002N04Rik, Gpr137, Unc5a, Lgr4, Chd4, Olr1, Prkaa2, Prkab2, Galnt2, B3gnt1, Uhrf2, Chd9, Rnf20, Txlna, Braf, Hdlbp, Lmx1a, Slc8a2, Hmga1-rs1, Egln2, Abcg1, Foxp2, Acta1, Acvr1b, Acvr2a, Adrb2, Adrb3, Ankfy1, Ap1s1, Speg, Aqp4, Bach1, Bcap29, Bcat1, Bcl2l1, Prdm1, Bsn, Cacna1d, Calm1, Cap1, Cask, Casp3, Cbx2, Cbx5, Ccnd1, Ccnd2, Cd86, Cdc25a, Chrd, Socs1, Tpp1, Cmah, Ccr7, Coil, Col14a1, Col15a1, Col3a1, Col4a1, Col4a2, Col4a5, Col5a2, Col1a2, Cpd, Crk, Cry2, Cyp46a1, Ddn, Dmd, Dmp1, Dnajc1, Arid3a, Dyrk1a, E2f5, Eda, Edn1, Ddx19a, Eif4g2, Epha3, Epha4, Esr2, Smarcad1, Igf2bp3, Ezh2, Fasl, Fgf4, Fgf5, Frk, Gabpa, Gabra6, Galnt1, Gas7, Mtpn, Nr6a1, Gdap2, Ghr, Gnal, Gng5, Grik2, Grin2b, Gtf2i, Hand1, Has2, Hic1, Hk2, Hmga1, Hmga2, Hoxd1, Htr4, Igf1r, Il10, Il13, Il6, Lrig1, Insr, Kcnc3, Khsrp, Kif21b, Klkb1, Kpna1, Lalba, Limk2, Sh2b3, Lor, Peg10, Slc24a6, Ppargc1b, Grid2ip, Mxd1, Masp1, Mdfi, Mllt10, Ndst2, Meis2, Meis3, Grpel2, Myo1f, Nefm, Ngf, Nid2, Nlk, Mycn, Nras, Nrtn, Ntn1, Osmr, P2rx1, P4ha2, Pldn, Pak1, Pappa, Pbx1, Pbx2, Pbx3, Pcx, Pdgfb, Piga, Pld3, Prrx1, Ppara, Ppargc1a, Mobkl3, Mapk11, Prlr, Socs7, Edem1, Dusp1, Pla2g15, Ptprd, Ptpru, Igdcc3, Rasgrp1, Tet3, Rb1, Rbpj, Rcn1, Rdx, Dpf2, Rgs16, Rnf7, Rorc, Sub1, Rrm2, Scd2, Scn5a, Scn8a, Msr1, Sema4c, Sema4f, Ostf1, St6gal1, St8sia1, Clpb, Skil, Slc31a1, Smarcc1, Snap23, Snn, Sox13, Sall3, Strbp, Dtx4, Wdr37, Gramd1c, Srebf2, Dot1l, Slk, Prrg3, Dock3, Cpeb3, Zc3hav1l, Stx3, Snx30, Zfp710, Igsf1, Lcorl, Tmc7, Syt1, Syt2, Tmem194, Trim41, Fam123c, Trib1, Plekhh1, Pde12, Zfyve26, Dcaf15, Lcor, Pqlc2, Mfsd4, Plekhg6, Tarbp2, Tbx5, Zfp354a, Nipal4, Tet2, Tcf7l1, Etnk2, Hip1, C330019G07Rik, Lrrc20, Cdc34, Eea1, 9-Mar, Pik3ip1, Fnip1, Tead3, Tead4, Wnt9a, Taok1, Hlf, Trib2, Pacs2, Tgfbr1, Tgfbr3, Thbs1, Thrsp, Tia1, Gcnt4, Marveld2, Timm17b, Slc4a7, Sec24c, D14Abb1e, Pcdh20, Tnfaip3, Tnfrsf1b, Usp12, Ugcg, Utrn, Farp1, Zc3h3, 5031439G07Rik, Gxylt1, Wnt1, Fam43a, Wnt9b, Impg2, Xk, Rab40c, Uhrf1bp1, Btbd9, Abcc10, Dlgap1, Map4k3, Thoc1, Ammecr1l, Apbb3, Cep120, Rnf165, Lrfn4, Trpm6, Fam178a, Yod1, Ahctf1, Zfp2, Vash2, B3gnt7, Ikzf2, Slc30a1, Slc35c1, Frmd5, Btbd3, Dlgap4, Ppp1r16b, Npepl1, Zbtb10, Ythdf3, Syt11, Pogz, Slc25a24, B230312A22Rik, Zbtb5, Ikbkap, Acer2, Slc5a9, Zfp362, Fras1, Frmd4b, Slc6a1, Dcun1d3, Psd3, Ddx19b, Tmem231, Adamts15, Zbtb16, Lingo1, Rab8b, Prtg, Ddx26b, Pcyt1b, Nxt2, Adamts14, Trhde, Ankrd52, Pla2g3, Ankrd43, Usp32, Adamts5, Akap6, Fam160b2, Adamts12, Rspo2, Mgll, Nek3, Smcr7l, Klhl6, Liph, Lmln, Gpr156, Arid1b, Scn11a, Ccnj, Slco5a1, Tor1aip2, Scyl3, Crb2, Tspan18, Slc7a14, Frem2, Zfp697, Gdf6, Zfp462, Klhl9, Gpatch3, E2f2, Napepld, Tmprss11f, Sbno1, Stard13, Styk1, Arhgap20, Tnfaip8l3, C230081A13Rik, Klhl31, Klf8, Trappc1, Onecut3, Srgap3, Gga3, Esrrb, Spata2, Map3k1, Mapk8, Sema4g, Homer2, Rab11fip4, Greb1, Slc38a9, 9930012K11Rik, B3galt1, Arhgap28, Dagla, Mgat4a, Cep110, Map4k4, Acvr1c, Znf512b, Trpc7, Lrig2, Vsnl1, Gpr157, Nat8l, AU019823, Rab6b, Mbtps2, Zfp275, Rpusd2, X99384, Dnajb9, Abcc5, Rasl10b, Nynrin, Klhl23, Efhd2, Ubfd1, D11Wsu99e, Limd1, Ulk2, Adamts8, Fbxl12, Fndc3a, Pld5, Dusp4, Hif1an, Igf2bp2, Apba1, Ercc6, Uggt1, Nceh1, Zbtb39, Zmat4, Lrrc58, Senp5, Iqcb1, Lrig3, Trank1, Ubn2, Phf8, Zbtb26, Pptc7, Slc26a9, Rfx6, Cdv3, Frs2, Pitpnm3, Gfod1, Zfp516, 8430427H17Rik, Fnip2, AI464131, Usp24, Acot11, Slc5a6, Sdk1, D630045J12Rik, Trim67, Thoc2, Kcnk18, Slc2a12, Col27a1, Serf2, Lin28b, Obscn, Begain, Mll2, Greb1l, 5930434B04Rik, Xkr8, Cep135, Pdp2, Scn4b, Fam84b, Taf9b, Gnptab, Nlrc5, Arhgef15, E2f6, Ero1l, Tmprss2, Dlc1, Mapk6, Dkk3, Tmod2, Rragd, Reep1, Atxn1l, Zfp644, Rufy3, Tmx4, D1Bwg0212e, Hif3a, Nap1l1, Cpsf4, Nme6, Slc4a4, Plagl2, Plxnc1, Arid3c, Rbm38, Tspan5, Styx, Mycbp, Abcb9, Arid3b, 1500003O03Rik, Syncrip, Ikbke, Ankrd49, Rbms2, Nme4, Habp4, Crtap, Igdcc4, Vav3, Tob2, Psors1c2, Golga7, Cd200r1, Bin3, Hic2, Fam184b, Arpp19, Fign, Kctd21, Trim71, Ralb, Zfp280b, Suv39h2, Tsc1, Cldn12, Tmem167, Arrdc4, Pcp4l1, Nova1, Tnik, Gdpd1, Dzip1, Abi3, Atg4b, Tmem41a, Dcbld1, Spryd4, Slc16a9, 1200014J11Rik, Riok3, Bzw1, Bzw2, Prpf38b, Golt1b, Edem3, Nkap, Polr3d, Gatm, Lipt2, Cap2, Brd3, Ergic1, 1810074P20Rik, Ttll4, Stx17, Fbxo32, Gpatch2, Plxnd1, Limd2, Ubxn4, Gipc1, Zcchc3, Ccny, Trabd, Aen, Cgnl1, Fam135a, Leprotl1, AI846148, 0610030E20Rik, Adipor2, Nsmce2, Ppm1f, Letmd1, Tab2, Zfp740, Ddi2, Vps26b, 2200002K05Rik, Snx5, Tmem110, Pcgf3, Fam83g, Pxdn, Ints2, Slc35d2, Naa30, Dusp16, Tspan2, Prss22, Pgm2l1, Stox2, Zfp689, Golga7b, Col24a1, Ing3, Slc16a14, Cpa4, Slc25a18, Fbxo30, Ppapdc1b, Nkiras2, Ppp2r2a, Fndc3b, Cyb561d1, Tsc22d2, Cnot2, Lsm11, 2410042D21Rik, Ppme1, 2810030E01Rik, B3gat3, Kctd17, Slc22a23, Tmed5, Tbkbp1, Wasl, Xpot, Fam118a, 1700052N19Rik, Vgll3, Ppp2r1b, 1110012D08Rik, Slc25a27, Nphp3, Armc8, Sec14l1, Crct1, Stk40, Dtx2, 1700017B05Rik, Gale, 4931428F04Rik, Lonrf3, Ppapdc2, Zswim5, Snx16, Usp38, Tmem65, Usp47, Rab11fip2, Dpp3, Etnk1, Arl5a, Gns, Slc10a6, Klhl24, Senp2, 6230409E13Rik, Stard3nl, Tgds, Clasp2, Slc10a7, Eif2c4, Atg16l1, Bcl7a, Rnf170, Lbh, Hook1, Klhdc8b, Rictor, Dlst, Chsy3, Spnb4, Tusc2, Med8, Mesdc1, Zfp202, Arl4d, Ndst3, Ctns, Lin28a, Elovl4, Slc12a9, Tmem2, Kremen1, Sec16b, Entpd7, Gpr45, Brwd1, Cecr6, Trim33, Cnnm4, Qars, Lbr, Rdh10, Clp1, Cercam, Sall4, Kcnc4

Supplementary Table 3. Gene-ontology (GO) terms enriched by miRNAs differentially expressed in LTP.

**Up-regulated miRNAs:**			
**GO ID (cellular Component)**	**Go Term (Cellular Component GO Term)**	**Gene Symbol (genes enriched by up-regulated miRNAs)**	**Up-regulated miRNAs**
GO:0000267	cell fraction	Casp3, Dock3, Dusp1, Dusp4, Ercc6, Fgf4, Limk2, Mapk11, Pcx, Pla2g15, Rrm2, Smarcc1, Tab2, Tpp1	mmu-let-7a, mmu-let-7b, mmu-let-7c, mmu-let-7e, mmu-let-7f, mmu-miR-98
GO:0000307	cyclin-dependent protein kinase holoenzyme complex	Ccnd1, Ccnd2, Ccny	mmu-let-7a, mmu-let-7b, mmu-let-7c, mmu-let-7e, mmu-let-7f, mmu-miR-98
GO:0000445	THO complex part of transcription export complex	Thoc1, Thoc2	mmu-let-7a, mmu-let-7b, mmu-let-7c, mmu-let-7e, mmu-let-7f, mmu-miR-98
GO:0001518	voltage-gated sodium channel complex	Scn11a, Scn4b, Scn5a, Scn8a	mmu-let-7a, mmu-let-7b, mmu-let-7c, mmu-let-7e, mmu-let-7f, mmu-miR-98
GO:0001950	plasma membrane enriched fraction	Snap23, Stx3	mmu-let-7a, mmu-let-7b, mmu-let-7c, mmu-let-7e, mmu-let-7f, mmu-miR-98
GO:0005578	proteinaceous extracellular matrix	Adamts12, Adamts15, Adamts5, Adamts8, Crtap, Dmp1, Impg2, Pxdn, Tgfbr3, Wnt1, Wnt9a, Wnt9b, Mmp11	mmu-let-7a, mmu-let-7b, mmu-let-7c, mmu-let-7e, mmu-let-7f, mmu-miR-98
GO:0005581	collagen	Col14a1, Col15a1, Col24a1, Col4a1, Col4a2, Col4a5, Eda, Msr1, P4ha2	mmu-let-7a, mmu-let-7b, mmu-let-7c, mmu-let-7e, mmu-let-7f, mmu-miR-98
GO:0005583	fibrillar collagen	Col1a2, Col27a1, Col5a2	mmu-let-7a, mmu-let-7b, mmu-let-7c, mmu-let-7e, mmu-let-7f, mmu-miR-98

GO:0005586	collagen type III	Col3a1	mmu-let-7a, mmu-let-7b, mmu-let-7c, mmu-let-7e, mmu-let-7f, mmu-miR-98
GO:0005587	collagen type IV	Col4a1, Col4a2, Col4a5	mmu-let-7a, mmu-let-7b, mmu-let-7c, mmu-let-7e, mmu-let-7f, mmu-miR-98
GO:0005604	basement membrane	Cask, Col15a1, Fras1, Frem2, Nid2, Ntn1	mmu-let-7a, mmu-let-7b, mmu-let-7c, mmu-let-7e, mmu-let-7f, mmu-miR-98
GO:0005622	intracellular	Apba1, Arhgap20, Arhgef15, Arl5a, Cacna1d, Ccr7, Col1a2, Gpatch2, Gpatch3, Igf1r, Il10, Lingo1, Map4k3, Nras, Plxnc1, Prlr, Ralb, Rasl10b, Sh2b3, Srgap3, Yod1	mmu-let-7a, mmu-let-7b, mmu-let-7c, mmu-let-7e, mmu-let-7f, mmu-miR-98
GO:0005643	nuclear pore	Ahctf1, Ddx19a, Lbr, Senp2	mmu-let-7a, mmu-let-7b, mmu-let-7c, mmu-let-7e, mmu-let-7f, mmu-miR-98
GO:0005652	nuclear lamina	Cask, Lbr	mmu-let-7a, mmu-let-7b, mmu-let-7c, mmu-let-7e, mmu-let-7f, mmu-miR-98
GO:0005667	transcription factor complex	E2f2, E2f5, Foxp2, Nr6a1, Onecut3, Pbx1, Pbx2, Pbx3, Rb1, Rbpj, Sall4, Sub1, Taf9b, Tbx5, Tcf7l1, Tead3, Tead4	mmu-let-7a, mmu-let-7b, mmu-let-7c, mmu-let-7e, mmu-let-7f, mmu-miR-98
GO:0005730	nucleolus	2410042D21Rik, Aen, Brwd1, Cask, Cbx5, Ccnd2, Cdv3, Coil, Dmp1, Dnajb9, Dyrk1a, Ercc6, Hand1, Ikbkap, Lbh, Lin28a, Phf8, Rbpj, Rdx, Rnf20, Sub1, Wasl, Zfp354a	mmu-let-7a, mmu-let-7b, mmu-let-7c, mmu-let-7e, mmu-let-7f, mmu-miR-98
GO:0005801	cis-Golgi network	B3gat3, Limk2, Tmed5, Trappc1	mmu-let-7a, mmu-let-7b, mmu-let-7c, mmu-let-7e, mmu-let-7f, mmu-miR-98
GO:0005901	caveola	Adrb2, Dlc1, Fasl, Igf1r, Insr	mmu-let-7a, mmu-let-7b, mmu-let-7c, mmu-let-7e, mmu-let-7f, mmu-miR-98

GO:0005969	serine-pyruvate aminotransferase complex	Eea1	mmu-let-7a, mmu-let-7b, mmu-let-7c, mmu-let-7e, mmu-let-7f, mmu-miR-98
GO:0012505	endomembrane system	Abcb9, Acer2, Agpat6, Ahctf1, AI464131, Akap6, Ap1s1, B3galt1, Ccnd2, Chsy3, Ddx19a, E2f5, Eda, Edem1, Elovl4, Ero1l, Fndc3a, Gipc1, Golga7, Lbr, Nat8l, Pak1, Piga, Rrm2, Sema4c, Senp2, Slc5a6, Srebf2, St6gal1, St8sia1, Stx17, Syt1, Vps26b, Wasl	mmu-let-7a, mmu-let-7b, mmu-let-7c, mmu-let-7e, mmu-let-7f, mmu-miR-98
GO:0016442	RNA-induced silencing complex	Limd1, Tarbp2	mmu-let-7a, mmu-let-7b, mmu-let-7c, mmu-let-7e, mmu-let-7f, mmu-miR-98
GO:0016605	PML body	Ikbke, Rb1, Senp2, Skil, Spnb4, Zbtb16, Elf4	mmu-let-7a, mmu-let-7b, mmu-let-7c, mmu-let-7e, mmu-let-7f, mmu-miR-98
GO:0019717	synaptosome	Braf, Bsn, Cask, Dlgap4, Grid2ip, Grin2b, P2rx1, Sema4f, Slc6a1, Snap23	mmu-let-7a, mmu-let-7b, mmu-let-7c, mmu-let-7e, mmu-let-7f, mmu-miR-98
GO:0019898	extrinsic to membrane	Eea1, Frmd5, Ghr, Gnal, Olr1, Rdx, Snx16, Snx5	mmu-let-7a, mmu-let-7b, mmu-let-7c, mmu-let-7e, mmu-let-7f, mmu-miR-98
GO:0030054	cell junction	Aqp4, Bsn, C230081A13Rik, Cask, Cgnl1, Cldn12, Dlc1, Dlgap1, Dmd, Epha4, Gabra6, Gpr156, Grid2ip, Grik2, Grin2b, Hic2, Homer2, Lmln, Marveld2, Pak1, Psd3, Ptpru, Scn5a, Sema4c, Snap23, Stx3, Syt1, Syt11, Syt2, Tgfbr1	mmu-let-7a, mmu-let-7b, mmu-let-7c, mmu-let-7e, mmu-let-7f, mmu-miR-98
GO:0030176	integral to endoplasmic reticulum membrane	Abcb9, Agpat6, Edem1, Elovl4, Ero1l, Srebf2	mmu-let-7a, mmu-let-7b, mmu-let-7c, mmu-let-7e, mmu-let-7f, mmu-miR-98
GO:0030313	cell envelope	Grin2b, P2rx1	mmu-let-7a, mmu-let-7b, mmu-let-7c, mmu-let-7e, mmu-let-7f, mmu-miR-98

GO:0030424	axon	Adrb2, Epha4, Grik2, Grin2b, Kcnc3, Kcnc4, Mapk8, Mtpn, Nefm, Nrtn, Pak1, Scn11a, Scn8a, Slc6a1, Spnb4, Tnfrsf1b	mmu-let-7a, mmu-let-7b, mmu-let-7c, mmu-let-7e, mmu-let-7f, mmu-miR-98
GO:0030425	dendrite	Adrb2, Arhgef15, Cask, Ddn, Dlgap4, Epha4, Gabra6, Gipc1, Grid2ip, Grik2, Grin2b, Homer2, Khsrp, Mapk8, Pak1, Scn8a, Sema4c, Slc8a2, Srebf2	mmu-let-7a, mmu-let-7b, mmu-let-7c, mmu-let-7e, mmu-let-7f, mmu-miR-98
GO:0030864	cortical actin cytoskeleton	Cap1, Myo1f, Spnb4, Utrn, Wasl	mmu-let-7a, mmu-let-7b, mmu-let-7c, mmu-let-7e, mmu-let-7f, mmu-miR-98
GO:0031226	intrinsic to plasma membrane	Acvr1b, Acvr1c, Adrb2, Aqp4, Cacna1d, Eda, Epha3, Epha4, Gabra6, Ghr, Grik2, Grin2b, Igdcc4, Il6, Insr, Kcnc3, Kcnc4, Kctd21, Osmr, Plxnd1, Scn11a, Scn4b, Scn5a, Scn8a, Slc4a4, Slc6a1, Syt11, Tgfbr3, Tmprss11f	mmu-let-7a, mmu-let-7b, mmu-let-7c, mmu-let-7e, mmu-let-7f, mmu-miR-98
GO:0031240	external side of cell outer membrane	P2rx1	mmu-let-7a, mmu-let-7b, mmu-let-7c, mmu-let-7e, mmu-let-7f, mmu-miR-98
GO:0031300	intrinsic to organelle membrane	Abcb9, Acer2, Agpat6, Edem1, Elovl4, Ero1l, Srebf2, St6gal1, St8sia1, Timm17b	mmu-let-7a, mmu-let-7b, mmu-let-7c, mmu-let-7e, mmu-let-7f, mmu-miR-98
GO:0031313	extrinsic to endosome membrane	Snx16, Snx5	mmu-let-7a, mmu-let-7b, mmu-let-7c, mmu-let-7e, mmu-let-7f, mmu-miR-98
GO:0031588	AMP-activated protein kinase complex	Prkaa2, Prkab2	mmu-let-7a, mmu-let-7b, mmu-let-7c, mmu-let-7e, mmu-let-7f, mmu-miR-98
GO:0031594	neuromuscular junction	Col4a5, Epha4, Kcnc3, Kcnc4, Nefm, Utrn	mmu-let-7a, mmu-let-7b, mmu-let-7c, mmu-let-7e, mmu-let-7f, mmu-miR-98
GO:0032809	neuronal cell body membrane	Adrb2, Gabra6, Kcnc3	mmu-let-7a, mmu-let-7b, mmu-let-7c, mmu-let-7e, mmu-let-7f, mmu-miR-98

GO:0034673	inhibin-betaglycan-ActRII complex	Acvr2a, Tgfbr3	mmu-let-7a, mmu-let-7b, mmu-let-7c, mmu-let-7e, mmu-let-7f, mmu-miR-98
GO:0034703	cation channel complex	Akap6, Cacna1d, Kcnc3, Kcnc4, Kctd21, Scn11a, Scn4b, Scn5a, Scn8a	mmu-let-7a, mmu-let-7b, mmu-let-7c, mmu-let-7e, mmu-let-7f, mmu-miR-98
GO:0035985	senescence-associated heterochromatin focus	Hmga1, Hmga2	mmu-let-7a, mmu-let-7b, mmu-let-7c, mmu-let-7e, mmu-let-7f, mmu-miR-98
GO:0042581	specific granule	Snap23, Stx3	mmu-let-7a, mmu-let-7b, mmu-let-7c, mmu-let-7e, mmu-let-7f, mmu-miR-98
GO:0042582	azurophil granule	Snap23, Stx3	mmu-let-7a, mmu-let-7b, mmu-let-7c, mmu-let-7e, mmu-let-7f, mmu-miR-98
GO:0042598	vesicular fraction	Adrb2, Cpd, Dlst, Dmd, Dnajc1, Fndc3a, Igf1r, Insr, Nat8l, Rdh10, Snap23, Srebf2, Syt2, Tsc1	mmu-let-7a, mmu-let-7b, mmu-let-7c, mmu-let-7e, mmu-let-7f, mmu-miR-98
GO:0042734	presynaptic membrane	Gabra6, Grik2, Grin2b, Syt1	mmu-let-7a, mmu-let-7b, mmu-let-7c, mmu-let-7e, mmu-let-7f, mmu-miR-98
GO:0043005	neuron projection	Bsn, Dmd, Esr2, Igf1r, Rufy3, Stx3, Syt1, Tmod2, Tsc1, Utrn	mmu-let-7a, mmu-let-7b, mmu-let-7c, mmu-let-7e, mmu-let-7f, mmu-miR-98
GO:0043235	receptor complex	Acvr1c, Adrb2, Adrb3, Gabra6, Grik2, Grin2b, Il6, Insr, Osmr, Tgfbr1	mmu-let-7a, mmu-let-7b, mmu-let-7c, mmu-let-7e, mmu-let-7f, mmu-miR-98
GO:0044432	endoplasmic reticulum part	Abcb9, Agpat6, Dnajc1, Eda, Edem1, Edem3, Elovl4, Ero1l, Nat8l, Piga, Srebf2, Stx17, Uggt1	mmu-let-7a, mmu-let-7b, mmu-let-7c, mmu-let-7e, mmu-let-7f, mmu-miR-98

GO:0044451	nucleoplasm part	Cbx5, Chd4, Coil, Cpsf4, Dyrk1a, E2f6, Ercc6, Ezh2, Ikbkap, Ikbke, Ing3, Ints2, Med8, Mll2, Polr3d, Ppargc1b, Senp2, Skil, Spnb4, Zbtb16, Elf4	mmu-let-7a, mmu-let-7b, mmu-let-7c, mmu-let-7e, mmu-let-7f, mmu-miR-98
GO:0044459	plasma membrane part	Abcg1, C230081A13Rik, Cask, Ccr7, Cd200r1, Cd276, Cd86, Cgnl1, Clasp2, Cldn12, Dmd, Eea1, Gnal, Hic2, Il13, Lmln, Lor, Marveld2, Olr1, Pak1, Pdgfb, Prkaa2, Prkab2, Slc26a9, Slc30a1, Slc4a7, Slc5a6, Snx5, Stx3, Tgfbr1, Thbs1, Tmem231, Trpm6, Utrn	mmu-let-7a, mmu-let-7b, mmu-let-7c, mmu-let-7e, mmu-let-7f, mmu-miR-98
GO:0044463	cell projection part	Adrb2, Bsn, Clasp2, Dmd, Epha4, Gipc1, Grid2ip, Grik2, Grin2b, Kcnc3, Kcnc4, Mapk8, Nefm, Pak1, Rufy3, Scn8a, Sema4c, Slc5a6, Slc8a2, Spnb4, Stx3, Tmem231, Tmod2, Tnfrsf1b, Trpm6, Tsc1, Utrn	mmu-let-7a, mmu-let-7b, mmu-let-7c, mmu-let-7e, mmu-let-7f, mmu-miR-98
GO:0045202	synapse	Bsn, Cask, Dlgap1, Dlgap4, Dmd, Gabra6, Gipc1, Gpr156, Grid2ip, Grik2, Grin2b, Homer2, Insr, Mgll, P2rx1, Psd3, Sema4c, Sema4f, Snap23, Syt1, Syt11, Syt2	mmu-let-7a, mmu-let-7b, mmu-let-7c, mmu-let-7e, mmu-let-7f, mmu-miR-98
GO:0045211	postsynaptic membrane	Dlgap1, Epha4, Gabra6, Gpr156, Grid2ip, Grik2, Grin2b, Homer2, P2rx1, Psd3, Sema4c, Sema4f	mmu-let-7a, mmu-let-7b, mmu-let-7c, mmu-let-7e, mmu-let-7f, mmu-miR-98
GO:0070195	growth hormone receptor complex	Ghr	mmu-let-7a, mmu-let-7b, mmu-let-7c, mmu-let-7e, mmu-let-7f, mmu-miR-98
GO:0070685	macropinocytic cup	Snx5	mmu-let-7a, mmu-let-7b, mmu-let-7c, mmu-let-7e, mmu-let-7f, mmu-miR-98
GO:0070971	endoplasmic reticulum exit site	Tmed5	mmu-let-7a, mmu-let-7b, mmu-let-7c, mmu-let-7e, mmu-let-7f, mmu-miR-98
GO:0071204	histone pre-mRNA 3'end processing complex	Lsm11, Syncrip	mmu-let-7a, mmu-let-7b, mmu-let-7c, mmu-let-7e, mmu-let-7f, mmu-miR-98

**GO ID (biological process)**	**Go Term (biological process GO Term)**	**Gene Symbol (genes enriched by up-regulated miRNAs)**	**Up-regulated miRNAs**
GO:0000082	G1/S transition of mitotic cell cycle	Acvr1b, Ccnd2, Cdc25a, E2f5, Phf8	mmu-let-7a, mmu-let-7b, mmu-let-7c, mmu-let-7e, mmu-let-7f, mmu-miR-98
GO:0000083	regulation of transcription involved in G1/S phase of mitotic cell cycle	Bach1, E2f6, Rb1	mmu-let-7a, mmu-let-7b, mmu-let-7c, mmu-let-7e, mmu-let-7f, mmu-miR-98
GO:0000084	S phase of mitotic cell cycle	Dcun1d3, Ezh2, Lsm11, Rb1	mmu-let-7a, mmu-let-7b, mmu-let-7c, mmu-let-7e, mmu-let-7f, mmu-miR-98
GO:0000117	regulation of transcription involved in G2/M-phase of mitotic cell cycle	Bach1	mmu-let-7a, mmu-let-7b, mmu-let-7c, mmu-let-7e, mmu-let-7f, mmu-miR-98
GO:0000122	negative regulation of transcription from RNA polymerase II promoter	Bach1, Cbx2, Cry2, Edn1, Esr2, Foxp2, Gabpa, Hand1, Hic1, Hmga2, Lcor, Mdfi, Nkap, Nr6a1, Ppara, Prdm1, Prrx1, Rb1, Sall4, Skil, Srebf2, Taf9b, Wnt1, Wnt9a, Wnt9b, Zbtb10, Zfp354a	mmu-let-7a, mmu-let-7b, mmu-let-7c, mmu-let-7e, mmu-let-7f, mmu-miR-98
GO:0000188	inactivation of MAPK activity	Dusp1, Dusp16, Dusp4, Spred3	mmu-let-7a, mmu-let-7b, mmu-let-7c, mmu-let-7e, mmu-let-7f, mmu-miR-98
GO:0000278	mitotic cell cycle	Acvr1b, Ahctf1, Arpp19, Bach1, Bcat1, Bcl2l1, Ccnd1, Ccnd2, Ccny, Cdc25a, Clasp2, Dcun1d3, E2f5, E2f6, Edn1, Ezh2, Hmga2, Insr, Klhl9, Lmln, Lsm11, Nek3, Nme6, Pdgfb, Phf8, Pogz, Rb1, Usp47, Wnt9a	mmu-let-7a, mmu-let-7b, mmu-let-7c, mmu-let-7e, mmu-let-7f, mmu-miR-98

GO:0000302	response to reactive oxygen species	Casp3, Ercc6, Il6, Mapk8, Ppargc1b, Pxdn	mmu-let-7a, mmu-let-7b, mmu-let-7c, mmu-let-7e, mmu-let-7f, mmu-miR-98
GO:0000902	cell morphogenesis	Bin3, Brwd1, C230081A13Rik, Cap1, Cap2, Dlc1, Dmd, Epha4, Esrrb, Gas7, Hmga2, Igf1r, Il6, Iqcb1, Limd1, Lmx1a, Nefm, Ngf, Ntn1, Pak1, Pla2g3, Rb1, Rbpj, Rufy3, Sema4f, Skil, Spnb4, Tgfbr1, Tgfbr3, Tmem231, Tnik, Ulk2, Unc5a, Wnt9b	mmu-let-7a, mmu-let-7b, mmu-let-7c, mmu-let-7e, mmu-let-7f, mmu-miR-98
GO:0000910	cytokinesis	Ahctf1, Bcl2l1, Bin3, Calm1, Igf1r, Klhl9, Rab11fip4, Ralb, Zfyve26	mmu-let-7a, mmu-let-7b, mmu-let-7c, mmu-let-7e, mmu-let-7f, mmu-miR-98
GO:0001655	urogenital system development	Esr2, Frs2, Gcnt4, Igf1r, Lgr4, Pbx1, Pdgfb, Prlr, Rdh10, Tet2, Tgfbr1, Tsc1, Wnt9b, Zbtb16	mmu-let-7a, mmu-let-7b, mmu-let-7c, mmu-let-7e, mmu-let-7f, mmu-miR-98
GO:0001662	behavioral fear response	Esr2, Grik2, Grin2b	mmu-let-7a, mmu-let-7b, mmu-let-7c, mmu-let-7e, mmu-let-7f, mmu-miR-98
GO:0001701	in utero embryonic development	Acvr1b, Apba1, Bcl2l1, Edn1, Etnk2, Gabpa, Grin2b, Mll2, Rcn1, Rdh10, Slc30a1, Wnt9b	mmu-let-7a, mmu-let-7b, mmu-let-7c, mmu-let-7e, mmu-let-7f, mmu-miR-98
GO:0001706	endoderm formation	Dusp1, Dusp4, Hmga2	mmu-let-7a, mmu-let-7b, mmu-let-7c, mmu-let-7e, mmu-let-7f, mmu-miR-98
GO:0001823	mesonephros development	Wnt9b, Zbtb16	mmu-let-7a, mmu-let-7b, mmu-let-7c, mmu-let-7e, mmu-let-7f, mmu-miR-98
GO:0001824	blastocyst development	Acvr1c, Sall4, Tgfbr1, Tgfbr3	mmu-let-7a, mmu-let-7b, mmu-let-7c, mmu-let-7e, mmu-let-7f, mmu-miR-98

GO:0001825	blastocyst formation	Esrrb, Skil, Tead4	mmu-let-7a, mmu-let-7b, mmu-let-7c, mmu-let-7e, mmu-let-7f, mmu-miR-98
GO:0001830	trophectodermal cell fate commitment	Tead4	mmu-let-7a, mmu-let-7b, mmu-let-7c, mmu-let-7e, mmu-let-7f, mmu-miR-98
GO:0001834	trophectodermal cell proliferation	Acvr1c, Esrrb	mmu-let-7a, mmu-let-7b, mmu-let-7c, mmu-let-7e, mmu-let-7f, mmu-miR-98
GO:0001892	embryonic placenta development	Esrrb, Hand1, Il10, Mdfi, Pdgfb, Prdm1	mmu-let-7a, mmu-let-7b, mmu-let-7c, mmu-let-7e, mmu-let-7f, mmu-miR-98
GO:0001928	regulation of exocyst assembly	Ralb	mmu-let-7a, mmu-let-7b, mmu-let-7c, mmu-let-7e, mmu-let-7f, mmu-miR-98
GO:0001932	regulation of protein phosphorylation	Ezh2, Fnip1, Fnip2, Socs1, Wnt9b	mmu-let-7a, mmu-let-7b, mmu-let-7c, mmu-let-7e, mmu-let-7f, mmu-miR-98
GO:0001934	positive regulation of protein phosphorylation	Acvr2a, Braf, Ccnd1, Ccnd2, Ghr, Il13, Il6, Insr, Pak1, Rictor, Senp2, Taok1, Wnt1	mmu-let-7a, mmu-let-7b, mmu-let-7c, mmu-let-7e, mmu-let-7f, mmu-miR-98
GO:0001952	regulation of cell-matrix adhesion	Epha3, Ppm1f, Tsc1, Utrn	mmu-let-7a, mmu-let-7b, mmu-let-7c, mmu-let-7e, mmu-let-7f, mmu-miR-98
GO:0001953	negative regulation of cell-matrix adhesion	Acer2, Cask, Thbs1	mmu-let-7a, mmu-let-7b, mmu-let-7c, mmu-let-7e, mmu-let-7f, mmu-miR-98
GO:0002009	morphogenesis of an epithelium	Esr2, Fras1, Frem2, Igf1r, Tbx5	mmu-let-7a, mmu-let-7b, mmu-let-7c, mmu-let-7e, mmu-let-7f, mmu-miR-98
GO:0002024	diet induced thermogenesis	Adrb2, Adrb3	mmu-let-7a, mmu-let-7b, mmu-let-7c, mmu-let-7e, mmu-let-7f, mmu-miR-98

GO:0002025	vasodilation by norepinephrine-epinephrine involved in regulation of systemic arterial blood pressure	Adrb2, Adrb3	mmu-let-7a, mmu-let-7b, mmu-let-7c, mmu-let-7e, mmu-let-7f, mmu-miR-98
GO:0002070	epithelial cell maturation	Agpat6, Esr2	mmu-let-7a, mmu-let-7b, mmu-let-7c, mmu-let-7e, mmu-let-7f, mmu-miR-98
GO:0002071	glandular epithelial cell maturation	Agpat6	mmu-let-7a, mmu-let-7b, mmu-let-7c, mmu-let-7e, mmu-let-7f, mmu-miR-98
GO:0002294	CD4-positive, alpha-beta T cell differentiation involved in immune response	Ccr7, Il6, Rorc	mmu-let-7a, mmu-let-7b, mmu-let-7c, mmu-let-7e, mmu-let-7f, mmu-miR-98
GO:0002384	hepatic immune response	Il6	mmu-let-7a, mmu-let-7b, mmu-let-7c, mmu-let-7e, mmu-let-7f, mmu-miR-98
GO:0002577	regulation of antigen processing and presentation	Ccr7, Thbs1	mmu-let-7a, mmu-let-7b, mmu-let-7c, mmu-let-7e, mmu-let-7f, mmu-miR-98
GO:0002581	negative regulation of antigen processing and presentation of peptide or polysaccharide antigen via MHC class II	Thbs1	mmu-let-7a, mmu-let-7b, mmu-let-7c, mmu-let-7e, mmu-let-7f, mmu-miR-98
GO:0002605	negative regulation of dendritic cell antigen processing and presentation	Thbs1	mmu-let-7a, mmu-let-7b, mmu-let-7c, mmu-let-7e, mmu-let-7f, mmu-miR-98

GO:0002632	negative regulation of granuloma formation	Tnfaip3	mmu-let-7a, mmu-let-7b, mmu-let-7c, mmu-let-7e, mmu-let-7f, mmu-miR-98
GO:0002644	negative regulation of tolerance induction	Cd86	mmu-let-7a, mmu-let-7b, mmu-let-7c, mmu-let-7e, mmu-let-7f, mmu-miR-98
GO:0002668	negative regulation of T cell anergy	Cd86	mmu-let-7a, mmu-let-7b, mmu-let-7c, mmu-let-7e, mmu-let-7f, mmu-miR-98
GO:0002676	regulation of chronic inflammatory response	Il10, Tnfaip3	mmu-let-7a, mmu-let-7b, mmu-let-7c, mmu-let-7e, mmu-let-7f, mmu-miR-98
GO:0002875	negative regulation of chronic inflammatory response to antigenic stimulus	Il10	mmu-let-7a, mmu-let-7b, mmu-let-7c, mmu-let-7e, mmu-let-7f, mmu-miR-98
GO:0003001	generation of a signal involved in cell-cell signaling	Acvr1c, Acvr2a, Apba1, Edn1, Gipc1, Grin2b, Hmga2, Il6, Kcnc3, Kcnc4, Ngf, P2rx1, Rab8b, Rfx6, Slc30a1, Slc6a1, Syt1	mmu-let-7a, mmu-let-7b, mmu-let-7c, mmu-let-7e, mmu-let-7f, mmu-miR-98
GO:0003002	regionalization	Acvr2a, Chrd, Edn1, Frs2, Mapk8, Mdfi, Pbx1, Pbx2, Senp2, Tcf7l1, Tgfbr1, Wnt1, Wnt9a, Wnt9b, Zbtb16	mmu-let-7a, mmu-let-7b, mmu-let-7c, mmu-let-7e, mmu-let-7f, mmu-miR-98
GO:0003044	regulation of systemic arterial blood pressure mediated by a chemical signal	Adrb2, Adrb3, Edn1, Ndst2	mmu-let-7a, mmu-let-7b, mmu-let-7c, mmu-let-7e, mmu-let-7f, mmu-miR-98
GO:0003059	positive regulation of the force of heart contraction by epinephrine	Adrb2	mmu-let-7a, mmu-let-7b, mmu-let-7c, mmu-let-7e, mmu-let-7f, mmu-miR-98
GO:0003104	positive regulation of glomerular filtration	Pdgfb	mmu-let-7a, mmu-let-7b, mmu-let-7c, mmu-let-7e, mmu-let-7f, mmu-miR-98

GO:0003131	mesodermal-endodermal cell signaling	Hmga2	mmu-let-7a, mmu-let-7b, mmu-let-7c, mmu-let-7e, mmu-let-7f, mmu-miR-98
GO:0003144	embryonic heart tube formation	Hand1	mmu-let-7a, mmu-let-7b, mmu-let-7c, mmu-let-7e, mmu-let-7f, mmu-miR-98
GO:0003207	cardiac chamber formation	Hand1, Tbx5	mmu-let-7a, mmu-let-7b, mmu-let-7c, mmu-let-7e, mmu-let-7f, mmu-miR-98
GO:0003218	cardiac left ventricle formation	Hand1, Tbx5	mmu-let-7a, mmu-let-7b, mmu-let-7c, mmu-let-7e, mmu-let-7f, mmu-miR-98
GO:0003311	pancreatic D cell differentiation	Rfx6	mmu-let-7a, mmu-let-7b, mmu-let-7c, mmu-let-7e, mmu-let-7f, mmu-miR-98
GO:0006066	alcohol metabolic process	Abcg1, Acer2, Agpat6, Arpp19, Cmah, Cyp46a1, Etnk1, Etnk2, Gale, Gcnt4, Gdpd1, Hdlbp, Hk2, Il6, Insr, Mbtps2, Pcx, Pcyt1b, Pgm2l1, Pla2g15, Ppara, Prkaa2, Srebf2	mmu-let-7a, mmu-let-7b, mmu-let-7c, mmu-let-7e, mmu-let-7f, mmu-miR-98
GO:0006107	oxaloacetate metabolic process	Ghr, Pcx	mmu-let-7a, mmu-let-7b, mmu-let-7c, mmu-let-7e, mmu-let-7f, mmu-miR-98
GO:0006109	regulation of carbohydrate metabolic process	Acer2, Arpp19, Il6, Insr, Ppara, Prkaa2	mmu-let-7a, mmu-let-7b, mmu-let-7c, mmu-let-7e, mmu-let-7f, mmu-miR-98
GO:0006139	nucleobase-containing compound metabolic process	Abcb9, Abcc10, Adrb3, Aldh6a1, Arhgap20, Arhgap28, Arhgef15, Ccr7, Cmah, Crk, Ctns, Ddx19a, Dhx57, Dlc1, Dlst, Entpd7, Epha3, Epha4, Gipc1, Gnal, Htr4, Lonrf3, Nkiras2, Nme4, Nme6, Plekhg6, Ralb, Rasl10b, Rgs16, Rictor, Srgap3, Stard13, Tgds, Tsc1, Vav3	mmu-let-7a, mmu-let-7b, mmu-let-7c, mmu-let-7e, mmu-let-7f, mmu-miR-98

GO:0006140	regulation of nucleotide metabolic process	Adrb2, Adrb3, Arhgap20, Arhgap28, Arhgef15, Ccr7, Crk, Dlc1, Edn1, Epha3, Epha4, Gipc1, Gnal, Htr4, Plekhg6, Rgs16, Rictor, Srgap3, Stard13, Tsc1, Vav3	mmu-let-7a, mmu-let-7b, mmu-let-7c, mmu-let-7e, mmu-let-7f, mmu-miR-98
GO:0006163	purine nucleotide metabolic process	Abcb9, Abcc10, Abcg1, Adrb2, Adrb3, Arhgap20, Arhgap28, Arhgef15, Ccr7, Chd4, Crk, Ctns, Ddx19a, Dhx57, Dlc1, Edn1, Epha3, Epha4, Ercc6, Gipc1, Gnal, Htr4, Lonrf3, Nkiras2, Nme4, Nme6, Plekhg6, Ralb, Rasl10b, Rgs16, Rictor, Srgap3, Stard13, Trank1, Tsc1, Vav3	mmu-let-7a, mmu-let-7b, mmu-let-7c, mmu-let-7e, mmu-let-7f, mmu-miR-98
GO:0006165	nucleoside diphosphate phosphorylation	Nme4, Nme6	mmu-let-7a, mmu-let-7b, mmu-let-7c, mmu-let-7e, mmu-let-7f, mmu-miR-98
GO:0006183	GTP biosynthetic process	Nme4, Nme6	mmu-let-7a, mmu-let-7b, mmu-let-7c, mmu-let-7e, mmu-let-7f, mmu-miR-98
GO:0006184	GTP catabolic process	Arhgap20, Arhgap28, Arhgef15, Ccr7, Crk, Dlc1, Epha3, Epha4, Gipc1, Gnal, Nkiras2, Plekhg6, Ralb, Rasl10b, Rgs16, Rictor, Srgap3, Stard13, Tsc1, Vav3	mmu-let-7a, mmu-let-7b, mmu-let-7c, mmu-let-7e, mmu-let-7f, mmu-miR-98
GO:0006228	UTP biosynthetic process	Nme4, Nme6	mmu-let-7a, mmu-let-7b, mmu-let-7c, mmu-let-7e, mmu-let-7f, mmu-miR-98
GO:0006241	CTP biosynthetic process	Nme4, Nme6	mmu-let-7a, mmu-let-7b, mmu-let-7c, mmu-let-7e, mmu-let-7f, mmu-miR-98
GO:0006284	base-excision repair	Ercc6, Hmga1, Hmga2, Usp47	mmu-let-7a, mmu-let-7b, mmu-let-7c, mmu-let-7e, mmu-let-7f, mmu-miR-98
GO:0006357	regulation of transcription from RNA polymerase II promoter	Brwd1, Ezh2, Ikbkap, Lcorl, Med8, Onecut3, Rorc, Sub1	mmu-let-7a, mmu-let-7b, mmu-let-7c, mmu-let-7e, mmu-let-7f, mmu-miR-98

GO:0006405	RNA export from nucleus	Thoc1, Thoc2, Tsc1, Zc3h3	mmu-let-7a, mmu-let-7b, mmu-let-7c, mmu-let-7e, mmu-let-7f, mmu-miR-98
GO:0006409	tRNA export from nucleus	Xpot	mmu-let-7a, mmu-let-7b, mmu-let-7c, mmu-let-7e, mmu-let-7f, mmu-miR-98
GO:0006417	regulation of translation	Dnajc1, Eif2c4, Eif4g2, Igf2bp2, Igf2bp3, Il6, Lin28a, Tarbp2, Thbs1, Tia1, Tsc1	mmu-let-7a, mmu-let-7b, mmu-let-7c, mmu-let-7e, mmu-let-7f, mmu-miR-98
GO:0006468	protein phosphorylation	Acvr1c, Cask, Epha3, Frk, Ikbkap, Ikbke, Limk2, Map4k3, Map4k4, Mapk11, Mapk6, Nek3, Obscn, Riok3, Rorc, Scyl3, Speg, Stk40, Styk1, Tgfbr3, Trib2, Trpm6	mmu-let-7a, mmu-let-7b, mmu-let-7c, mmu-let-7e, mmu-let-7f, mmu-miR-98
GO:0006470	protein dephosphorylation	Dlc1, Dusp1, Dusp16, Dusp22, Dusp4, Nceh1, Ppp2r2a, Ptprd, Ptpru	mmu-let-7a, mmu-let-7b, mmu-let-7c, mmu-let-7e, mmu-let-7f, mmu-miR-98
GO:0006486	protein glycosylation	Acer2, B3galt1, B3gnt7, Galnt1, Galnt2, Gxylt1, St6gal1, St8sia1, Uggt1	mmu-let-7a, mmu-let-7b, mmu-let-7c, mmu-let-7e, mmu-let-7f, mmu-miR-98
GO:0006508	proteolysis	Adamts12, Adamts14, Adamts15, Adamts5, Adamts8, Atg4b, Casp3, Cpa4, Cpd, Ddi2, Dpp3, Il10, Klkb1, Lmln, Lonrf3, Masp1, Mbtps2, Npepl1, Pappa, Prss22, Senp2, Senp5, Tmprss11f, Tmprss2, Tnfaip3, Tpp1, Trhde, Usp32, Mmp11	mmu-let-7a, mmu-let-7b, mmu-let-7c, mmu-let-7e, mmu-let-7f, mmu-miR-98
GO:0006511	ubiquitin-dependent protein catabolic process	Cdc34, Dnajb9, Edem1, Edem3, Fbxl12, Fbxl14, Gipc1, Map3k1, Rnf20, Trib1, Trib2, Uhrf2, Usp12, Usp24, Usp38, Usp47, Wnt1, Yod1	mmu-let-7a, mmu-let-7b, mmu-let-7c, mmu-let-7e, mmu-let-7f, mmu-miR-98
GO:0006574	valine catabolic process	Aldh6a1	mmu-let-7a, mmu-let-7b, mmu-let-7c, mmu-let-7e, mmu-let-7f, mmu-miR-98
GO:0006580	ethanolamine metabolic process	Etnk1, Etnk2	mmu-let-7a, mmu-let-7b, mmu-let-7c, mmu-let-7e, mmu-let-7f, mmu-miR-98

GO:0006600	creatine metabolic process	Gatm, Ghr	mmu-let-7a, mmu-let-7b, mmu-let-7c, mmu-let-7e, mmu-let-7f, mmu-miR-98
GO:0006631	fatty acid metabolic process	Acot11, Adipor2, Agpat6, Edn1, Elovl4, Ghr, Lipt2, Mgll, Pla2g15, Ppara, Ppargc1a, Prkaa2, Prkab2, Scd2	mmu-let-7a, mmu-let-7b, mmu-let-7c, mmu-let-7e, mmu-let-7f, mmu-miR-98
GO:0006646	phosphatidylethanolamine biosynthetic process	Etnk1, Etnk2	mmu-let-7a, mmu-let-7b, mmu-let-7c, mmu-let-7e, mmu-let-7f, mmu-miR-98
GO:0006814	sodium ion transport	Adrb2, Slc10a6, Slc24a6, Slc38a9, Slc4a4, Slc5a9, Slc8a2	mmu-let-7a, mmu-let-7b, mmu-let-7c, mmu-let-7e, mmu-let-7f, mmu-miR-98
GO:0006836	neurotransmitter transport	Kcnc3, Kcnc4, Nat8l, Ngf, Slc30a1, Slc6a1, Stx3, Syt1	mmu-let-7a, mmu-let-7b, mmu-let-7c, mmu-let-7e, mmu-let-7f, mmu-miR-98
GO:0006865	amino acid transport	Apba1, Ctns, Slc38a9, Slc5a6, Slc6a1, Slc7a14, Xk	mmu-let-7a, mmu-let-7b, mmu-let-7c, mmu-let-7e, mmu-let-7f, mmu-miR-98
GO:0006885	regulation of pH	Edn1, Fasl, Slc26a9, Slc4a4	mmu-let-7a, mmu-let-7b, mmu-let-7c, mmu-let-7e, mmu-let-7f, mmu-miR-98
GO:0006906	vesicle fusion	Eea1, Pldn, Vav3	mmu-let-7a, mmu-let-7b, mmu-let-7c, mmu-let-7e, mmu-let-7f, mmu-miR-98
GO:0006915	apoptotic process	Bcap29, Dpf2, E2f2, Ntn1, Pacs2, Peg10, Ppm1f, Ppp2r1b, Ralb, Slk, Taok1, Tia1, Unc5a	mmu-let-7a, mmu-let-7b, mmu-let-7c, mmu-let-7e, mmu-let-7f, mmu-miR-98
GO:0006917	induction of apoptosis	Abcb9, Acvr1b, Aen, Casp3, Ddx19a, Dlc1, Ercc6, Fasl, Fnip2, Hic1, Hip1, Ikbke, Mapk8, Plagl2, Rnf7, Tbx5, Tgfbr1, Thbs1	mmu-let-7a, mmu-let-7b, mmu-let-7c, mmu-let-7e, mmu-let-7f, mmu-miR-98

GO:0006919	activation of cysteine-type endopeptidase activity involved in apoptotic process	Dlc1, Fasl, Hip1, P2rx1, Rnf7	mmu-let-7a, mmu-let-7b, mmu-let-7c, mmu-let-7e, mmu-let-7f, mmu-miR-98
GO:0006986	response to unfolded protein	Ccnd1, Edem1, Edem3, Ero1l, Ubxn4, Yod1	mmu-let-7a, mmu-let-7b, mmu-let-7c, mmu-let-7e, mmu-let-7f, mmu-miR-98
GO:0007049	cell cycle	Bin3, Calm1, Cdc34, Cep110, Cep120, Cep135, Dusp1, E2f2, Mapk6, Nras, Rab11fip4, Ralb, Senp5, Stard13, Suv39h2, Tet2, Uhrf2, Wasl, Zfyve26, Ercc4	mmu-let-7a, mmu-let-7b, mmu-let-7c, mmu-let-7e, mmu-let-7f, mmu-miR-98
GO:0007050	cell cycle arrest	Bcat1, Bcl2l1, Ccnd1, Ezh2, Hmga2, Rb1, Rbm38, Skil, Taok1, Thbs1, Wnt9a	mmu-let-7a, mmu-let-7b, mmu-let-7c, mmu-let-7e, mmu-let-7f, mmu-miR-98
GO:0007064	mitotic sister chromatid cohesion	Pogz, Rb1	mmu-let-7a, mmu-let-7b, mmu-let-7c, mmu-let-7e, mmu-let-7f, mmu-miR-98
GO:0007162	negative regulation of cell adhesion	Acer2, Cask, Myo1f, Thbs1, Tnfaip3, Wnt1	mmu-let-7a, mmu-let-7b, mmu-let-7c, mmu-let-7e, mmu-let-7f, mmu-miR-98
GO:0007167	enzyme linked receptor protein signaling pathway	Acvr1b, Acvr1c, Acvr2a, Chrd, Col1a2, Col3a1, Dusp22, Gdf6, Gipc1, Map3k1, Nlk, Peg10, Skil, Tgfbr1, Tgfbr3, Trim33	mmu-let-7a, mmu-let-7b, mmu-let-7c, mmu-let-7e, mmu-let-7f, mmu-miR-98
GO:0007169	transmembrane receptor protein tyrosine kinase signaling pathway	AI464131, Esr2, Fasl, Ghr, Igf1r, Insr, Ngf, Nrtn, Prlr, Smarcc1, Socs1, Socs7, Wnt1	mmu-let-7a, mmu-let-7b, mmu-let-7c, mmu-let-7e, mmu-let-7f, mmu-miR-98
GO:0007179	transforming growth factor beta receptor signaling pathway	Col1a2, Col3a1, Dusp22, Map3k1, Nlk, Pdgfb, Tgfbr1, Trim33	mmu-let-7a, mmu-let-7b, mmu-let-7c, mmu-let-7e, mmu-let-7f, mmu-miR-98

GO:0007181	transforming growth factor beta receptor complex assembly	Acvr1c, Tgfbr3	mmu-let-7a, mmu-let-7b, mmu-let-7c, mmu-let-7e, mmu-let-7f, mmu-miR-98
GO:0007189	activation of adenylate cyclase activity by G-protein signaling [illegible]	Adrb2, Adrb3, Gnal, Htr4	mmu-let-7a, mmu-let-7b, mmu-let-7c, mmu-let-7e, mmu-let-7f, mmu-miR-98
GO:0007216	G-protein coupled glutamate receptor signaling pathway	Grid2ip, Homer2	mmu-let-7a, mmu-let-7b, mmu-let-7c, mmu-let-7e, mmu-let-7f, mmu-miR-98
GO:0007256	activation of JNKK activity	Ercc6, Map3k1, Tnik	mmu-let-7a, mmu-let-7b, mmu-let-7c, mmu-let-7e, mmu-let-7f, mmu-miR-98
GO:0007257	activation of JUN kinase activity	Ccr7, Ercc6, Map3k1, Mdfi	mmu-let-7a, mmu-let-7b, mmu-let-7c, mmu-let-7e, mmu-let-7f, mmu-miR-98
GO:0007270	neuron-neuron synaptic transmission	Adrb2, Gabra6, Grik2, Nat8l, P2rx1, Slc6a1, Tmod2	mmu-let-7a, mmu-let-7b, mmu-let-7c, mmu-let-7e, mmu-let-7f, mmu-miR-98
GO:0007281	germ cell development	Bcl2l1, Fndc3a, Hook1, Lin28a, Mll2, Prdm1, Strbp, Zbtb16	mmu-let-7a, mmu-let-7b, mmu-let-7c, mmu-let-7e, mmu-let-7f, mmu-miR-98
GO:0007350	blastoderm segmentation	Frs2, Tcf7l1	mmu-let-7a, mmu-let-7b, mmu-let-7c, mmu-let-7e, mmu-let-7f, mmu-miR-98
GO:0007369	gastrulation	Acvr2a, Chrd, Dusp1, Dusp4, Frs2, Hand1, Hmga2, Tnfaip3	mmu-let-7a, mmu-let-7b, mmu-let-7c, mmu-let-7e, mmu-let-7f, mmu-miR-98
GO:0007417	central nervous system development	Cep120, Chrd, Ctns, Dlc1, Epha4, Esr2, Ezh2, Foxp2, Frs2, Hmga2, Igf1r, Lingo1, Lmx1a, Mycn, Pbx3, Rbpj, Sall3, Sema4c, Slc4a7, Tcf7l1, Tsc1, Wnt1, Zbtb16	mmu-let-7a, mmu-let-7b, mmu-let-7c, mmu-let-7e, mmu-let-7f, mmu-miR-98

GO:0007431	salivary gland development	Eda, Il6, Pdgfb, Plxnd1	mmu-let-7a, mmu-let-7b, mmu-let-7c, mmu-let-7e, mmu-let-7f, mmu-miR-98
GO:0007517	muscle organ development	Acta1, Adrb2, AI464131, Col4a1, Col4a5, Dmd, Foxp2, Hand1, Il6, Pak1, Rbm38, Scn8a, Skil, Tbx5, Tgfbr3, Utrn, Wnt1	mmu-let-7a, mmu-let-7b, mmu-let-7c, mmu-let-7e, mmu-let-7f, mmu-miR-98
GO:0007519	skeletal muscle tissue development	Acta1, Adrb2, AI464131, Col4a1, Col4a5, Dmd, Foxp2, Pak1, Rbm38, Skil, Utrn, Wnt1	mmu-let-7a, mmu-let-7b, mmu-let-7c, mmu-let-7e, mmu-let-7f, mmu-miR-98
GO:0007528	neuromuscular junction development	Col4a1, Col4a5, Pak1, Utrn	mmu-let-7a, mmu-let-7b, mmu-let-7c, mmu-let-7e, mmu-let-7f, mmu-miR-98
GO:0007548	sex differentiation	Acvr2a, Bcl2l1, Cbx2, Ccnd1, Esr2, Fndc3a, Hmga2, Lgr4, Pbx1, Pcyt1b, Rdh10, Wnt9b	mmu-let-7a, mmu-let-7b, mmu-let-7c, mmu-let-7e, mmu-let-7f, mmu-miR-98
GO:0007576	nucleolar fragmentation	Zfp354a	mmu-let-7a, mmu-let-7b, mmu-let-7c, mmu-let-7e, mmu-let-7f, mmu-miR-98
GO:0007595	lactation	Agpat6, Ccnd1, Prlr	mmu-let-7a, mmu-let-7b, mmu-let-7c, mmu-let-7e, mmu-let-7f, mmu-miR-98
GO:0007638	mechanosensory behavior	Foxp2, Strbp	mmu-let-7a, mmu-let-7b, mmu-let-7c, mmu-let-7e, mmu-let-7f, mmu-miR-98
GO:0008219	cell death	Bcap29, Dpf2, E2f2, Hk2, Ntn1, Olr1, P2rx1, Pacs2, Peg10, Ppm1f, Ppp2r1b, Ralb, Rb1, Slc4a7, Slk, Taok1, Tia1, Tnfrsf1b, Unc5a	mmu-let-7a, mmu-let-7b, mmu-let-7c, mmu-let-7e, mmu-let-7f, mmu-miR-98
GO:0008284	positive regulation of cell proliferation	Acer2, Adrb2, Bcl2l1, Ccr7, Cd276, Cd86, Edn1, Fasl, Fgf4, Fgf5, Htr4, Insr, Mll2, Mycn, Ntn1, Osmr, Pbx1, Prrx1, Rbpj, Rrm2, St8sia1, Tbx5, Tgfbr1, Vav3, Wnt1	mmu-let-7a, mmu-let-7b, mmu-let-7c, mmu-let-7e, mmu-let-7f, mmu-miR-98

GO:0008285	negative regulation of cell proliferation	Cask, Casp3, Cd276, Dlc1, Esr2, Hmga1, Il10, Il6, Pak1, Prdm1, Ptpru, Rb1, Rbm38, Rbpj, Tbx5, Tgfbr3, Thbs1, Tnfaip3, Trib1, Tsc1, Wnt9a, Zbtb16	mmu-let-7a, mmu-let-7b, mmu-let-7c, mmu-let-7e, mmu-let-7f, mmu-miR-98
GO:0008356	asymmetric cell division	Wnt9b, Zbtb16	mmu-let-7a, mmu-let-7b, mmu-let-7c, mmu-let-7e, mmu-let-7f, mmu-miR-98
GO:0008543	fibroblast growth factor receptor signaling pathway	Fgf4, Fgf5, Frs2, Pdgfb, Thbs1	mmu-let-7a, mmu-let-7b, mmu-let-7c, mmu-let-7e, mmu-let-7f, mmu-miR-98
GO:0008595	anterior/posterior axis specification, embryo	Frs2, Tcf7l1	mmu-let-7a, mmu-let-7b, mmu-let-7c, mmu-let-7e, mmu-let-7f, mmu-miR-98
GO:0008610	lipid biosynthetic process	Abcg1, Acer2, Cercam, Dkk3, Edn1, Elovl4, Igf1r, Lipt2, Mgll, P2rx1, Pbx1, Prkaa2, Prkab2, Scd2, St8sia1, Ugcg	mmu-let-7a, mmu-let-7b, mmu-let-7c, mmu-let-7e, mmu-let-7f, mmu-miR-98
GO:0008625	induction of apoptosis via death domain receptors	Casp3, Fasl	mmu-let-7a, mmu-let-7b, mmu-let-7c, mmu-let-7e, mmu-let-7f, mmu-miR-98
GO:0008630	DNA damage response, signal transduction resulting in induction of apoptosis	Aen, Ercc6, Fnip2, Hic1, Ikbke	mmu-let-7a, mmu-let-7b, mmu-let-7c, mmu-let-7e, mmu-let-7f, mmu-miR-98
GO:0008631	induction of apoptosis by oxidative stress	Casp3, Rnf7	mmu-let-7a, mmu-let-7b, mmu-let-7c, mmu-let-7e, mmu-let-7f, mmu-miR-98
GO:0008637	apoptotic mitochondrial changes	Bcl2l1, Casp3, Hk2, Map3k1, Rnf7	mmu-let-7a, mmu-let-7b, mmu-let-7c, mmu-let-7e, mmu-let-7f, mmu-miR-98
GO:0008643	carbohydrate transport	Adipor2, Edn1, Hk2, Insr, Slc2a12, Slc35c1, Slc35d2	mmu-let-7a, mmu-let-7b, mmu-let-7c, mmu-let-7e, mmu-let-7f, mmu-miR-98

GO:0009081	branched chain family amino acid metabolic process	Aldh6a1, Bcat1, Ghr	mmu-let-7a, mmu-let-7b, mmu-let-7c, mmu-let-7e, mmu-let-7f, mmu-miR-98
GO:0009100	glycoprotein metabolic process	Abcg1, Acer2, B3galt1, B3gnt7, Ccr7, Edem3, Galnt1, Galnt2, Gxylt1, Insr, Ndst2, St6gal1, St8sia1, Uggt1	mmu-let-7a, mmu-let-7b, mmu-let-7c, mmu-let-7e, mmu-let-7f, mmu-miR-98
GO:0009141	nucleoside triphosphate metabolic process	Abcb9, Abcc10, Abcg1, Arhgap20, Arhgap28, Arhgef15, Ccr7, Chd4, Crk, Ctns, Ddx19a, Dhx57, Dlc1, Entpd7, Epha3, Epha4, Ercc6, Gipc1, Gnal, Lonrf3, Nkiras2, Nme4, Nme6, Plekhg6, Ralb, Rasl10b, Rgs16, Rictor, Srgap3, Stard13, Trank1, Tsc1, Vav3	mmu-let-7a, mmu-let-7b, mmu-let-7c, mmu-let-7e, mmu-let-7f, mmu-miR-98
GO:0009146	purine nucleoside triphosphate catabolic process	Abcb9, Abcc10, Abcg1, Arhgap20, Arhgap28, Arhgef15, Ccr7, Chd4, Crk, Ddx19a, Dhx57, Dlc1, Epha3, Epha4, Ercc6, Gipc1, Gnal, Lonrf3, Nkiras2, Plekhg6, Ralb, Rasl10b, Rgs16, Rictor, Srgap3, Stard13, Trank1, Tsc1, Vav3	mmu-let-7a, mmu-let-7b, mmu-let-7c, mmu-let-7e, mmu-let-7f, mmu-miR-98
GO:0009148	pyrimidine nucleoside triphosphate biosynthetic process	Nme4, Nme6	mmu-let-7a, mmu-let-7b, mmu-let-7c, mmu-let-7e, mmu-let-7f, mmu-miR-98
GO:0009154	purine ribonucleotide catabolic process	Abcb9, Abcc10, Abcg1, Arhgap20, Arhgap28, Arhgef15, Ccr7, Chd4, Crk, Ddx19a, Dhx57, Dlc1, Epha3, Epha4, Ercc6, Gipc1, Gnal, Lonrf3, Nkiras2, Plekhg6, Ralb, Rasl10b, Rgs16, Rictor, Srgap3, Stard13, Trank1, Tsc1, Vav3	mmu-let-7a, mmu-let-7b, mmu-let-7c, mmu-let-7e, mmu-let-7f, mmu-miR-98
GO:0009166	nucleotide catabolic process	Abcb9, Abcc10, Abcg1, Arhgap20, Arhgap28, Arhgef15, Ccr7, Chd4, Crk, Ddx19a, Dhx57, Dlc1, Entpd7, Epha3, Epha4, Ercc6, Gipc1, Gnal, Lonrf3, Nkiras2, Plekhg6, Ralb, Rasl10b, Rgs16, Rictor, Srgap3, Stard13, Trank1, Tsc1, Vav3	mmu-let-7a, mmu-let-7b, mmu-let-7c, mmu-let-7e, mmu-let-7f, mmu-miR-98

GO:0009203	ribonucleoside triphosphate catabolic process	Abcb9, Abcc10, Abcg1, Arhgap20, Arhgap28, Arhgef15, Ccr7, Chd4, Crk, Ddx19a, Dhx57, Dlc1, Entpd7, Epha3, Epha4, Ercc6, Gipc1, Gnal, Lonrf3, Nkiras2, Plekhg6, Ralb, Rasl10b, Rgs16, Rictor, Srgap3, Stard13, Trank1, Tsc1, Vav3	mmu-let-7a, mmu-let-7b, mmu-let-7c, mmu-let-7e, mmu-let-7f, mmu-miR-98
GO:0009205	purine ribonucleoside triphosphate metabolic process	Abcb9, Abcc10, Abcg1, Arhgap20, Arhgap28, Arhgef15, Ccr7, Chd4, Crk, Ctns, Ddx19a, Dhx57, Dlc1, Epha3, Epha4, Ercc6, Gipc1, Gnal, Lonrf3, Nkiras2, Nme4, Nme6, Plekhg6, Ralb, Rasl10b, Rgs16, Rictor, Srgap3, Stard13, Trank1, Tsc1, Vav3	mmu-let-7a, mmu-let-7b, mmu-let-7c, mmu-let-7e, mmu-let-7f, mmu-miR-98
GO:0009208	pyrimidine ribonucleoside triphosphate metabolic process	Nme4, Nme6	mmu-let-7a, mmu-let-7b, mmu-let-7c, mmu-let-7e, mmu-let-7f, mmu-miR-98
GO:0009259	ribonucleotide metabolic process	Abcb9, Abcc10, Abcg1, Arhgap20, Arhgap28, Arhgef15, Ccr7, Chd4, Crk, Ctns, Ddx19a, Dhx57, Dlc1, Entpd7, Epha3, Epha4, Ercc6, Gipc1, Gnal, Lonrf3, Nkiras2, Nme4, Nme6, Plekhg6, Ralb, Rasl10b, Rgs16, Rictor, Srgap3, Stard13, Trank1, Tsc1, Vav3	mmu-let-7a, mmu-let-7b, mmu-let-7c, mmu-let-7e, mmu-let-7f, mmu-miR-98
GO:0009411	response to UV	Casp3, Ccnd1, Dcun1d3, Ercc6, Mapk8, Ercc4	mmu-let-7a, mmu-let-7b, mmu-let-7c, mmu-let-7e, mmu-let-7f, mmu-miR-98
GO:0009628	response to abiotic stimulus	Adrb2, Adrb3, Aen, Bcat1, Bcl2l1, Casp3, Ccnd1, Cdc25a, Clpb, Col3a1, Dcun1d3, Ercc6, Foxp2, Grin2b, Habp4, Hmga2, Kcnk18, Map3k1, Mapk8, St8sia1, Strbp, Tsc22d2, Usp47, Ercc4	mmu-let-7a, mmu-let-7b, mmu-let-7c, mmu-let-7e, mmu-let-7f, mmu-miR-98
GO:0009636	response to toxin	Ercc6, Scn8a, Slc30a1, Slc6a1, Trpm6	mmu-let-7a, mmu-let-7b, mmu-let-7c, mmu-let-7e, mmu-let-7f, mmu-miR-98
GO:0009725	response to hormone stimulus	Acvr1c, Adipor2, Braf, Ccr7, Esr2, Gatm, Ghr, Il10, Il6, Insr, Mll2, Nr6a1, Pak1, Pappa, Ppara, Rorc, Smarcc1, Socs1, Socs7, Srebf2, Tgfbr1, Tgfbr3, Tsc1	mmu-let-7a, mmu-let-7b, mmu-let-7c, mmu-let-7e, mmu-let-7f, mmu-miR-98

GO:0009755	hormone-mediated signaling pathway	Adipor2, Esr2, Ghr, Nr6a1, Ppara, Rorc	mmu-let-7a, mmu-let-7b, mmu-let-7c, mmu-let-7e, mmu-let-7f, mmu-miR-98
GO:0009792	embryo development ending in birth or egg hatching	Acvr1b, Acvr1c, Acvr2a, Apba1, Bcl2l1, Dlc1, Edn1, Esrrb, Etnk2, Gabpa, Grin2b, Hand1, Hoxd1, Il10, Mdfi, Mll2, Mycn, Pbx1, Pdgfb, Prdm1, Prrx1, Rcn1, Rdh10, Sall4, Sema4c, Skil, Slc30a1, Tead4, Tgfbr1, Tgfbr3, Tsc1, Wnt1, Wnt9a, Wnt9b	mmu-let-7a, mmu-let-7b, mmu-let-7c, mmu-let-7e, mmu-let-7f, mmu-miR-98
GO:0009798	axis specification	Frs2, Mdfi, Senp2, Tcf7l1, Wnt1, Wnt9a, Wnt9b	mmu-let-7a, mmu-let-7b, mmu-let-7c, mmu-let-7e, mmu-let-7f, mmu-miR-98
GO:0009896	positive regulation of catabolic process	Atg4b, Egln2, Insr, Prkaa2, Tnfaip3, Trib1, Trib2	mmu-let-7a, mmu-let-7b, mmu-let-7c, mmu-let-7e, mmu-let-7f, mmu-miR-98
GO:0009968	negative regulation of signal transduction	Adrb2, Bcl2l1, Ccnd1, Cdc34, Dkk3, Dlc1, Dusp1, Dusp16, Dusp4, Esr2, Ezh2, Hic1, Hmga2, Igf1r, Mdfi, Nlk, Nlrc5, Nphp3, Peg10, Prkaa2, Rgs16, Skil, Socs1, Socs7, Spred3, Tgfbr3, Trim33, Tsc1, Wnt1	mmu-let-7a, mmu-let-7b, mmu-let-7c, mmu-let-7e, mmu-let-7f, mmu-miR-98
GO:0009987	cellular process	Btbd9, Cap2, Cdh22, Col14a1, Col15a1, Col4a5, Dlgap4, Dzip1, Fndc3a, Fras1, Frem2, Frmd4b, Kif21b, Nid2, Pappa, Pcdh20, Pla2g3, Rab11fip2, Sdk1, Sema4g, Slc8a2, Snx30, Spnb4, Strbp, Tmed5	mmu-let-7a, mmu-let-7b, mmu-let-7c, mmu-let-7e, mmu-let-7f, mmu-miR-98
GO:0010463	mesenchymal cell proliferation	Fgf4, Foxp2, Mycn, Prrx1	mmu-let-7a, mmu-let-7b, mmu-let-7c, mmu-let-7e, mmu-let-7f, mmu-miR-98
GO:0010468	regulation of gene expression	Apba1, Clp1, Dnajc1, Eif2c4, Eif4g2, Fgf5, Gipc1, Igf2bp2, Igf2bp3, Masp1, Rbm38, Syncrip, Tarbp2, Thbs1, Tia1, Tnfrsf1b, Tob2, Tsc1	mmu-let-7a, mmu-let-7b, mmu-let-7c, mmu-let-7e, mmu-let-7f, mmu-miR-98
GO:0010498	proteasomal protein catabolic process	Cdc34, Dlgap1, Dnajb9, Edem1, Edem3, Gipc1, Trib1, Trib2, Yod1	mmu-let-7a, mmu-let-7b, mmu-let-7c, mmu-let-7e, mmu-let-7f, mmu-miR-98

GO:0010506	regulation of autophagy	Prkaa2, Tnfaip3, Ulk2	mmu-let-7a, mmu-let-7b, mmu-let-7c, mmu-let-7e, mmu-let-7f, mmu-miR-98
GO:0010560	positive regulation of glycoprotein biosynthetic process	Ccr7, Insr	mmu-let-7a, mmu-let-7b, mmu-let-7c, mmu-let-7e, mmu-let-7f, mmu-miR-98
GO:0010578	regulation of adenylate cyclase activity involved in G-protein signaling pathway	Adrb2, Adrb3, Gnal, Htr4	mmu-let-7a, mmu-let-7b, mmu-let-7c, mmu-let-7e, mmu-let-7f, mmu-miR-98
GO:0010587	miRNA catabolic process	Lin28a, Lin28b	mmu-let-7a, mmu-let-7b, mmu-let-7c, mmu-let-7e, mmu-let-7f, mmu-miR-98
GO:0010604	positive regulation of macromolecule metabolic process	Acvr2a, Atg4b, Ccnd1, Ccnd2, Dlc1, Egln2, Ghr, Il13, Pak1, Rictor, Senp2, Taok1, Tnfaip3, Trib1, Trib2	mmu-let-7a, mmu-let-7b, mmu-let-7c, mmu-let-7e, mmu-let-7f, mmu-miR-98
GO:0010613	positive regulation of cardiac muscle hypertrophy	Edn1, Mtpn	mmu-let-7a, mmu-let-7b, mmu-let-7c, mmu-let-7e, mmu-let-7f, mmu-miR-98
GO:0010721	negative regulation of cell development	Epha4, Hmga2, Lin28a, Lingo1, Mycn, Ntn1, Rufy3, Sema4f, Tgfbr3, Ulk2	mmu-let-7a, mmu-let-7b, mmu-let-7c, mmu-let-7e, mmu-let-7f, mmu-miR-98
GO:0010745	negative regulation of macrophage derived foam cell differentiation	Abcg1, Ppara	mmu-let-7a, mmu-let-7b, mmu-let-7c, mmu-let-7e, mmu-let-7f, mmu-miR-98
GO:0010751	negative regulation of nitric oxide mediated signal transduction	Thbs1	mmu-let-7a, mmu-let-7b, mmu-let-7c, mmu-let-7e, mmu-let-7f, mmu-miR-98

GO:0010754	negative regulation of cGMP-mediated signaling	Thbs1	mmu-let-7a, mmu-let-7b, mmu-let-7c, mmu-let-7e, mmu-let-7f, mmu-miR-98
GO:0010769	regulation of cell morphogenesis involved in differentiation	Epha4, Nefm, Ngf, Ntn1, Rufy3, Sema4f, Skil, Tgfbr3, Tnik, Ulk2, Wnt9b	mmu-let-7a, mmu-let-7b, mmu-let-7c, mmu-let-7e, mmu-let-7f, mmu-miR-98
GO:0010793	regulation of mRNA export from nucleus	Zc3h3	mmu-let-7a, mmu-let-7b, mmu-let-7c, mmu-let-7e, mmu-let-7f, mmu-miR-98
GO:0010811	positive regulation of cell-substrate adhesion	Dmp1, Ppm1f, Thbs1, Tsc1, Utrn	mmu-let-7a, mmu-let-7b, mmu-let-7c, mmu-let-7e, mmu-let-7f, mmu-miR-98
GO:0010862	positive regulation of pathway-restricted SMAD protein phosphorylation	Acvr1b, Gdf6, Tgfbr1	mmu-let-7a, mmu-let-7b, mmu-let-7c, mmu-let-7e, mmu-let-7f, mmu-miR-98
GO:0010886	positive regulation of cholesterol storage	Msr1, Srebf2	mmu-let-7a, mmu-let-7b, mmu-let-7c, mmu-let-7e, mmu-let-7f, mmu-miR-98
GO:0010888	negative regulation of lipid storage	Abcg1, Ppara	mmu-let-7a, mmu-let-7b, mmu-let-7c, mmu-let-7e, mmu-let-7f, mmu-miR-98
GO:0010921	regulation of phosphatase activity	Akap6, Farp1, Gpatch2, Ppargc1b, Slc7a14, Tsc1	mmu-let-7a, mmu-let-7b, mmu-let-7c, mmu-let-7e, mmu-let-7f, mmu-miR-98
GO:0010941	regulation of cell death	Abcb9, Acvr1b, Acvr1c, Adrb2, Aen, Dcun1d3, Ddx19a, Dlc1, Egln2, Ercc6, Fasl, Fnip2, Ghr, Grin2b, Hic1, Hip1, Ikbke, Ing3, Map3k1, Mycn, Plagl2, Ppara, Skil, Tbx5, Thoc1, Zbtb16	mmu-let-7a, mmu-let-7b, mmu-let-7c, mmu-let-7e, mmu-let-7f, mmu-miR-98
GO:0014013	regulation of gliogenesis	Epha4, Ezh2, Hmga2, Lin28a, Lingo1, Mycn	mmu-let-7a, mmu-let-7b, mmu-let-7c, mmu-let-7e, mmu-let-7f, mmu-miR-98

GO:0014031	mesenchymal cell development	Edn1, Hmga2, Nrtn, Rbpj, Rdh10, Tgfbr1, Tgfbr3	mmu-let-7a, mmu-let-7b, mmu-let-7c, mmu-let-7e, mmu-let-7f, mmu-miR-98
GO:0014047	glutamate secretion	Apba1, Gipc1, Grin2b	mmu-let-7a, mmu-let-7b, mmu-let-7c, mmu-let-7e, mmu-let-7f, mmu-miR-98
GO:0014051	gamma-aminobutyric acid secretion	Apba1, Slc6a1	mmu-let-7a, mmu-let-7b, mmu-let-7c, mmu-let-7e, mmu-let-7f, mmu-miR-98
GO:0014074	response to purine-containing compound	Slc6a1	mmu-let-7a, mmu-let-7b, mmu-let-7c, mmu-let-7e, mmu-let-7f, mmu-miR-98
GO:0015701	bicarbonate transport	Slc26a9, Slc4a4	mmu-let-7a, mmu-let-7b, mmu-let-7c, mmu-let-7e, mmu-let-7f, mmu-miR-98
GO:0015878	biotin transport	Slc5a6	mmu-let-7a, mmu-let-7b, mmu-let-7c, mmu-let-7e, mmu-let-7f, mmu-miR-98
GO:0015887	pantothenate transmembrane transport	Slc5a6	mmu-let-7a, mmu-let-7b, mmu-let-7c, mmu-let-7e, mmu-let-7f, mmu-miR-98
GO:0016032	viral reproduction	Bcl2l1, Hmga2, Insr, Map3k1, Tarbp2, Thoc1, Thoc2	mmu-let-7a, mmu-let-7b, mmu-let-7c, mmu-let-7e, mmu-let-7f, mmu-miR-98
GO:0016043	cellular component organization	Abcg1, Acvr1b, Adipor2, Adrb2, Ankfy1, Ap1s1, Aqp4, Braf, C230081A13Rik, Col4a1, Col4a5, Dcun1d3, Ercc6, Esrrb, Gabra6, Ghr, Grik2, Grin2b, Msr1, Nphp3, Olr1, P2rx1, Pacs2, Rbpj, Reep1, Rrm2, Slc6a1, Snap23, Snx5, Taf9b, Thbs1, Tmprss2, Usp47, Utrn, Wnt9b	mmu-let-7a, mmu-let-7b, mmu-let-7c, mmu-let-7e, mmu-let-7f, mmu-miR-98

GO:0016044	cellular membrane organization	Adrb2, Ankfy1, Ap1s1, Bcl2l1, Cap1, Casp3, Ccr7, Eea1, Ghr, Grik2, Grin2b, Hip1, Msr1, Olr1, Pacs2, Pak1, Pldn, Reep1, Sec16b, Smcr7l, Snap23, Snx5, Stx3, Thbs1, Tmprss2, Vav3	mmu-let-7a, mmu-let-7b, mmu-let-7c, mmu-let-7e, mmu-let-7f, mmu-miR-98
GO:0016049	cell growth	Acvr1b, Adipor2, Bin3, Dcun1d3, Edn1, Il6, Lmx1a, Mll2, Nme6, Sema4f, Tgfbr3, Ulk2	mmu-let-7a, mmu-let-7b, mmu-let-7c, mmu-let-7e, mmu-let-7f, mmu-miR-98
GO:0016192	vesicle-mediated transport	1500003O03Rik, Adrb2, Ankfy1, Ap1s1, Bcap29, Cap1, Ccr7, Ergic1, Gga3, Ghr, Golga7, Golt1b, Hip1, Hook1, Msr1, Myo1f, Olr1, P2rx1, Rab6b, Ralb, Sec16b, Snap23, Snx16, Snx5, Stx17, Stx3, Syt1, Thbs1, Tmprss2, Trappc1, Txlna	mmu-let-7a, mmu-let-7b, mmu-let-7c, mmu-let-7e, mmu-let-7f, mmu-miR-98
GO:0016441	posttranscriptional gene silencing	Clp1, Eif2c4, Limd1, Lin28a, Lin28b, Tarbp2	mmu-let-7a, mmu-let-7b, mmu-let-7c, mmu-let-7e, mmu-let-7f, mmu-miR-98
GO:0018022	peptidyl-lysine methylation	Ezh2, Mll2, Suv39h2	mmu-let-7a, mmu-let-7b, mmu-let-7c, mmu-let-7e, mmu-let-7f, mmu-miR-98
GO:0018105	peptidyl-serine phosphorylation	Braf, Hmga2, Il6, Map3k1, Mapk8, Nlk, Pak1, Pdgfb, Ppm1f, Prkaa2, Sbk1, Tgfbr1	mmu-let-7a, mmu-let-7b, mmu-let-7c, mmu-let-7e, mmu-let-7f, mmu-miR-98
GO:0018107	peptidyl-threonine phosphorylation	Acvr1b, Mapk8, Nlk, Sbk1, Tgfbr1	mmu-let-7a, mmu-let-7b, mmu-let-7c, mmu-let-7e, mmu-let-7f, mmu-miR-98
GO:0018149	peptide cross-linking	Col3a1, Lor, Thbs1	mmu-let-7a, mmu-let-7b, mmu-let-7c, mmu-let-7e, mmu-let-7f, mmu-miR-98
GO:0018193	peptidyl-amino acid modification	Acvr1b, Braf, Dyrk1a, Epha4, Ezh2, Galnt1, Galnt2, Ghr, Hif1an, Hmga2, Il13, Il6, Ing3, Insr, Map3k1, Mapk8, Mll2, Nlk, P4ha2, Pak1, Pdgfb, Ppm1f, Prkaa2, Rictor, Sbk1, Socs1, Suv39h2, Tgfbr1, Ttll4	mmu-let-7a, mmu-let-7b, mmu-let-7c, mmu-let-7e, mmu-let-7f, mmu-miR-98

GO:0018242	protein O-linked glycosylation via serine	Galnt1, Galnt2	mmu-let-7a, mmu-let-7b, mmu-let-7c, mmu-let-7e, mmu-let-7f, mmu-miR-98
GO:0018243	protein O-linked glycosylation via threonine	Galnt1, Galnt2	mmu-let-7a, mmu-let-7b, mmu-let-7c, mmu-let-7e, mmu-let-7f, mmu-miR-98
GO:0019080	viral genome expression	Hmga2, Map3k1, Tarbp2	mmu-let-7a, mmu-let-7b, mmu-let-7c, mmu-let-7e, mmu-let-7f, mmu-miR-98
GO:0019216	regulation of lipid metabolic process	Abcg1, Dkk3, Igf1r, Pdgfb, Ppara, Ppargc1a, Prkaa2, Rb1, Thrsp, Vav3	mmu-let-7a, mmu-let-7b, mmu-let-7c, mmu-let-7e, mmu-let-7f, mmu-miR-98
GO:0019220	regulation of phosphate metabolic process	Akap6, Casp3, Ccny, Ccr7, Dusp1, Dusp16, Dusp4, Edn1, Epha4, Ercc6, Ezh2, Farp1, Fnip1, Fnip2, Frs2, Gpatch2, Igf1r, Ikbkap, Map3k1, Mdfi, Nlrc5, Ppm1f, Prlr, Rb1, Slc7a14, Socs1, Spred3, Tab2, Tnfaip3, Tnik, Trib1, Trib2, Tsc1, Vav3, Wnt9b	mmu-let-7a, mmu-let-7b, mmu-let-7c, mmu-let-7e, mmu-let-7f, mmu-miR-98
GO:0019226	transmission of nerve impulse	Adrb2, Apba1, Arhgef15, Dlgap1, Dmd, Edn1, Gabra6, Gipc1, Grin2b, Il6, Kcnc3, Kcnc4, Nat8l, Ngf, P2rx1, Scn8a, Slc30a1, Spnb4, Syt1, Tmod2, Tsc1	mmu-let-7a, mmu-let-7b, mmu-let-7c, mmu-let-7e, mmu-let-7f, mmu-miR-98
GO:0019484	beta-alanine catabolic process	Aldh6a1	mmu-let-7a, mmu-let-7b, mmu-let-7c, mmu-let-7e, mmu-let-7f, mmu-miR-98
GO:0021549	cerebellum development	Ezh2, Foxp2, Lmx1a, Sema4c, Wnt1	mmu-let-7a, mmu-let-7b, mmu-let-7c, mmu-let-7e, mmu-let-7f, mmu-miR-98
GO:0021588	cerebellum formation	Wnt1	mmu-let-7a, mmu-let-7b, mmu-let-7c, mmu-let-7e, mmu-let-7f, mmu-miR-98
GO:0021629	olfactory nerve structural organization	Dmd	mmu-let-7a, mmu-let-7b, mmu-let-7c, mmu-let-7e, mmu-let-7f, mmu-miR-98

GO:0021757	caudate nucleus development	Foxp2	mmu-let-7a, mmu-let-7b, mmu-let-7c, mmu-let-7e, mmu-let-7f, mmu-miR-98
GO:0021758	putamen development	Foxp2	mmu-let-7a, mmu-let-7b, mmu-let-7c, mmu-let-7e, mmu-let-7f, mmu-miR-98
GO:0021919	BMP signaling pathway involved in spinal cord dorsal/ventral patterning	Chrd	mmu-let-7a, mmu-let-7b, mmu-let-7c, mmu-let-7e, mmu-let-7f, mmu-miR-98
GO:0022004	midbrain-hindbrain boundary maturation during brain development	Wnt1	mmu-let-7a, mmu-let-7b, mmu-let-7c, mmu-let-7e, mmu-let-7f, mmu-miR-98
GO:0022603	regulation of anatomical structure morphogenesis	Brwd1, Col4a2, Dlc1, Edn1, Epha4, Fasl, Foxp2, Frs2, Gas7, Gtf2i, Il6, Lgr4, Limd1, Nefm, Ngf, Ntn1, Plxnd1, Rufy3, Sema4f, Skil, Tgfbr3, Thbs1, Tnfaip3, Tnik, Ulk2, Vash2, Wnt9b	mmu-let-7a, mmu-let-7b, mmu-let-7c, mmu-let-7e, mmu-let-7f, mmu-miR-98
GO:0022610	biological adhesion	Acer2, Btbd9, C230081A13Rik, Cask, Cdh22, Cercam, Chrd, Col14a1, Col15a1, Col3a1, Dlc1, Dmp1, Eda, Epha3, Epha4, Fgf4, Fndc3a, Frem2, Lmln, Mycn, Myo1f, Nid2, Ntn1, Olr1, Pcdh20, Ppm1f, Prlr, Ptpru, Sdk1, Thbs1, Tnfaip3, Tsc1, Utrn, Vav3, Wnt1	mmu-let-7a, mmu-let-7b, mmu-let-7c, mmu-let-7e, mmu-let-7f, mmu-miR-98
GO:0022612	gland morphogenesis	Eda, Esr2, Frs2, Igf1r, Il6, Ntn1, Pdgfb, Plxnd1	mmu-let-7a, mmu-let-7b, mmu-let-7c, mmu-let-7e, mmu-let-7f, mmu-miR-98
GO:0023014	signal transduction by phosphorylation	Acvr1b, Insr	mmu-let-7a, mmu-let-7b, mmu-let-7c, mmu-let-7e, mmu-let-7f, mmu-miR-98

GO:0030029	actin filament-based process	Acta1, Cap1, Ccr7, Crk, Dlc1, Edn1, Epha3, Gas7, Grid2ip, Map3k1, Myo1f, Pak1, Pdgfb, Ppargc1b, Ppm1f, Rdx, Rictor, Tnik, Tsc1, Wasl	mmu-let-7a, mmu-let-7b, mmu-let-7c, mmu-let-7e, mmu-let-7f, mmu-miR-98
GO:0030111	regulation of Wnt receptor signaling pathway	Ccnd1, Ccny, Dkk3, Eda, Hic1, Mdfi, Nlk, Nphp3, Rspo2	mmu-let-7a, mmu-let-7b, mmu-let-7c, mmu-let-7e, mmu-let-7f, mmu-miR-98
GO:0030163	protein catabolic process	Cdc34, Dnajb9, Edem1, Edem3, Fbxl12, Fbxl14, Map3k1, Rnf20, Uhrf2, Usp12, Usp24, Usp38, Usp47, Yod1	mmu-let-7a, mmu-let-7b, mmu-let-7c, mmu-let-7e, mmu-let-7f, mmu-miR-98
GO:0030182	neuron differentiation	Dmd, Epha3, Epha4, Gas7, Gdf6, Igf1r, Il6, Lin28a, Lingo1, Lmx1a, Mapk8, Nefm, Ngf, Nrtn, Ntn1, Pak1, Pbx1, Pbx3, Rb1, Rbpj, Rufy3, Sall3, Sema4f, Skil, Slc4a7, Spnb4, Stx3, Tgfbr1, Tnik, Ulk2, Unc5a, Wnt1	mmu-let-7a, mmu-let-7b, mmu-let-7c, mmu-let-7e, mmu-let-7f, mmu-miR-98
GO:0030195	negative regulation of blood coagulation	Klkb1, Pdgfb, Thbs1	mmu-let-7a, mmu-let-7b, mmu-let-7c, mmu-let-7e, mmu-let-7f, mmu-miR-98
GO:0030199	collagen fibril organization	Adamts14, Col1a2, Col3a1, Col5a2, Tgfbr1	mmu-let-7a, mmu-let-7b, mmu-let-7c, mmu-let-7e, mmu-let-7f, mmu-miR-98
GO:0030238	male sex determination	Igf1r, Insr	mmu-let-7a, mmu-let-7b, mmu-let-7c, mmu-let-7e, mmu-let-7f, mmu-miR-98
GO:0030307	positive regulation of cell growth	Mtpn, Ngf, Ntn1, Taf9b, Tgfbr1, Usp47	mmu-let-7a, mmu-let-7b, mmu-let-7c, mmu-let-7e, mmu-let-7f, mmu-miR-98
GO:0030326	embryonic limb morphogenesis	Fgf4, Mycn, Pbx1, Pbx2, Prrx1, Rdh10, Rspo2, Sall4, Tbx5, Wnt9a, Zbtb16	mmu-let-7a, mmu-let-7b, mmu-let-7c, mmu-let-7e, mmu-let-7f, mmu-miR-98
GO:0030336	negative regulation of cell migration	Chrd, Dlc1, Pdgfb, Ptpru, Rnf20, Tbx5, Tgfbr3, Thbs1, Trib1	mmu-let-7a, mmu-let-7b, mmu-let-7c, mmu-let-7e, mmu-let-7f, mmu-miR-98

GO:0030423	targeting of mRNA for destruction involved in RNA interference	Clp1, Tarbp2	mmu-let-7a, mmu-let-7b, mmu-let-7c, mmu-let-7e, mmu-let-7f, mmu-miR-98
GO:0030433	ER-associated protein catabolic process	Dnajb9, Edem1, Yod1	mmu-let-7a, mmu-let-7b, mmu-let-7c, mmu-let-7e, mmu-let-7f, mmu-miR-98
GO:0030501	positive regulation of bone mineralization	Acvr2a, Adrb2, Cd276	mmu-let-7a, mmu-let-7b, mmu-let-7c, mmu-let-7e, mmu-let-7f, mmu-miR-98
GO:0030511	positive regulation of transforming growth factor beta receptor signaling pathway	Gipc1, Tgfbr3, Thbs1	mmu-let-7a, mmu-let-7b, mmu-let-7c, mmu-let-7e, mmu-let-7f, mmu-miR-98
GO:0030512	negative regulation of transforming growth factor beta receptor signaling pathway	Peg10, Skil, Tgfbr3, Wnt1	mmu-let-7a, mmu-let-7b, mmu-let-7c, mmu-let-7e, mmu-let-7f, mmu-miR-98
GO:0030522	intracellular receptor mediated signaling pathway	Esr2, Ezh2, Hmga2, Mll2, Pak1, Ppara, Ppargc1b, Tnfaip3	mmu-let-7a, mmu-let-7b, mmu-let-7c, mmu-let-7e, mmu-let-7f, mmu-miR-98
GO:0030886	negative regulation of myeloid dendritic cell activation	Il10	mmu-let-7a, mmu-let-7b, mmu-let-7c, mmu-let-7e, mmu-let-7f, mmu-miR-98
GO:0030889	negative regulation of B cell proliferation	Casp3, Il10, Prdm1	mmu-let-7a, mmu-let-7b, mmu-let-7c, mmu-let-7e, mmu-let-7f, mmu-miR-98
GO:0030902	hindbrain development	Dlc1, Ezh2, Foxp2, Lmx1a, Sema4c, Slc4a7, Wnt1	mmu-let-7a, mmu-let-7b, mmu-let-7c, mmu-let-7e, mmu-let-7f, mmu-miR-98

GO:0030968	endoplasmic reticulum unfolded protein response	Ccnd1, Ero1l, Yod1	mmu-let-7a, mmu-let-7b, mmu-let-7c, mmu-let-7e, mmu-let-7f, mmu-miR-98
GO:0031000	response to caffeine	Gnal, Prkaa2	mmu-let-7a, mmu-let-7b, mmu-let-7c, mmu-let-7e, mmu-let-7f, mmu-miR-98
GO:0031017	exocrine pancreas development	Igf1r, Insr	mmu-let-7a, mmu-let-7b, mmu-let-7c, mmu-let-7e, mmu-let-7f, mmu-miR-98
GO:0031052	chromosome breakage	Hmga2	mmu-let-7a, mmu-let-7b, mmu-let-7c, mmu-let-7e, mmu-let-7f, mmu-miR-98
GO:0031054	pre-miRNA processing	Lin28a, Lin28b, Tarbp2	mmu-let-7a, mmu-let-7b, mmu-let-7c, mmu-let-7e, mmu-let-7f, mmu-miR-98
GO:0031123	RNA 3'-end processing	Lin28a, Lin28b, Lsm11, Zc3h3	mmu-let-7a, mmu-let-7b, mmu-let-7c, mmu-let-7e, mmu-let-7f, mmu-miR-98
GO:0031175	neuron projection development	Dmd, Epha3, Epha4, Gas7, Igf1r, Il6, Lingo1, Lmx1a, Mapk8, Nefm, Ngf, Nrtn, Ntn1, Pak1, Rb1, Rufy3, Sema4f, Skil, Spnb4, Stx3, Tnik, Ulk2, Unc5a	mmu-let-7a, mmu-let-7b, mmu-let-7c, mmu-let-7e, mmu-let-7f, mmu-miR-98
GO:0031329	regulation of cellular catabolic process	Arhgap20, Arhgap28, Arhgef15, Ccr7, Crk, Dlc1, Dlgap1, Epha3, Epha4, Gipc1, Il10, Plekhg6, Rgs16, Rictor, Srgap3, Stard13, Trib1, Trib2, Tsc1, Vav3, Wnt1	mmu-let-7a, mmu-let-7b, mmu-let-7c, mmu-let-7e, mmu-let-7f, mmu-miR-98
GO:0031344	regulation of cell projection organization	Ccr7, Epha3, Epha4, Nefm, Ngf, Ntn1, Rab8b, Rufy3, Sema4f, Skil, Tgfbr1, Tnik, Ulk2, Wnt1	mmu-let-7a, mmu-let-7b, mmu-let-7c, mmu-let-7e, mmu-let-7f, mmu-miR-98

GO:0031347	regulation of defense response	Adrb2, Ccr7, Cd276, Cd86, Il10, Il6, Masp1, Mgll, Myo1f, Nlrc5, Polr3d, Tarbp2, Tnfaip3, Tnfrsf1b	mmu-let-7a, mmu-let-7b, mmu-let-7c, mmu-let-7e, mmu-let-7f, mmu-miR-98
GO:0031397	negative regulation of protein ubiquitination	1810074P20Rik, Tnfaip3, Wnt1	mmu-let-7a, mmu-let-7b, mmu-let-7c, mmu-let-7e, mmu-let-7f, mmu-miR-98
GO:0031570	DNA integrity checkpoint	Bcat1, Ccnd1, Hmga2, Rbm38, Taok1, Wnt1	mmu-let-7a, mmu-let-7b, mmu-let-7c, mmu-let-7e, mmu-let-7f, mmu-miR-98
GO:0031571	mitotic cell cycle G1/S transition DNA damage checkpoint	Bcat1, Ccnd1, Rbm38	mmu-let-7a, mmu-let-7b, mmu-let-7c, mmu-let-7e, mmu-let-7f, mmu-miR-98
GO:0031589	cell-substrate adhesion	Acer2, C230081A13Rik, Cask, Col3a1, Dlc1, Eda, Epha3, Nid2, Wnt1	mmu-let-7a, mmu-let-7b, mmu-let-7c, mmu-let-7e, mmu-let-7f, mmu-miR-98
GO:0031644	regulation of neurological system process	Adrb2, Edn1, Gipc1, Grid2ip, Grik2, Grin2b, Il10, Il6, Kcnc3, Kcnc4, Mgll, Nat8l, Ngf, Slc30a1, Slc6a1	mmu-let-7a, mmu-let-7b, mmu-let-7c, mmu-let-7e, mmu-let-7f, mmu-miR-98
GO:0031665	negative regulation of lipopolysaccharide-mediated signaling pathway	Prdm1, Trib1	mmu-let-7a, mmu-let-7b, mmu-let-7c, mmu-let-7e, mmu-let-7f, mmu-miR-98
GO:0031954	positive regulation of protein autophosphorylation	Ngf, Pdgfb	mmu-let-7a, mmu-let-7b, mmu-let-7c, mmu-let-7e, mmu-let-7f, mmu-miR-98
GO:0032006	regulation of TOR signaling cascade	Prkaa2, Rictor, Tsc1	mmu-let-7a, mmu-let-7b, mmu-let-7c, mmu-let-7e, mmu-let-7f, mmu-miR-98
GO:0032091	negative regulation of protein binding	Adrb2, Mapk8, Ppara, Senp2	mmu-let-7a, mmu-let-7b, mmu-let-7c, mmu-let-7e, mmu-let-7f, mmu-miR-98

GO:0032101	regulation of response to external stimulus	Adrb2, Cask, Ccr7, Cd276, Edn1, Epha4, Il10, Il6, Klkb1, Masp1, Mgll, Pdgfb, Ppara, Ppm1f, Thbs1, Tnfaip3, Tnfrsf1b	mmu-let-7a, mmu-let-7b, mmu-let-7c, mmu-let-7e, mmu-let-7f, mmu-miR-98
GO:0032147	activation of protein kinase activity	Braf, Ccr7, Edn1, Ercc6, Frs2, Ghr, Insr, Map3k1, Mdfi, Pdgfb, Prlr, Tgfbr1, Thbs1, Tnik	mmu-let-7a, mmu-let-7b, mmu-let-7c, mmu-let-7e, mmu-let-7f, mmu-miR-98
GO:0032268	regulation of cellular protein metabolic process	Casp3, Ccny, Ccr7, Dlgap1, Dusp1, Dusp16, Dusp4, Epha4, Ercc6, Ezh2, Fnip1, Fnip2, Frs2, Igf1r, Map3k1, Mapk8, Mdfi, Prlr, Rb1, Spred3, Tab2, Tnik, Wnt9b	mmu-let-7a, mmu-let-7b, mmu-let-7c, mmu-let-7e, mmu-let-7f, mmu-miR-98
GO:0032269	negative regulation of cellular protein metabolic process	1810074P20Rik, Acer2, Edn1, Eif2c4, Gipc1, Il10, Insr, Ppm1f, Socs1, Tia1, Tnfaip3, Tsc1, Wnt1	mmu-let-7a, mmu-let-7b, mmu-let-7c, mmu-let-7e, mmu-let-7f, mmu-miR-98
GO:0032270	positive regulation of cellular protein metabolic process	Acvr1b, Acvr2a, Braf, Ccnd1, Ccnd2, Dlc1, Gdf6, Ghr, Il13, Il6, Insr, Lin28a, Ngf, Pak1, Pdgfb, Rictor, Senp2, Taok1, Tgfbr1, Thbs1, Trib1, Trib2, Wnt1	mmu-let-7a, mmu-let-7b, mmu-let-7c, mmu-let-7e, mmu-let-7f, mmu-miR-98
GO:0032319	regulation of Rho GTPase activity	Ccr7, Crk, Dlc1, Epha3, Epha4, Rictor, Tsc1	mmu-let-7a, mmu-let-7b, mmu-let-7c, mmu-let-7e, mmu-let-7f, mmu-miR-98
GO:0032502	developmental process	Adrb3, Aldh6a1, Brwd1, C230081A13Rik, Cap1, Cap2, Ero1l, Gnptab, Hip1, Limd1, Pappa, Pla2g3, Pldn, Ptpru, Rasgrp1, Socs1, Socs7, Speg, Suv39h2, Tmem231, Uhrf2	mmu-let-7a, mmu-let-7b, mmu-let-7c, mmu-let-7e, mmu-let-7f, mmu-miR-98
GO:0032504	multicellular organism reproduction	Acvr2a, Agpat6, Bcl2l1, Ccnd1, Dzip1, Esr2, Fndc3a, Hmga1, Hmga2, Hook1, Limk2, Lin28a, Mll2, Nr6a1, P2rx1, Pcyt1b, Prdm1, Prlr, Strbp, Styx, Tgfbr1, Zbtb16	mmu-let-7a, mmu-let-7b, mmu-let-7c, mmu-let-7e, mmu-let-7f, mmu-miR-98
GO:0032514	positive regulation of protein phosphatase type 2B activity	Akap6	mmu-let-7a, mmu-let-7b, mmu-let-7c, mmu-let-7e, mmu-let-7f, mmu-miR-98

GO:0032535	regulation of cellular component size	Edn1, Map3k1, Mtpn, Nefm, Ngf, Ntn1, Rdx, Rictor, Sema4f, Tsc1, Ulk2, Wasl	mmu-let-7a, mmu-let-7b, mmu-let-7c, mmu-let-7e, mmu-let-7f, mmu-miR-98
GO:0032695	negative regulation of interleukin-12 production	Il10, Thbs1	mmu-let-7a, mmu-let-7b, mmu-let-7c, mmu-let-7e, mmu-let-7f, mmu-miR-98
GO:0032723	positive regulation of connective tissue growth factor production	Il13	mmu-let-7a, mmu-let-7b, mmu-let-7c, mmu-let-7e, mmu-let-7f, mmu-miR-98
GO:0032784	regulation of transcription elongation, DNA-dependent	Ercc6, Ezh2, Thoc1	mmu-let-7a, mmu-let-7b, mmu-let-7c, mmu-let-7e, mmu-let-7f, mmu-miR-98
GO:0032800	receptor biosynthetic process	Edn1, Il10, Ppara	mmu-let-7a, mmu-let-7b, mmu-let-7c, mmu-let-7e, mmu-let-7f, mmu-miR-98
GO:0032868	response to insulin stimulus	Acvr1c, Insr, Pak1, Ppara, Smarcc1, Socs1, Socs7, Tsc1	mmu-let-7a, mmu-let-7b, mmu-let-7c, mmu-let-7e, mmu-let-7f, mmu-miR-98
GO:0032879	regulation of localization	Abi3, Bcl2l1, Cep120, Chrd, Dlc1, Igf1r, Map3k1, Mapk8, Msr1, Myo1f, Ntn1, Plxnd1, Ppm1f, Ptpru, Rb1, Rnf20, Tbx5, Tgfbr3, Trib1	mmu-let-7a, mmu-let-7b, mmu-let-7c, mmu-let-7e, mmu-let-7f, mmu-miR-98
GO:0032927	positive regulation of activin receptor signaling pathway	Acvr1b, Acvr2a	mmu-let-7a, mmu-let-7b, mmu-let-7c, mmu-let-7e, mmu-let-7f, mmu-miR-98
GO:0032940	secretion by cell	1500003O03Rik, Acvr1c, Acvr2a, Ccr7, Dnajc1, Edn1, Gnptab, Hmga2, Il10, Il13, Il6, Kcnc3, Kcnc4, Myo1f, Ngf, P2rx1, Pldn, Rab8b, Ralb, Rbpj, Rfx6, Slc30a1, Snap23, Stx3, Syt1, Txlna	mmu-let-7a, mmu-let-7b, mmu-let-7c, mmu-let-7e, mmu-let-7f, mmu-miR-98

GO:0032943	mononuclear cell proliferation	Casp3, Ccr7, Cd276, Cd86, Il10, Il13, Il6, Prdm1, Vav3, Elf4	mmu-let-7a, mmu-let-7b, mmu-let-7c, mmu-let-7e, mmu-let-7f, mmu-miR-98
GO:0032956	regulation of actin cytoskeleton organization	Crk, Dlc1, Edn1, Epha3, Map3k1, Mtpn, Myo1f, Pak1, Ppm1f, Rdx, Rictor, Tsc1, Wasl	mmu-let-7a, mmu-let-7b, mmu-let-7c, mmu-let-7e, mmu-let-7f, mmu-miR-98
GO:0033002	muscle cell proliferation	Edn1, Il13, Il6, Pdgfb, Tbx5, Tgfbr3, Tnfaip3, Trib1	mmu-let-7a, mmu-let-7b, mmu-let-7c, mmu-let-7e, mmu-let-7f, mmu-miR-98
GO:0033043	regulation of organelle organization	Clasp2, Crk, Dlc1, Epha3, Map3k1, Mapk8, Mtpn, Myo1f, Pak1, Ppm1f, Rdx, Rictor, Taok1, Tsc1, Wasl, Ercc4	mmu-let-7a, mmu-let-7b, mmu-let-7c, mmu-let-7e, mmu-let-7f, mmu-miR-98
GO:0033121	regulation of purine nucleotide catabolic process	Arhgap20, Arhgap28, Arhgef15, Ccr7, Crk, Dlc1, Epha3, Epha4, Gipc1, Plekhg6, Rgs16, Rictor, Srgap3, Stard13, Tsc1, Vav3	mmu-let-7a, mmu-let-7b, mmu-let-7c, mmu-let-7e, mmu-let-7f, mmu-miR-98
GO:0033143	regulation of intracellular steroid hormone receptor signaling pathway	Esr2, Hmga2, Mll2, Pak1	mmu-let-7a, mmu-let-7b, mmu-let-7c, mmu-let-7e, mmu-let-7f, mmu-miR-98
GO:0033148	positive regulation of intracellular estrogen receptor signaling pathway	Mll2, Pak1	mmu-let-7a, mmu-let-7b, mmu-let-7c, mmu-let-7e, mmu-let-7f, mmu-miR-98
GO:0033673	negative regulation of kinase activity	Casp3, Dusp1, Dusp16, Dusp4, Igf1r, Il6, Ppm1f, Rb1, Spred3, Tnfaip3, Trib1	mmu-let-7a, mmu-let-7b, mmu-let-7c, mmu-let-7e, mmu-let-7f, mmu-miR-98
GO:0033674	positive regulation of kinase activity	Braf, Ccnd1, Ccnd2, Ccny, Ccr7, Edn1, Epha4, Ercc6, Frs2, Ghr, Insr, Map3k1, Mdfi, Pak1, Pdgfb, Prlr, Tab2, Tgfbr1, Thbs1, Tnik, Vav3	mmu-let-7a, mmu-let-7b, mmu-let-7c, mmu-let-7e, mmu-let-7f, mmu-miR-98
GO:0033993	response to lipid	Abcg1, Ccr7, Pdgfb, Tgfbr1, Tgfbr3	mmu-let-7a, mmu-let-7b, mmu-let-7c, mmu-let-7e, mmu-let-7f, mmu-miR-98

GO:0034148	negative regulation of toll-like receptor 5 signaling pathway	Tnfaip3	mmu-let-7a, mmu-let-7b, mmu-let-7c, mmu-let-7e, mmu-let-7f, mmu-miR-98
GO:0034629	cellular protein complex localization	Ralb, Wasl	mmu-let-7a, mmu-let-7b, mmu-let-7c, mmu-let-7e, mmu-let-7f, mmu-miR-98
GO:0034637	cellular carbohydrate biosynthetic process	Arpp19, B3gnt1, Cercam, Il6, Insr, Lalba, Pcx	mmu-let-7a, mmu-let-7b, mmu-let-7c, mmu-let-7e, mmu-let-7f, mmu-miR-98
GO:0034644	cellular response to UV	Bcat1, Cdc25a, Usp47	mmu-let-7a, mmu-let-7b, mmu-let-7c, mmu-let-7e, mmu-let-7f, mmu-miR-98
GO:0034695	response to prostaglandin E stimulus	Ccr7, Tgfbr3	mmu-let-7a, mmu-let-7b, mmu-let-7c, mmu-let-7e, mmu-let-7f, mmu-miR-98
GO:0034699	response to luteinizing hormone stimulus	Tgfbr3	mmu-let-7a, mmu-let-7b, mmu-let-7c, mmu-let-7e, mmu-let-7f, mmu-miR-98
GO:0035020	regulation of Rac protein signal transduction	Crk, Epha4, Rictor	mmu-let-7a, mmu-let-7b, mmu-let-7c, mmu-let-7e, mmu-let-7f, mmu-miR-98
GO:0035087	siRNA loading onto RISC involved in RNA interference	Clp1	mmu-let-7a, mmu-let-7b, mmu-let-7c, mmu-let-7e, mmu-let-7f, mmu-miR-98
GO:0035107	appendage morphogenesis	Fgf4, Mycn, Pbx1, Pbx2, Prrx1, Rdh10, Rspo2, Sall3, Sall4, Tbx5, Wnt9a, Zbtb16	mmu-let-7a, mmu-let-7b, mmu-let-7c, mmu-let-7e, mmu-let-7f, mmu-miR-98
GO:0035117	embryonic arm morphogenesis	Tbx5, Wnt9a	mmu-let-7a, mmu-let-7b, mmu-let-7c, mmu-let-7e, mmu-let-7f, mmu-miR-98

GO:0035136	forelimb morphogenesis	Rdh10, Rspo2, Sall3, Tbx5, Wnt9a, Zbtb16	mmu-let-7a, mmu-let-7b, mmu-let-7c, mmu-let-7e, mmu-let-7f, mmu-miR-98
GO:0035137	hindlimb morphogenesis	Fgf4, Rspo2, Sall3, Zbtb16	mmu-let-7a, mmu-let-7b, mmu-let-7c, mmu-let-7e, mmu-let-7f, mmu-miR-98
GO:0035195	gene silencing by miRNA	Eif2c4, Limd1, Lin28a, Lin28b	mmu-let-7a, mmu-let-7b, mmu-let-7c, mmu-let-7e, mmu-let-7f, mmu-miR-98
GO:0035280	miRNA loading onto RISC involved in gene silencing by miRNA	Tarbp2	mmu-let-7a, mmu-let-7b, mmu-let-7c, mmu-let-7e, mmu-let-7f, mmu-miR-98
GO:0035725	sodium ion transmembrane transport	Scn11a, Scn4b, Scn5a, Scn8a, Slc10a7, Slc5a6, Slc6a1	mmu-let-7a, mmu-let-7b, mmu-let-7c, mmu-let-7e, mmu-let-7f, mmu-miR-98
GO:0035967	cellular response to topologically incorrect protein	Ccnd1, Ero1l, Yod1	mmu-let-7a, mmu-let-7b, mmu-let-7c, mmu-let-7e, mmu-let-7f, mmu-miR-98
GO:0035970	peptidyl-threonine dephosphorylation	Pdp2, Ppm1f	mmu-let-7a, mmu-let-7b, mmu-let-7c, mmu-let-7e, mmu-let-7f, mmu-miR-98
GO:0035978	histone H2A-S139 phosphorylation	Hmga2	mmu-let-7a, mmu-let-7b, mmu-let-7c, mmu-let-7e, mmu-let-7f, mmu-miR-98
GO:0035986	senescence-associated heterochromatin focus assembly	Hmga1, Hmga2	mmu-let-7a, mmu-let-7b, mmu-let-7c, mmu-let-7e, mmu-let-7f, mmu-miR-98
GO:0040007	growth	Adrb3, Agpat6, Apba1, Bcl2l1, Etnk2, Foxp2, Gdf6, Il10, Ing3, Rspo2, Socs1, Socs7	mmu-let-7a, mmu-let-7b, mmu-let-7c, mmu-let-7e, mmu-let-7f, mmu-miR-98

GO:0040013	negative regulation of locomotion	Chrd, Dlc1, Pdgfb, Ptpru, Rnf20, Tbx5, Tgfbr3, Thbs1, Trib1	mmu-let-7a, mmu-let-7b, mmu-let-7c, mmu-let-7e, mmu-let-7f, mmu-miR-98
GO:0040015	negative regulation of multicellular organism growth	Adrb2, Adrb3	mmu-let-7a, mmu-let-7b, mmu-let-7c, mmu-let-7e, mmu-let-7f, mmu-miR-98
GO:0042036	negative regulation of cytokine biosynthetic process	Cd276, Il10, Il6, Tia1	mmu-let-7a, mmu-let-7b, mmu-let-7c, mmu-let-7e, mmu-let-7f, mmu-miR-98
GO:0042060	wound healing	Adrb2, Cask, Col3a1, Klkb1, Map3k1, P2rx1, Pak1, Pdgfb, Pldn, Ppara, Thbs1	mmu-let-7a, mmu-let-7b, mmu-let-7c, mmu-let-7e, mmu-let-7f, mmu-miR-98
GO:0042176	regulation of protein catabolic process	Atg4b, Dlgap1, Egln2, Gipc1, Il10, Tnfaip3, Trib1, Trib2, Wnt1	mmu-let-7a, mmu-let-7b, mmu-let-7c, mmu-let-7e, mmu-let-7f, mmu-miR-98
GO:0042265	peptidyl-asparagine hydroxylation	Hif1an	mmu-let-7a, mmu-let-7b, mmu-let-7c, mmu-let-7e, mmu-let-7f, mmu-miR-98
GO:0042313	protein kinase C deactivation	Edn1	mmu-let-7a, mmu-let-7b, mmu-let-7c, mmu-let-7e, mmu-let-7f, mmu-miR-98
GO:0042340	keratan sulfate catabolic process	Gns	mmu-let-7a, mmu-let-7b, mmu-let-7c, mmu-let-7e, mmu-let-7f, mmu-miR-98
GO:0042526	positive regulation of tyrosine phosphorylation of Stat6 protein	Il13	mmu-let-7a, mmu-let-7b, mmu-let-7c, mmu-let-7e, mmu-let-7f, mmu-miR-98
GO:0042981	regulation of apoptotic process	Abcb9, Acvr1b, Acvr1c, Adrb2, Aen, Dcun1d3, Ddx19a, Dlc1, Egln2, Ercc6, Fasl, Fnip2, Ghr, Hic1, Hip1, Ikbke, Ing3, Map3k1, Plagl2, Ppara, Skil, Tbx5, Thoc1, Zbtb16	mmu-let-7a, mmu-let-7b, mmu-let-7c, mmu-let-7e, mmu-let-7f, mmu-miR-98

GO:0043032	positive regulation of macrophage activation	Il13, Thbs1	mmu-let-7a, mmu-let-7b, mmu-let-7c, mmu-let-7e, mmu-let-7f, mmu-miR-98
GO:0043066	negative regulation of apoptotic process	Apbb3, Bcl2l1, Braf, Casp3, Ccr7, Dusp1, Edn1, Esr2, Fgf4, Grik2, Hmga2, Igf1r, Il10, Il6, Mapk8, Ngf, Ppargc1a, Prkaa2, Qars, Rnf7, Taf9b, Tgfbr1, Thbs1, Tnfaip3, Usp47, Wnt1	mmu-let-7a, mmu-let-7b, mmu-let-7c, mmu-let-7e, mmu-let-7f, mmu-miR-98
GO:0043068	positive regulation of programmed cell death	Abcb9, Acvr1b, Acvr1c, Adrb2, Aen, Bcl2l1, Casp3, Dcun1d3, Ddx19a, Dlc1, Dusp1, Ercc6, Esr2, Fasl, Fnip2, Hic1, Hip1, Hmga2, Ikbke, Il10, Ing3, Map3k1, Mapk8, Plagl2, Rnf7, Tbx5, Tgfbr1, Thbs1, Zbtb16	mmu-let-7a, mmu-let-7b, mmu-let-7c, mmu-let-7e, mmu-let-7f, mmu-miR-98
GO:0043085	positive regulation of catalytic activity	Adrb2, Adrb3, Braf, Ccnd1, Ccnd2, Ccny, Epha4, Ercc6, Frs2, Ghr, Gnal, Htr4, Insr, Map3k1, Mdfi, Pak1, Pdgfb, Prlr, Tab2, Tgfbr1, Thbs1, Tnik, Trib1, Trib2, Vav3, Wnt9b	mmu-let-7a, mmu-let-7b, mmu-let-7c, mmu-let-7e, mmu-let-7f, mmu-miR-98
GO:0043087	regulation of GTPase activity	Arhgap20, Arhgap28, Arhgef15, Ccr7, Crk, Dlc1, Epha3, Epha4, Gipc1, Plekhg6, Rgs16, Rictor, Srgap3, Stard13, Tsc1, Vav3	mmu-let-7a, mmu-let-7b, mmu-let-7c, mmu-let-7e, mmu-let-7f, mmu-miR-98
GO:0043113	receptor clustering	Grik2, Grin2b, Pak1	mmu-let-7a, mmu-let-7b, mmu-let-7c, mmu-let-7e, mmu-let-7f, mmu-miR-98
GO:0043154	negative regulation of cysteine-type endopeptidase activity involved in apoptotic process	Bcl2l1, Il6, Thbs1, Tnfaip3, Usp47, Wnt9a	mmu-let-7a, mmu-let-7b, mmu-let-7c, mmu-let-7e, mmu-let-7f, mmu-miR-98
GO:0043251	sodium-dependent organic anion transport	Slc10a6	mmu-let-7a, mmu-let-7b, mmu-let-7c, mmu-let-7e, mmu-let-7f, mmu-miR-98

GO:0043392	negative regulation of DNA binding	Habp4, Hmga2, Mdfi, Ppara, Zfp462	mmu-let-7a, mmu-let-7b, mmu-let-7c, mmu-let-7e, mmu-let-7f, mmu-miR-98
GO:0043405	regulation of MAP kinase activity	Ccr7, Dusp1, Dusp16, Dusp4, Edn1, Epha4, Ercc6, Frs2, Ghr, Insr, Map3k1, Mdfi, Pak1, Pdgfb, Spred3, Thbs1, Trib1	mmu-let-7a, mmu-let-7b, mmu-let-7c, mmu-let-7e, mmu-let-7f, mmu-miR-98
GO:0043408	regulation of MAPK cascade	Ccr7, Dusp1, Dusp16, Dusp4, Edn1, Epha4, Ercc6, Grik2, Grin2b, Map3k1, Map4k4, Mdfi, Pak1, Spred3	mmu-let-7a, mmu-let-7b, mmu-let-7c, mmu-let-7e, mmu-let-7f, mmu-miR-98
GO:0043410	positive regulation of MAPK cascade	Adrb2, Adrb3, Braf, Dusp22, Esr2, Fgf4, Igf1r, Il6, Insr, Pdgfb, Sema4c, Taok1	mmu-let-7a, mmu-let-7b, mmu-let-7c, mmu-let-7e, mmu-let-7f, mmu-miR-98
GO:0043433	negative regulation of sequence-specific DNA binding transcription factor activity	1810074P20Rik, Hand1, Nlrc5, Pbx1, Tnfaip3, Trib1, Trib2	mmu-let-7a, mmu-let-7b, mmu-let-7c, mmu-let-7e, mmu-let-7f, mmu-miR-98
GO:0043471	regulation of cellular carbohydrate catabolic process	Insr, Ppara, Prkaa2	mmu-let-7a, mmu-let-7b, mmu-let-7c, mmu-let-7e, mmu-let-7f, mmu-miR-98
GO:0043507	positive regulation of JUN kinase activity	Edn1, Epha4, Pak1	mmu-let-7a, mmu-let-7b, mmu-let-7c, mmu-let-7e, mmu-let-7f, mmu-miR-98
GO:0043526	neuroprotection	Esr2, Ghr, Il6, Ppara	mmu-let-7a, mmu-let-7b, mmu-let-7c, mmu-let-7e, mmu-let-7f, mmu-miR-98
GO:0043568	positive regulation of insulin-like growth factor receptor signaling pathway	AI464131, Wnt1	mmu-let-7a, mmu-let-7b, mmu-let-7c, mmu-let-7e, mmu-let-7f, mmu-miR-98

GO:0043588	skin development	Col1a2, Col3a1, Col5a2, Eda, Fras1, Tcf7l1	mmu-let-7a, mmu-let-7b, mmu-let-7c, mmu-let-7e, mmu-let-7f, mmu-miR-98
GO:0043632	modification-dependent macromolecule catabolic process	Cdc34, Dnajb9, Edem1, Edem3, Fbxl12, Fbxl14, Gipc1, Map3k1, Rnf20, Trib1, Trib2, Uhrf2, Usp12, Usp24, Usp38, Usp47, Wnt1, Yod1	mmu-let-7a, mmu-let-7b, mmu-let-7c, mmu-let-7e, mmu-let-7f, mmu-miR-98
GO:0043900	regulation of multi-organism process	Il10, Prdm1, Tarbp2, Tnfaip3, Trib1	mmu-let-7a, mmu-let-7b, mmu-let-7c, mmu-let-7e, mmu-let-7f, mmu-miR-98
GO:0044092	negative regulation of molecular function	Adrb2, Bcl2l1, Casp3, Dusp1, Dusp16, Dusp4, Farp1, Gpatch2, Habp4, Hmga2, Igf1r, Il6, Insr, Mapk8, Mdfi, Ppara, Ppm1f, Rb1, Senp2, Slc7a14, Spred3, Thbs1, Usp47, Wnt9a, Zfp462	mmu-let-7a, mmu-let-7b, mmu-let-7c, mmu-let-7e, mmu-let-7f, mmu-miR-98
GO:0044246	regulation of multicellular organismal metabolic process	Adrb2, Adrb3, Il6	mmu-let-7a, mmu-let-7b, mmu-let-7c, mmu-let-7e, mmu-let-7f, mmu-miR-98
GO:0044387	negative regulation of protein kinase activity by regulation of protein phosphorylation	Ppm1f	mmu-let-7a, mmu-let-7b, mmu-let-7c, mmu-let-7e, mmu-let-7f, mmu-miR-98
GO:0045017	glycerolipid biosynthetic process	Agpat6, Etnk1, Etnk2, Pcyt1b, Pdgfb, Piga, Thrsp	mmu-let-7a, mmu-let-7b, mmu-let-7c, mmu-let-7e, mmu-let-7f, mmu-miR-98
GO:0045081	negative regulation of interleukin-10 biosynthetic process	Trib2	mmu-let-7a, mmu-let-7b, mmu-let-7c, mmu-let-7e, mmu-let-7f, mmu-miR-98

GO:0045444	fat cell differentiation	Ccnd1, Fndc3b, Hmga2, Socs1, Socs7, Trib2, Wnt1, Zbtb16	mmu-let-7a, mmu-let-7b, mmu-let-7c, mmu-let-7e, mmu-let-7f, mmu-miR-98
GO:0045494	photoreceptor cell maintenance	Ercc6, Iqcb1, Nphp3	mmu-let-7a, mmu-let-7b, mmu-let-7c, mmu-let-7e, mmu-let-7f, mmu-miR-98
GO:0045596	negative regulation of cell differentiation	Ccnd1, Chrd, Esrrb, Ezh2, Fgf4, Lmx1a, Nkap, Pbx1, Rbpj, Skil, Tcf7l1, Tgfbr1, Tob2, Trib2, Wnt1, Wnt9a, Zbtb16	mmu-let-7a, mmu-let-7b, mmu-let-7c, mmu-let-7e, mmu-let-7f, mmu-miR-98
GO:0045624	positive regulation of T-helper cell differentiation	Ccr7, Il6	mmu-let-7a, mmu-let-7b, mmu-let-7c, mmu-let-7e, mmu-let-7f, mmu-miR-98
GO:0045686	negative regulation of glial cell differentiation	Hmga2, Lin28a, Lingo1, Mycn	mmu-let-7a, mmu-let-7b, mmu-let-7c, mmu-let-7e, mmu-let-7f, mmu-miR-98
GO:0045737	positive regulation of cyclin-dependent protein kinase activity	Ccnd1, Ccnd2, Ccny, Pdgfb	mmu-let-7a, mmu-let-7b, mmu-let-7c, mmu-let-7e, mmu-let-7f, mmu-miR-98
GO:0045740	positive regulation of DNA replication	Igf1r, Il6, Insr, Mapk8, Pdgfb	mmu-let-7a, mmu-let-7b, mmu-let-7c, mmu-let-7e, mmu-let-7f, mmu-miR-98
GO:0045768	positive regulation of anti-apoptosis	Bcl2l1, Dusp1, Igf1r, Il6	mmu-let-7a, mmu-let-7b, mmu-let-7c, mmu-let-7e, mmu-let-7f, mmu-miR-98
GO:0045778	positive regulation of ossification	Tob2, Zbtb16	mmu-let-7a, mmu-let-7b, mmu-let-7c, mmu-let-7e, mmu-let-7f, mmu-miR-98
GO:0045786	negative regulation of cell cycle	Casp3, Dcun1d3, Nme6, Tnfaip3, Usp47	mmu-let-7a, mmu-let-7b, mmu-let-7c, mmu-let-7e, mmu-let-7f, mmu-miR-98
GO:0045821	positive regulation of glycolysis	Insr, Prkaa2	mmu-let-7a, mmu-let-7b, mmu-let-7c, mmu-let-7e, mmu-let-7f, mmu-miR-98

GO:0045840	positive regulation of mitosis	Edn1, Insr, Pdgfb, Rb1	mmu-let-7a, mmu-let-7b, mmu-let-7c, mmu-let-7e, mmu-let-7f, mmu-miR-98
GO:0045859	regulation of protein kinase activity	Braf, Casp3, Ccnd1, Ccnd2, Ccny, Ccr7, Dusp1, Dusp16, Dusp4, Edn1, Epha4, Ercc6, Frs2, Ghr, Igf1r, Il6, Insr, Map3k1, Mdfi, Pak1, Pdgfb, Ppm1f, Prlr, Rb1, Spred3, Tab2, Tgfbr1, Thbs1, Tnfaip3, Tnik, Trib1, Tsc1	mmu-let-7a, mmu-let-7b, mmu-let-7c, mmu-let-7e, mmu-let-7f, mmu-miR-98
GO:0045879	negative regulation of smoothened signaling pathway	Chrd, Rb1, Sall3	mmu-let-7a, mmu-let-7b, mmu-let-7c, mmu-let-7e, mmu-let-7f, mmu-miR-98
GO:0045892	negative regulation of transcription, DNA-dependent	Cbx5, Dkk3, Ezh2, Hmga1, Lgr4, Limd1, Mll2, Pdgfb, Phf8, Ppargc1b, Ppm1f, Rbpj, Tcf7l1, Usp47, Zbtb16	mmu-let-7a, mmu-let-7b, mmu-let-7c, mmu-let-7e, mmu-let-7f, mmu-miR-98
GO:0045927	positive regulation of growth	Adrb2, Ghr, Hmga2, Insr, Ppm1f, Spnb4	mmu-let-7a, mmu-let-7b, mmu-let-7c, mmu-let-7e, mmu-let-7f, mmu-miR-98
GO:0045937	positive regulation of phosphate metabolic process	Acvr1b, Acvr2a, Braf, Ccnd1, Ccnd2, Dlc1, Gdf6, Ghr, Il13, Il6, Insr, Ngf, Pak1, Pdgfb, Ppargc1b, Rictor, Senp2, Taok1, Tgfbr1, Thbs1, Wnt1	mmu-let-7a, mmu-let-7b, mmu-let-7c, mmu-let-7e, mmu-let-7f, mmu-miR-98
GO:0045944	positive regulation of transcription from RNA polymerase II promoter	Acvr1b, Adrb2, Cask, Esr2, Fgf4, Gabpa, Hand1, Hif3a, Hmga1, Hmga2, Ikzf2, Il10, Il6, Map3k1, Meis2, Mll2, Mllt10, Mycn, Nlrc5, Pbx1, Pbx2, Plagl2, Ppargc1a, Ppargc1b, Rb1, Rbpj, Sall4, Smarcc1, Srebf2, Tbx5, Tcf7l1, Tead3, Tead4, Wnt1, Zfp462, Elf4	mmu-let-7a, mmu-let-7b, mmu-let-7c, mmu-let-7e, mmu-let-7f, mmu-miR-98
GO:0046321	positive regulation of fatty acid oxidation	Ppara, Ppargc1a	mmu-let-7a, mmu-let-7b, mmu-let-7c, mmu-let-7e, mmu-let-7f, mmu-miR-98
GO:0046328	regulation of JNK cascade	Ccr7, Dusp22, Edn1, Epha4, Ercc6, Grik2, Igf1r, Map3k1, Map4k4, Mdfi, Pak1, Taok1	mmu-let-7a, mmu-let-7b, mmu-let-7c, mmu-let-7e, mmu-let-7f, mmu-miR-98

GO:0046425	regulation of JAK-STAT cascade	Dot1l, Ghr, Il13, Il6, Nlk	mmu-let-7a, mmu-let-7b, mmu-let-7c, mmu-let-7e, mmu-let-7f, mmu-miR-98
GO:0046426	negative regulation of JAK-STAT cascade	Hmga2, Socs1	mmu-let-7a, mmu-let-7b, mmu-let-7c, mmu-let-7e, mmu-let-7f, mmu-miR-98
GO:0046470	phosphatidylcholine metabolic process	Agpat6, Pcyt1b, Pla2g15	mmu-let-7a, mmu-let-7b, mmu-let-7c, mmu-let-7e, mmu-let-7f, mmu-miR-98
GO:0046546	development of primary male sexual characteristics	Acvr2a, Bcl2l1, Ccnd1, Esr2, Fndc3a, Hmga2, Lgr4, Wnt9b	mmu-let-7a, mmu-let-7b, mmu-let-7c, mmu-let-7e, mmu-let-7f, mmu-miR-98
GO:0046619	optic placode formation involved in camera-type eye formation	Frs2	mmu-let-7a, mmu-let-7b, mmu-let-7c, mmu-let-7e, mmu-let-7f, mmu-miR-98
GO:0046666	retinal cell programmed cell death	Fasl, Slc4a7	mmu-let-7a, mmu-let-7b, mmu-let-7c, mmu-let-7e, mmu-let-7f, mmu-miR-98
GO:0046777	protein autophosphorylation	Acvr1b, C230081A13Rik, Dyrk1a, Epha4, Igf1r, Insr, Map3k1, Nlk, Pak1, Slk, Tgfbr1, Tnik, Ulk2	mmu-let-7a, mmu-let-7b, mmu-let-7c, mmu-let-7e, mmu-let-7f, mmu-miR-98
GO:0046782	regulation of viral transcription	Hmga2, Map3k1, Tarbp2	mmu-let-7a, mmu-let-7b, mmu-let-7c, mmu-let-7e, mmu-let-7f, mmu-miR-98
GO:0046784	intronless viral mRNA export from host nucleus	Thoc1, Thoc2	mmu-let-7a, mmu-let-7b, mmu-let-7c, mmu-let-7e, mmu-let-7f, mmu-miR-98
GO:0046898	response to cycloheximide	Bcl2l1, Ghr	mmu-let-7a, mmu-let-7b, mmu-let-7c, mmu-let-7e, mmu-let-7f, mmu-miR-98

GO:0046907	intracellular transport	Abcg1, Ap1s1, Apba1, Bcap29, Bcl2l1, Cask, Cry2, Eda, Ergic1, Gga3, Gipc1, Golga7, Grik2, Hook1, Il6, Kpna1, Nefm, Pacs2, Sec16b, Snx16, Stx17, Stx3, Tgfbr1, Thoc1, Thoc2, Timm17b, Trappc1, Tsc1, Vps26b, Xpot, Zc3h3	mmu-let-7a, mmu-let-7b, mmu-let-7c, mmu-let-7e, mmu-let-7f, mmu-miR-98
GO:0046928	regulation of neurotransmitter secretion	Kcnc3, Kcnc4, Ngf, Slc30a1	mmu-let-7a, mmu-let-7b, mmu-let-7c, mmu-let-7e, mmu-let-7f, mmu-miR-98
GO:0048013	ephrin receptor signaling pathway	Crk, Epha3, Epha4	mmu-let-7a, mmu-let-7b, mmu-let-7c, mmu-let-7e, mmu-let-7f, mmu-miR-98
GO:0048208	COPII vesicle coating	Sec16b	mmu-let-7a, mmu-let-7b, mmu-let-7c, mmu-let-7e, mmu-let-7f, mmu-miR-98
GO:0048332	mesoderm morphogenesis	Chrd, Hand1, Hmga2, Tcf7l1, Wnt1	mmu-let-7a, mmu-let-7b, mmu-let-7c, mmu-let-7e, mmu-let-7f, mmu-miR-98
GO:0048468	cell development	Acta1, Agpat6, AI464131, C230081A13Rik, Ccr7, Col4a1, Col4a5, Dmd, Epha3, Esr2, Ezh2, Fasl, Gas7, Gdf6, Igf1r, Il6, Lmx1a, Mapk8, Nefm, Ngf, Pak1, Pbx1, Pbx3, Pdgfb, Rb1, Rbm38, Sall3, Skil, Slc4a7, Spnb4, Stx3, Tnik, Unc5a, Utrn, Wnt9b	mmu-let-7a, mmu-let-7b, mmu-let-7c, mmu-let-7e, mmu-let-7f, mmu-miR-98
GO:0048496	maintenance of organ identity	Iqcb1, Nphp3	mmu-let-7a, mmu-let-7b, mmu-let-7c, mmu-let-7e, mmu-let-7f, mmu-miR-98
GO:0048518	positive regulation of biological process	Abcg1, Adipor2, Atg4b, Egln2, Klhl6, Klkb1, Masp1, Nat8l, P2rx1, Polr3d, Scn5a, Tarbp2, Tob2	mmu-let-7a, mmu-let-7b, mmu-let-7c, mmu-let-7e, mmu-let-7f, mmu-miR-98
GO:0048585	negative regulation of response to stimulus	Cask, Cd276, Col3a1, Epha4, Il10, Masp1, Ppara, Tarbp2, Tnfrsf1b	mmu-let-7a, mmu-let-7b, mmu-let-7c, mmu-let-7e, mmu-let-7f, mmu-miR-98

GO:0048589	developmental growth	Acvr1c, Adrb2, Bin3, Esrrb, Insr, Mll2, Ngf, Prlr, Rdh10, Sall4, Tbx5, Thbs1	mmu-let-7a, mmu-let-7b, mmu-let-7c, mmu-let-7e, mmu-let-7f, mmu-miR-98
GO:0048598	embryonic morphogenesis	Dlc1, Edn1, Esrrb, Fgf4, Il10, Mdfi, Mycn, Pbx1, Pbx2, Prrx1, Rdh10, Rspo2, Sall4, Sema4c, Tbx5, Tgfbr1, Tsc1, Wnt1, Wnt9a, Wnt9b, Zbtb16	mmu-let-7a, mmu-let-7b, mmu-let-7c, mmu-let-7e, mmu-let-7f, mmu-miR-98
GO:0048608	reproductive structure development	Acvr2a, Bcl2l1, Ccnd1, Esr2, Fndc3a, Frs2, Hmga2, Igf1r, Pcyt1b, Prlr, Rdh10, Wnt9b	mmu-let-7a, mmu-let-7b, mmu-let-7c, mmu-let-7e, mmu-let-7f, mmu-miR-98
GO:0048633	positive regulation of skeletal muscle tissue growth	Adrb2	mmu-let-7a, mmu-let-7b, mmu-let-7c, mmu-let-7e, mmu-let-7f, mmu-miR-98
GO:0048640	negative regulation of developmental growth	Ntn1, Sema4f, Ulk2	mmu-let-7a, mmu-let-7b, mmu-let-7c, mmu-let-7e, mmu-let-7f, mmu-miR-98
GO:0048699	generation of neurons	Esr2, Ezh2, Frs2, Hmga2, Mycn, Tcf7l1	mmu-let-7a, mmu-let-7b, mmu-let-7c, mmu-let-7e, mmu-let-7f, mmu-miR-98
GO:0048701	embryonic cranial skeleton morphogenesis	Prrx1, Rdh10, Tgfbr1, Wnt9b	mmu-let-7a, mmu-let-7b, mmu-let-7c, mmu-let-7e, mmu-let-7f, mmu-miR-98
GO:0048705	skeletal system morphogenesis	Fgf4, Mdfi, Mycn, Ppargc1b, Prrx1, Rdh10, Tgfbr1, Thbs1, Wnt9a, Wnt9b	mmu-let-7a, mmu-let-7b, mmu-let-7c, mmu-let-7e, mmu-let-7f, mmu-miR-98
GO:0048706	embryonic skeletal system development	Acvr2a, Hoxd1, Mdfi, Mycn, Pbx1, Prrx1, Rdh10, Tgfbr1, Wnt9a, Wnt9b	mmu-let-7a, mmu-let-7b, mmu-let-7c, mmu-let-7e, mmu-let-7f, mmu-miR-98
GO:0048710	regulation of astrocyte differentiation	Epha4, Hmga2, Mycn	mmu-let-7a, mmu-let-7b, mmu-let-7c, mmu-let-7e, mmu-let-7f, mmu-miR-98
GO:0048747	muscle fiber development	Acta1, AI464131, Col4a1, Col4a5, Pak1, Rbm38, Utrn	mmu-let-7a, mmu-let-7b, mmu-let-7c, mmu-let-7e, mmu-let-7f, mmu-miR-98

GO:0048754	branching morphogenesis of a tube	Edn1, Lgr4, Mycn, Pak1, Pbx1, Plxnd1, Rdh10, Rspo2, Wnt1, Wnt9b	mmu-let-7a, mmu-let-7b, mmu-let-7c, mmu-let-7e, mmu-let-7f, mmu-miR-98
GO:0048864	stem cell development	Esrrb, Fgf4, Hmga2, Lin28a, Nkap, Tcf7l1	mmu-let-7a, mmu-let-7b, mmu-let-7c, mmu-let-7e, mmu-let-7f, mmu-miR-98
GO:0050657	nucleic acid transport	Ahctf1, Ddx19a, Khsrp, Nxt2, Senp2, Thoc1, Thoc2, Tsc1, Xpot, Zc3h3	mmu-let-7a, mmu-let-7b, mmu-let-7c, mmu-let-7e, mmu-let-7f, mmu-miR-98
GO:0050670	regulation of lymphocyte proliferation	Casp3, Ccr7, Cd276, Cd86, Il10, Il13, Il6, Prdm1, Vav3	mmu-let-7a, mmu-let-7b, mmu-let-7c, mmu-let-7e, mmu-let-7f, mmu-miR-98
GO:0050679	positive regulation of epithelial cell proliferation	Ccnd1, Ccnd2, Foxp2, Il6, Pdgfb, Rictor, Tnfaip3, Vash2	mmu-let-7a, mmu-let-7b, mmu-let-7c, mmu-let-7e, mmu-let-7f, mmu-miR-98
GO:0050728	negative regulation of inflammatory response	Adrb2, Cd276, Il10, Tnfaip3, Tnfrsf1b	mmu-let-7a, mmu-let-7b, mmu-let-7c, mmu-let-7e, mmu-let-7f, mmu-miR-98
GO:0050771	negative regulation of axonogenesis	Epha4, Ntn1, Rufy3, Sema4f, Ulk2	mmu-let-7a, mmu-let-7b, mmu-let-7c, mmu-let-7e, mmu-let-7f, mmu-miR-98
GO:0050864	regulation of B cell activation	Casp3, Il10, Il13, Il6, Prdm1, Tnfaip3, Vav3	mmu-let-7a, mmu-let-7b, mmu-let-7c, mmu-let-7e, mmu-let-7f, mmu-miR-98
GO:0050867	positive regulation of cell activation	Ccr7, Cd276, Cd86, Il13, Il6, Nkap, Pldn, Prdm1, Thbs1, Vav3	mmu-let-7a, mmu-let-7b, mmu-let-7c, mmu-let-7e, mmu-let-7f, mmu-miR-98
GO:0050873	brown fat cell differentiation	Adrb2, Adrb3, Aldh6a1, Ero1l	mmu-let-7a, mmu-let-7b, mmu-let-7c, mmu-let-7e, mmu-let-7f, mmu-miR-98
GO:0050878	regulation of body fluid levels	Adrb2, Agpat6, Aqp4, Ccnd1, Edn1, Klkb1, P2rx1, Pdgfb, Pldn, Prlr, Thbs1	mmu-let-7a, mmu-let-7b, mmu-let-7c, mmu-let-7e, mmu-let-7f, mmu-miR-98

GO:0051017	actin filament bundle assembly	Dlc1, Gas7, Map3k1, Pak1, Ppm1f, Tsc1	mmu-let-7a, mmu-let-7b, mmu-let-7c, mmu-let-7e, mmu-let-7f, mmu-miR-98
GO:0051028	mRNA transport	Ahctf1, Ddx19a, Khsrp, Nxt2, Senp2, Thoc1, Thoc2, Zc3h3	mmu-let-7a, mmu-let-7b, mmu-let-7c, mmu-let-7e, mmu-let-7f, mmu-miR-98
GO:0051056	regulation of small GTPase mediated signal transduction	Arhgef15, Arpp19, Ccr7, Crk, Dlc1, Epha3, Epha4, Obscn, Plekhg6, Psd3, Rasgrp1, Rictor, Tsc1, Vav3	mmu-let-7a, mmu-let-7b, mmu-let-7c, mmu-let-7e, mmu-let-7f, mmu-miR-98
GO:0051091	positive regulation of sequence-specific DNA binding transcription factor activity	Eda, Esr2, Hmga2, Il10, Il6, Mtpn, Ngf, Ppargc1a, Tgfbr3, Wnt1	mmu-let-7a, mmu-let-7b, mmu-let-7c, mmu-let-7e, mmu-let-7f, mmu-miR-98
GO:0051093	negative regulation of developmental process	Arhgef15, Col4a2, Fasl, Gtf2i, Il6, Tbx5, Thbs1	mmu-let-7a, mmu-let-7b, mmu-let-7c, mmu-let-7e, mmu-let-7f, mmu-miR-98
GO:0051094	positive regulation of developmental process	Acvr1b, Acvr2a, Adrb2, Ccr7, Cd276, Edn1, Fndc3b, Frs2, Gdf6, Hmga1, Hmga2, Il6, Insr, Lgr4, Lin28a, Mapk8, Msr1, Ngf, Nkap, Ntn1, Ppargc1b, Prdm1, Rb1, Skil, Tbx5, Thbs1, Vash2, Wnt1, Wnt9a, Zbtb16	mmu-let-7a, mmu-let-7b, mmu-let-7c, mmu-let-7e, mmu-let-7f, mmu-miR-98
GO:0051098	regulation of binding	Adrb2, Calm1, Dot1l, Habp4, Hmga2, Mapk8, Mdfi, Ppara, Senp2, Tgfbr1, Tgfbr3, Zfp462	mmu-let-7a, mmu-let-7b, mmu-let-7c, mmu-let-7e, mmu-let-7f, mmu-miR-98
GO:0051129	negative regulation of cellular component organization	Arhgef15, Bcl2l1, Clasp2, Dlc1, Epha4, Map3k1, Mtpn, Nme6, Ntn1, Rdx, Rufy3, Sema4f, Tgfbr3, Ulk2, Ercc4	mmu-let-7a, mmu-let-7b, mmu-let-7c, mmu-let-7e, mmu-let-7f, mmu-miR-98
GO:0051146	striated muscle cell differentiation	Acta1, AI464131, Col4a1, Col4a5, Edn1, Ezh2, Pak1, Rb1, Rbm38, Tbx5, Tsc1, Utrn, Wnt1	mmu-let-7a, mmu-let-7b, mmu-let-7c, mmu-let-7e, mmu-let-7f, mmu-miR-98
GO:0051216	cartilage development	Edn1, Fgf4, Hmga2, Mycn, Prrx1, Thbs1, Wnt9a, Zbtb16	mmu-let-7a, mmu-let-7b, mmu-let-7c, mmu-let-7e, mmu-let-7f, mmu-miR-98

GO:0051236	establishment of RNA localization	Ahctf1, Ddx19a, Khsrp, Nxt2, Senp2, Thoc1, Thoc2, Tsc1, Xpot, Zc3h3	mmu-let-7a, mmu-let-7b, mmu-let-7c, mmu-let-7e, mmu-let-7f, mmu-miR-98
GO:0051239	regulation of multicellular organismal process	Acvr1c, Agpat6, Ghr, Gipc1, Grid2ip, Grik2, Grin2b, Il10, Il13, Kcnc3, Kcnc4, Klkb1, Mgll, Mtpn, Nat8l, P2rx1, Pbx3, Polr3d, Ppm1f, Prkaa2, Rab8b, Scn5a, Slc30a1, Slc6a1, Spnb4, Tia1, Trib2	mmu-let-7a, mmu-let-7b, mmu-let-7c, mmu-let-7e, mmu-let-7f, mmu-miR-98
GO:0051251	positive regulation of lymphocyte activation	Ccr7, Cd276, Cd86, Il13, Il6, Nkap, Pldn, Prdm1, Vav3	mmu-let-7a, mmu-let-7b, mmu-let-7c, mmu-let-7e, mmu-let-7f, mmu-miR-98
GO:0051271	negative regulation of cellular component movement	Chrd, Dlc1, Pdgfb, Ptpru, Rnf20, Tbx5, Tgfbr3, Thbs1, Trib1	mmu-let-7a, mmu-let-7b, mmu-let-7c, mmu-let-7e, mmu-let-7f, mmu-miR-98
GO:0051290	protein heterotetramerization	Insr, Rrm2	mmu-let-7a, mmu-let-7b, mmu-let-7c, mmu-let-7e, mmu-let-7f, mmu-miR-98
GO:0051291	protein heterooligomerization	Braf, Gabra6, Igf1r, P2rx1, Prkaa2, Tsc1	mmu-let-7a, mmu-let-7b, mmu-let-7c, mmu-let-7e, mmu-let-7f, mmu-miR-98
GO:0051302	regulation of cell division	Calm1, Fgf4, Fgf5, Igf1r, Pdgfb, Wnt9b	mmu-let-7a, mmu-let-7b, mmu-let-7c, mmu-let-7e, mmu-let-7f, mmu-miR-98
GO:0051325	interphase	Acvr1b, Arpp19, Bach1, Bcat1, Bcl2l1, Ccnd1, Ccnd2, Ccny, Cdc25a, Dcun1d3, E2f5, E2f6, Ezh2, Hmga2, Lsm11, Phf8, Rb1, Usp47, Wnt9a	mmu-let-7a, mmu-let-7b, mmu-let-7c, mmu-let-7e, mmu-let-7f, mmu-miR-98
GO:0051338	regulation of transferase activity	Braf, Casp3, Ccnd1, Ccnd2, Ccny, Ccr7, Dusp1, Dusp16, Dusp4, Edn1, Epha4, Ercc6, Frs2, Ghr, Igf1r, Il6, Insr, Map3k1, Mdfi, Nlrc5, Pak1, Pdgfb, Ppm1f, Prlr, Rb1, Spred3, Tab2, Tgfbr1, Thbs1, Tnfaip3, Tnik, Trib1, Tsc1, Vav3	mmu-let-7a, mmu-let-7b, mmu-let-7c, mmu-let-7e, mmu-let-7f, mmu-miR-98

GO:0051345	positive regulation of hydrolase activity	Akap6, Arhgap20, Arhgap28, Arhgef15, Ccr7, Dlc1, Edn1, Fasl, Gipc1, Hip1, Mapk8, P2rx1, Plekhg6, Ppargc1b, Ppm1f, Rgs16, Rnf7, Srgap3, Stard13, Tsc1	mmu-let-7a, mmu-let-7b, mmu-let-7c, mmu-let-7e, mmu-let-7f, mmu-miR-98
GO:0051389	inactivation of MAPKK activity	Igf1r	mmu-let-7a, mmu-let-7b, mmu-let-7c, mmu-let-7e, mmu-let-7f, mmu-miR-98
GO:0051402	neuron apoptosis	Bcl2l1, Braf, Casp3, Egln2, Esr2, Fasl, Ghr, Grik2, Il6, Ngf, Ppara, Ppargc1a, Rb1	mmu-let-7a, mmu-let-7b, mmu-let-7c, mmu-let-7e, mmu-let-7f, mmu-miR-98
GO:0051403	stress-activated MAPK cascade	Ccr7, Dusp22, Edn1, Epha4, Ercc6, Grik2, Igf1r, Map3k1, Map4k4, Mapk8, Mdfi, Pak1, Sema4c, Taok1, Tnik, Trib1	mmu-let-7a, mmu-let-7b, mmu-let-7c, mmu-let-7e, mmu-let-7f, mmu-miR-98
GO:0051492	regulation of stress fiber assembly	Dlc1, Pak1, Ppm1f, Tsc1	mmu-let-7a, mmu-let-7b, mmu-let-7c, mmu-let-7e, mmu-let-7f, mmu-miR-98
GO:0051495	positive regulation of cytoskeleton organization	Edn1, Map3k1, Mapk8, Pak1, Ppm1f, Rictor, Wasl	mmu-let-7a, mmu-let-7b, mmu-let-7c, mmu-let-7e, mmu-let-7f, mmu-miR-98
GO:0051603	proteolysis involved in cellular protein catabolic process	Fbxl12, Fbxl14, Map3k1, Rnf20, Uhrf2, Usp12, Usp24, Usp38, Usp47, Wnt1	mmu-let-7a, mmu-let-7b, mmu-let-7c, mmu-let-7e, mmu-let-7f, mmu-miR-98
GO:0051701	interaction with host	Bcl2l1, Insr, Thoc1, Thoc2	mmu-let-7a, mmu-let-7b, mmu-let-7c, mmu-let-7e, mmu-let-7f, mmu-miR-98
GO:0051783	regulation of nuclear division	Edn1, Insr, Nme6, Pdgfb, Rb1	mmu-let-7a, mmu-let-7b, mmu-let-7c, mmu-let-7e, mmu-let-7f, mmu-miR-98
GO:0051894	positive regulation of focal adhesion assembly	Ppm1f, Tsc1	mmu-let-7a, mmu-let-7b, mmu-let-7c, mmu-let-7e, mmu-let-7f, mmu-miR-98

GO:0051897	positive regulation of protein kinase B signaling cascade	AI464131, Igf1r, Il6, Insr, Rictor, Tgfbr1, Thbs1	mmu-let-7a, mmu-let-7b, mmu-let-7c, mmu-let-7e, mmu-let-7f, mmu-miR-98
GO:0051899	membrane depolarization	Adrb2, Edn1, Grik2, Grin2b, Scn5a	mmu-let-7a, mmu-let-7b, mmu-let-7c, mmu-let-7e, mmu-let-7f, mmu-miR-98
GO:0051917	regulation of fibrinolysis	Klkb1, Thbs1	mmu-let-7a, mmu-let-7b, mmu-let-7c, mmu-let-7e, mmu-let-7f, mmu-miR-98
GO:0051930	regulation of sensory perception of pain	Adrb2, Edn1, Il10, Mgll	mmu-let-7a, mmu-let-7b, mmu-let-7c, mmu-let-7e, mmu-let-7f, mmu-miR-98
GO:0051939	gamma-aminobutyric acid import	Slc6a1	mmu-let-7a, mmu-let-7b, mmu-let-7c, mmu-let-7e, mmu-let-7f, mmu-miR-98
GO:0051960	regulation of nervous system development	Arhgef15, Chrd, Epha3, Epha4, Ezh2, Gdf6, Hmga2, Il6, Lin28a, Lingo1, Lmx1a, Mycn, Nefm, Ngf, Ntn1, Pbx1, Rufy3, Sema4f, Skil, Tnik, Ulk2	mmu-let-7a, mmu-let-7b, mmu-let-7c, mmu-let-7e, mmu-let-7f, mmu-miR-98
GO:0051970	negative regulation of transmission of nerve impulse	Grid2ip, Grik2, Mgll, Slc6a1	mmu-let-7a, mmu-let-7b, mmu-let-7c, mmu-let-7e, mmu-let-7f, mmu-miR-98
GO:0060011	Sertoli cell proliferation	Acvr2a, Esr2	mmu-let-7a, mmu-let-7b, mmu-let-7c, mmu-let-7e, mmu-let-7f, mmu-miR-98
GO:0060070	canonical Wnt receptor signaling pathway	Ccnd1, Ccny, Dkk3, Eda, Lgr4, Nphp3, Ptpru, Rspo2, Tcf7l1, Wnt1, Wnt9a, Wnt9b	mmu-let-7a, mmu-let-7b, mmu-let-7c, mmu-let-7e, mmu-let-7f, mmu-miR-98
GO:0060173	limb development	Fgf4, Mycn, Pbx1, Pbx2, Prrx1, Rdh10, Rspo2, Sall3, Sall4, Tbx5, Wnt9a, Zbtb16	mmu-let-7a, mmu-let-7b, mmu-let-7c, mmu-let-7e, mmu-let-7f, mmu-miR-98

GO:0060178	regulation of exocyst localization	Ralb	mmu-let-7a, mmu-let-7b, mmu-let-7c, mmu-let-7e, mmu-let-7f, mmu-miR-98
GO:0060255	regulation of macromolecule metabolic process	Casp3, Ccny, Dlgap1, Dusp1, Dusp16, Dusp4, Epha4, Fnip1, Fnip2, Frs2, Ndst2, Prlr, Smarcad1, Socs1, Spred3, Tab2, Tnik, Ercc4	mmu-let-7a, mmu-let-7b, mmu-let-7c, mmu-let-7e, mmu-let-7f, mmu-miR-98
GO:0060292	long term synaptic depression	Grid2ip, Mgll	mmu-let-7a, mmu-let-7b, mmu-let-7c, mmu-let-7e, mmu-let-7f, mmu-miR-98
GO:0060339	negative regulation of type I interferon-mediated signaling pathway	Nlrc5	mmu-let-7a, mmu-let-7b, mmu-let-7c, mmu-let-7e, mmu-let-7f, mmu-miR-98
GO:0060445	branching involved in salivary gland morphogenesis	Il6, Pdgfb, Plxnd1	mmu-let-7a, mmu-let-7b, mmu-let-7c, mmu-let-7e, mmu-let-7f, mmu-miR-98
GO:0060485	mesenchyme development	Edn1, Hand1, Hmga2, Nrtn, Rbpj, Rdh10, Tbx5, Tgfbr1, Tgfbr3	mmu-let-7a, mmu-let-7b, mmu-let-7c, mmu-let-7e, mmu-let-7f, mmu-miR-98
GO:0060536	cartilage morphogenesis	Hand1, Rspo2	mmu-let-7a, mmu-let-7b, mmu-let-7c, mmu-let-7e, mmu-let-7f, mmu-miR-98
GO:0060537	muscle tissue development	Acta1, Adrb2, AI464131, Col4a1, Col4a5, Dmd, Foxp2, Hand1, Pak1, Rbm38, Skil, Tbx5, Tgfbr3, Tsc1, Utrn, Wnt1	mmu-let-7a, mmu-let-7b, mmu-let-7c, mmu-let-7e, mmu-let-7f, mmu-miR-98
GO:0060548	negative regulation of cell death	Apbb3, Bcl2l1, Braf, Casp3, Ccr7, Dusp1, Edn1, Esr2, Fgf4, Grik2, Hmga2, Igf1r, Il10, Il6, Mapk8, Ngf, Ppargc1a, Prkaa2, Qars, Rnf7, Taf9b, Tgfbr1, Thbs1, Tnfaip3, Usp47, Wnt1, Wnt9a	mmu-let-7a, mmu-let-7b, mmu-let-7c, mmu-let-7e, mmu-let-7f, mmu-miR-98
GO:0060554	induction of necroptosis of activated-T cells	Fasl	mmu-let-7a, mmu-let-7b, mmu-let-7c, mmu-let-7e, mmu-let-7f, mmu-miR-98

GO:0060562	epithelial tube morphogenesis	Dlc1, Hand1, Lgr4, Ntn1, Pbx1, Rdh10, Rspo2, Sall4, Sema4c, Tsc1, Wnt1, Wnt9b	mmu-let-7a, mmu-let-7b, mmu-let-7c, mmu-let-7e, mmu-let-7f, mmu-miR-98
GO:0060664	epithelial cell proliferation involved in salivary gland morphogenesis	Il6, Pdgfb	mmu-let-7a, mmu-let-7b, mmu-let-7c, mmu-let-7e, mmu-let-7f, mmu-miR-98
GO:0060707	trophoblast giant cell differentiation	Hand1, Mdfi	mmu-let-7a, mmu-let-7b, mmu-let-7c, mmu-let-7e, mmu-let-7f, mmu-miR-98
GO:0061025	membrane fusion	Eea1, Pldn, Smcr7l, Snap23, Stx3, Vav3	mmu-let-7a, mmu-let-7b, mmu-let-7c, mmu-let-7e, mmu-let-7f, mmu-miR-98
GO:0061138	morphogenesis of a branching epithelium	Edn1, Frs2, Il10, Lgr4, Pbx1, Rdh10, Rspo2, Wnt1, Wnt9b	mmu-let-7a, mmu-let-7b, mmu-let-7c, mmu-let-7e, mmu-let-7f, mmu-miR-98
GO:0061387	regulation of extent of cell growth	Ngf, Ntn1, Sema4f, Ulk2	mmu-let-7a, mmu-let-7b, mmu-let-7c, mmu-let-7e, mmu-let-7f, mmu-miR-98
GO:0061418	regulation of transcription from RNA polymerase II promoter in response to hypoxia	Bach1	mmu-let-7a, mmu-let-7b, mmu-let-7c, mmu-let-7e, mmu-let-7f, mmu-miR-98
GO:0070085	glycosylation	Acer2, B3galt1, B3gnt7, Galnt1, Galnt2, Gxylt1, Slc35c1, St6gal1, St8sia1, Uggt1	mmu-let-7a, mmu-let-7b, mmu-let-7c, mmu-let-7e, mmu-let-7f, mmu-miR-98
GO:0070208	protein heterotrimerization	Col1a2, Skil	mmu-let-7a, mmu-let-7b, mmu-let-7c, mmu-let-7e, mmu-let-7f, mmu-miR-98

GO:0070302	regulation of stress-activated protein kinase signaling cascade	Ccr7, Dusp22, Edn1, Epha4, Ercc6, Grik2, Igf1r, Map3k1, Map4k4, Mdfi, Pak1, Sema4c, Taok1	mmu-let-7a, mmu-let-7b, mmu-let-7c, mmu-let-7e, mmu-let-7f, mmu-miR-98
GO:0070429	negative regulation of nucleotide-binding oligomerization domain containing 1 signaling pathway	Tnfaip3	mmu-let-7a, mmu-let-7b, mmu-let-7c, mmu-let-7e, mmu-let-7f, mmu-miR-98
GO:0070433	negative regulation of nucleotide-binding oligomerization domain containing 2 signaling pathway	Tnfaip3	mmu-let-7a, mmu-let-7b, mmu-let-7c, mmu-let-7e, mmu-let-7f, mmu-miR-98
GO:0070536	protein K63-linked deubiquitination	Tnfaip3, Yod1	mmu-let-7a, mmu-let-7b, mmu-let-7c, mmu-let-7e, mmu-let-7f, mmu-miR-98
GO:0070647	protein modification by small protein conjugation or removal	1810074P20Rik, Cdc34, Fbxl14, Fbxo30, Klhl9, Map3k1, Rnf20, Rnf7, Senp2, Socs7, Tgfbr1, Tnfaip3, Trib1, Trib2, Trim33, Uhrf2, Usp12, Usp47, Wnt1, Yod1	mmu-let-7a, mmu-let-7b, mmu-let-7c, mmu-let-7e, mmu-let-7f, mmu-miR-98
GO:0070663	regulation of leukocyte proliferation	Casp3, Ccr7, Cd276, Cd86, Il10, Il13, Il6, Prdm1, Vav3	mmu-let-7a, mmu-let-7b, mmu-let-7c, mmu-let-7e, mmu-let-7f, mmu-miR-98
GO:0070828	heterochromatin organization	Hmga1, Hmga2	mmu-let-7a, mmu-let-7b, mmu-let-7c, mmu-let-7e, mmu-let-7f, mmu-miR-98
GO:0070918	production of small RNA involved in gene silencing by RNA	Lin28a, Lin28b, Tarbp2	mmu-let-7a, mmu-let-7b, mmu-let-7c, mmu-let-7e, mmu-let-7f, mmu-miR-98
GO:0071310	cellular response to organic substance	Adipor2, Aqp4, Bcl2l1, Cask, Casp3, Col1a2, Col3a1, Col4a1, Col5a2, Esr2, Fgf4, Fgf5, Frs2, Ghr, Il13, Il6, Insr, Nlrc5, Nr6a1, Osmr, Pak1, Pdgfb, Ppara, Prlr, Rorc, Smarcc1, Socs1, Socs7, Thbs1, Tnfrsf1b, Trim71	mmu-let-7a, mmu-let-7b, mmu-let-7c, mmu-let-7e, mmu-let-7f, mmu-miR-98

GO:0071359	cellular response to dsRNA	Lin28a, Lin28b, Tarbp2	mmu-let-7a, mmu-let-7b, mmu-let-7c, mmu-let-7e, mmu-let-7f, mmu-miR-98
GO:0071425	hemopoietic stem cell proliferation	Nkap, Wnt1	mmu-let-7a, mmu-let-7b, mmu-let-7c, mmu-let-7e, mmu-let-7f, mmu-miR-98
GO:0071478	cellular response to radiation	Bcat1, Bcl2l1, Cdc25a, Hmga2, Usp47	mmu-let-7a, mmu-let-7b, mmu-let-7c, mmu-let-7e, mmu-let-7f, mmu-miR-98
GO:0071495	cellular response to endogenous stimulus	Adipor2, Bcl2l1, Col1a2, Col3a1, Col4a1, Col5a2, Esr2, Fgf4, Fgf5, Frs2, Ghr, Insr, Nr6a1, Pak1, Pdgfb, Ppara, Rorc, Smarcc1, Socs1, Socs7, Thbs1	mmu-let-7a, mmu-let-7b, mmu-let-7c, mmu-let-7e, mmu-let-7f, mmu-miR-98
GO:0071580	regulation of zinc ion transmembrane transport	Slc30a1	mmu-let-7a, mmu-let-7b, mmu-let-7c, mmu-let-7e, mmu-let-7f, mmu-miR-98
GO:0071582	negative regulation of zinc ion transport	Slc30a1	mmu-let-7a, mmu-let-7b, mmu-let-7c, mmu-let-7e, mmu-let-7f, mmu-miR-98
GO:0071584	negative regulation of zinc ion import	Slc30a1	mmu-let-7a, mmu-let-7b, mmu-let-7c, mmu-let-7e, mmu-let-7f, mmu-miR-98
GO:0071774	response to fibroblast growth factor stimulus	Fgf4, Fgf5, Frs2, Pdgfb, Thbs1	mmu-let-7a, mmu-let-7b, mmu-let-7c, mmu-let-7e, mmu-let-7f, mmu-miR-98
GO:0071779	G1/S transition checkpoint	Bcat1, Ccnd1, Rbm38, Wnt9a	mmu-let-7a, mmu-let-7b, mmu-let-7c, mmu-let-7e, mmu-let-7f, mmu-miR-98
GO:0071780	mitotic cell cycle G2/M transition checkpoint	Bcl2l1, Hmga2	mmu-let-7a, mmu-let-7b, mmu-let-7c, mmu-let-7e, mmu-let-7f, mmu-miR-98
GO:0071842	cellular component organization at cellular level	Adamts14, Col1a2, Col3a1, Col5a2, Dmd, Dmp1, Epha4, Igf1r, Il6, Lingo1, Lmx1a, Ngf, Nrtn, Ntn1, Pla2g3, Pxdn, Rab8b, Rufy3, Sema4f, Skil, Spnb4, Stx3, Ulk2, Unc5a	mmu-let-7a, mmu-let-7b, mmu-let-7c, mmu-let-7e, mmu-let-7f, mmu-miR-98

GO:0071844	cellular component assembly at cellular level	Acta1, Acvr1c, Ahctf1, Atg16l1, Atg4b, Bin3, Ccr7, Clp1, Dlgap1, Edn1, Epha3, Hip1, Hmga1, Hmga2, Iqcb1, Limd1, Mapk8, Nap1l1, Nefm, Pdgfb, Pogz, Prlr, Ralb, Rdx, Rictor, Tarbp2, Tgfbr1, Tgfbr3, Tmem231, Vav3, Wasl, Wnt1	mmu-let-7a, mmu-let-7b, mmu-let-7c, mmu-let-7e, mmu-let-7f, mmu-miR-98
GO:0071875	adrenergic receptor signaling pathway	Adrb2, Adrb3	mmu-let-7a, mmu-let-7b, mmu-let-7c, mmu-let-7e, mmu-let-7f, mmu-miR-98
GO:0071901	negative regulation of protein serine/threonine kinase activity	Casp3, Dusp1, Dusp16, Dusp4, Spred3, Tnfaip3	mmu-let-7a, mmu-let-7b, mmu-let-7c, mmu-let-7e, mmu-let-7f, mmu-miR-98
GO:0071902	positive regulation of protein serine/threonine kinase activity	Ccr7, Edn1, Epha4, Ercc6, Frs2, Ghr, Insr, Map3k1, Mdfi, Pak1, Thbs1	mmu-let-7a, mmu-let-7b, mmu-let-7c, mmu-let-7e, mmu-let-7f, mmu-miR-98
GO:0072003	kidney rudiment formation	Wnt9b	mmu-let-7a, mmu-let-7b, mmu-let-7c, mmu-let-7e, mmu-let-7f, mmu-miR-98
GO:0072046	establishment of planar polarity involved in nephron morphogenesis	Wnt9b	mmu-let-7a, mmu-let-7b, mmu-let-7c, mmu-let-7e, mmu-let-7f, mmu-miR-98
GO:0072170	metanephric tubule development	Lgr4, Wnt9b	mmu-let-7a, mmu-let-7b, mmu-let-7c, mmu-let-7e, mmu-let-7f, mmu-miR-98
GO:0072209	metanephric mesangial cell differentiation	Pdgfb	mmu-let-7a, mmu-let-7b, mmu-let-7c, mmu-let-7e, mmu-let-7f, mmu-miR-98
GO:0072210	metanephric nephron development	Lgr4, Pdgfb, Wnt9b	mmu-let-7a, mmu-let-7b, mmu-let-7c, mmu-let-7e, mmu-let-7f, mmu-miR-98

GO:0072255	metanephric glomerular mesangial cell development	Pdgfb	mmu-let-7a, mmu-let-7b, mmu-let-7c, mmu-let-7e, mmu-let-7f, mmu-miR-98
GO:0072363	regulation of glycolysis by positive regulation of transcription from RNA polymerase II promoter	Ppara	mmu-let-7a, mmu-let-7b, mmu-let-7c, mmu-let-7e, mmu-miR-98
GO:0072366	regulation of cellular ketone metabolic process by positive regulation of transcription from RNA polymerase II promoter	Ppara	mmu-let-7a, mmu-let-7b, mmu-let-7c, mmu-let-7e, mmu-miR-98
GO:0072367	regulation of lipid transport by regulation of transcription from RNA polymerase II promoter	Ppara, Srebf2	mmu-let-7a, mmu-let-7b, mmu-let-7c, mmu-let-7e, mmu-let-7f, mmu-miR-98
GO:0072523	purine-containing compound catabolic process	Abcb9, Abcc10, Abcg1, Arhgap20, Arhgap28, Arhgef15, Ccr7, Chd4, Crk, Ddx19a, Dhx57, Dlc1, Epha3, Epha4, Ercc6, Gipc1, Gnal, Lonrf3, Nkiras2, Plekhg6, Ralb, Rasl10b, Rgs16, Rictor, Srgap3, Stard13, Trank1, Tsc1, Vav3	mmu-let-7a, mmu-let-7b, mmu-let-7c, mmu-let-7e, mmu-let-7f, mmu-miR-98
GO:0072573	tolerance induction to lipopolysaccharide	Tnfaip3	mmu-let-7a, mmu-let-7b, mmu-let-7c, mmu-let-7e, mmu-let-7f, mmu-miR-98
GO:0072610	interleukin-12 secretion	Ccr7	mmu-let-7a, mmu-let-7b, mmu-let-7c, mmu-let-7e, mmu-let-7f, mmu-miR-98

GO:0080134	regulation of response to stress	Cask, Dusp22, Edn1, Epha4, Ercc6, Grik2, Hic1, Hmga1, Hmga2, Igf1r, Klkb1, Map3k1, Map4k4, Mdfi, Pak1, Pdgfb, Sema4c, Taok1, Thbs1, Wnt1	mmu-let-7a, mmu-let-7b, mmu-let-7c, mmu-let-7e, mmu-let-7f, mmu-miR-98
GO:0090077	foam cell differentiation	Abcg1, Msr1, Ppara	mmu-let-7a, mmu-let-7b, mmu-let-7c, mmu-let-7e, mmu-let-7f, mmu-miR-98
GO:0090092	regulation of transmembrane receptor protein serine/threonine kinase signaling pathway	Acvr1b, Acvr2a, Chrd, Gdf6, Gipc1, Peg10, Skil, Tgfbr1, Tgfbr3, Thbs1, Trim33, Wnt1	mmu-let-7a, mmu-let-7b, mmu-let-7c, mmu-let-7e, mmu-let-7f, mmu-miR-98
GO:0090104	pancreatic E cell differentiation	Rfx6	mmu-let-7a, mmu-let-7b, mmu-let-7c, mmu-let-7e, mmu-let-7f, mmu-miR-98
GO:0090109	regulation of cell-substrate junction assembly	Epha3, Ppm1f, Tsc1	mmu-let-7a, mmu-let-7b, mmu-let-7c, mmu-let-7e, mmu-let-7f, mmu-miR-98
GO:0090285	negative regulation of protein glycosylation in Golgi	Acer2	mmu-let-7a, mmu-let-7b, mmu-let-7c, mmu-let-7e, mmu-let-7f, mmu-miR-98
GO:0090342	regulation of cell aging	Hmga1, Hmga2, Wnt1	mmu-let-7a, mmu-let-7b, mmu-let-7c, mmu-let-7e, mmu-let-7f, mmu-miR-98
GO:0090402	oncogene-induced senescence	Hmga1, Hmga2	mmu-let-7a, mmu-let-7b, mmu-let-7c, mmu-let-7e, mmu-let-7f, mmu-miR-98
GO:0097022	lymphocyte migration into lymph node	Ccr7	mmu-let-7a, mmu-let-7b, mmu-let-7c, mmu-let-7e, mmu-let-7f, mmu-miR-98

GO:0097026	dendritic cell dendrite assembly	Ccr7	mmu-let-7a, mmu-let-7b, mmu-let-7c, mmu-let-7e, mmu-let-7f, mmu-miR-98
GO:2000002	negative regulation of DNA damage checkpoint	Wnt1	mmu-let-7a, mmu-let-7b, mmu-let-7c, mmu-let-7e, mmu-let-7f, mmu-miR-98
GO:2000026	regulation of multicellular organismal development	Acvr1b, Acvr2a, Adrb2, Ccnd1, Ccr7, Cd276, Cd86, Col4a2, Edn1, Fasl, Foxp2, Frs2, Gtf2i, Insr, Lgr4, Mapk8, Nkap, Pdgfb, Plxnd1, Ppargc1b, Prdm1, Prlr, Rb1, Rbm38, Tbx5, Tgfbr1, Tgfbr3, Thbs1, Tnfaip3, Tob2, Vash2, Wnt1, Wnt9a, Wnt9b, Zbtb16	mmu-let-7a, mmu-let-7b, mmu-let-7c, mmu-let-7e, mmu-let-7f, mmu-miR-98
GO:2000036	regulation of stem cell maintenance	Hmga2, Tcf7l1	mmu-let-7a, mmu-let-7b, mmu-let-7c, mmu-let-7e, mmu-let-7f, mmu-miR-98
GO:2000045	regulation of G1/S transition of mitotic cell cycle	Bcat1, Ccnd1, Rbm38, Wnt9a	mmu-let-7a, mmu-let-7b, mmu-let-7c, mmu-let-7e, mmu-let-7f, mmu-miR-98
GO:2000116	regulation of cysteine-type endopeptidase activity	Bcl2l1, Il6, Thbs1, Tnfaip3, Usp47, Wnt9a	mmu-let-7a, mmu-let-7b, mmu-let-7c, mmu-let-7e, mmu-let-7f, mmu-miR-98
GO:2000145	regulation of cell motility	Abi3, Ccr7, Chrd, Dlc1, Edn1, Igf1r, Insr, Map3k1, Mapk8, Myo1f, Ntn1, Pdgfb, Plxnd1, Ppm1f, Ptpru, Rnf20, Tbx5, Tgfbr3, Thbs1, Trib1	mmu-let-7a, mmu-let-7b, mmu-let-7c, mmu-let-7e, mmu-let-7f, mmu-miR-98
GO:2000231	positive regulation of pancreatic stellate cell proliferation	Il13	mmu-let-7a, mmu-let-7b, mmu-let-7c, mmu-let-7e, mmu-let-7f, mmu-miR-98
GO:2000349	negative regulation of CD40 signaling pathway	Tnfaip3	mmu-let-7a, mmu-let-7b, mmu-let-7c, mmu-let-7e, mmu-let-7f, mmu-miR-98

GO:2000377	regulation of reactive oxygen species metabolic process	Insr, Mycn, Pdgfb, Thbs1	mmu-let-7a, mmu-let-7b, mmu-let-7c, mmu-let-7e, mmu-let-7f, mmu-miR-98
GO:2000602	regulation of interphase of mitotic cell cycle	Bcl2l1, Dcun1d3, Hmga2, Rb1, Usp47	mmu-let-7a, mmu-let-7b, mmu-let-7c, mmu-let-7e, mmu-let-7f, mmu-miR-98
GO:2000648	positive regulation of stem cell proliferation	Hmga2, Ngf	mmu-let-7a, mmu-let-7b, mmu-let-7c, mmu-let-7e, mmu-let-7f, mmu-miR-98
GO:2000677	regulation of transcription regulatory region DNA binding	Dot1l, Hmga2, Ppara	mmu-let-7a, mmu-let-7b, mmu-let-7c, mmu-let-7e, mmu-let-7f, mmu-miR-98
GO:2000685	positive regulation of cellular response to X-ray	Hmga2	mmu-let-7a, mmu-let-7b, mmu-let-7c, mmu-let-7e, mmu-let-7f, mmu-miR-98
GO:2000774	positive regulation of cellular senescence	Hmga1, Hmga2	mmu-let-7a, mmu-let-7b, mmu-let-7c, mmu-let-7e, mmu-let-7f, mmu-miR-98
GO:2000812	regulation of barbed-end actin filament capping	Mtpn	mmu-let-7a, mmu-let-7b, mmu-let-7c, mmu-let-7e, mmu-let-7f, mmu-miR-98
GO:2001033	negative regulation of double-strand break repair via nonhomologous end joining	Hmga2	mmu-let-7a, mmu-let-7b, mmu-let-7c, mmu-let-7e, mmu-let-7f, mmu-miR-98
GO:2001056	positive regulation of cysteine-type endopeptidase activity	Dlc1, Fasl, Hip1, P2rx1, Ppm1f, Rnf7	mmu-let-7a, mmu-let-7b, mmu-let-7c, mmu-let-7e, mmu-let-7f, mmu-miR-98

GO:2001108	positive regulation of Rho guanyl-nucleotide exchange factor activity	Epha4	mmu-let-7a, mmu-let-7b, mmu-let-7c, mmu-let-7e, mmu-let-7f, mmu-miR-98
**GO ID (molecular function)**	**Go Term (molecular function GO Term)**	**Gene Symbol (genes enriched by up-regulated miRNAs)**	**Up-regulated miRNAs**
GO:0001047	core promoter binding	E2f2, Esr2, Ezh2, Hmga2, Nlrc5, Rb1, Rbpj	mmu-let-7a, mmu-let-7b, mmu-let-7c, mmu-let-7e, mmu-let-7f, mmu-miR-98
GO:0003676	nucleic acid binding	Aen, Cpeb3, Cpsf4, Ddx19a, Dhx57, Dpf2, Eif2c4, Eif4g2, Gpatch2, Gpatch3, Hdlbp, Igf2bp2, Igf2bp3, Lsm11, Nova1, Nynrin, Ppargc1b, Qars, Rbm38, Rbms2, Rpusd2, Rpusd3, Strbp, Syncrip, Tarbp2, Thoc2, Tia1, Xpot, Zc3h3, Zc3hav1l, Zfp740	mmu-let-7a, mmu-let-7b, mmu-let-7c, mmu-let-7e, mmu-let-7f, mmu-miR-98
GO:0003682	chromatin binding	Cbx2, Cbx5, Ercc6, Ezh2, Foxp2, Hmga2, Nkap, Pbx2, Phf8, Prkaa2, Rbpj, Skil, Smarcc1, Srebf2, Suv39h2, Tcf7l1	mmu-let-7a, mmu-let-7b, mmu-let-7c, mmu-let-7e, mmu-let-7f, mmu-miR-98
GO:0003700	sequence-specific DNA binding transcription factor activity	Ahctf1, Arid3a, Bach1, E2f2, E2f5, E2f6, Esr2, Foxp2, Gabpa, Hic1, Hif3a, Hlf, Hmga1, Hmga2, Lcor, Lmx1a, Meis2, Meis3, Mycn, Nr6a1, Onecut3, Pbx1, Pbx2, Pbx3, Ppara, Prrx1, Rbpj, Rorc, Srebf2, Tbx5, Tcf7l1, Tead3, Tead4, Zbtb16, Zfp354a, Elf4	mmu-let-7a, mmu-let-7b, mmu-let-7c, mmu-let-7e, mmu-let-7f, mmu-miR-98
GO:0003730	mRNA 3'-UTR binding	Igf2bp2, Igf2bp3, Khsrp, Rbm38	mmu-let-7a, mmu-let-7b, mmu-let-7c, mmu-let-7e, mmu-let-7f, mmu-miR-98
GO:0003906	DNA-(apurinic or apyrimidinic site) lyase activity	Hmga1, Hmga2	mmu-let-7a, mmu-let-7b, mmu-let-7c, mmu-let-7e, mmu-let-7f, mmu-miR-98

GO:0003978	UDP-glucose 4-epimerase activity	Gale	mmu-let-7a, mmu-let-7b, mmu-let-7c, mmu-let-7e, mmu-let-7f, mmu-miR-98
GO:0003980	UDP-glucose:glycoprotein glucosyltransferase activity	Uggt1	mmu-let-7a, mmu-let-7b, mmu-let-7c, mmu-let-7e, mmu-let-7f, mmu-miR-98
GO:0004149	dihydrolipoyllysine-residue succinyltransferase activity	Dlst	mmu-let-7a, mmu-let-7b, mmu-let-7c, mmu-let-7e, mmu-let-7f, mmu-miR-98
GO:0004221	ubiquitin thiolesterase activity	Tnfaip3, Usp12, Usp24, Usp32, Usp38, Usp47	mmu-let-7a, mmu-let-7b, mmu-let-7c, mmu-let-7e, mmu-let-7f, mmu-miR-98
GO:0004222	metalloendopeptidase activity	Adamts12, Adamts15, Adamts5, Adamts8, Lmln, Mbtps2, Mmp11	mmu-let-7a, mmu-let-7b, mmu-let-7c, mmu-let-7e, mmu-let-7f, mmu-miR-98
GO:0004305	ethanolamine kinase activity	Etnk1, Etnk2	mmu-let-7a, mmu-let-7b, mmu-let-7c, mmu-let-7e, mmu-let-7f, mmu-miR-98
GO:0004491	methylmalonate-semialdehyde dehydrogenase (acylating) activity	Aldh6a1	mmu-let-7a, mmu-let-7b, mmu-let-7c, mmu-let-7e, mmu-let-7f, mmu-miR-98
GO:0004550	nucleoside diphosphate kinase activity	Nme4, Nme6	mmu-let-7a, mmu-let-7b, mmu-let-7c, mmu-let-7e, mmu-let-7f, mmu-miR-98
GO:0004571	mannosyl-oligosaccharide 1,2-alpha-mannosidase activity	Edem1, Edem3	mmu-let-7a, mmu-let-7b, mmu-let-7c, mmu-let-7e, mmu-let-7f, mmu-miR-98
GO:0004672	protein kinase activity	C230081A13Rik, Ccnd1, Frk, Ikbkap, Scyl3, Styk1, Trib2	mmu-let-7a, mmu-let-7b, mmu-let-7c, mmu-let-7e, mmu-let-7f, mmu-miR-98

GO:0004674	protein serine/threonine kinase activity	Acvr1c, Cask, Dyrk1a, Hmga2, Limk2, Map4k3, Map4k4, Nek3, Obscn, Pak1, Riok3, Sbk1, Slk, Speg, Stk40, Taok1, Tgfbr1, Tgfbr3, Tnik, Trpm6, Ulk2	mmu-let-7a, mmu-let-7b, mmu-let-7c, mmu-let-7e, mmu-let-7f, mmu-miR-98
GO:0004679	AMP-activated protein kinase activity	Prkaa2, Prkab2	mmu-let-7a, mmu-let-7b, mmu-let-7c, mmu-let-7e, mmu-let-7f, mmu-miR-98
GO:0004702	receptor signaling protein serine/threonine kinase activity	Acvr1b, Acvr2a, Braf, Ikbke, Map3k1	mmu-let-7a, mmu-let-7b, mmu-let-7c, mmu-let-7e, mmu-let-7f, mmu-miR-98
GO:0004707	MAP kinase activity	Mapk11, Mapk6, Mapk8, Nlk	mmu-let-7a, mmu-let-7b, mmu-let-7c, mmu-let-7e, mmu-let-7f, mmu-miR-98
GO:0004710	MAPK/ERK kinase kinase activity	Map3k1	mmu-let-7a, mmu-let-7b, mmu-let-7c, mmu-let-7e, mmu-let-7f, mmu-miR-98
GO:0004715	non-membrane spanning protein tyrosine kinase activity	C230081A13Rik, Dyrk1a, Frk, Styk1	mmu-let-7a, mmu-let-7b, mmu-let-7c, mmu-let-7e, mmu-let-7f, mmu-miR-98
GO:0004721	phosphoprotein phosphatase activity	Cdc25a, Dusp16, Dusp22, Pdp2, Ppm1f, Ppp2r2a, Pptc7, Ptprd, Ptpru	mmu-let-7a, mmu-let-7b, mmu-let-7c, mmu-let-7e, mmu-let-7f, mmu-miR-98
GO:0004736	pyruvate carboxylase activity	Pcx	mmu-let-7a, mmu-let-7b, mmu-let-7c, mmu-let-7e, mmu-let-7f, mmu-miR-98
GO:0004819	glutamine-tRNA ligase activity	Qars	mmu-let-7a, mmu-let-7b, mmu-let-7c, mmu-let-7e, mmu-let-7f, mmu-miR-98
GO:0004843	ubiquitin-specific protease activity	Tnfaip3, Usp12, Usp47, Yod1	mmu-let-7a, mmu-let-7b, mmu-let-7c, mmu-let-7e, mmu-let-7f, mmu-miR-98

GO:0004903	growth hormone receptor activity	Ghr	mmu-let-7a, mmu-let-7b, mmu-let-7c, mmu-let-7e, mmu-let-7f, mmu-miR-98
GO:0004924	oncostatin-M receptor activity	Osmr	mmu-let-7a, mmu-let-7b, mmu-let-7c, mmu-let-7e, mmu-let-7f, mmu-miR-98
GO:0004925	prolactin receptor activity	Prlr	mmu-let-7a, mmu-let-7b, mmu-let-7c, mmu-let-7e, mmu-let-7f, mmu-miR-98
GO:0004935	adrenergic receptor activity	Adrb2, Adrb3	mmu-let-7a, mmu-let-7b, mmu-let-7c, mmu-let-7e, mmu-let-7f, mmu-miR-98
GO:0004941	beta2-adrenergic receptor activity	Adrb2	mmu-let-7a, mmu-let-7b, mmu-let-7c, mmu-let-7e, mmu-let-7f, mmu-miR-98
GO:0005004	GPI-linked ephrin receptor activity	Epha3, Epha4	mmu-let-7a, mmu-let-7b, mmu-let-7c, mmu-let-7e, mmu-let-7f, mmu-miR-98
GO:0005024	transforming growth factor beta-activated receptor activity	Acvr1b, Acvr1c, Acvr2a, Tgfbr1	mmu-let-7a, mmu-let-7b, mmu-let-7c, mmu-let-7e, mmu-let-7f, mmu-miR-98
GO:0005104	fibroblast growth factor receptor binding	Fgf4, Fgf5, Frs2	mmu-let-7a, mmu-let-7b, mmu-let-7c, mmu-let-7e, mmu-let-7f, mmu-miR-98
GO:0005114	type II transforming growth factor beta receptor binding	Tgfbr1, Tgfbr3	mmu-let-7a, mmu-let-7b, mmu-let-7c, mmu-let-7e, mmu-let-7f, mmu-miR-98
GO:0005141	interleukin-10 receptor binding	Il10	mmu-let-7a, mmu-let-7b, mmu-let-7c, mmu-let-7e, mmu-let-7f, mmu-miR-98

GO:0005144	interleukin-13 receptor binding	Il13	mmu-let-7a, mmu-let-7b, mmu-let-7c, mmu-let-7e, mmu-let-7f, mmu-miR-98
GO:0005163	nerve growth factor receptor binding	Ngf	mmu-let-7a, mmu-let-7b, mmu-let-7c, mmu-let-7e, mmu-let-7f, mmu-miR-98
GO:0005201	extracellular matrix structural constituent	Col1a2, Col24a1, Col27a1, Col3a1, Col4a1, Col4a2, Col5a2, Pxdn	mmu-let-7a, mmu-let-7b, mmu-let-7c, mmu-let-7e, mmu-let-7f, mmu-miR-98
GO:0005248	voltage-gated sodium channel activity	Scn11a, Scn4b, Scn5a, Scn8a	mmu-let-7a, mmu-let-7b, mmu-let-7c, mmu-let-7e, mmu-let-7f, mmu-miR-98
GO:0005261	cation channel activity	Cacna1d, Grin2b, Kcnc3, Kcnc4, Kcnk18, Kctd21, P2rx1, Scn11a, Scn4b, Scn5a, Scn8a, Trpc7, Trpm6	mmu-let-7a, mmu-let-7b, mmu-let-7c, mmu-let-7e, mmu-let-7f, mmu-miR-98
GO:0005539	glycosaminoglycan binding	Adamts8, Chrd, Gns, Habp4, Impg2, Liph, Rspo2, Tgfbr3, Thbs1	mmu-let-7a, mmu-let-7b, mmu-let-7c, mmu-let-7e, mmu-let-7f, mmu-miR-98
GO:0005544	calcium-dependent phospholipid binding	Syt1, Syt11, Syt2	mmu-let-7a, mmu-let-7b, mmu-let-7c, mmu-let-7e, mmu-let-7f, mmu-miR-98
GO:0008022	protein C-terminus binding	Cask, Cep120, Cep135, Coil, Ercc6, Fign, Hic2, Srebf2, Zbtb16, Ercc4	mmu-let-7a, mmu-let-7b, mmu-let-7c, mmu-let-7e, mmu-let-7f, mmu-miR-98
GO:0008134	transcription factor binding	Cbx5, Chd4, Dot1l, E2f2, E2f5, Foxp2, Gnptab, Hand1, Hif1an, Hmga1, Hmga2, Lcor, Mdfi, Nlk, Pbx1, Pbx2, Ppara, Ppargc1a, Rb1, Rbpj, Tbx5, Tob2, Trib1, Trib2	mmu-let-7a, mmu-let-7b, mmu-let-7c, mmu-let-7e, mmu-let-7f, mmu-miR-98
GO:0008194	UDP-glycosyltransferase activity	B3galt1, B3gat3, B3gnt1, B3gnt7, Chsy3, Galnt1, Galnt2, Gxylt1, Has2, Lalba, Mgat4a, Ugcg, Uggt1	mmu-let-7a, mmu-let-7b, mmu-let-7c, mmu-let-7e, mmu-let-7f, mmu-miR-98

GO:0008233	peptidase activity	Adamts12, Adamts14, Adamts15, Adamts5, Adamts8, Atg4b, Casp3, Cpa4, Cpd, Ddi2, Dpp3, Klkb1, Lmln, Lonrf3, Masp1, Mbtps2, Npepl1, Pappa, Prss22, Senp2, Senp5, Tmprss11f, Tmprss2, Tnfaip3, Tpp1, Trhde, Usp12, Usp24, Usp32, Usp38, Usp47, Yod1, Mmp11	mmu-let-7a, mmu-let-7b, mmu-let-7c, mmu-let-7e, mmu-let-7f, mmu-miR-98
GO:0008234	cysteine-type peptidase activity	Atg4b, Casp3, Senp2, Senp5, Tnfaip3, Usp12, Usp24, Usp32, Usp38, Usp47, Yod1	mmu-let-7a, mmu-let-7b, mmu-let-7c, mmu-let-7e, mmu-let-7f, mmu-miR-98
GO:0008237	metallopeptidase activity	Adamts14, Cpa4, Cpd, Dpp3, Npepl1, Pappa, Trhde	mmu-let-7a, mmu-let-7b, mmu-let-7c, mmu-let-7e, mmu-let-7f, mmu-miR-98
GO:0008238	exopeptidase activity	Cpa4, Cpd, Dpp3, Npepl1, Tpp1, Trhde	mmu-let-7a, mmu-let-7b, mmu-let-7c, mmu-let-7e, mmu-let-7f, mmu-miR-98
GO:0008330	protein tyrosine/threonine phosphatase activity	Dusp1, Dusp4	mmu-let-7a, mmu-let-7b, mmu-let-7c, mmu-let-7e, mmu-let-7f, mmu-miR-98
GO:0008460	dTDP-glucose 4,6-dehydratase activity	Tgds	mmu-let-7a, mmu-let-7b, mmu-let-7c, mmu-let-7e, mmu-let-7f, mmu-miR-98
GO:0008508	bile acid:sodium symporter activity	Slc10a6, Slc10a7	mmu-let-7a, mmu-let-7b, mmu-let-7c, mmu-let-7e, mmu-let-7f, mmu-miR-98
GO:0008510	sodium:bicarbonate symporter activity	Slc4a4	mmu-let-7a, mmu-let-7b, mmu-let-7c, mmu-let-7e, mmu-let-7f, mmu-miR-98
GO:0008523	sodium-dependent multivitamin transmembrane transporter activity	Slc5a6	mmu-let-7a, mmu-let-7b, mmu-let-7c, mmu-let-7e, mmu-let-7f, mmu-miR-98

GO:0008532	N-acetyllactosaminide beta-1,3-N-acetylglucosaminyltransferase activity	B3gnt1	mmu-let-7a, mmu-let-7b, mmu-let-7c, mmu-let-7e, mmu-let-7f, mmu-miR-98
GO:0008780	acyl-[acyl-carrier-protein]-UDP-N-acetylglucosamine O-acyltransferase activity	Agpat6	mmu-let-7a, mmu-let-7b, mmu-let-7c, mmu-let-7e, mmu-let-7f, mmu-miR-98
GO:0015016	[heparan sulfate]-glucosamine N-sulfotransferase activity	Ndst2, Ndst3	mmu-let-7a, mmu-let-7b, mmu-let-7c, mmu-let-7e, mmu-let-7f, mmu-miR-98
GO:0015026	coreceptor activity	Acvr2a, Igsf1, Tgfbr3	mmu-let-7a, mmu-let-7b, mmu-let-7c, mmu-let-7e, mmu-let-7f, mmu-miR-98
GO:0015052	beta3-adrenergic receptor activity	Adrb3	mmu-let-7a, mmu-let-7b, mmu-let-7c, mmu-let-7e, mmu-let-7f, mmu-miR-98
GO:0015068	glycine amidinotransferase activity	Gatm	mmu-let-7a, mmu-let-7b, mmu-let-7c, mmu-let-7e, mmu-let-7f, mmu-miR-98
GO:0015291	secondary active transmembrane transporter activity	Slc10a6, Slc10a7, Slc16a14, Slc16a9, Slc24a6, Slc25a18, Slc26a9, Slc4a4, Slc5a6, Slc6a1, Slc8a2	mmu-let-7a, mmu-let-7b, mmu-let-7c, mmu-let-7e, mmu-let-7f, mmu-miR-98
GO:0015294	solute:cation symporter activity	Slc10a6, Slc10a7, Slc4a4, Slc5a6, Slc6a1	mmu-let-7a, mmu-let-7b, mmu-let-7c, mmu-let-7e, mmu-let-7f, mmu-miR-98
GO:0016278	lysine N-methyltransferase activity	Dot1l, Ezh2, Mll2, Suv39h2	mmu-let-7a, mmu-let-7b, mmu-let-7c, mmu-let-7e, mmu-let-7f, mmu-miR-98

GO:0016298	lipase activity	Dagla, Liph, Mgll, Napepld, Pla2g15, Pld3	mmu-let-7a, mmu-let-7b, mmu-let-7c, mmu-let-7e, mmu-let-7f, mmu-miR-98
GO:0016361	activin receptor activity, type I	Acvr1b, Acvr1c	mmu-let-7a, mmu-let-7b, mmu-let-7c, mmu-let-7e, mmu-let-7f, mmu-miR-98
GO:0016362	activin receptor activity, type II	Acvr2a	mmu-let-7a, mmu-let-7b, mmu-let-7c, mmu-let-7e, mmu-let-7f, mmu-miR-98
GO:0016538	cyclin-dependent protein kinase regulator activity	Casp3, Ccnd1, Ccny	mmu-let-7a, mmu-let-7b, mmu-let-7c, mmu-let-7e, mmu-let-7f, mmu-miR-98
GO:0016706	oxidoreductase activity, acting on paired donors, with incorporation or reduction of molecular oxygen, 2-oxoglutarate as one donor, and incorporation of one atom each of oxygen into both donors	Egln2, Hif1an, P4ha2, Phf8	mmu-let-7a, mmu-let-7b, mmu-let-7c, mmu-let-7e, mmu-let-7f, mmu-miR-98
GO:0016740	transferase activity	A130022J15Rik, Agpat6, B3galt1, B3gat3, B3gnt1, B3gnt7, Bcat1, Chsy3, Dlst, Dot1l, Elovl4, Ezh2, Galnt1, Galnt2, Gatm, Gxylt1, Has2, Lalba, Lipt2, Mgat4a, Mll2, Naa30, Nat8l, Ndst2, Ndst3, Piga, Pla2g15, St6gal1, St8sia1, Suv39h2, Ugcg, Uggt1	mmu-let-7a, mmu-let-7b, mmu-let-7c, mmu-let-7e, mmu-let-7f, mmu-miR-98
GO:0016758	transferase activity, transferring hexosyl groups	B3galt1, B3gat3, B3gnt1, B3gnt7, Chsy3, Galnt1, Galnt2, Has2, Lalba, Mgat4a	mmu-let-7a, mmu-let-7b, mmu-let-7c, mmu-let-7e, mmu-let-7f, mmu-miR-98

GO:0016788	hydrolase activity, acting on ester bonds	Acot11, Aen, Aldh6a1, Cdc25a, Dusp1, Dusp16, Dusp22, Dusp4, Gdpd1, Gns, Nceh1, Pdp2, Ppapdc1b, Ppm1f, Ppme1, Ppp2r2a, Pptc7, Ptprd, Ptpru, Slk, Tnfaip3, Usp12, Usp24, Usp32, Usp38, Usp47, Ercc4	mmu-let-7a, mmu-let-7b, mmu-let-7c, mmu-let-7e, mmu-let-7f, mmu-miR-98
GO:0016866	intramolecular transferase activity	Pgm2l1, Rpusd2, Rpusd3	mmu-let-7a, mmu-let-7b, mmu-let-7c, mmu-let-7e, mmu-let-7f, mmu-miR-98
GO:0017017	MAP kinase tyrosine/serine/threonine phosphatase activity	Dusp1, Dusp16, Dusp4	mmu-let-7a, mmu-let-7b, mmu-let-7c, mmu-let-7e, mmu-let-7f, mmu-miR-98
GO:0017188	aspartate N-acetyltransferase activity	Nat8l	mmu-let-7a, mmu-let-7b, mmu-let-7c, mmu-let-7e, mmu-let-7f, mmu-miR-98
GO:0018024	histone-lysine N-methyltransferase activity	Dot1l, Ezh2, Mll2, Suv39h2	mmu-let-7a, mmu-let-7b, mmu-let-7c, mmu-let-7e, mmu-let-7f, mmu-miR-98
GO:0018478	malonate-semialdehyde dehydrogenase (acetylating) activity	Aldh6a1	mmu-let-7a, mmu-let-7b, mmu-let-7c, mmu-let-7e, mmu-let-7f, mmu-miR-98
GO:0019199	transmembrane receptor protein kinase activity	Acvr1b, Acvr1c, Acvr2a, Epha3, Epha4, Igf1r, Insr, Tgfbr1, Tgfbr3	mmu-let-7a, mmu-let-7b, mmu-let-7c, mmu-let-7e, mmu-let-7f, mmu-miR-98
GO:0019210	kinase inhibitor activity	Casp3, Socs1, Trib1, Trib2	mmu-let-7a, mmu-let-7b, mmu-let-7c, mmu-let-7e, mmu-let-7f, mmu-miR-98
GO:0019826	oxygen sensor activity	Egln2	mmu-let-7a, mmu-let-7b, mmu-let-7c, mmu-let-7e, mmu-let-7f, mmu-miR-98
GO:0019838	growth factor binding	Acvr2a, Ghr, Igf1r, Insr, Osmr, Thbs1	mmu-let-7a, mmu-let-7b, mmu-let-7c, mmu-let-7e, mmu-let-7f, mmu-miR-98

GO:0019901	protein kinase binding	Bcl2l1, Ccnd1, Ccnd2, Ccny, Cdc25a, Ghr, Gtf2i, Insr, Mapk6, Pak1, Ppme1, Prkab2, Prlr, Qars, Rab11fip2, Socs1, Taok1	mmu-let-7a, mmu-let-7b, mmu-let-7c, mmu-let-7e, mmu-let-7f, mmu-miR-98
GO:0022851	GABA-gated chloride ion channel activity	Gabra6	mmu-let-7a, mmu-let-7b, mmu-let-7c, mmu-let-7e, mmu-let-7f, mmu-miR-98
GO:0030165	PDZ domain binding	Acvr2a, Apba1, Cask, Dmd, Gipc1, Grik2, Tgfbr3	mmu-let-7a, mmu-let-7b, mmu-let-7c, mmu-let-7e, mmu-let-7f, mmu-miR-98
GO:0030306	ADP-ribosylation factor binding	Gga3, Rab11fip4	mmu-let-7a, mmu-let-7b, mmu-let-7c, mmu-let-7e, mmu-let-7f, mmu-miR-98
GO:0030338	CMP-N-acetylneuraminate monooxygenase activity	Cmah	mmu-let-7a, mmu-let-7b, mmu-let-7c, mmu-let-7e, mmu-let-7f, mmu-miR-98
GO:0030506	ankyrin binding	Obscn, Spnb4	mmu-let-7a, mmu-let-7b, mmu-let-7c, mmu-let-7e, mmu-let-7f, mmu-miR-98
GO:0030674	protein binding, bridging	Cbx5, Col1a2, Crk, Frs2, Lor, Prkaa2	mmu-let-7a, mmu-let-7b, mmu-let-7c, mmu-let-7e, mmu-let-7f, mmu-miR-98
GO:0030695	GTPase regulator activity	Arhgap20, Arhgap28, Arhgef15, Dlc1, Gipc1, Map4k3, Map4k4, Obscn, Plekhg6, Psd3, Rasgrp1, Rgs16, Srgap3, Stard13, Tnik, Tsc1, Vav3, Wasl	mmu-let-7a, mmu-let-7b, mmu-let-7c, mmu-let-7e, mmu-let-7f, mmu-miR-98
GO:0031432	titin binding	Calm1, Obscn	mmu-let-7a, mmu-let-7b, mmu-let-7c, mmu-let-7e, mmu-let-7f, mmu-miR-98

GO:0031434	mitogen-activated protein kinase kinase binding	Braf, Map3k1, Trib1	mmu-let-7a, mmu-let-7b, mmu-let-7c, mmu-let-7e, mmu-let-7f, mmu-miR-98
GO:0031624	ubiquitin conjugating enzyme binding	Grik2, Ppara	mmu-let-7a, mmu-let-7b, mmu-let-7c, mmu-let-7e, mmu-let-7f, mmu-miR-98
GO:0031625	ubiquitin protein ligase binding	Acvr1b, Grik2, Nlk, Rb1, Rnf20, Tgfbr1, Trib1, Trib2	mmu-let-7a, mmu-let-7b, mmu-let-7c, mmu-let-7e, mmu-let-7f, mmu-miR-98
GO:0031699	beta-3 adrenergic receptor binding	Adrb3	mmu-let-7a, mmu-let-7b, mmu-let-7c, mmu-let-7e, mmu-let-7f, mmu-miR-98
GO:0031707	endothelin A receptor binding	Edn1	mmu-let-7a, mmu-let-7b, mmu-let-7c, mmu-let-7e, mmu-let-7f, mmu-miR-98
GO:0031994	insulin-like growth factor I binding	Igf1r, Insr	mmu-let-7a, mmu-let-7b, mmu-let-7c, mmu-let-7e, mmu-let-7f, mmu-miR-98
GO:0031997	N-terminal myristoylation domain binding	Calm1	mmu-let-7a, mmu-let-7b, mmu-let-7c, mmu-let-7e, mmu-let-7f, mmu-miR-98
GO:0033781	cholesterol 24-hydroxylase activity	Cyp46a1	mmu-let-7a, mmu-let-7b, mmu-let-7c, mmu-let-7e, mmu-let-7f, mmu-miR-98
GO:0033819	lipoyl(octanoyl) transferase activity	Lipt2	mmu-let-7a, mmu-let-7b, mmu-let-7c, mmu-let-7e, mmu-let-7f, mmu-miR-98
GO:0034041	sterol-transporting ATPase activity	Abcg1	mmu-let-7a, mmu-let-7b, mmu-let-7c, mmu-let-7e, mmu-let-7f, mmu-miR-98
GO:0034711	inhibin binding	Acvr1b	mmu-let-7a, mmu-let-7b, mmu-let-7c, mmu-let-7e, mmu-let-7f, mmu-miR-98

GO:0035064	methylated histone residue binding	Cbx2, Cbx5, Ing3, Phf8	mmu-let-7a, mmu-let-7b, mmu-let-7c, mmu-let-7e, mmu-let-7f, mmu-miR-98
GO:0035500	MH2 domain binding	Hmga2	mmu-let-7a, mmu-let-7b, mmu-let-7c, mmu-let-7e, mmu-let-7f, mmu-miR-98
GO:0035501	MH1 domain binding	Hmga2	mmu-let-7a, mmu-let-7b, mmu-let-7c, mmu-let-7e, mmu-let-7f, mmu-miR-98
GO:0035757	chemokine (C-C motif) ligand 19 binding	Ccr7	mmu-let-7a, mmu-let-7b, mmu-let-7c, mmu-let-7e, mmu-let-7f, mmu-miR-98
GO:0035758	chemokine (C-C motif) ligand 21 binding	Ccr7	mmu-let-7a, mmu-let-7b, mmu-let-7c, mmu-let-7e, mmu-let-7f, mmu-miR-98
GO:0042169	SH2 domain binding	Crk, Dlc1, Ghr, Insr, Nlk	mmu-let-7a, mmu-let-7b, mmu-let-7c, mmu-let-7e, mmu-let-7f, mmu-miR-98
GO:0042301	phosphate ion binding	Nceh1, Sh2b3	mmu-let-7a, mmu-let-7b, mmu-let-7c, mmu-let-7e, mmu-let-7f, mmu-miR-98
GO:0042562	hormone binding	Adipor2, Esr2, Ghr, Igf1r, Insr, Prlr	mmu-let-7a, mmu-let-7b, mmu-let-7c, mmu-let-7e, mmu-let-7f, mmu-miR-98
GO:0042802	identical protein binding	Adipor2, Bcat1, Bcl2l1, Coil, Col1a2, Hook1, Igf1r, Kctd17, Pldn, Prkab2, Snx16, Syt1, Uhrf1bp1	mmu-let-7a, mmu-let-7b, mmu-let-7c, mmu-let-7e, mmu-let-7f, mmu-miR-98
GO:0042803	protein homodimerization activity	Abcb9, Abcg1, Adrb2, Adrb3, Arid3a, Eea1, Foxp2, Gale, Gdf6, Ghr, Gipc1, Grik2, Grpel2, Hand1, Hif1an, Homer2, Ikzf2, Masp1, Mgll, Nr6a1, Pdgfb, Rab11fip2, Rab11fip4, Slk, Tarbp2, Thrsp, Zbtb16	mmu-let-7a, mmu-let-7b, mmu-let-7c, mmu-let-7e, mmu-let-7f, mmu-miR-98

GO:0042826	histone deacetylase binding	Cbx5, Hic1, Mapk8, Prdm1, Uhrf1bp1	mmu-let-7a, mmu-let-7b, mmu-let-7c, mmu-let-7e, mmu-let-7f, mmu-miR-98
GO:0043250	sodium-dependent organic anion transmembrane transporter activity	Slc10a6	mmu-let-7a, mmu-let-7b, mmu-let-7c, mmu-let-7e, mmu-let-7f, mmu-miR-98
GO:0043548	phosphatidylinositol 3-kinase binding	Calm1, Igf1r, Insr	mmu-let-7a, mmu-let-7b, mmu-let-7c, mmu-let-7e, mmu-let-7f, mmu-miR-98
GO:0043559	insulin binding	Igf1r, Insr	mmu-let-7a, mmu-let-7b, mmu-let-7c, mmu-let-7e, mmu-let-7f, mmu-miR-98
GO:0043560	insulin receptor substrate binding	Igf1r, Insr	mmu-let-7a, mmu-let-7b, mmu-let-7c, mmu-let-7e, mmu-let-7f, mmu-miR-98
GO:0043565	sequence-specific DNA binding	Bach1, E2f5, Esr2, Esrrb, Ezh2, Foxp2, Gabpa, Hand1, Hic1, Hlf, Hmga2, Lmx1a, Meis2, Meis3, Nlrc5, Nr6a1, Onecut3, Pbx1, Pbx2, Pbx3, Ppara, Prdm1, Prrx1, Rbpj, Rorc, Sox13, Srebf2, Tbx5, Tcf7l1, Elf4	mmu-let-7a, mmu-let-7b, mmu-let-7c, mmu-let-7e, mmu-let-7f, mmu-miR-98
GO:0046332	SMAD binding	Acvr1b, Acvr1c, Col1a2, Col3a1, Col5a2, Hmga2, Skil, Tgfbr1, Tgfbr3, Trim33	mmu-let-7a, mmu-let-7b, mmu-let-7c, mmu-let-7e, mmu-let-7f, mmu-miR-98
GO:0046527	glucosyltransferase activity	Ugcg, Uggt1	mmu-let-7a, mmu-let-7b, mmu-let-7c, mmu-let-7e, mmu-let-7f, mmu-miR-98
GO:0046982	protein heterodimerization activity	Abcg1, Adipor2, Bcl2l1, Braf, Foxp2, Gabpa, Grin2b, Hand1, Homer2, Ikzf2, Limk2, Mapk6, Pbx1, Pdgfb, Ppara, Rragd, Tgfbr1	mmu-let-7a, mmu-let-7b, mmu-let-7c, mmu-let-7e, mmu-let-7f, mmu-miR-98

GO:0047933	glucose-1,6-bisphosphate synthase activity	Pgm2l1	mmu-let-7a, mmu-let-7b, mmu-let-7c, mmu-let-7e, mmu-let-7f, mmu-miR-98
GO:0048027	mRNA 5'-UTR binding	Igf2bp2, Igf2bp3	mmu-let-7a, mmu-let-7b, mmu-let-7c, mmu-let-7e, mmu-let-7f, mmu-miR-98
GO:0048185	activin binding	Acvr1b, Acvr1c, Acvr2a, Tgfbr3	mmu-let-7a, mmu-let-7b, mmu-let-7c, mmu-let-7e, mmu-let-7f, mmu-miR-98
GO:0048186	inhibin beta-A binding	Acvr2a, Igsf1	mmu-let-7a, mmu-let-7b, mmu-let-7c, mmu-let-7e, mmu-let-7f, mmu-miR-98
GO:0048187	inhibin beta-B binding	Acvr2a, Igsf1	mmu-let-7a, mmu-let-7b, mmu-let-7c, mmu-let-7e, mmu-let-7f, mmu-miR-98
GO:0048407	platelet-derived growth factor binding	Col1a2, Col3a1, Col4a1, Pdgfb	mmu-let-7a, mmu-let-7b, mmu-let-7c, mmu-let-7e, mmu-let-7f, mmu-miR-98
GO:0050431	transforming growth factor beta binding	Tgfbr1, Tgfbr3	mmu-let-7a, mmu-let-7b, mmu-let-7c, mmu-let-7e, mmu-let-7f, mmu-miR-98
GO:0051087	chaperone binding	Dlst, Dnajc1, Grpel2, Tsc1	mmu-let-7a, mmu-let-7b, mmu-let-7c, mmu-let-7e, mmu-let-7f, mmu-miR-98
GO:0051379	epinephrine binding	Adrb2, Adrb3	mmu-let-7a, mmu-let-7b, mmu-let-7c, mmu-let-7e, mmu-let-7f, mmu-miR-98
GO:0051380	norepinephrine binding	Adrb2, Adrb3	mmu-let-7a, mmu-let-7b, mmu-let-7c, mmu-let-7e, mmu-let-7f, mmu-miR-98
GO:0051575	5'-deoxyribose-5-phosphate lyase activity	Hmga1, Hmga2	mmu-let-7a, mmu-let-7b, mmu-let-7c, mmu-let-7e, mmu-let-7f, mmu-miR-98

GO:0051722	protein C-terminal methylesterase activity	Ppme1	mmu-let-7a, mmu-let-7b, mmu-let-7c, mmu-let-7e, mmu-let-7f, mmu-miR-98
GO:0051736	ATP-dependent polyribonucleotide 5'-hydroxyl-kinase activity	Clp1	mmu-let-7a, mmu-let-7b, mmu-let-7c, mmu-let-7e, mmu-let-7f, mmu-miR-98
GO:0051787	misfolded protein binding	Dnajb9, Edem1	mmu-let-7a, mmu-let-7b, mmu-let-7c, mmu-let-7e, mmu-let-7f, mmu-miR-98
GO:0055106	ubiquitin-protein ligase regulator activity	Trib1, Trib2	mmu-let-7a, mmu-let-7b, mmu-let-7c, mmu-let-7e, mmu-let-7f, mmu-miR-98
GO:0070123	transforming growth factor beta receptor activity, type III	Tgfbr3	mmu-let-7a, mmu-let-7b, mmu-let-7c, mmu-let-7e, mmu-let-7f, mmu-miR-98
GO:0070215	MDM2 binding	Bcl2l1, Ppara	mmu-let-7a, mmu-let-7b, mmu-let-7c, mmu-let-7e, mmu-let-7f, mmu-miR-98
GO:0070290	NAPE-specific phospholipase D activity	Napepld, Pld3	mmu-let-7a, mmu-let-7b, mmu-let-7c, mmu-let-7e, mmu-let-7f, mmu-miR-98
GO:0070579	methylcytosine dioxygenase activity	Tet2, Tet3	mmu-let-7a, mmu-let-7b, mmu-let-7c, mmu-let-7e, mmu-let-7f, mmu-miR-98
GO:0071209	U7 snRNA binding	Lsm11	mmu-let-7a, mmu-let-7b, mmu-let-7c, mmu-let-7e, mmu-let-7f, mmu-miR-98
GO:0071987	WD40-repeat domain binding	Usp47	mmu-let-7a, mmu-let-7b, mmu-let-7c, mmu-let-7e, mmu-let-7f, mmu-miR-98
GO:0097161	DH domain binding	Epha4	mmu-let-7a, mmu-let-7b, mmu-let-7c, mmu-let-7e, mmu-let-7f, mmu-miR-98

**Down-regulated miRNAs:**			
**GO ID (cellular Component)**	**Go Term (Cellular Component GO Term)**	**Gene Symbol (genes enriched by down-regulated miRNAs)**	**Down-regulated miRNAs**
GO:0001650	fibrillar center	Ube2i	mmu-miR-124, mmu-miR-30e, mmu-miR-384-5p
GO:0001726	ruffle	Frmd4b, Gsn, Lcp1, Myo5a	mmu-miR-124, mmu-miR-26a, mmu-miR-30e, mmu-miR-384-5p
GO:0005622	intracellular	Zfp608, Zfp706, Adcy9, Epha2, Rasd2, Rhog, Ror2, Rras, Sos2, Tbc1d9b	mmu-miR-124, mmu-miR-26a, mmu-miR-30e, mmu-miR-384-5p
GO:0005626	insoluble fraction	Arhgap17, Capn1, Ppp3cb	mmu-miR-124, mmu-miR-26a, mmu-miR-30e, mmu-miR-384-5p
GO:0005635	nuclear envelope	Lass2, Ache, Ankle2, Ednrb, Ern1, Htatip2, Itpr3, Lemd2, Smad1	mmu-miR-124, mmu-miR-26a, mmu-miR-30e, mmu-miR-384-5p
GO:0005637	nuclear inner membrane	Ern1, Lemd2, Smad1	mmu-miR-124, mmu-miR-26a, mmu-miR-30e, mmu-miR-384-5p
GO:0005759	mitochondrial matrix	Acadvl, Acss1, Aldh4a1, Bckdha, Gstk1, Pde2a, Suclg2	mmu-miR-124, mmu-miR-379
GO:0005764	lysosome	Acp2, Cd164, Dram1, Galc, Kif2a, Lmbrd1, Pcyox1, Plekhf1, Prdx6, Rab27a	mmu-miR-124, mmu-miR-26a, mmu-miR-30e, mmu-miR-384-5p
GO:0005773	vacuole	Acp2, Cd164, Dram1, Galc, Kif2a, Lmbrd1, Pcyox1, Plekhf1, Prdx6, Rab27a, Ttc3, Vps35	mmu-miR-124, mmu-miR-26a, mmu-miR-30e, mmu-miR-384-5p
GO:0005788	endoplasmic reticulum lumen	Dnajc1, Edem3, Ache	mmu-miR-124, mmu-miR-30e, mmu-miR-379, mmu-miR-384-5p

GO:0005789	endoplasmic reticulum membrane	Faf2, Dgat2, Cyb5, Ern1, Itpr3, Lman2l, Mtdh, Pemt, Rhbdf1, Ryr1, Erlin1	mmu-miR-124, mmu-miR-26a, mmu-miR-30e, mmu-miR-384-5p
GO:0005792	microsome	Dmd, Dnajc1, Rdh10, Dgat2, Atp1a1, Clip3, Cpt1a, Cyb5, Itpr3, Sc5d, Scd1, Slc27a1, Tmed10	mmu-miR-124, mmu-miR-26a, mmu-miR-30e, mmu-miR-384-5p
GO:0005793	endoplasmic reticulum-Golgi intermediate compartment	Surf4, Tmed1, Tmed10	mmu-miR-124, mmu-miR-26a, mmu-miR-30e, mmu-miR-384-5p
GO:0005811	lipid particle	Faf2, Dgat2, Cav1, Lipe	mmu-miR-124, mmu-miR-30e, mmu-miR-384-5p
GO:0005815	microtubule organizing center	Abca2, Fgfr1op, Cspp1, Dctn4, Flot1, Gnai1, Gnai2, Kif2a, Kif3a, Pan3, Poc1b, Usp2	mmu-miR-124, mmu-miR-30e, mmu-miR-384-5p
GO:0005834	heterotrimeric G-protein complex	Gnal, Gna13, Gnai1, Gnai2, Gng10, Rgs9	mmu-miR-124, mmu-miR-26a, mmu-miR-30e, mmu-miR-384-5p
GO:0005911	cell-cell junction	Dlg5, Myo1e, Arhgap17, Twf1, Ocln, Anxa5, Fzd4, Mtdh, Pcdh1, Tjp2, Abi2, Epb4.1l3	mmu-miR-124, mmu-miR-26a, mmu-miR-30e, mmu-miR-384-5p
GO:0005912	adherens junction	Dlg5, Myo1e, Aif1l, Cav1, Epha2, Sdc4, Abi2	mmu-miR-124, mmu-miR-26a, mmu-miR-30e, mmu-miR-384-5p
GO:0008021	synaptic vesicle	Gria2, Kif3a, Myo5a, Sphk1, Sypl	mmu-miR-124, mmu-miR-30e, mmu-miR-379, mmu-miR-384-5p
GO:0009898	internal side of plasma membrane	Gnal, Gcom1, Gna13, Gnai1, Gnai2, Gng10, Rgs9	mmu-miR-124, mmu-miR-26a, mmu-miR-30e, mmu-miR-384-5p
GO:0012505	endomembrane system	Faf2, Dgat2, Gria2, Abcc4, Cav1, Clip3, Cyb5, Lman2l, Mtdh, Pemt, Qsox1, Rab27a, Rhbdf1, Ryr1, Tmed10, Erlin1	mmu-miR-124, mmu-miR-26a, mmu-miR-30e, mmu-miR-379, mmu-miR-384-5p

GO:0015629	actin cytoskeleton	Myo1e, Twf1, Aif1l, Arpc1b, Dapk1, Gas2l1, Gcom1, Gsn, Lcp1, Myh10, Myo5a, Pgm2, Sept9, Pawr	mmu-miR-124, mmu-miR-26a, mmu-miR-30e, mmu-miR-384-5p
GO:0016020	membrane	Edem3, Rffl, 2610018G03Rik, Cadps, Gas2, Plod3, Rufy1, Sdf2l1, Snap29, Yme1l1, Fkbp3, Sema3a	mmu-miR-124, mmu-miR-26a, mmu-miR-30e, mmu-miR-379, mmu-miR-384-5p
GO:0016323	basolateral plasma membrane	Adam9, Slc22a5, Abcc4, Atp1a1, Slc29a1, Stk39	mmu-miR-124, mmu-miR-26a, mmu-miR-30e, mmu-miR-379, mmu-miR-384-5p
GO:0016324	apical plasma membrane	Slc22a5, Anxa4, Cav1, Dsg2, Mtdh, Rab27a, Slc29a1, Stk39	mmu-miR-124, mmu-miR-26a, mmu-miR-30e, mmu-miR-384-5p
GO:0016529	sarcoplasmic reticulum	Agl, Calu, Casq2, Ryr1	mmu-miR-124, mmu-miR-30e, mmu-miR-384-5p
GO:0019897	extrinsic to plasma membrane	Gnal, Gcom1, Gna13, Gnai1, Gnai2, Gng10, Ppl, Rgs9	mmu-miR-124, mmu-miR-26a, mmu-miR-30e, mmu-miR-384-5p
GO:0030027	lamellipodium	Gsn, Pip5k1a, Sh3rf1, Spata13, Abi2, Ptpn13	mmu-miR-124, mmu-miR-26a, mmu-miR-30e, mmu-miR-384-5p
GO:0030054	cell junction	Homer2, Pcdh8, Ctnnd2, Gria2, Ache, Cacng5, Cadps, Cbln4, Gcom1, Kctd8, Lcp1, Ppl, Snap29, Snta1	mmu-miR-124, mmu-miR-26a, mmu-miR-30e, mmu-miR-379, mmu-miR-384-5p
GO:0030055	cell-substrate junction	Dmd, Aif1l, Cav1, Epha2, Sdc4	mmu-miR-124, mmu-miR-26a, mmu-miR-30e, mmu-miR-384-5p
GO:0030057	desmosome	Dsg2, Pnn	mmu-miR-124, mmu-miR-30e, mmu-miR-384-5p
GO:0030425	dendrite	Homer2, Ctnnd2, Gria2, Ache, Cacng5, Myh10, Numa1, Rab27a, Slc1a4, Ube2i, Abi2, Sema3a	mmu-miR-124, mmu-miR-26a, mmu-miR-30e, mmu-miR-379, mmu-miR-384-5p

GO:0030667	secretory granule membrane	Abcc4, Cav1, Rab27a, Tmed10	mmu-miR-124, mmu-miR-26a, mmu-miR-30e, mmu-miR-379, mmu-miR-384-5p
GO:0031372	UBC13-MMS2 complex	Ube2v2	mmu-miR-124, mmu-miR-30e, mmu-miR-384-5p
GO:0031526	brush border membrane	Slc22a5, Gna13, Pemt	mmu-miR-124, mmu-miR-26a
GO:0032281	alpha-amino-3-hydroxy-5-methyl-4-isoxazolepropionic acid selective glutamate receptor complex	Gria2, Cacng5	mmu-miR-124, mmu-miR-30e, mmu-miR-379, mmu-miR-384-5p
GO:0032587	ruffle membrane	Twf1, Pip5k1a, Spata13	mmu-miR-124, mmu-miR-26a, mmu-miR-30e, mmu-miR-384-5p
GO:0042383	sarcolemma	Dmd, Anxa5, Atp1a1, Flot1, Pemt, Ryr1, Snta1	mmu-miR-124, mmu-miR-30e, mmu-miR-384-5p
GO:0042470	melanosome	Myo5a, Rab27a	mmu-miR-124, mmu-miR-30e, mmu-miR-384-5p
GO:0042588	zymogen granule	Rab3d, Tmed10	mmu-miR-124, mmu-miR-26a, mmu-miR-30e, mmu-miR-384-5p
GO:0042642	actomyosin, myosin complex part	Myo5a	mmu-miR-124, mmu-miR-30e, mmu-miR-384-5p
GO:0042734	presynaptic membrane	Gria2, Ache, Pde2a	mmu-miR-124, mmu-miR-30e, mmu-miR-379, mmu-miR-384-5p
GO:0043034	costamere	Dmd, Fxr1, Sdc4	mmu-miR-124, mmu-miR-30e, mmu-miR-384-5p
GO:0043292	contractile fiber	Dmd, Twf1, Fxr1, Gcom1, Ryr1, Sdc4	mmu-miR-124, mmu-miR-26a, mmu-miR-30e, mmu-miR-384-5p

GO:0044424	intracellular part	Gnal, Fbxo27, Gna13, Gng10, Ppp3cb	mmu-miR-124, mmu-miR-26a, mmu-miR-30e, mmu-miR-384-5p
GO:0045121	membrane raft	Dmd, Clip3, Ednrb, Efnb1, Gnai1, Gnai2, Pde2a, Sdc4, Usp48	mmu-miR-124, mmu-miR-26a, mmu-miR-30e, mmu-miR-379, mmu-miR-384-5p
GO:0045202	synapse	Dmd, Homer2, Pcdh8, Ctnnd2, Gria2, Ache, Cacng5, Cadps, Cbln4, Efnb1, Kctd8, Kif3a, Myh10, Myo5a, Pde2a, Snap29, Snta1, Sphk1, Sypl, Ube2i, Usp48	mmu-miR-124, mmu-miR-26a, mmu-miR-30e, mmu-miR-379, mmu-miR-384-5p
GO:0045211	postsynaptic membrane	Homer2, Pcdh8, Gria2, Ache, Cacng5, Kctd8, Snta1, Usp48	mmu-miR-124, mmu-miR-26a, mmu-miR-30e, mmu-miR-379, mmu-miR-384-5p
GO:0048471	perinuclear region of cytoplasm	Ppm1f, Twf1, Dgat2, Fgfr1op, Kalrn, Ache, Apln, Cav1, Chodl, Gsn, Inf2, Mtdh, Pde2a, Phex, Sept9, Tpd52l2, Usp2	mmu-miR-124, mmu-miR-26a, mmu-miR-30e, mmu-miR-379, mmu-miR-384-5p
GO:0070765	gamma-secretase complex	Tmed10	mmu-miR-124, mmu-miR-26a, mmu-miR-30e, mmu-miR-384-5p
**GO ID (biological process)**	**Go Term (biological process GO Term)**	**Gene Symbol (genes enriched by down-regulated miRNAs)**	**Down-regulated miRNAs**
GO:0000038	very long-chain fatty acid metabolic process	Acadvl, Elovl5, Sphk1	mmu-miR-124, mmu-miR-30e, mmu-miR-384-5p
GO:0000122	negative regulation of transcription from RNA polymerase II promoter	Cbx2, Edn1, Sall4, Bcl6, Aes, Barx2, Cav1, Ednrb, Mtdh, Pde2a, Rarg, Rxra, Snai2, Ube2i, Usp2, Yeats2, Fst	mmu-miR-124, mmu-miR-26a, mmu-miR-30e, mmu-miR-379, mmu-miR-384-5p

GO:0000186	activation of MAPKK activity	Map3k1, Adam9, Gnai2	mmu-miR-124, mmu-miR-26a, mmu-miR-30e, mmu-miR-379, mmu-miR-384-5p
GO:0000904	cell morphogenesis involved in differentiation	Epha2, Fzd4, Plod3, Snai2, Ttc3	mmu-miR-124, mmu-miR-26a, mmu-miR-30e, mmu-miR-384-5p
GO:0001501	skeletal system development	Edn1, Acp2, Aes, Epha2, Ryr1, Snai2, Smad1	mmu-miR-124, mmu-miR-26a, mmu-miR-30e, mmu-miR-379, mmu-miR-384-5p
GO:0001525	angiogenesis	Edn1, Pdcd10, C1galt1, Dll4, Epha2, Gna13, Htatip2, Mtdh, Nr4a1, Ovol2, Sphk1	mmu-miR-124, mmu-miR-26a, mmu-miR-30e, mmu-miR-379, mmu-miR-384-5p
GO:0001655	urogenital system development	Rdh10, Eya1, Myo1e, Anxa4, Bmp6, C1galt1, Kif3a, Rarg, Rxra, Sox8, Nid1	mmu-miR-124, mmu-miR-26a, mmu-miR-30e, mmu-miR-384-5p
GO:0001701	in utero embryonic development	Edn1, Rdh10, Sall4, Myo1e, Tet1, Apba3, Gna13, Kif3a, Myh10, Ovol2, Plod3, Sox8	mmu-miR-124, mmu-miR-26a, mmu-miR-30e, mmu-miR-379, mmu-miR-384-5p
GO:0001755	neural crest cell migration	Ednrb, Efnb1, Ovol2, Sox8	mmu-miR-124, mmu-miR-30e, mmu-miR-384-5p
GO:0001822	kidney development	Rdh10, Eya1, Myo1e, Anxa4, Bmp6, C1galt1, Kif3a, Sox8, Nid1	mmu-miR-124, mmu-miR-26a, mmu-miR-30e, mmu-miR-384-5p
GO:0001932	regulation of protein phosphorylation	Edn1, Map3k1, Tcfap4, Pdcd10, Adam9, Fgfr1op, Akt1s1, Bmp6, Cav1, Clip3, Ednrb, Ern1, Fzd4, Gnai2, Sdc4, Sfrp5, Slc27a1, Sphk1, Trib3	mmu-miR-124, mmu-miR-26a, mmu-miR-30e, mmu-miR-379, mmu-miR-384-5p
GO:0001944	vasculature development	Edn1, Eya1, Myo1e, Pdcd10, Rapgef1, C1galt1, Cav1, Dll4, Epha2, Fzd4, Gna13, Htatip2, Itga7, Mtdh, Nr4a1, Ovol2, Rxra, Sfrp5, Sphk1	mmu-miR-124, mmu-miR-26a, mmu-miR-30e, mmu-miR-379, mmu-miR-384-5p
GO:0001952	regulation of cell-matrix adhesion	Epha3, Ppm1f, Bcl6, Sdc4	mmu-miR-124, mmu-miR-30e, mmu-miR-384-5p

GO:0003018	vascular process in circulatory system	Edn1, Apln, Cav1, Ednrb, Pde2a, Plod3	mmu-miR-124, mmu-miR-30e, mmu-miR-379, mmu-miR-384-5p
GO:0003430	growth plate cartilage chondrocyte growth	Rarg	mmu-miR-124, mmu-miR-30e, mmu-miR-384-5p
GO:0003433	chondrocyte development involved in endochondral bone morphogenesis	Rarg	mmu-miR-124, mmu-miR-30e, mmu-miR-384-5p
GO:0006140	regulation of nucleotide metabolic process	Edn1, Epha3, Gnal, Arhgap17, Bcl6, Adcy9, Epha2, Gna13, Gnai1, Gnai2, Pde2a, Pip5k1a, Rgs9, Rhog, Tbc1d9b	mmu-miR-124, mmu-miR-26a, mmu-miR-30e, mmu-miR-379, mmu-miR-384-5p
GO:0006164	purine nucleotide biosynthetic process	Edn1, Gnal, Adcy9, Gna13, Gnai1, Gnai2, Pde2a	mmu-miR-124, mmu-miR-26a, mmu-miR-30e, mmu-miR-379, mmu-miR-384-5p
GO:0006184	GTP catabolic process	Epha3, Gnal, Arhgap17, Atl3, Bcl6, Epha2, Gna13, Gnai1, Gnai2, Pip5k1a, Rab27a, Rasd2, Rgs9, Rhog, Rras, Tbc1d9b	mmu-miR-124, mmu-miR-26a, mmu-miR-30e, mmu-miR-384-5p
GO:0006282	regulation of DNA repair	Eya1, Ube2v2, Usp1	mmu-miR-124, mmu-miR-30e, mmu-miR-384-5p
GO:0006357	regulation of transcription from RNA polymerase II promoter	Map3k1, Eya1, Tcfap4, Abca2, Lhx2, Bmp6, Ctdsp1, Dlx5, Eaf1, Fzd4, Hoxb1, Htatip2, Nr4a1, Sfrp5, Sox8, Tbx19, Xrcc6, Smad1	mmu-miR-124, mmu-miR-26a, mmu-miR-30e, mmu-miR-379, mmu-miR-384-5p
GO:0006464	protein modification process	P4ha2, Suv39h2, Eya1, Ttl, P4ha1, C1galt1, Ctdsp1, Eya2, Galnt10, Mtr, Qsox1, Rab3d, Snai2, Spock3, Ssh3, Stt3a, Yeats2, Fkbp3, Ptpn13	mmu-miR-124, mmu-miR-26a, mmu-miR-30e, mmu-miR-384-5p
GO:0006468	protein phosphorylation	Epha3, Kalrn, 2610018G03Rik, Dapk1, Epha2, Htatip2, Pan3, Pim3, Ror2, Srpk3, Stk39, Abi2, Smad1, Rps6ka2, Ppp3cb	mmu-miR-124, mmu-miR-26a, mmu-miR-30e, mmu-miR-384-5p
GO:0006511	ubiquitin-dependent protein catabolic process	Edem3, Map3k1, Ube2v2, Trib3, Ttc3, Ube2i, Usp1, Usp2, Usp30, Usp48, Erlin1	mmu-miR-124, mmu-miR-26a, mmu-miR-30e, mmu-miR-379, mmu-miR-384-5p

GO:0006520	cellular amino acid metabolic process	Aldh4a1, Bckdha, Farsa, Gfpt2, Hars2, Htatip2, Mtr	mmu-miR-124, mmu-miR-30e, mmu-miR-384-5p
GO:0006575	cellular modified amino acid metabolic process	Ctns, P4ha2, P4ha1, Fpgs, Gstk1, Pcyox1	mmu-miR-124, mmu-miR-30e, mmu-miR-384-5p
GO:0006633	fatty acid biosynthetic process	Edn1, Scd2, Fa2h, Scd1, Sphk1	mmu-miR-124, mmu-miR-26a, mmu-miR-30e, mmu-miR-379, mmu-miR-384-5p
GO:0006665	sphingolipid metabolic process	Fa2h, Lass2, B4galt6, Galc, Sphk1	mmu-miR-124, mmu-miR-26a, mmu-miR-30e, mmu-miR-384-5p
GO:0006730	one-carbon metabolic process	Suv39h2, Tet1, Ocln, Aldh1l2, Fpgs, Ftsjd1, Mat2a, Mtr, Nsun2, Pemt, Rab3d	mmu-miR-124, mmu-miR-26a, mmu-miR-30e, mmu-miR-384-5p
GO:0006796	phosphate-containing compound metabolic process	Epha3, Nme4, Ak2, Pip4k2a, Kalrn, 2610018G03Rik, Cycs, Dapk1, Dcakd, Epha2, Gk5, Htatip2, Pan3, Pim3, Pip5k1a, Ror2, Srpk3, Stk39, Abi2, Smad1, Rps6ka2	mmu-miR-124, mmu-miR-26a, mmu-miR-30e, mmu-miR-384-5p
GO:0006810	transport	Fa2h, Myo1e, Arhgap17, Slc25a1, Ache, Aldh1l2, Casc3, Chic2, Cyb5, Eny2, Flot1, Gsn, Htatip2, Lmbrd1, Myh10, Plekhf1, Slc35f5, Sypl, Vamp4	mmu-miR-124, mmu-miR-26a, mmu-miR-30e, mmu-miR-384-5p
GO:0006855	drug transmembrane transport	Slc22a5, Abcc4	mmu-miR-124, mmu-miR-26a, mmu-miR-30e, mmu-miR-379, mmu-miR-384-5p
GO:0006885	regulation of pH	Edn1, Ednrb, Slc9a2, Slc9a9	mmu-miR-124, mmu-miR-26a, mmu-miR-30e, mmu-miR-379, mmu-miR-384-5p
GO:0006915	apoptotic process	Ppm1f, Trim39, Rffl, Dram1, Epha2, Eya2, Gas2, Gsn, Peg3, Trib3, Sema3a	mmu-miR-124, mmu-miR-26a, mmu-miR-30e, mmu-miR-379, mmu-miR-384-5p
GO:0006928	cellular component movement	Kif21b, Celsr3, Ednrb, Efnb1, Itga7, Kif2a, Kif3a, Mdk, Myh10, Myo5a, Nr4a1, Ovol2, Pip5k1a, Rhbdf1, Rhog, Sox8, Abi2	mmu-miR-124, mmu-miR-26a, mmu-miR-30e, mmu-miR-384-5p

GO:0006939	smooth muscle contraction	Edn1, Cav1, Ednrb, Sphk1, Tpcn2, Htr1d	mmu-miR-124, mmu-miR-30e, mmu-miR-379, mmu-miR-384-5p
GO:0006970	response to osmotic stress	Map3k1, Tsc22d4, Xrcc6	mmu-miR-124, mmu-miR-30e, mmu-miR-379, mmu-miR-384-5p
GO:0007015	actin filament organization	Ppm1f, Arhgap17, Lcp1, Sdc4, Pawr	mmu-miR-124, mmu-miR-26a, mmu-miR-30e, mmu-miR-384-5p
GO:0007017	microtubule-based process	Epha3, Kif21b, Eya1, Ttl, Fgfr1op, Cav1, Clip3, Gas2l1, Kif2a, Kif3a, Numa1, Ranbp10	mmu-miR-124, mmu-miR-26a, mmu-miR-30e, mmu-miR-384-5p
GO:0007155	cell adhesion	Sdk1, Pcdh8, Cd164, Cdh20, Ctnnd2, Rapgef1, Acan, Ache, Barx2, Celsr3, Col12a1, Dsg2, Itga7, Itgae, Pcdh1, Pcdh17, Pip5k1a, Pnn, Ror2, Tmem8b	mmu-miR-124, mmu-miR-26a, mmu-miR-30e, mmu-miR-379, mmu-miR-384-5p
GO:0007156	homophilic cell adhesion	Pcdh8, Cdh20, Celsr3, Dsg2, Pcdh1	mmu-miR-124, mmu-miR-26a, mmu-miR-30e, mmu-miR-384-5p
GO:0007160	cell-matrix adhesion	Adam9, Pip5k1a, Tmem8b, Nid1	mmu-miR-124, mmu-miR-26a, mmu-miR-30e, mmu-miR-384-5p
GO:0007254	JNK cascade	Edn1, Map3k1, Fzd4, Ror2, Sfrp5, Sh3rf1	mmu-miR-124, mmu-miR-26a, mmu-miR-30e, mmu-miR-379, mmu-miR-384-5p
GO:0007265	Ras protein signal transduction	Bcl6, Kalrn, Arhgef4, Dhcr24, Flot1, Gna13, Rhog, Sos2, Spata13, Brap	mmu-miR-124, mmu-miR-26a, mmu-miR-30e, mmu-miR-384-5p
GO:0007268	synaptic transmission	Dmd, Edn1, Cacnb2, Pcdh8, Ctnnd2, Gria2, Ube2v2, Ache, Aldh9a1, Apba3, Cadps, Gnai1, Gnai2, Myo5a, Rasd2, Slc29a1, Sphk1	mmu-miR-124, mmu-miR-26a, mmu-miR-30e, mmu-miR-379, mmu-miR-384-5p
GO:0007389	pattern specification process	Edn1, Eya1, Pcdh8, Lhx2, Efnb1, Gas1, Gna13, Hoxb1, Kif3a, Ovol2, Rarg, Ror2, Fst	mmu-miR-124, mmu-miR-26a, mmu-miR-30e, mmu-miR-379, mmu-miR-384-5p
GO:0007411	axon guidance	Lhx2, Dlx5, Efnb1, Flot1, Gas1, Myh10, Slit1, Sema3a	mmu-miR-124, mmu-miR-30e, mmu-miR-379, mmu-miR-384-5p
GO:0007413	axonal fasciculation	Celsr3, Sema3a	mmu-miR-124, mmu-miR-30e, mmu-miR-379, mmu-miR-384-5p

GO:0007420	brain development	Ctns, Lhx2, Fzd4, Gas1, Hoxb1, Mdk, Myh10, Sfrp5, Slit1, Sphk1, Tbx19, Twsg1, Smad1	mmu-miR-124, mmu-miR-26a, mmu-miR-30e, mmu-miR-384-5p
GO:0007423	sensory organ development	Edn1, Map3k1, Eya1, Ache, Bmp6, Epha2, Fzd4, Gas1, Myh10, Rarg, Ror2, Sox8, Twsg1, Abi2, Mab21l1	mmu-miR-124, mmu-miR-26a, mmu-miR-30e, mmu-miR-379, mmu-miR-384-5p
GO:0007497	posterior midgut development	Ednrb	mmu-miR-124, mmu-miR-30e, mmu-miR-384-5p
GO:0007517	muscle organ development	Dmd, Fxr1, Cd164, Barx2, Cav1, Lemd2, Rcan1, Rxra, Ryr1, Snta1, Sox8, Srpk3, Usp2	mmu-miR-124, mmu-miR-30e, mmu-miR-384-5p
GO:0007519	skeletal muscle tissue development	Dmd, Barx2, Cav1, Lemd2, Rcan1, Ryr1, Snta1, Sox8, Srpk3, Usp2	mmu-miR-124, mmu-miR-30e, mmu-miR-384-5p
GO:0008152	metabolic process	Kif21b, Acp2, Rffl, Dram1, Enpp4, Galnt12, Galnt9, Glt8d1, Kif2a, Kif3a, Myh10, Parp16, Parp9, Ptprz1, Tmed10, Tpst2, Zdhhc20, Mkrn3	mmu-miR-124, mmu-miR-26a, mmu-miR-30e, mmu-miR-384-5p
GO:0008154	actin polymerization or depolymerization	Map3k1, Twf1, Arpc1b, Gsn, Ssh3	mmu-miR-124, mmu-miR-26a, mmu-miR-30e, mmu-miR-379, mmu-miR-384-5p
GO:0008219	cell death	Ppm1f, Acp2, Trim39, Rffl, 2610018G03Rik, Ache, Bcl2l13, Dram1, Dsg2, Epha2, Eya2, Gas2, Gsn, Mdk, Mmd2, Peg3, Rilpl1, Trib3, Sema3a	mmu-miR-124, mmu-miR-26a, mmu-miR-30e, mmu-miR-379, mmu-miR-384-5p
GO:0008284	positive regulation of cell proliferation	Edn1, Eya1, Pdcd10, Bcl6, Fgfr1op, Abcc4, Apln, Bmp6, Dlx5, Ednrb, Efnb1, Gas1, Gnai2, Gpam, Nr4a1, Sox8, Sphk1, Mab21l1	mmu-miR-124, mmu-miR-26a, mmu-miR-30e, mmu-miR-379, mmu-miR-384-5p
GO:0008285	negative regulation of cell proliferation	Tcfap4, Bcl6, Cav1, Dhcr24, Gas1, Ovol2, Pemt, Rarg, Ror2, Rxra, Sfrp5, Pawr, Smad1, Rps6ka2	mmu-miR-124, mmu-miR-26a, mmu-miR-30e, mmu-miR-384-5p
GO:0008361	regulation of cell size	Edn1, Ttl, Akt1s1, Slit1, Sema3a	mmu-miR-124, mmu-miR-30e, mmu-miR-379, mmu-miR-384-5p
GO:0008544	epidermis development	Barx2, Dhcr24, Epha2, Myo5a, Ovol2, Plod3, Ppl, Ryr1, Fst	mmu-miR-124, mmu-miR-26a, mmu-miR-30e, mmu-miR-379, mmu-miR-384-5p

GO:0008643	carbohydrate transport	Edn1, Slc2a12, Clip3, Slc17a5, Trib3	mmu-miR-124, mmu-miR-379
GO:0009057	macromolecule catabolic process	Agl, Casc3, Cycs, Dhcr24, Nthl1, Pan3, Tmed10	mmu-miR-124, mmu-miR-26a, mmu-miR-30e, mmu-miR-384-5p
GO:0009058	biosynthetic process	Ache, Aldh1l2, Dhcr24, Gfpt2, Gpt2, Sc5d	mmu-miR-124, mmu-miR-30e, mmu-miR-384-5p
GO:0009078	pyruvate family amino acid metabolic process	Gpt2	mmu-miR-124, mmu-miR-30e, mmu-miR-384-5p
GO:0009100	glycoprotein metabolic process	Edem3, Acan, C1galt1, Dhcr24, Galnt10, Spock3, Stt3a, Tmed10, Pawr	mmu-miR-124, mmu-miR-26a, mmu-miR-30e, mmu-miR-379, mmu-miR-384-5p
GO:0009108	coenzyme biosynthetic process	Acss1, Dcakd, Fpgs, Mat2a	mmu-miR-124, mmu-miR-26a, mmu-miR-30e, mmu-miR-384-5p
GO:0009119	ribonucleoside metabolic process	Nme4, Ocln, Dcakd, Mat2a, Pemt	mmu-miR-124, mmu-miR-26a, mmu-miR-30e, mmu-miR-384-5p
GO:0009124	nucleoside monophosphate biosynthetic process	Edn1, Gnal, Adcy9, Ampd3, Gna13, Gnai1, Gnai2, Pde2a	mmu-miR-124, mmu-miR-26a, mmu-miR-30e, mmu-miR-379, mmu-miR-384-5p
GO:0009146	purine nucleoside triphosphate catabolic process	Epha3, Gnal, Myo1e, Arhgap17, Atl3, Bcl6, Abca2, Atp1a1, Epha2, Gna13, Gnai1, Gnai2, Pcyox1, Pip5k1a, Rab27a, Rasd2, Rgs9, Rhog, Rras, Tbc1d9b, Xrcc6	mmu-miR-124, mmu-miR-26a, mmu-miR-30e, mmu-miR-384-5p
GO:0009154	purine ribonucleotide catabolic process	Epha3, Gnal, Myo1e, Arhgap17, Atl3, Bcl6, Abca2, Atp1a1, Epha2, Gna13, Gnai1, Gnai2, Pcyox1, Pip5k1a, Rab27a, Rasd2, Rgs9, Rhog, Rras, Tbc1d9b, Xrcc6	mmu-miR-124, mmu-miR-26a, mmu-miR-30e, mmu-miR-384-5p
GO:0009166	nucleotide catabolic process	Epha3, Gnal, Myo1e, Arhgap17, Atl3, Bcl6, Abca2, Atp1a1, Epha2, Gna13, Gnai1, Gnai2, Pcyox1, Pde2a, Pip5k1a, Rab27a, Rasd2, Rgs9, Rhog, Rras, Tbc1d9b, Xrcc6	mmu-miR-124, mmu-miR-26a, mmu-miR-30e, mmu-miR-379, mmu-miR-384-5p

GO:0009203	ribonucleoside triphosphate catabolic process	Epha3, Gnal, Myo1e, Arhgap17, Atl3, Bcl6, Abca2, Atp1a1, Epha2, Gna13, Gnai1, Gnai2, Pcyox1, Pip5k1a, Rab27a, Rasd2, Rgs9, Rhog, Rras, Tbc1d9b, Xrcc6	mmu-miR-124, mmu-miR-26a, mmu-miR-30e, mmu-miR-384-5p
GO:0009306	protein secretion	Dnajc1, Adam9, Cbln4, Gpam, Rhbdf1, Steap3	mmu-miR-124, mmu-miR-26a, mmu-miR-30e, mmu-miR-384-5p
GO:0009792	embryo development ending in birth or egg hatching	Edn1, Rdh10, Sall4, Eya1, Myo1e, Pcdh8, Tet1, Lhx2, Apba3, Epha2, Gas1, Gna13, Hoxb1, Kif3a, Myh10, Ovol2, Plod3, Ror2, Sfrp5, Sox8	mmu-miR-124, mmu-miR-26a, mmu-miR-30e, mmu-miR-379, mmu-miR-384-5p
GO:0010591	regulation of lamellipodium assembly	Epha2, Ssh3	mmu-miR-124, mmu-miR-26a
GO:0010648	negative regulation of cell communication	Bcl6, Aes, Akt1s1, Cav1, Epha2, Gas1, Gnai1, Gnai2, Lemd2, Ovol2, Rgs9, Ror2, Sfrp5, Sh3rf1, Snai2, Snx6, Twsg1, Fst, Pawr, Brap	mmu-miR-124, mmu-miR-26a, mmu-miR-30e, mmu-miR-379, mmu-miR-384-5p
GO:0010817	regulation of hormone levels	Edn1, Rdh10, Apln, Atp1a1, Bmp6, Cpt1a, Fzd4, Hadh, Myo5a, Pim3, Sox8, Ppp3cb	mmu-miR-124, mmu-miR-26a, mmu-miR-30e, mmu-miR-379, mmu-miR-384-5p
GO:0010837	regulation of keratinocyte proliferation	Ovol2, Snai2	mmu-miR-124, mmu-miR-30e, mmu-miR-384-5p
GO:0010942	positive regulation of cell death	Map3k1, Tcfap4, Bcl6, Anxa5, Cav1, Clip3, Cycs, Dapk1, Ern1, Htatip2, Nr4a1, Plekhf1, Rab27a, Rarg, Rxra, Sh3rf1, Steap3, Pawr, Rps6ka2, Ppp3cb	mmu-miR-124, mmu-miR-26a, mmu-miR-30e, mmu-miR-379, mmu-miR-384-5p
GO:0010957	negative regulation of vitamin D biosynthetic process	Snai2	mmu-miR-124, mmu-miR-30e, mmu-miR-384-5p
GO:0010975	regulation of neuron projection development	Epha3, Ttl, Ube2v2, Kalrn, Ache, Slit1, Sphk1, Ssh3, Sema3a	mmu-miR-124, mmu-miR-26a, mmu-miR-30e, mmu-miR-379, mmu-miR-384-5p
GO:0014028	notochord formation	Epha2	mmu-miR-124, mmu-miR-26a

GO:0014032	neural crest cell development	Edn1, Rdh10, Snai2	mmu-miR-124, mmu-miR-30e, mmu-miR-379, mmu-miR-384-5p
GO:0014826	vein smooth muscle contraction	Edn1, Ednrb	mmu-miR-124, mmu-miR-30e, mmu-miR-379, mmu-miR-384-5p
GO:0014889	muscle atrophy	Fbxo32	mmu-miR-30e, mmu-miR-379, mmu-miR-384-5p
GO:0015031	protein transport	Bcl6, Rffl, Ap1m2, Apba3, Cadps, Clip3, Lman2l, Pip5k1a, Pttg1ip, Rab27a, Rab3d, Rufy1, Sdad1, Sec61a2, Sft2d2, Slc15a4, Snap29, Snx6, Tmed1, Tmed10, Vps35	mmu-miR-124, mmu-miR-26a, mmu-miR-30e, mmu-miR-384-5p
GO:0015833	peptide transport	Edn1, Cpt1a, Hadh, Myo5a, Pim3, Slc15a4, Ppp3cb	mmu-miR-124, mmu-miR-26a, mmu-miR-30e, mmu-miR-379, mmu-miR-384-5p
GO:0016032	viral reproduction	Map3k1, Tcfap4, Rxra, Zkscan3, Zscan20	mmu-miR-124, mmu-miR-30e, mmu-miR-379, mmu-miR-384-5p
GO:0016044	cellular membrane organization	Myo1e, Gria2, Ache, Cav1, Clip3, Dhcr24, Epha2, Flot1, Kif3a, Lpcat2, Myh10, Pip5k1a, Plekhf1, Rhog, Rufy1, Tmed10, Zmpste24	mmu-miR-124, mmu-miR-26a, mmu-miR-30e, mmu-miR-379, mmu-miR-384-5p
GO:0016311	dephosphorylation	Ppm1f, Acp2, Eya1, Ctdsp1, Eya2, Ptprz1, Rcan1, Ssh3, Wdr81, Ptpn13, Ppp3cb	mmu-miR-124, mmu-miR-26a, mmu-miR-30e, mmu-miR-384-5p
GO:0016331	morphogenesis of embryonic epithelium	Rdh10, Sall4, Pcdh8, Lhx2, Ovol2, Sfrp5, Sox8	mmu-miR-124, mmu-miR-30e, mmu-miR-384-5p
GO:0016477	cell migration	Celsr3, Efnb1, Itga7, Mdk, Myh10, Ovol2, Rhbdf1, Slit1, Sox8, Abi2	mmu-miR-124, mmu-miR-26a, mmu-miR-30e, mmu-miR-384-5p
GO:0016567	protein ubiquitination	Map3k1, Rasd2, Rnf135, Sphk1, Trib3, Ttc3, Ube2i, Ube4a, Ube2e3, Brap, Ube2ql1	mmu-miR-124, mmu-miR-26a, mmu-miR-30e, mmu-miR-379, mmu-miR-384-5p
GO:0016576	histone dephosphorylation	Eya1, Eya2	mmu-miR-124, mmu-miR-30e, mmu-miR-384-5p

GO:0018130	heterocycle biosynthetic process	Edn1, Gnal, Nme4, Ak2, Adcy9, Aldh4a1, Ampd3, Atp1a1, Fpgs, Gna13, Gnai1, Gnai2, Mat2a, Pde2a, Slc25a13	mmu-miR-124, mmu-miR-26a, mmu-miR-30e, mmu-miR-379, mmu-miR-384-5p
GO:0018208	peptidyl-proline modification	P4ha2, P4ha1, Fkbp3	mmu-miR-124, mmu-miR-26a, mmu-miR-30e, mmu-miR-384-5p
GO:0018401	peptidyl-proline hydroxylation to 4-hydroxy-L-proline	P4ha2, P4ha1	mmu-miR-124, mmu-miR-30e, mmu-miR-384-5p
GO:0019216	regulation of lipid metabolic process	Atp1a1, Bmp6, Gpam, Scd1, Slc27a1, Snai2	mmu-miR-124, mmu-miR-30e, mmu-miR-384-5p
GO:0019222	regulation of metabolic process	Gria2, Kalrn, Ache, Apba3, Apln, Arhgef4, Cycs, Dhcr24, Gas2l1, Rcan1, Serinc2, Sh3rf1, Sos2, Spata13, Spock3, Wdr81, Ppp3cb	mmu-miR-124, mmu-miR-26a, mmu-miR-30e, mmu-miR-379, mmu-miR-384-5p
GO:0019637	organophosphate metabolic process	Pip4k2a, Agpat5, Gpam, Lpcat2, Lpcat3, Pemt, Pip5k1a, Prdx6, Serinc2, Slc27a1	mmu-miR-124, mmu-miR-30e, mmu-miR-384-5p
GO:0019932	second-messenger-mediated signaling	Edn1, Gnal, Ednrb, Gna13, Gnai1, Gnai2, Itpr3, Pde2a, Rasd2, Rcan1, Sphk1	mmu-miR-124, mmu-miR-26a, mmu-miR-30e, mmu-miR-379, mmu-miR-384-5p
GO:0019933	cAMP-mediated signaling	Gnal, Gna13, Gnai1, Gnai2, Pde2a, Rasd2	mmu-miR-124, mmu-miR-26a, mmu-miR-30e, mmu-miR-379, mmu-miR-384-5p
GO:0019934	cGMP-mediated signaling	Ednrb, Pde2a	mmu-miR-124, mmu-miR-30e, mmu-miR-379, mmu-miR-384-5p
GO:0021553	olfactory nerve development	Dmd	mmu-miR-124, mmu-miR-30e, mmu-miR-384-5p
GO:0021602	cranial nerve morphogenesis	Dmd, Hoxb1	mmu-miR-124, mmu-miR-30e, mmu-miR-384-5p
GO:0021629	olfactory nerve structural organization	Dmd	mmu-miR-124, mmu-miR-30e, mmu-miR-384-5p

GO:0021904	dorsal/ventral neural tube patterning	Gas1, Kif3a	mmu-miR-124, mmu-miR-30e, mmu-miR-384-5p
GO:0021915	neural tube development	Sall4, Lhx2, Epha2, Gas1, Kif3a, Ovol2, Plod3, Sfrp5	mmu-miR-124, mmu-miR-26a, mmu-miR-30e, mmu-miR-384-5p
GO:0022604	regulation of cell morphogenesis	Ttl, Ache, Brwd3, Gas2, Gna13, Myh10, Slit1, Ssh3, Ttc3, Sema3a	mmu-miR-124, mmu-miR-26a, mmu-miR-30e, mmu-miR-379, mmu-miR-384-5p
GO:0022612	gland morphogenesis	Cav1, Epha2, Rarg, Rxra, Twsg1, Sema3a	mmu-miR-124, mmu-miR-26a, mmu-miR-30e, mmu-miR-379, mmu-miR-384-5p
GO:0023051	regulation of signaling	Ctnnd2, Gria2, Ache, Apln, Cpt1a, Hadh, Pim3, Ppp3cb	mmu-miR-124, mmu-miR-26a, mmu-miR-30e, mmu-miR-379, mmu-miR-384-5p
GO:0023057	negative regulation of signaling	Bcl6, Aes, Akt1s1, Cav1, Epha2, Gas1, Gnai1, Gnai2, Lemd2, Ovol2, Rgs9, Ror2, Sfrp5, Sh3rf1, Snai2, Snx6, Twsg1, Fst, Pawr, Brap	mmu-miR-124, mmu-miR-26a, mmu-miR-30e, mmu-miR-379, mmu-miR-384-5p
GO:0030001	metal ion transport	Cacnb2, Slc22a5, Atp1a1, Cacng5, Cav1, Gnai2, Itpr3, Kcnk10, Kcnk2, Kctd5, Kctd8, Myo5a, Nipa1, Ryr1, Scn7a, Slc1a4, Slc9a2, Slc9a9, Steap3, Stk39, Tpcn2	mmu-miR-124, mmu-miR-26a, mmu-miR-30e, mmu-miR-384-5p
GO:0030030	cell projection organization	Dmd, Epha3, Ttl, Lhx2, Ube2v2, Kalrn, Ache, Celsr3, Dlx5, Efhd1, Efnb1, Flot1, Gas1, Gsn, Ptprz1, Slit1, Sphk1, Abi2, Sema3a	mmu-miR-124, mmu-miR-26a, mmu-miR-30e, mmu-miR-379, mmu-miR-384-5p
GO:0030031	cell projection assembly	Arhgef4, Epha2, Kif3a, Pip5k1a, Poc1b, Prdx6, Spata13, Ssh3	mmu-miR-124, mmu-miR-26a, mmu-miR-30e, mmu-miR-384-5p
GO:0030036	actin cytoskeleton organization	Edn1, Epha3, Bcl6, Inf2, Myh10, Pip5k1a, Rhog, Epb4.1l3	mmu-miR-124, mmu-miR-26a, mmu-miR-30e, mmu-miR-379, mmu-miR-384-5p
GO:0030047	actin modification	Twf1	mmu-miR-26a, mmu-miR-30e, mmu-miR-384-5p
GO:0030050	vesicle transport along actin filament	Myo5a	mmu-miR-124, mmu-miR-30e, mmu-miR-384-5p

GO:0030154	cell differentiation	Suv39h2, Fxr1, Adamts9, C1galt1, Dmrta1, Gna13, Htatip2, Kif2a, Mdk, Myo5a, Numa1, Nxn, Paqr8, Ppl, Rab27a, Ror2, Scd1, Sema6d, Sfrp5, Srpk3, Tbx19, Smad1, Ppp3cb	mmu-miR-124, mmu-miR-26a, mmu-miR-30e, mmu-miR-384-5p
GO:0030155	regulation of cell adhesion	Epha3, Ppm1f, Adam9, Bcl6, Epha2, Fzd4, Gsn, Sdc4, Snai2, Nid1	mmu-miR-124, mmu-miR-26a, mmu-miR-30e, mmu-miR-384-5p
GO:0030163	protein catabolic process	Edem3, Map3k1, Adam9, Ube2v2, Fbxo27, Pcyox1, Rhbdf1, Trib3, Ttc3, Ube2i, Usp1, Usp2, Usp30, Usp48, Yme1l1, Zmpste24, Erlin1	mmu-miR-124, mmu-miR-26a, mmu-miR-30e, mmu-miR-379, mmu-miR-384-5p
GO:0030199	collagen fibril organization	P4ha1, Acan, Plod3	mmu-miR-124, mmu-miR-30e, mmu-miR-384-5p
GO:0030318	melanocyte differentiation	Adamts9, Ednrb, Myo5a, Rab27a	mmu-miR-124, mmu-miR-30e, mmu-miR-384-5p
GO:0030326	embryonic limb morphogenesis	Rdh10, Sall4, Lnp, Dlx5, Gas1, Rarg	mmu-miR-124, mmu-miR-26a, mmu-miR-30e, mmu-miR-384-5p
GO:0030334	regulation of cell migration	Edn1, Map3k1, Ppm1f, Adam9, Fgfr1op, Acan, Dll4, Epha2, Gna13, Ror2, Rras, Snai2, Spata13, Sphk1, Sema3a	mmu-miR-124, mmu-miR-26a, mmu-miR-30e, mmu-miR-379, mmu-miR-384-5p
GO:0030497	fatty acid elongation	Elovl5, Pecr	mmu-miR-124, mmu-miR-30e, mmu-miR-384-5p
GO:0030509	BMP signaling pathway	Bmp6, Cav1, Twsg1, Fst, Smad1	mmu-miR-124, mmu-miR-26a, mmu-miR-30e, mmu-miR-379, mmu-miR-384-5p
GO:0030516	regulation of axon extension	Ttl, Slit1, Sema3a	mmu-miR-124, mmu-miR-30e, mmu-miR-379, mmu-miR-384-5p
GO:0030800	negative regulation of cyclic nucleotide metabolic process	Edn1, Gnai1, Gnai2, Pde2a	mmu-miR-124, mmu-miR-30e, mmu-miR-379, mmu-miR-384-5p

GO:0030802	regulation of cyclic nucleotide biosynthetic process	Edn1, Gnal, Adcy9, Gna13, Gnai1, Gnai2, Pde2a	mmu-miR-124, mmu-miR-26a, mmu-miR-30e, mmu-miR-379, mmu-miR-384-5p
GO:0030809	negative regulation of nucleotide biosynthetic process	Edn1, Gnai1, Gnai2, Pde2a	mmu-miR-124, mmu-miR-30e, mmu-miR-379, mmu-miR-384-5p
GO:0030814	regulation of cAMP metabolic process	Edn1, Gnal, Adcy9, Gna13, Gnai1, Gnai2, Pde2a	mmu-miR-124, mmu-miR-26a, mmu-miR-30e, mmu-miR-379, mmu-miR-384-5p
GO:0030818	negative regulation of cAMP biosynthetic process	Edn1, Gnai1, Gnai2, Pde2a	mmu-miR-124, mmu-miR-30e, mmu-miR-379, mmu-miR-384-5p
GO:0030832	regulation of actin filament length	Map3k1, Twf1, Arpc1b, Gsn, Ssh3	mmu-miR-124, mmu-miR-26a, mmu-miR-30e, mmu-miR-379, mmu-miR-384-5p
GO:0030833	regulation of actin filament polymerization	Map3k1, Twf1, Arpc1b, Gsn	mmu-miR-124, mmu-miR-26a, mmu-miR-30e, mmu-miR-379, mmu-miR-384-5p
GO:0030857	negative regulation of epithelial cell differentiation	Cav1, Ovol2	mmu-miR-124, mmu-miR-30e, mmu-miR-384-5p
GO:0031325	positive regulation of cellular metabolic process	Edn1, Apln, Cav1, Clip3, Ednrb, Gpam, Pemt, Rasd2, Scd1, Snai2, Sphk1, Pawr	mmu-miR-124, mmu-miR-26a, mmu-miR-30e, mmu-miR-379, mmu-miR-384-5p
GO:0031331	positive regulation of cellular catabolic process	Adam9, Ube2v2, Cpt1a, Mtdh, Trib3	mmu-miR-124, mmu-miR-26a, mmu-miR-30e, mmu-miR-384-5p
GO:0031585	regulation of inositol 1,4,5-trisphosphate-sensitive calcium-release channel activity	Myo5a	mmu-miR-124, mmu-miR-30e, mmu-miR-384-5p

GO:0031667	response to nutrient levels	Epha3, Gdap2, Agl, Gas2l1, Pemt, Rarg, Rxra, Snai2	mmu-miR-124, mmu-miR-30e, mmu-miR-384-5p
GO:0031987	locomotion involved in locomotory behavior	Fzd4, Myo5a	mmu-miR-124, mmu-miR-30e, mmu-miR-384-5p
GO:0032231	regulation of actin filament bundle assembly	Map3k1, Ppm1f, Sdc4	mmu-miR-124, mmu-miR-30e, mmu-miR-379, mmu-miR-384-5p
GO:0032272	negative regulation of protein polymerization	Twf1, Clip3, Gsn	mmu-miR-124, mmu-miR-26a, mmu-miR-30e, mmu-miR-384-5p
GO:0032319	regulation of Rho GTPase activity	Epha3, Bcl6, Epha2, Pip5k1a, Rhog	mmu-miR-124, mmu-miR-26a, mmu-miR-30e, mmu-miR-384-5p
GO:0032331	negative regulation of chondrocyte differentiation	Rarg, Snai2	mmu-miR-124, mmu-miR-30e, mmu-miR-384-5p
GO:0032400	melanosome localization	Myo5a, Rab27a	mmu-miR-124, mmu-miR-30e, mmu-miR-384-5p
GO:0032402	melanosome transport	Myo5a, Rab27a	mmu-miR-124, mmu-miR-30e, mmu-miR-384-5p
GO:0032526	response to retinoic acid	Epha3, Gdap2, Rarg, Rxra	mmu-miR-124, mmu-miR-30e, mmu-miR-384-5p
GO:0032728	positive regulation of interferon-beta production	Polr3g, Rnf135	mmu-miR-124, mmu-miR-30e, mmu-miR-384-5p
GO:0032764	negative regulation of mast cell cytokine production	Bcl6	mmu-miR-124, mmu-miR-30e, mmu-miR-384-5p
GO:0032787	monocarboxylic acid metabolic process	Edn1, P4ha2, Rdh10, Scd2, Fa2h, P4ha1, Cav1, Cyb5, Elovl5, Gpam, Myo5a, Pecr, Scd1, Slc27a1, Sphk1, Trib3	mmu-miR-124, mmu-miR-26a, mmu-miR-30e, mmu-miR-379, mmu-miR-384-5p

GO:0032836	glomerular basement membrane development	Myo1e, Nid1	mmu-miR-124, mmu-miR-26a, mmu-miR-30e, mmu-miR-384-5p
GO:0032844	regulation of homeostatic process	Edn1, Slc22a5, Apln, Cav1, Ednrb, Gas2l1, Gpam, Pim3, Ryr1, Slc27a1, Tpcn2, Ppp3cb	mmu-miR-124, mmu-miR-26a, mmu-miR-30e, mmu-miR-379, mmu-miR-384-5p
GO:0032856	activation of Ras GTPase activity	Epha2, Pip5k1a, Rhog	mmu-miR-124, mmu-miR-26a
GO:0032863	activation of Rac GTPase activity	Epha2, Pip5k1a, Rhog	mmu-miR-124, mmu-miR-26a
GO:0032970	regulation of actin filament-based process	Edn1, Epha3, Map3k1, Ppm1f, Twf1, Arpc1b, Gsn, Sdc4, Ssh3	mmu-miR-124, mmu-miR-26a, mmu-miR-30e, mmu-miR-379, mmu-miR-384-5p
GO:0032989	cellular component morphogenesis	Edn1, Bcl6, Brwd3, Epha2, Fzd4, Gas2, Gna13, Plod3, Snai2, Ttc3	mmu-miR-124, mmu-miR-26a, mmu-miR-30e, mmu-miR-379, mmu-miR-384-5p
GO:0032990	cell part morphogenesis	Dmd, Ttl, Lhx2, Kalrn, Ache, Celsr3, Dlx5, Efnb1, Flot1, Gas1, Gsn, Kif3a, Myh10, Poc1b, Ptprz1, Slit1, Ssh3, Sema3a	mmu-miR-124, mmu-miR-26a, mmu-miR-30e, mmu-miR-379, mmu-miR-384-5p
GO:0033043	regulation of organelle organization	Edn1, Epha3, Map3k1, Ppm1f, Tet1, Twf1, Arpc1b, Cav1, Clip3, Gas2l1, Gsn, Sdc4, Snai2, Ssh3, Surf4	mmu-miR-124, mmu-miR-26a, mmu-miR-30e, mmu-miR-379, mmu-miR-384-5p
GO:0033159	negative regulation of protein import into nucleus, translocation	Pde2a	mmu-miR-124, mmu-miR-379
GO:0033273	response to vitamin	Epha3, Gdap2, Pemt, Rarg, Rxra, Snai2	mmu-miR-124, mmu-miR-30e, mmu-miR-384-5p
GO:0034241	positive regulation of macrophage fusion	Adam9	mmu-miR-26a, mmu-miR-30e, mmu-miR-384-5p
GO:0034329	cell junction assembly	Epha3, Ppm1f, Pip5k1a, Sdc4, Snai2	mmu-miR-124, mmu-miR-30e, mmu-miR-384-5p

GO:0034654	nucleobase-containing compound biosynthetic process	Edn1, Gnal, Nme4, Ak2, Adcy9, Ampd3, Atp1a1, Gna13, Gnai1, Gnai2, Mat2a, Pde2a, Slc25a13	mmu-miR-124, mmu-miR-26a, mmu-miR-30e, mmu-miR-379, mmu-miR-384-5p
GO:0034765	regulation of ion transmembrane transport	Cacnb2, Cacng5, Clic6, Gnai2, Kcnk2, Kctd5, Kctd8, Myo5a, Scn7a, Tpcn2	mmu-miR-124, mmu-miR-30e, mmu-miR-384-5p
GO:0035023	regulation of Rho protein signal transduction	Kalrn, Arhgef4, Flot1, Sos2, Spata13	mmu-miR-124, mmu-miR-26a, mmu-miR-30e, mmu-miR-384-5p
GO:0035107	appendage morphogenesis	Rdh10, Sall4, Lnp, Dlx5, Gas1, Rarg	mmu-miR-124, mmu-miR-26a, mmu-miR-30e, mmu-miR-384-5p
GO:0035121	tail morphogenesis	Epha2, Sfrp5	mmu-miR-124, mmu-miR-26a
GO:0035239	tube morphogenesis	Edn1, Rdh10, Sall4, Eya1, Lhx2, Epha2, Gna13, Ovol2, Sfrp5, Sox8	mmu-miR-124, mmu-miR-26a, mmu-miR-30e, mmu-miR-379, mmu-miR-384-5p
GO:0035414	negative regulation of catenin import into nucleus	Sfrp5, Snai2	mmu-miR-124, mmu-miR-30e, mmu-miR-384-5p
GO:0035637	multicellular organismal signaling	Dmd, Edn1, Cacnb2, Pcdh8, Ctnnd2, Gria2, Ube2v2, Ache, Aldh9a1, Apba3, Cadps, Gnai1, Gnai2, Myo5a, Rasd2, Slc29a1, Sphk1	mmu-miR-124, mmu-miR-26a, mmu-miR-30e, mmu-miR-379, mmu-miR-384-5p
GO:0035645	enteric smooth muscle cell differentiation	Ednrb	mmu-miR-124, mmu-miR-30e, mmu-miR-384-5p
GO:0035690	cellular response to drug	Ppm1f, Pde2a, Ppp3cb	mmu-miR-124, mmu-miR-26a, mmu-miR-30e, mmu-miR-379, mmu-miR-384-5p
GO:0035810	positive regulation of urine volume	Edn1, Ednrb	mmu-miR-124, mmu-miR-30e, mmu-miR-379, mmu-miR-384-5p
GO:0035815	positive regulation of renal sodium excretion	Edn1, Ednrb	mmu-miR-124, mmu-miR-30e, mmu-miR-379, mmu-miR-384-5p

GO:0035921	desmosome disassembly	Snai2	mmu-miR-124, mmu-miR-30e, mmu-miR-384-5p
GO:0035964	COPI-coated vesicle budding	Tmed10	mmu-miR-124, mmu-miR-26a, mmu-miR-30e, mmu-miR-384-5p
GO:0040008	regulation of growth	Ppm1f, Ttl, Bcl6, Fgfr1op, Aes, Gas1, Gas2l1, Gpam, Scd1, Sfrp5, Slit1, Sphk1, Tmem8b, Ube2e3, Sema3a	mmu-miR-124, mmu-miR-30e, mmu-miR-379, mmu-miR-384-5p
GO:0040012	regulation of locomotion	Edn1, Map3k1, Ppm1f, Adam9, Fgfr1op, Acan, Dll4, Epha2, Gna13, Ror2, Rras, Slit1, Snai2, Spata13, Sphk1, Sema3a	mmu-miR-124, mmu-miR-26a, mmu-miR-30e, mmu-miR-379, mmu-miR-384-5p
GO:0042045	epithelial fluid transport	Edn1, Ednrb	mmu-miR-124, mmu-miR-30e, mmu-miR-379, mmu-miR-384-5p
GO:0042117	monocyte activation	Adam9	mmu-miR-26a, mmu-miR-30e, mmu-miR-384-5p
GO:0042275	error-free postreplication DNA repair	Ube2v2	mmu-miR-124, mmu-miR-30e, mmu-miR-384-5p
GO:0042278	purine nucleoside metabolic process	Ocln, Dcakd, Mat2a, Pemt	mmu-miR-124, mmu-miR-26a, mmu-miR-30e, mmu-miR-384-5p
GO:0042310	vasoconstriction	Edn1, Apln, Cav1, Ednrb	mmu-miR-124, mmu-miR-30e, mmu-miR-379, mmu-miR-384-5p
GO:0042330	taxis	Edn1, Ppm1f, Lhx2, Dlx5, Ednrb, Efnb1, Epha2, Flot1, Gas1, Myh10, Nr4a1, Pip5k1a, Rhog, Slit1, Sema3a	mmu-miR-124, mmu-miR-26a, mmu-miR-30e, mmu-miR-379, mmu-miR-384-5p
GO:0042474	middle ear morphogenesis	Edn1, Eya1, Gas1	mmu-miR-124, mmu-miR-379
GO:0042493	response to drug	Slc22a5, Abcc4, Atp1a1, Hadh, Hadha, Mat2a, Pemt, Smad1	mmu-miR-124, mmu-miR-26a, mmu-miR-30e, mmu-miR-379, mmu-miR-384-5p
GO:0042692	muscle cell differentiation	Edn1, Barx2, Ednrb, Lemd2, Myh10, Rcan1, Rxra, Ryr1, Snta1, Sox8	mmu-miR-124, mmu-miR-30e, mmu-miR-379, mmu-miR-384-5p

GO:0042759	long-chain fatty acid biosynthetic process	Elovl5, Myo5a	mmu-miR-124, mmu-miR-30e, mmu-miR-384-5p
GO:0042851	L-alanine metabolic process	Gpt2	mmu-miR-124, mmu-miR-30e, mmu-miR-384-5p
GO:0042981	regulation of apoptotic process	2610018G03Rik, Bcl2l13, Htatip2, Mdk, Rilpl1	mmu-miR-124, mmu-miR-30e, mmu-miR-384-5p
GO:0042986	positive regulation of amyloid precursor protein biosynthetic process	Pawr	mmu-miR-26a, mmu-miR-30e, mmu-miR-384-5p
GO:0042987	amyloid precursor protein catabolic process	Dhcr24, Tmed10	mmu-miR-124, mmu-miR-26a, mmu-miR-30e, mmu-miR-384-5p
GO:0043010	camera-type eye development	Map3k1, Rdh10, Ache, Epha2, Fzd4, Gas1, Myh10, Sox8, Twsg1, Abi2, Mab21l1	mmu-miR-124, mmu-miR-26a, mmu-miR-30e, mmu-miR-379, mmu-miR-384-5p
GO:0043065	positive regulation of apoptotic process	Map3k1, Tcfap4, Bcl6, Anxa5, Cav1, Clip3, Cycs, Dapk1, Ern1, Nr4a1, Plekhf1, Rab27a, Rarg, Rxra, Sh3rf1, Steap3, Pawr, Rps6ka2, Ppp3cb	mmu-miR-124, mmu-miR-26a, mmu-miR-30e, mmu-miR-379, mmu-miR-384-5p
GO:0043066	negative regulation of apoptotic process	Edn1, Eya1, Tcfap4, Pdcd10, Bcl6, Ube2v2, Akt1s1, Cav1, Dapk1, Dhcr24, Ednrb, Gas1, Gpam, Mtdh, Pim3, Sh3rf1, Snai2, Sox8, Sphk1	mmu-miR-124, mmu-miR-26a, mmu-miR-30e, mmu-miR-379, mmu-miR-384-5p
GO:0043112	receptor metabolic process	Dmd, Edn1, Gria2, Ache, Cav1	mmu-miR-124, mmu-miR-30e, mmu-miR-379, mmu-miR-384-5p
GO:0043506	regulation of JUN kinase activity	Edn1, Map3k1, Fzd4, Sfrp5	mmu-miR-124, mmu-miR-30e, mmu-miR-379, mmu-miR-384-5p
GO:0043538	regulation of actin phosphorylation	Twf1	mmu-miR-26a, mmu-miR-30e, mmu-miR-384-5p
GO:0043549	regulation of kinase activity	Edn1, Map3k1, Ppm1f, Tcfap4, Pdcd10, Adam9, Fgfr1op, Akt1s1, Cav1, Ern1, Fzd4, Gnai2, Sdc4, Sfrp5, Slc27a1, Sphk1, Trib3	mmu-miR-124, mmu-miR-26a, mmu-miR-30e, mmu-miR-379, mmu-miR-384-5p

GO:0043632	modification-dependent macromolecule catabolic process	Edem3, Map3k1, Ube2v2, Pcyox1, Trib3, Ttc3, Ube2i, Usp1, Usp2, Usp30, Usp48, Zmpste24, Erlin1	mmu-miR-124, mmu-miR-26a, mmu-miR-30e, mmu-miR-379, mmu-miR-384-5p
GO:0044087	regulation of cellular component biogenesis	Edn1, Twf1, Ube2v2, Arpc1b, Cav1, Clip3, Epha2, Gsn, Snai2, Ssh3	mmu-miR-124, mmu-miR-26a, mmu-miR-30e, mmu-miR-379, mmu-miR-384-5p
GO:0044249	cellular biosynthetic process	Rdh10, Scd2, Fa2h, Dgat2, Acss1, Aldh9a1, B4galt6, Dcakd, Elovl5, Mtr, Myo5a, Pecr, Scd1	mmu-miR-124, mmu-miR-26a, mmu-miR-30e, mmu-miR-384-5p
GO:0044255	cellular lipid metabolic process	Edn1, Rdh10, Scd2, Fa2h, Lass2, B4galt6, Cyb5, Elovl5, Lpcat3, Myo5a, Pecr, Scd1, Sphk1, Trib3	mmu-miR-124, mmu-miR-26a, mmu-miR-30e, mmu-miR-379, mmu-miR-384-5p
GO:0044283	small molecule biosynthetic process	Edn1, Scd2, Fa2h, Acadvl, Acss1, Aldh4a1, Elovl5, Fpgs, Mat2a, Mtr, Myo5a, Pecr, Scd1, Slc27a1, Snai2, Sphk1, Trib3	mmu-miR-124, mmu-miR-26a, mmu-miR-30e, mmu-miR-379, mmu-miR-384-5p
GO:0045137	development of primary sexual characteristics	Cbx2, Rdh10, Bmp6, Dhcr24, Fzd4, Sfrp5, Sox8, Fst	mmu-miR-124, mmu-miR-30e, mmu-miR-379, mmu-miR-384-5p
GO:0045446	endothelial cell differentiation	Bmp6, Pde2a, Plod3	mmu-miR-124, mmu-miR-379
GO:0045596	negative regulation of cell differentiation	Tet1, Bcl6, Ctdsp1, Gas2l1, Rcan1, Slit1, Sox8, Trib3, Ttc3, Twsg1, Fst, Sema3a	mmu-miR-124, mmu-miR-26a, mmu-miR-30e, mmu-miR-379, mmu-miR-384-5p
GO:0045664	regulation of neuron differentiation	Eya1, Bmp6, Ctdsp1, Ednrb, Sox8, Ttc3	mmu-miR-124, mmu-miR-30e, mmu-miR-384-5p
GO:0045667	regulation of osteoblast differentiation	Bmp6, Dlx5, Snai2, Twsg1, Smad1	mmu-miR-124, mmu-miR-26a, mmu-miR-30e, mmu-miR-384-5p
GO:0045761	regulation of adenylate cyclase activity	Gnal, Adcy9, Gna13, Gnai1, Gnai2	mmu-miR-124, mmu-miR-26a, mmu-miR-30e, mmu-miR-384-5p

GO:0045933	positive regulation of muscle contraction	Edn1, Atp1a1, Sphk1	mmu-miR-124, mmu-miR-379
GO:0045939	negative regulation of steroid metabolic process	Atp1a1, Snai2	mmu-miR-124, mmu-miR-30e, mmu-miR-384-5p
GO:0046394	carboxylic acid biosynthetic process	Aldh4a1, Fpgs, Mat2a, Mtr, Slc27a1	mmu-miR-124, mmu-miR-26a, mmu-miR-30e, mmu-miR-384-5p
GO:0046486	glycerolipid metabolic process	Pip4k2a, Dgat2, Agpat5, Cav1, Cpt1a, Gpam, Lipe, Lpcat2, Pemt, Pip5k1a, Serinc2, Slc27a1	mmu-miR-124, mmu-miR-30e, mmu-miR-384-5p
GO:0046498	S-adenosylhomocysteine metabolic process	Ocln, Pemt	mmu-miR-124, mmu-miR-26a, mmu-miR-30e, mmu-miR-384-5p
GO:0046500	S-adenosylmethionine metabolic process	Ocln, Mat2a, Pemt	mmu-miR-124, mmu-miR-26a, mmu-miR-30e, mmu-miR-384-5p
GO:0046879	hormone secretion	Edn1, Apln, Cpt1a, Fzd4, Hadh, Myo5a, Pim3, Ppp3cb	mmu-miR-124, mmu-miR-26a, mmu-miR-30e, mmu-miR-379, mmu-miR-384-5p
GO:0048066	developmental pigmentation	Adamts9, Ednrb, Myo5a, Rab27a	mmu-miR-124, mmu-miR-30e, mmu-miR-384-5p
GO:0048468	cell development	Dmd, Rdh10, Myo1e, Tet1, Pip4k2a, Bcl6, Acan, Efhd1, Ovol2, Pde2a, Rarg, Rcan1, Ryr1, Snta1, Abi2, Rps6ka2	mmu-miR-124, mmu-miR-26a, mmu-miR-30e, mmu-miR-379, mmu-miR-384-5p
GO:0048484	enteric nervous system development	Ednrb, Sox8	mmu-miR-124, mmu-miR-30e, mmu-miR-384-5p
GO:0048532	anatomical structure arrangement	Dmd, Hoxb1	mmu-miR-124, mmu-miR-30e, mmu-miR-384-5p
GO:0048545	response to steroid hormone stimulus	Tcfap4, Adam9, Agl, Cav1, Nr4a1, Rarg, Rcan1, Rxra	mmu-miR-124, mmu-miR-26a, mmu-miR-30e, mmu-miR-384-5p

GO:0048562	embryonic organ morphogenesis	Edn1, Rdh10, Eya1, Dlx5, Epha2, Gas1, Hoxb1, Ovol2, Rarg, Ror2	mmu-miR-124, mmu-miR-26a, mmu-miR-30e, mmu-miR-379, mmu-miR-384-5p
GO:0048585	negative regulation of response to stimulus	Bcl6, Aes, Akt1s1, Cav1, Epha2, Gas1, Lemd2, Ovol2, Rgs9, Ror2, Sfrp5, Sh3rf1, Slit1, Snai2, Snx6, Twsg1, Fst, Sema3a, Pawr, Ppp3cb, Brap	mmu-miR-124, mmu-miR-26a, mmu-miR-30e, mmu-miR-379, mmu-miR-384-5p
GO:0048589	developmental growth	Rdh10, Sall4, Ttl, Gas1, Myo5a, Rarg, Sfrp5, Slit1, Sema3a	mmu-miR-124, mmu-miR-30e, mmu-miR-379, mmu-miR-384-5p
GO:0048608	reproductive structure development	Rdh10, Slc22a5, Fzd4, Rarg, Rxra, Sfrp5, Sox8, Fst	mmu-miR-124, mmu-miR-30e, mmu-miR-379, mmu-miR-384-5p
GO:0048646	anatomical structure formation involved in morphogenesis	Rdh10, Sall4, Eya1, Pcdh8, Tet1, Adam9, Lhx2, Dlx5, Hoxb1, Kif3a, Myh10, Poc1b, Ror2, Sfrp5, Snai2, Sox8, Twsg1, Smad1	mmu-miR-124, mmu-miR-26a, mmu-miR-30e, mmu-miR-384-5p
GO:0048667	cell morphogenesis involved in neuron differentiation	Ttl, Lhx2, Kalrn, Ache, Celsr3, Dlx5, Efnb1, Flot1, Gas1, Myh10, Ptprz1, Slit1, Ssh3, Sema3a	mmu-miR-124, mmu-miR-26a, mmu-miR-30e, mmu-miR-379, mmu-miR-384-5p
GO:0048705	skeletal system morphogenesis	Rdh10, Eya1, Bmp6, Dlx5, Gas1, Hoxb1, Rarg	mmu-miR-124, mmu-miR-30e, mmu-miR-384-5p
GO:0048729	tissue morphogenesis	Edn1, Rdh10, Sall4, Eya1, Pcdh8, Lhx2, Epha2, Gna13, Ovol2, Plod3, Rarg, Ror2, Rxra, Sfrp5, Snai2, Sox8, Twsg1, Fst, Sema3a, Smad1	mmu-miR-124, mmu-miR-26a, mmu-miR-30e, mmu-miR-379, mmu-miR-384-5p
GO:0048762	mesenchymal cell differentiation	Edn1, Rdh10, Ednrb, Efnb1, Ovol2, Snai2, Sox8	mmu-miR-124, mmu-miR-30e, mmu-miR-379, mmu-miR-384-5p
GO:0048812	neuron projection morphogenesis	Dmd, Ttl, Lhx2, Kalrn, Ache, Celsr3, Dlx5, Efnb1, Flot1, Gas1, Myh10, Ptprz1, Slit1, Ssh3, Sema3a	mmu-miR-124, mmu-miR-26a, mmu-miR-30e, mmu-miR-379, mmu-miR-384-5p
GO:0048820	hair follicle maturation	Barx2, Myo5a	mmu-miR-124, mmu-miR-30e, mmu-miR-384-5p
GO:0048843	negative regulation of axon extension involved in axon guidance	Slit1, Sema3a	mmu-miR-124, mmu-miR-30e, mmu-miR-379, mmu-miR-384-5p

GO:0048856	anatomical structure development	Pcdh8, Adam9, Ctnnd2, Lnp, Brwd3, Eya2, Gas2, Gsn, Poc1b	mmu-miR-124, mmu-miR-26a, mmu-miR-30e, mmu-miR-384-5p
GO:0050434	positive regulation of viral transcription	Map3k1, Tcfap4	mmu-miR-124, mmu-miR-30e, mmu-miR-379, mmu-miR-384-5p
GO:0050872	white fat cell differentiation	Scd1, Snai2	mmu-miR-124, mmu-miR-30e, mmu-miR-384-5p
GO:0050878	regulation of body fluid levels	Edn1, Lnp, Anxa5, Apln, Cav1, Ednrb, Gna13, Rab27a	mmu-miR-124, mmu-miR-26a, mmu-miR-30e, mmu-miR-379, mmu-miR-384-5p
GO:0050886	endocrine process	Edn1, Apln, Fzd4, Rps6ka2	mmu-miR-124, mmu-miR-26a, mmu-miR-30e, mmu-miR-379, mmu-miR-384-5p
GO:0050919	negative chemotaxis	Slit1, Sema3a	mmu-miR-124, mmu-miR-30e, mmu-miR-379, mmu-miR-384-5p
GO:0051046	regulation of secretion	Dnajc1, Edn1, Adam9, Apln, Cadps, Cpt1a, Ednrb, Gpam, Hadh, Myo5a, Pim3, Rab27a, Rab3d, Rhbdf1, Sphk1, Ppp3cb	mmu-miR-124, mmu-miR-26a, mmu-miR-30e, mmu-miR-379, mmu-miR-384-5p
GO:0051049	regulation of transport	Cacnb2, Abca2, Atp1a1, Cacng5, Clic6, Gnai2, Kcnk2, Kctd5, Kctd8, Scn7a, Stk39, Tpcn2, Trib3	mmu-miR-124, mmu-miR-26a, mmu-miR-30e, mmu-miR-384-5p
GO:0051055	negative regulation of lipid biosynthetic process	Atp1a1, Slc27a1, Snai2	mmu-miR-124, mmu-miR-30e, mmu-miR-384-5p
GO:0051056	regulation of small GTPase mediated signal transduction	Epha3, Bcl6, Kalrn, Arhgef4, Epha2, Flot1, Pip5k1a, Rhog, Sos2, Spata13, Tbc1d9b	mmu-miR-124, mmu-miR-26a, mmu-miR-30e, mmu-miR-384-5p
GO:0051223	regulation of protein transport	Dnajc1, Adam9, Gpam, Lcp1, Pde2a, Rhbdf1, Sfrp5, Snai2, Sphk1	mmu-miR-124, mmu-miR-26a, mmu-miR-30e, mmu-miR-379, mmu-miR-384-5p
GO:0051270	regulation of cellular component movement	Edn1, Map3k1, Ppm1f, Adam9, Bcl6, Fgfr1op, Acan, Dll4, Epha2, Gna13, Ror2, Rras, Slit1, Snai2, Spata13, Sphk1, Sema3a	mmu-miR-124, mmu-miR-26a, mmu-miR-30e, mmu-miR-379, mmu-miR-384-5p

GO:0051384	response to glucocorticoid stimulus	Tcfap4, Adam9, Agl, Rxra	mmu-miR-124, mmu-miR-26a, mmu-miR-30e, mmu-miR-384-5p
GO:0051494	negative regulation of cytoskeleton organization	Map3k1, Twf1, Clip3, Gas2l1, Gsn	mmu-miR-124, mmu-miR-26a, mmu-miR-30e, mmu-miR-379, mmu-miR-384-5p
GO:0051495	positive regulation of cytoskeleton organization	Edn1, Map3k1, Ppm1f, Cav1, Sdc4	mmu-miR-124, mmu-miR-30e, mmu-miR-379, mmu-miR-384-5p
GO:0051592	response to calcium ion	Adam9, Cav1, Itpr3, Slc25a13	mmu-miR-124, mmu-miR-26a, mmu-miR-30e, mmu-miR-384-5p
GO:0051603	proteolysis involved in cellular protein catabolic process	Edem3, Map3k1, Ube2v2, Pcyox1, Rhbdf1, Trib3, Ttc3, Ube2i, Usp1, Usp2, Usp30, Usp48, Zmpste24, Erlin1	mmu-miR-124, mmu-miR-26a, mmu-miR-30e, mmu-miR-379, mmu-miR-384-5p
GO:0051604	protein maturation	Capn1, Dhcr24, Gas1, Vps35, Rps6ka2	mmu-miR-124, mmu-miR-26a, mmu-miR-30e, mmu-miR-384-5p
GO:0051643	endoplasmic reticulum localization	Myo5a	mmu-miR-124, mmu-miR-30e, mmu-miR-384-5p
GO:0051656	establishment of organelle localization	Myo5a, Rab27a, Tmed10	mmu-miR-124, mmu-miR-26a, mmu-miR-30e, mmu-miR-384-5p
GO:0051898	negative regulation of protein kinase B signaling cascade	Epha2, Sfrp5	mmu-miR-124, mmu-miR-26a
GO:0051905	establishment of pigment granule localization	Myo5a, Rab27a	mmu-miR-124, mmu-miR-30e, mmu-miR-384-5p
GO:0051960	regulation of nervous system development	Epha3, Eya1, Ttl, Ube2v2, Kalrn, Ache, Aspa, Bmp6, Ctdsp1, Dll4, Ednrb, Sfrp5, Slit1, Sox8, Sphk1, Ssh3, Ttc3, Xrcc6, Sema3a	mmu-miR-124, mmu-miR-26a, mmu-miR-30e, mmu-miR-379, mmu-miR-384-5p
GO:0055002	striated muscle cell development	Edn1, Myh10, Rcan1, Ryr1, Snta1, Sox8	mmu-miR-124, mmu-miR-379

GO:0055086	nucleobase-containing small molecule metabolic process	Ocln, Mat2a, Pemt	mmu-miR-124, mmu-miR-26a, mmu-miR-30e, mmu-miR-384-5p
GO:0060035	notochord cell development	Epha2	mmu-miR-124, mmu-miR-26a
GO:0060047	heart contraction	Edn1, Apln, Atp1a1, Sema3a, Rps6ka2	mmu-miR-124, mmu-miR-26a, mmu-miR-30e, mmu-miR-379, mmu-miR-384-5p
GO:0060142	regulation of syncytium formation by plasma membrane fusion	Adam9	mmu-miR-26a, mmu-miR-30e, mmu-miR-384-5p
GO:0060284	regulation of cell development	Edn1, Epha3, Eya1, Ttl, Ube2v2, Kalrn, Ache, Aspa, Bmp6, Ctdsp1, Dll4, Ednrb, Slit1, Sox8, Sphk1, Ssh3, Ttc3, Xrcc6, Sema3a	mmu-miR-124, mmu-miR-26a, mmu-miR-30e, mmu-miR-379, mmu-miR-384-5p
GO:0060326	cell chemotaxis	Edn1, Ednrb, Epha2, Nr4a1, Pip5k1a, Rhog	mmu-miR-124, mmu-miR-26a, mmu-miR-30e, mmu-miR-379, mmu-miR-384-5p
GO:0060537	muscle tissue development	Dmd, Eya1, Barx2, Cav1, Eya2, Lemd2, Myh10, Rcan1, Rxra, Ryr1, Snta1, Sox8, Srpk3, Usp2	mmu-miR-124, mmu-miR-30e, mmu-miR-384-5p
GO:0060548	negative regulation of cell death	Edn1, Eya1, Tcfap4, Pdcd10, Bcl6, Ube2v2, Akt1s1, Cav1, Dapk1, Dhcr24, Ednrb, Gas1, Gpam, Mtdh, Pim3, Sh3rf1, Snai2, Sox8, Sphk1	mmu-miR-124, mmu-miR-26a, mmu-miR-30e, mmu-miR-379, mmu-miR-384-5p
GO:0060627	regulation of vesicle-mediated transport	Cadps, Cav1, Clip3, Gas1, Kif3a, Myo5a, Rab27a, Rab3d, Rufy1	mmu-miR-124, mmu-miR-30e, mmu-miR-384-5p
GO:0061035	regulation of cartilage development	Rarg, Snai2, Smad1	mmu-miR-124, mmu-miR-26a, mmu-miR-30e, mmu-miR-384-5p
GO:0061138	morphogenesis of a branching epithelium	Edn1, Rdh10, Eya1, Epha2, Gna13, Rxra, Sox8, Sema3a	mmu-miR-124, mmu-miR-26a, mmu-miR-30e, mmu-miR-379, mmu-miR-384-5p

GO:0070201	regulation of establishment of protein localization	Clip3, Epha2, Rhog	mmu-miR-124, mmu-miR-26a
GO:0070384	Harderian gland development	Rarg	mmu-miR-124, mmu-miR-30e, mmu-miR-384-5p
GO:0070563	negative regulation of vitamin D receptor signaling pathway	Snai2	mmu-miR-124, mmu-miR-30e, mmu-miR-384-5p
GO:0070647	protein modification by small protein conjugation or removal	Map3k1, Eny2, Rasd2, Rnf135, Sphk1, Trib3, Ttc3, Ube2i, Ube4a, Usp1, Usp2, Ube2e3, Brap, Ube2ql1	mmu-miR-124, mmu-miR-26a, mmu-miR-30e, mmu-miR-379, mmu-miR-384-5p
GO:0070727	cellular macromolecule localization	Eya1, Bcl6, Rffl, Ap1m2, Casc3, Clip3, Epha2, Flot1, Lcp1, Pde2a, Pip5k1a, Pttg1ip, Rab27a, Rhog, Sfrp5, Snai2, Snx6, Sphk1, Tmed10	mmu-miR-124, mmu-miR-26a, mmu-miR-30e, mmu-miR-379, mmu-miR-384-5p
GO:0071322	cellular response to carbohydrate stimulus	Pim3, Rps6ka2, Ppp3cb	mmu-miR-124, mmu-miR-26a, mmu-miR-30e, mmu-miR-384-5p
GO:0071702	organic substance transport	Ctns, Abca2, Slc22a5, Cadps, Cav1, Fzd4, Osbpl11, Osbpl7, Slc15a4, Slc1a4, Slc25a13, Slc27a1	mmu-miR-124, mmu-miR-26a, mmu-miR-30e, mmu-miR-384-5p
GO:0072358	cardiovascular system development	Sall4, Slc22a5, Kif3a, Myh10, Ryr1, Snai2, Rps6ka2, Ppp3cb	mmu-miR-124, mmu-miR-26a, mmu-miR-30e, mmu-miR-384-5p
GO:0072523	purine-containing compound catabolic process	Epha3, Gnal, Myo1e, Arhgap17, Atl3, Bcl6, Abca2, Atp1a1, Epha2, Gna13, Gnai1, Gnai2, Pcyox1, Pde2a, Pip5k1a, Rab27a, Rasd2, Rgs9, Rhog, Rras, Tbc1d9b, Xrcc6	mmu-miR-124, mmu-miR-26a, mmu-miR-30e, mmu-miR-379, mmu-miR-384-5p
GO:0072659	protein localization in plasma membrane	Clip3, Epha2, Flot1, Pip5k1a, Rhog	mmu-miR-124, mmu-miR-26a
GO:0080135	regulation of cellular response to stress	Edn1, Map3k1, Eya1, Ube2v2, Fzd4, Sfrp5, Sh3rf1, Snai2, Usp1	mmu-miR-124, mmu-miR-26a, mmu-miR-30e, mmu-miR-379, mmu-miR-384-5p

GO:0090004	positive regulation of establishment of protein localization in plasma membrane	Clip3, Epha2, Rhog	mmu-miR-124, mmu-miR-26a
GO:0090066	regulation of anatomical structure size	Edn1, Map3k1, Ttl, Twf1, Akt1s1, Apln, Arpc1b, Cav1, Ednrb, Gsn, Plod3, Slit1, Ssh3, Sema3a	mmu-miR-124, mmu-miR-26a, mmu-miR-30e, mmu-miR-379, mmu-miR-384-5p
GO:0090090	negative regulation of canonical Wnt receptor signaling pathway	Aes, Cav1, Ror2, Sfrp5, Snai2	mmu-miR-124, mmu-miR-30e, mmu-miR-384-5p
GO:0090101	negative regulation of transmembrane receptor protein serine/threonine kinase signaling pathway	Cav1, Snx6, Twsg1, Fst	mmu-miR-124, mmu-miR-30e, mmu-miR-379, mmu-miR-384-5p
GO:0090150	establishment of protein localization in membrane	Clip3, Epha2, Pip5k1a, Rhog	mmu-miR-124, mmu-miR-26a
GO:0090257	regulation of muscle system process	Edn1, Atp1a1, Casq2, Cav1, Ryr1, Sphk1	mmu-miR-124, mmu-miR-379
GO:0097011	cellular response to granulocyte macrophage colony-stimulating factor stimulus	Pde2a	mmu-miR-124, mmu-miR-379
GO:2000027	regulation of organ morphogenesis	Edn1, Eya1, Ror2, Rxra, Sfrp5, Sox8	mmu-miR-124, mmu-miR-379
GO:2000647	negative regulation of stem cell proliferation	Snai2	mmu-miR-124, mmu-miR-30e, mmu-miR-384-5p

GO ID (molecular function)	Go Term (molecular function GO Term)	Gene Symbol (genes enriched by down-regulated miRNAs)	Down-regulated miRNAs
GO:0000146	microfilament motor activity	Myh10, Myo5a	mmu-miR-124, mmu-miR-30e, mmu-miR-384-5p
GO:0001664	G-protein coupled receptor binding	Edn1, Gnal, Homer2, Apln, Clic6, Ednrb, Gna13, Gnai1, Gnai2, Ror2	mmu-miR-124, mmu-miR-26a, mmu-miR-30e, mmu-miR-379, mmu-miR-384-5p
GO:0001948	glycoprotein binding	Dmd, Fbxo27, Slit1, Fst	mmu-miR-124, mmu-miR-30e, mmu-miR-379, mmu-miR-384-5p
GO:0002151	G-quadruplex RNA binding	Fxr1	mmu-miR-124, mmu-miR-30e, mmu-miR-384-5p
GO:0003708	retinoic acid receptor activity	Rarg, Rxra	mmu-miR-124, mmu-miR-30e, mmu-miR-384-5p
GO:0003779	actin binding	Dmd, Homer2, Twf1, Arpc1b, Gsn, Inf2, Myo5a, Snta1, Ssh3, Pawr, Epb4.1l3	mmu-miR-124, mmu-miR-26a, mmu-miR-30e, mmu-miR-379, mmu-miR-384-5p
GO:0003924	GTPase activity	Gnal, Atl3, Gna13, Gnai1, Gnai2, Rab27a, Rasd2, Rhog, Rras	mmu-miR-124, mmu-miR-26a, mmu-miR-30e, mmu-miR-384-5p
GO:0003950	NAD+ ADP-ribosyltransferase activity	Parp16, Parp9	mmu-miR-124, mmu-miR-30e, mmu-miR-384-5p
GO:0004021	L-alanine:2-oxoglutarate aminotransferase activity	Gpt2	mmu-miR-124, mmu-miR-30e, mmu-miR-384-5p
GO:0004221	ubiquitin thiolesterase activity	Usp1, Usp2, Usp30, Usp48	mmu-miR-124, mmu-miR-26a, mmu-miR-30e, mmu-miR-384-5p
GO:0004360	glutamine-fructose-6-phosphate transaminase (isomerizing) activity	Gfpt2	mmu-miR-124, mmu-miR-30e, mmu-miR-384-5p

GO:0004478	methionine adenosyltransferase activity	Mat2a	mmu-miR-124, mmu-miR-26a, mmu-miR-30e, mmu-miR-384-5p
GO:0004569	glycoprotein endo-alpha-1,2-mannosidase activity	Edem3	mmu-miR-30e, mmu-miR-379, mmu-miR-384-5p
GO:0004656	procollagen-proline 4-dioxygenase activity	P4ha2, P4ha1	mmu-miR-124, mmu-miR-30e, mmu-miR-384-5p
GO:0004706	JUN kinase kinase kinase activity	Map3k1	mmu-miR-124, mmu-miR-30e, mmu-miR-379, mmu-miR-384-5p
GO:0004710	MAPK/ERK kinase kinase activity	Map3k1	mmu-miR-124, mmu-miR-30e, mmu-miR-379, mmu-miR-384-5p
GO:0004711	ribosomal protein S6 kinase activity	Rps6ka2	mmu-miR-26a, mmu-miR-30e, mmu-miR-384-5p
GO:0004721	phosphoprotein phosphatase activity	Acp2, Eya1, Eya2, Ptprz1, Ssh3, Ptpn13	mmu-miR-124, mmu-miR-26a, mmu-miR-30e, mmu-miR-384-5p
GO:0004722	protein serine/threonine phosphatase activity	Ppm1f, Ctdsp1, Ppp3cb	mmu-miR-124, mmu-miR-26a, mmu-miR-30e, mmu-miR-384-5p
GO:0004842	ubiquitin-protein ligase activity	Map3k1, Rnf135, Ttc3, Ube2i, Ube4a, Ube2e3, Brap, Ube2ql1	mmu-miR-124, mmu-miR-26a, mmu-miR-30e, mmu-miR-379, mmu-miR-384-5p
GO:0005003	ephrin receptor activity	Epha3, Epha2	mmu-miR-124, mmu-miR-26a
GO:0005088	Ras guanyl-nucleotide exchange factor activity	Kalrn, Arhgef4, Ranbp10, Sos2, Spata13	mmu-miR-124, mmu-miR-26a, mmu-miR-30e, mmu-miR-384-5p
GO:0005244	voltage-gated ion channel activity	Cacnb2, Cacng5, Clic6, Kcnk2, Kctd5, Kctd8, Ryr1, Scn7a, Tpcn2	mmu-miR-124, mmu-miR-30e, mmu-miR-384-5p
GO:0005261	cation channel activity	Cacnb2, Cacng5, Itpr3, Kcnk10, Kcnk2, Kctd5, Kctd8, Ryr1, Scn7a, Tpcn2	mmu-miR-124, mmu-miR-30e, mmu-miR-384-5p

GO:0005506	iron ion binding	P4ha2, Scd2, Fa2h, Tet1, P4ha1, 1110031I02Rik, Cyb5, Cycs, Plod3, Scd1, Steap3	mmu-miR-124, mmu-miR-26a, mmu-miR-30e, mmu-miR-384-5p
GO:0005516	calmodulin binding	Myo1e, Dapk1, Myh10, Myo5a, Snta1, Sphk1, Ppp3cb	mmu-miR-124, mmu-miR-26a, mmu-miR-30e, mmu-miR-384-5p
GO:0005518	collagen binding	Adam9, Ache, Nid1	mmu-miR-124, mmu-miR-26a, mmu-miR-30e, mmu-miR-384-5p
GO:0005525	GTP binding	Gnal, Atl3, Gna13, Gnai1, Gnai2, Rab27a, Rab3d, Rasd2, Rhog, Rras, Sept10, Sept9, Suclg2	mmu-miR-124, mmu-miR-26a, mmu-miR-30e, mmu-miR-384-5p
GO:0005543	phospholipid binding	Myo1e, Twf1, Kalrn, Anxa4, Anxa5, Arhgef4, Cadps, Itpr3, Pemt, Plekhf1, Snta1, Snx6, Sos2, Spata13	mmu-miR-124, mmu-miR-26a, mmu-miR-30e, mmu-miR-384-5p
GO:0008119	thiopurine S-methyltransferase activity	Ocln	mmu-miR-26a, mmu-miR-30e, mmu-miR-384-5p
GO:0008168	methyltransferase activity	Suv39h2, Ocln, Aldh1l2, Ftsjd1, Mtr, Nsun2, Pemt	mmu-miR-124, mmu-miR-26a, mmu-miR-30e, mmu-miR-384-5p
GO:0008489	UDP-galactose:glucosylceramide beta-1,4-galactosyltransferase activity	B4galt6	mmu-miR-124, mmu-miR-30e, mmu-miR-384-5p
GO:0015075	ion transmembrane transporter activity	Cacnb2, 2310046O06Rik, Gria2, Slc22a5, Atp1a1, Cacng5, Clic6, Itpr3, Kcnk10, Kcnk2, Kctd5, Kctd8, Pcyox1, Ryr1, Scn7a, Slc1a4, Slc9a2, Slc9a9, Tmco3, Tpcn2	mmu-miR-124, mmu-miR-26a, mmu-miR-30e, mmu-miR-379, mmu-miR-384-5p
GO:0015077	monovalent inorganic cation transmembrane transporter activity	2310046O06Rik, Atp1a1, Kcnk10, Kcnk2, Kctd5, Kctd8, Slc1a4, Slc9a2, Slc9a9, Tmco3	mmu-miR-124, mmu-miR-26a, mmu-miR-30e, mmu-miR-384-5p

GO:0015291	secondary active transmembrane transporter activity	Slc22a5, Slc15a4, Slc17a5, Slc1a4, Slc9a2, Slc9a9, Tmco3	mmu-miR-124, mmu-miR-26a
GO:0015299	solute:hydrogen antiporter activity	Slc9a2, Slc9a9, Tmco3	mmu-miR-124, mmu-miR-26a
GO:0015300	solute:solute antiporter activity	Slc9a2, Slc9a9, Tmco3	mmu-miR-124, mmu-miR-26a
GO:0015385	sodium:hydrogen antiporter activity	Slc9a2, Slc9a9	mmu-miR-124, mmu-miR-26a
GO:0015491	cation:cation antiporter activity	Slc9a2, Slc9a9	mmu-miR-124, mmu-miR-26a
GO:0016307	phosphatidylinositol phosphate kinase activity	Pip4k2a, Pip5k1a	mmu-miR-124, mmu-miR-30e, mmu-miR-384-5p
GO:0016462	pyrophosphatase activity	Gnal, Kif21b, Myo1e, Atl3, Abca2, Atp1a1, Gna13, Gnai1, Gnai2, Kif2a, Kif3a, Myh10, Myo5a, Pcyox1, Rab27a, Rasd2, Rhog, Rras, Xrcc6, Yme1l1	mmu-miR-124, mmu-miR-26a, mmu-miR-30e, mmu-miR-384-5p
GO:0016702	oxidoreductase activity, acting on single donors with incorporation of molecular oxygen, incorporation of two atoms of oxygen	P4ha2, Tet1, P4ha1, 1110031I02Rik, Plod3	mmu-miR-124, mmu-miR-26a, mmu-miR-30e, mmu-miR-384-5p
GO:0016706	oxidoreductase activity, acting on paired donors, with incorporation or reduction of molecular oxygen, 2-oxoglutarate as one donor, and incorporation of one atom each of oxygen into both donors	Tet1, 1110031I02Rik, Plod3	mmu-miR-124, mmu-miR-26a, mmu-miR-30e, mmu-miR-384-5p

GO:0016747	transferase activity, transferring acyl groups other than amino-acyl groups	Lass2, Dgat2, Agpat5, Cpt1a, Elovl5, Gpam, Hadha, Lpcat2, Lpcat3	mmu-miR-124, mmu-miR-30e, mmu-miR-384-5p
GO:0016757	transferase activity, transferring glycosyl groups	Agl, B4galt6, C1galt1, Galnt10, Galnt12, Galnt9, Glt8d1, Parp16, Parp9, Plod3, Stt3a	mmu-miR-124, mmu-miR-30e, mmu-miR-384-5p
GO:0016817	hydrolase activity, acting on acid anhydrides	Gnal, Kif21b, Myo1e, Atl3, Abca2, Atp1a1, Gna13, Gnai1, Gnai2, Kif2a, Kif3a, Myh10, Myo5a, Pcyox1, Rab27a, Rasd2, Rhog, Rras, Xrcc6, Yme1l1	mmu-miR-124, mmu-miR-26a, mmu-miR-30e, mmu-miR-384-5p
GO:0016874	ligase activity	Map3k1, Ttl, Rffl, Acss1, Farsa, Fpgs, Hars2, Rnf135, Sh3rf1, Slc27a1, Suclg2, Ttc3, Ube2i, Ube4a, Ube2e3, Brap, Mkrn3, Ube2ql1	mmu-miR-124, mmu-miR-26a, mmu-miR-30e, mmu-miR-379, mmu-miR-384-5p
GO:0016881	acid-amino acid ligase activity	Map3k1, Ttl, Rnf135, Ttc3, Ube2i, Ube4a, Ube2e3, Brap, Ube2ql1	mmu-miR-124, mmu-miR-26a, mmu-miR-30e, mmu-miR-379, mmu-miR-384-5p
GO:0019001	guanyl nucleotide binding	Gnal, Atl3, Gna13, Gnai1, Gnai2, Pde2a, Rab27a, Rab3d, Rasd2, Rhog, Rras, Sept10, Sept9, Suclg2	mmu-miR-124, mmu-miR-26a, mmu-miR-30e, mmu-miR-379, mmu-miR-384-5p
GO:0019905	syntaxin binding	Cav1, Myo5a, Snap29	mmu-miR-124, mmu-miR-30e, mmu-miR-384-5p
GO:0022803	passive transmembrane transporter activity	Cacnb2, Gria2, Cacng5, Clic6, Itpr3, Kcnk10, Kcnk2, Kctd5, Kctd8, Ryr1, Scn7a, Slc1a4, Tpcn2	mmu-miR-124, mmu-miR-30e, mmu-miR-379, mmu-miR-384-5p
GO:0022836	gated channel activity	Cacnb2, Gria2, Cacng5, Clic6, Itpr3, Kcnk2, Kctd5, Kctd8, Ryr1, Scn7a, Tpcn2	mmu-miR-124, mmu-miR-30e, mmu-miR-379, mmu-miR-384-5p
GO:0022838	substrate-specific channel activity	Cacnb2, Gria2, Cacng5, Clic6, Itpr3, Kcnk10, Kcnk2, Kctd5, Kctd8, Ryr1, Scn7a, Slc1a4, Tpcn2	mmu-miR-124, mmu-miR-30e, mmu-miR-379, mmu-miR-384-5p
GO:0022891	substrate-specific transmembrane transporter activity	Slc2a12, Sec61a2, Slc29a1, Slc2a13	mmu-miR-124, mmu-miR-26a, mmu-miR-30e, mmu-miR-384-5p
GO:0030165	PDZ domain binding	Dmd, Gria2, Slc22a5, Fzd4, Sfrp5, Snta1	mmu-miR-124, mmu-miR-30e, mmu-miR-379, mmu-miR-384-5p

GO:0030275	LRR domain binding	Lrrfip2, Pawr	mmu-miR-124, mmu-miR-26a, mmu-miR-30e, mmu-miR-384-5p
GO:0031418	L-ascorbic acid binding	P4ha2, P4ha1, 1110031I02Rik, Plod3	mmu-miR-124, mmu-miR-30e, mmu-miR-384-5p
GO:0031543	peptidyl-proline dioxygenase activity	P4ha2, P4ha1	mmu-miR-124, mmu-miR-30e, mmu-miR-384-5p
GO:0031683	G-protein beta/gamma-subunit complex binding	Gnal, Gna13, Gnai1, Gnai2	mmu-miR-124, mmu-miR-26a, mmu-miR-30e, mmu-miR-384-5p
GO:0031702	type 1 angiotensin receptor binding	Ednrb, Gna13	mmu-miR-124, mmu-miR-26a, mmu-miR-30e, mmu-miR-384-5p
GO:0031752	D5 dopamine receptor binding	Gna13	mmu-miR-124, mmu-miR-26a
GO:0033192	calmodulin-dependent protein phosphatase activity	Ppm1f, Ppp3cb	mmu-miR-124, mmu-miR-26a, mmu-miR-30e, mmu-miR-384-5p
GO:0036004	GAF domain binding	Pde2a	mmu-miR-124, mmu-miR-379
GO:0036094	small molecule binding	P4ha2, P4ha1, 1110031I02Rik, Ache, Aldh1l2, Gpt2, Itpr3, Lmbrd1, Mtr, Plod3	mmu-miR-124, mmu-miR-30e, mmu-miR-384-5p
GO:0042578	phosphoric ester hydrolase activity	Ppm1f, Acp2, Eya1, Atp1a1, Ctdsp1, Eya2, Pde2a, Ptprz1, Ssh3, Ptpn13, Ppp3cb	mmu-miR-124, mmu-miR-26a, mmu-miR-30e, mmu-miR-379, mmu-miR-384-5p
GO:0042802	identical protein binding	Homer2, Tcfap4, Pdcd10, Atl3, Trim39, Dgat2, Fgfr1op, 2610018G03Rik, Ache, Aldh9a1, Casc3, Clic6, Cpt1a, Fzd4, Lcp1, Nr4a1, Pde2a, Slc27a1, Snx6, Tpcn2, Tpd52l2, Usp2, Smad1	mmu-miR-124, mmu-miR-26a, mmu-miR-30e, mmu-miR-379, mmu-miR-384-5p
GO:0043236	laminin binding	Adam9, Ache, Itga7, Nid1	mmu-miR-124, mmu-miR-26a, mmu-miR-30e, mmu-miR-384-5p
GO:0043395	heparan sulfate proteoglycan binding	Slit1, Fst	mmu-miR-124, mmu-miR-30e, mmu-miR-379, mmu-miR-384-5p

GO:0046625	sphingolipid binding	Map3k1, Clip3	mmu-miR-124, mmu-miR-30e, mmu-miR-379, mmu-miR-384-5p
GO:0050998	nitric-oxide synthase binding	Dmd, Cav1	mmu-miR-124, mmu-miR-30e, mmu-miR-384-5p

Supplementary Table 4. Statistical analysis of electrophysiology data in Fig. 2a.

		Comparison of 3 groups (EGFP, miR-26a, miR-384-5p)				miR-26a vs. EGFP				miR-384-5p vs. EGFP		
Time after LTP induction (min)	Normality test passed	Equal variance test passed	P-value (One-way ANOVA)	P-value (Kruskal-Wallis test)	Normality test passed	Equal variance test passed	P-value (t-test)	P-value (Mann-Whitney U Test)	Normality test passed	Equal variance test passed	P-value (t-test)	P-value (Mann-Whitney U Test)
3	N			0.074								
6	N			0.126								
9	N			0.114								
12	N			0.323								
15	N			0.111								
18	N			0.076								
21	N			0.184								
24	N			0.165								
27	N			0.342								
30	N			0.237								
33	N			0.113								
36	N			0.05								
39	N			0.086								
42	N			0.041	N			0.022	Y	N		0.058
45	N			0.013	Y	N		0.026	Y	N		0.006
48	N			0.024	N			0.076	N			0.01
51	N			0.022	Y	N		0.018	Y	N		0.023
54	N			0.024	Y	N		0.018	Y	N		0.028
57	N			0.037	Y	Y	0.021		Y	N		0.033

60	N			0.02	Y	N		0.057	Y	N		0.008
63	N			0.022	N			0.03	Y	N		0.015
66	N			0.01	Y	N		0.009	Y	N		0.015
69	Y	Y	0.003		Y	Y	0.008		Y	Y	0.01	
72	Y	N		0.003	Y	N		0.003	Y	N		0.005
75	Y	N		0.006	Y	N		0.004	Y	N		0.01
78	Y	N		0.003	Y	N		0.004	Y	N		0.005
81	N			0.007	Y	N		0.007	Y	N		0.02
84	N			0.023	Y	Y	0.012		N			0.055
87	N			0.006	Y	N		0.004	Y	N		0.013
90	Y	N		0.002	Y	N		0.001	Y	Y	0.006	
93	N			<0.001	N			<0.001	Y	Y	0.004	
96	N			0.001	Y	Y	<0.001		Y	Y	0.009	
99	N			0.002	N			0.001	Y	Y	0.016	
102	N			0.001	N			<0.001	Y	Y	0.005	
105	N			<0.001	N			<0.001	N			0.002
108	N			<0.001	N			<0.001	N			0.002
111	N			<0.001	N			<0.001	Y	Y	0.02	
114	N			0.001	N			0.001	Y	Y	0.014	
117	N			<0.001	N			<0.001	N			<0.001
120	N			<0.001	N			<0.001	Y	N		0.005

Supplementary Table 5. Statistic analysis of electrophysiology data in Fig. 2b.

	Comparison of 3 groups (EGFP, miR-26a, miR-384-5p)			
Time after LTP induction (min)	Normality test passed	Equal variance test passed	P-value (One-way ANOVA)	P-value (Kruskal-Wallis test)
3	Y	Y	0.925	
6	Y	Y	0.937	
9	Y	Y	0.982	
12	Y	N		0.763
15	Y	Y	0.891	
18	N			0.811
21	Y	Y	0.554	
24	Y	Y	0.214	
27	Y	Y	0.2	
30	N			0.145
33	N			0.454
36	Y	Y	0.437	
39	Y	Y	0.359	
42	Y	Y	0.201	
45	Y	Y	0.29	
48	Y	Y	0.267	
51	Y	Y	0.343	
54	Y	Y	0.286	
57	Y	Y	0.079	
60	Y	Y	0.239	
63	Y	Y	0.124	
66	Y	Y	0.229	
69	Y	Y	0.079	
72	Y	Y	0.151	
75	Y	Y	0.264	
78	Y	Y	0.055	
81	Y	Y	0.463	
84	Y	Y	0.242	
87	Y	Y	0.604	

90	Y	Y	0.173	
93	Y	Y	0.055	
96	Y	Y	0.227	
99	Y	Y	0.318	
102	Y	Y	0.507	
105	Y	Y	0.202	
108	Y	Y	0.555	
111	Y	Y	0.835	
114	Y	Y	0.221	
117	Y	Y	0.389	
120	Y	Y	0.474	

Supplementary Table 6. Statistical analysis of electrophysiology data in Fig. 2c.

	**Comparison of 6 groups (control, miR-26a-AS-Scr, miR-384-5p-AS-Scr, miR-26a-AS, miR-384-5p-AS, miR-191-AS)**			
Time (min)	Normality test passed	Equal variance test passed	P-value (One-way ANOVA)	P-value (Kruskal-Wallis test)
1	Y	Y	0.788	
2	N			0.114
3	Y	Y	0.982	
4	Y	Y	0.257	
5	Y	Y	0.594	
6	Y	Y	0.02	
7	Y	Y	<0.001	
8	Y	N		0.003
9	N			<0.001
10	Y	N		<0.001
11	N			<0.001
12	Y	N		0.004
13	Y	N		0.007
14	Y	N		0.003
15	N			0.006
16	Y	N		<0.001
17	N			<0.001
18	Y	N		<0.001
19	N			<0.001
20	N			<0.001
21	N			<0.001
22	N			<0.001
23	N			<0.001
24	N			<0.001
25	N			<0.001
26	N			<0.001
27	N			<0.001
28	N			<0.001
29	N			<0.001
30	N			<0.001
31	N			<0.001
32	N			<0.001
33	N			<0.001
34	Y	N		0.001
35	N			<0.001
36	N			<0.001
37	N			<0.001
38	N			<0.001
39	N			<0.001
40	N			<0.001
41	N			<0.001

42	Y	N		<0.001
43	N			<0.001
44	Y	Y	<0.001	
45	N			<0.001
46	N			<0.001
47	N			<0.001
48	Y	Y	<0.001	
49	Y	Y	<0.001	
50	N			<0.001
51	Y	Y	<0.001	
52	Y	Y	<0.001	
53	N			0.001
54	Y	Y	<0.001	
55	Y	Y	<0.001	
56	Y	Y	<0.001	
57	Y	Y	<0.001	
58	Y	Y	<0.001	
59	Y	Y	<0.001	
60	Y	Y	<0.001	

	miR-26a-AS-Scr vs. control			
Time (min)	Normality test passed	Equal variance test passed	P-value (t-test)	P-value (Mann-Whitney U Test)
1				
2				
3				
4				
5				
6	Y	Y	0.102	
7	Y	Y	0.569	
8	Y	Y	0.447	
9	Y	Y	0.065	
10	Y	Y	0.808	
11	N			0.336
12	Y	Y	0.793	
13	Y	Y	0.916	
14	Y	Y	0.664	
15	Y	Y	0.969	
16	Y	Y	0.853	
17	Y	Y	0.746	
18	Y	Y	0.802	
19	Y	Y	0.668	
20	Y	Y	0.189	
21	N			0.867
22	N			0.779
23	Y	Y	0.319	
24	Y	Y	0.365	
25	Y	Y	0.626	
26	Y	Y	0.972	
27	Y	Y	0.748	
28	Y	Y	0.899	
29	Y	Y	0.779	
30	Y	Y	0.179	
31	Y	Y	0.17	
32	Y	Y	0.796	
33	Y	Y	0.648	
34	Y	Y	0.286	
35	Y	Y	0.505	
36	Y	Y	0.526	
37	Y	Y	0.494	
38	Y	Y	0.966	
39	Y	Y	0.566	
40	Y	Y	0.606	
41	Y	Y	0.203	

42	N			0.463
43	Y	Y	0.196	
44	N			0.613
45	N			0.613
46	Y	Y	0.591	
47	N			0.694
48	Y	Y	0.966	
49	Y	Y	0.776	
50	Y	Y	0.437	
51	Y	Y	0.872	
52	Y	Y	0.724	
53	Y	Y	0.345	
54	Y	Y	0.186	
55	Y	Y	0.984	
56	Y	Y	0.773	
57	Y	Y	0.844	
58	Y	Y	0.714	
59	Y	Y	0.987	
60	Y	Y	0.969	

	miR-384-5p-AS-Scr vs. control			
Time (min)	Normality test passed	Equal variance test passed	P-value (t-test)	P-value (Mann-Whitney U Test)
1				
2				
3				
4				
5				
6	Y	Y	0.9	
7	Y	Y	0.736	
8	Y	Y	0.902	
9	Y	Y	0.006	
10	Y	Y	0.763	
11	N			0.195
12	Y	Y	0.807	
13	Y	Y	0.962	
14	Y	Y	0.799	
15	Y	Y	0.941	
16	Y	Y	0.902	
17	Y	Y	0.422	
18	Y	Y	0.702	
19	Y	Y	0.248	
20	Y	Y	0.694	
21	N			0.574
22	N			0.645
23	Y	N		0.779
24	Y	Y	0.521	
25	Y	Y	0.856	
26	Y	Y	0.534	
27	Y	Y	0.994	
28	Y	Y	0.939	
29	Y	Y	0.399	
30	Y	Y	0.723	
31	Y	Y	0.294	
32	Y	Y	0.982	
33	Y	Y	0.416	
34	Y	Y	0.211	
35	Y	Y	0.443	
36	Y	Y	0.614	
37	Y	Y	0.416	
38	Y	Y	0.862	
39	Y	Y	0.467	
40	Y	Y	0.996	
41	Y	Y	0.365	

42	N			0.328
43	Y	Y	0.173	
44	N			0.959
45	N			0.645
46	N			0.574
47	N			1
48	N			0.505
49	Y	N		0.798
50	Y	Y	0.347	
51	Y	Y	0.737	
52	Y	Y	0.541	
53	Y	Y	0.294	
54	Y	Y	0.192	
55	Y	Y	0.783	
56	Y	Y	0.694	
57	Y	Y	0.982	
58	Y	Y	0.56	
59	N			0.536
60	Y	Y	0.75	

	miR-26a-AS vs. control			
Time (min)	Normality test passed	Equal variance test passed	P-value (t-test)	P-value (Mann-Whitney U Test)
1				
2				
3				
4				
5				
6	Y	Y	0.049	
7	Y	Y	0.002	
8	Y	Y	0.007	
9	N			<0.001
10	Y	Y	0.004	
11	Y	Y	0.012	
12	Y	N		0.054
13	Y	N		0.021
14	Y	N		0.072
15	Y	Y	0.054	
16	Y	Y	0.017	
17	N			0.021
18	Y	N		0.011
19	Y	N		0.035
20	Y	N		0.04
21	N			0.04
22	N			0.094
23	N			0.026
24	N			0.152
25	N			0.152
26	N			0.014
27	N			0.054
28	N			0.021
29	N			0.009
30	N			0.029
31	Y	Y	0.025	
32	Y	N		0.014
33	Y	N		0.021
34	Y	Y	0.089	
35	Y	Y	0.033	
36	N			0.094
37	Y	N		0.011
38	Y	N		0.019
39	Y	N		0.108
40	N			0.008
41	Y	Y	0.035	

42	Y	N		0.005
43	Y	N		0.03
44	Y	Y	0.003	
45	Y	Y	0.02	
46	Y	Y	0.013	
47	Y	Y	0.004	
48	Y	Y	0.004	
49	Y	Y	0.002	
50	Y	Y	0.002	
51	Y	Y	0.006	
52	Y	Y	0.003	
53	Y	Y	0.013	
54	Y	Y	0.009	
55	Y	Y	0.002	
56	Y	Y	0.001	
57	Y	Y	0.002	
58	Y	Y	<0.001	
59	Y	Y	0.003	
60	Y	Y	0.002	

	miR-384-5p-AS vs. control			
Time (min)	Normality test passed	Equal variance test passed	P-value (t-test)	P-value (Mann-Whitney U Test)
1				
2				
3				
4				
5				
6	Y	Y	0.036	
7	Y	Y	0.004	
8	Y	N		0.014
9	Y	N		0.001
10	Y	N		0.005
11	Y	Y	0.008	
12	Y	N		0.008
13	Y	N		0.03
14	Y	N		0.008
15	Y	N		0.049
16	Y	Y	0.011	
17	Y	Y	0.003	
18	Y	Y	0.003	
19	Y	N		0.003
20	Y	Y	0.007	
21	N			0.03
22	N			0.049
23	Y	Y	0.023	
24	Y	Y	0.03	
25	Y	Y	0.009	
26	Y	Y	0.003	
27	Y	Y	0.01	
28	N			0.005
29	Y	Y	<0.001	
30	Y	Y	0.004	
31	N			0.011
32	N			0.008
33	N			0.006
34	Y	Y	0.069	
35	N			0.014
36	N			0.011
37	N			0.006
38	N			0.008
39	Y	Y	0.009	
40	N			0.008
41	Y	Y	0.029	

42	N			0.014
43	N			0.011
44	Y	Y	0.012	
45	N			0.039
46	Y	Y	0.028	
47	N			0.03
48	Y	Y	0.042	
49	Y	Y	0.036	
50	Y	Y	0.015	
51	Y	Y	0.02	
52	N			0.015
53	Y	Y	0.038	
54	Y	Y	0.103	
55	Y	Y	0.042	
56	N			0.009
57	Y	Y	0.013	
58	Y	Y	0.013	
59	Y	Y	0.021	
60	Y	Y	0.035	

	miR-191-antisense vs. control			
Time (min)	Normality test passed	Equal variance test passed	P-value (t-test)	P-value (Mann-Whitney U Test)
1				
2				
3				
4				
5				
6	Y	Y	0.664	
7	Y	Y	0.815	
8	Y	Y	0.977	
9	Y	Y	0.234	
10	Y	Y	0.619	
11	Y	Y	0.444	
12	Y	Y	0.273	
13	Y	Y	0.399	
14	Y	Y	0.129	
15	Y	Y	0.147	
16	Y	Y	0.131	
17	Y	Y	0.02	
18	N			0.026
19	Y	Y	0.101	
20	Y	Y	0.011	
21	N			0.011
22	N			0.018
23	Y	Y	0.064	
24	Y	Y	0.01	
25	N			0.018
26	Y	Y	0.243	
27	Y	Y	0.056	
28	Y	Y	0.185	
29	Y	Y	0.125	
30	Y	Y	0.05	
31	Y	Y	0.023	
32	Y	Y	0.043	
33	Y	Y	0.052	
34	Y	Y	0.025	
35	Y	Y	0.042	
36	Y	Y	0.01	
37	Y	Y	0.008	
38	Y	Y	0.008	
39	Y	Y	0.005	
40	Y	Y	<0.001	
41	Y	Y	<0.001	

42	Y	Y	0.002	
43	Y	Y	<0.001	
44	N			0.018
45	N			0.006
46	Y	Y	0.01	
47	Y	Y	0.009	
48	Y	Y	0.035	
49	Y	Y	0.008	
50	Y	Y	0.004	
51	Y	Y	0.023	
52	Y	Y	0.05	
53	N			0.073
54	Y	Y	0.021	
55	Y	Y	0.14	
56	Y	Y	0.266	
57	Y	Y	0.179	
58	Y	Y	0.301	
59	Y	Y	0.259	
60	Y	Y	0.139	

Supplementary Table 7. Statistical analysis of electrophysiology data in Fig. 3g

Comparison between vehicle and BI-D1870 treated groups				
Time after LTP induction (min)	Normality Test passed	Equal Variance Test passed	P value (T-test)	P value (Mann-Whitney U Test)
3	Y	Y	0.907	
6	Y	Y	0.295	
9	Y	Y	0.294	
12	Y	Y	0.318	
15	Y	Y	0.251	
18	Y	Y	0.205	
21	Y	Y	0.354	
24	Y	Y	0.499	
27	Y	Y	0.406	
30	Y	Y	0.304	
33	Y	Y	0.539	
36	Y	Y	0.402	
39	Y	Y	0.138	
42	Y	Y	0.133	
45	Y	Y	0.135	
48	Y	Y	0.172	
51	Y	Y	0.086	
54	N			0.117
57	Y	Y	0.088	
60	Y	Y	0.064	
63	N			0.117
66	Y	Y	0.026	
69	Y	Y	0.072	
72	Y	Y	0.117	
75	Y	Y	0.008	
78	Y	Y	0.022	
81	Y	Y	0.028	
84	Y	Y	0.074	
87	Y	Y	0.073	
90	Y	Y	0.027	
93	Y	Y	0.027	
96	Y	N		0.033
99	Y	Y	0.015	
102	Y	Y	0.011	
105	Y	Y	0.008	
108	Y	Y	0.014	
111	Y	Y	0.018	
114	Y	Y	0.014	
117	Y	Y	0.01	
120	Y	Y	0.013	

Supplementary Table 8. Statistical analysis of electrophysiology data in Supplementary Fig. 7a

Comparison between pre-stimulation baseline and post-stimulation fEPSPs					
	Time (min)	Normality Test passed	Equal Variance Test passed	P value (T-test)	P value (Mann-Whitney U Test)
After first HFS	5	Y	Y	<0.001	
After first HFS	10	Y	Y	<0.001	
After first HFS	15	Y	Y	<0.001	
After first HFS	20	Y	Y	0.006	
After first HFS	25	N			0.008
After first HFS	30	Y	Y	<0.001	
After first HFS	35	N			0.008
After first HFS	40	Y	Y	<0.001	
After first HFS	45	N			0.008
After first HFS	50	N			0.008
After first HFS	55	N			0.008
After first HFS	60	N			0.008
After first HFS	65	Y	Y	0.004	
After first HFS	70	N			0.008
After first HFS	75	N			0.008
After first HFS	80	N			0.008
After first HFS	85	N			0.008
After first HFS	90	N			0.008
After first HFS	95	Y	Y	0.01	
After first HFS	100	Y	Y	0.024	
After first HFS	105	Y	Y	0.018	
After first HFS	110	Y	Y	0.095	
After first HFS	115	N			0.69
After first HFS	120	N			0.151
After washout	5	N			0.151
After washout	10	N			0.151
After washout	15	N			0.008
After washout	20	N			0.008

After washout	25	Y	Y	0.115	
After washout	30	N			0.008
After washout	35	N			0.151
After washout	40	Y	Y	0.143	
After washout	45	Y	Y	0.136	
After washout	50	N			0.69
After washout	55	N			0.69
After washout	60	N			0.151
After second HFS	5	N			0.008
After second HFS	10	Y	Y	<0.001	
After second HFS	15	Y	Y	<0.001	
After second HFS	20	Y	Y	<0.001	
After second HFS	25	Y	Y	<0.001	
After second HFS	30	Y	Y	<0.001	
After second HFS	35	N			0.008
After second HFS	40	N			0.008
After second HFS	45	Y	Y	<0.001	
After second HFS	50	N			0.008
After second HFS	55	Y	Y	<0.001	
After second HFS	60	N			0.008
After second HFS	65	Y	Y	<0.001	
After second HFS	70	Y	Y	<0.001	
After second HFS	75	Y	Y	<0.001	
After second HFS	80	Y	Y	<0.001	
After second HFS	85	Y	Y	<0.001	
After second HFS	90	Y	Y	<0.001	
After second HFS	95	Y	Y	<0.001	
After second HFS	100	N			0.008
After second HFS	105	N			0.008
After second HFS	110	Y	Y	<0.001	
After second HFS	115	Y	Y	<0.001	
After second HFS	120	Y	Y	<0.001	

Supplementary Table 9. Statistical analysis of electrophysiology data in Fig. 3h.

Comparison of 4 groups (EGFP, RSK3 siRNA, RSK3 siRNA+RSK3-m, RSK3)				
Time after LTP induction (min)	Normality test passed	Equal variance test passed	P-value (One-way ANOVA)	P-value (Kruskal-Wallis test)
3	Y	Y	0.282	
6	Y	N		0.726
9	Y	Y	0.495	
12	Y	N		0.747
15	N			0.709
18	N			0.71
21	Y	Y	0.554	
24	Y	N		0.701
27	Y	Y	0.556	
30	Y	Y	0.734	
33	Y	Y	0.639	
36	Y	N		0.786
39	Y	Y	0.417	
42	Y	Y	0.421	
45	Y	Y	0.283	
48	Y	Y	0.623	
51	Y	Y	0.498	
54	Y	Y	0.28	
57	Y	Y	0.204	
60	Y	Y	0.161	
63	Y	Y	0.257	
66	Y	N		0.04
69	Y	Y	0.096	
72	Y	Y	0.049	
75	Y	N		0.045
78	Y	Y	0.099	
81	Y	Y	0.156	
84	Y	Y	0.041	
87	Y	Y	0.038	
90	Y	Y	0.013	
93	Y	Y	0.018	
96	Y	Y	0.024	
99	N			0.013
102	Y	Y	0.056	
105	Y	Y	0.01	
108	Y	Y	0.023	
111	Y	Y	0.016	
114	Y	Y	0.006	
117	N			0.004
120	Y	Y	0.012	

	RSK3 siRNA vs. EGFP			
Time after LTP induction (min)	Normality test passed	Equal variance test passed	P-value (t-test)	P-value (Mann-Whitney U Test)
3				
6				
9				
12				
15				
18				
21				
24				
27				
30				
33				
36				
39				
42				
45				
48				
51				
54				
57				
60				
63				
66	Y	N		0.04
69				
72	Y	N		0.025
75	Y	N		0.011
78				
81				
84	N			0.006
87	Y	Y	0.016	
90	Y	Y	0.004	
93	Y	Y	0.008	
96	Y	Y	0.015	
99	N			0.025
102				
105	N			0.004
108	Y	Y	0.015	
111	Y	Y	0.021	
114	Y	Y	0.006	
117	N			0.004
120	Y	N		0.008

	RSK3 siRNA+RSK3-m vs. EGFP			
Time after LTP induction (min)	Normality test passed	Equal variance test passed	P-value (t-test)	P-value (Mann-Whitney U Test)
3				
6				
9				
12				
15				
18				
21				
24				
27				
30				
33				
36				
39				
42				
45				
48				
51				
54				
57				
60				
63				
66	Y	Y	0.881	
69				
72	Y	Y	0.302	
75	Y	Y	0.436	
78				
81				
84	Y	Y	0.811	
87	Y	Y	0.824	
90	Y	Y	0.755	
93	Y	Y	0.822	
96	Y	Y	0.802	
99	Y	Y	0.881	
102				
105	Y	Y	0.557	
108	Y	Y	0.777	
111	Y	Y	0.933	
114	Y	Y	0.93	
117	Y	Y	0.718	
120	Y	Y	0.727	

	RSK3 vs. EGFP			
Time after LTP induction (min)	Normality test passed	Equal variance test passed	P-value (t-test)	P-value (Mann-Whitney U Test)
3				
6				
9				
12				
15				
18				
21				
24				
27				
30				
33				
36				
39				
42				
45				
48				
51				
54				
57				
60				
63				
66	Y	Y	0.56	
69				
72	Y	Y	0.328	
75	Y	Y	0.403	
78				
81				
84	Y	Y	0.83	
87	Y	Y	0.832	
90	Y	Y	0.718	
93	Y	Y	0.934	
96	Y	Y	0.607	
99	Y	Y	0.673	
102				
105	Y	Y	0.677	
108	Y	Y	0.7	
111	Y	Y	0.377	
114	Y	Y	0.566	
117	Y	Y	0.962	
120	Y	Y	0.987	

Supplementary Table 10. Statistical analysis of electrophysiology data in Fig. 3i.

Comparison of 3 groups (EGFP, miR-26a+RSK3, miR-384-5p+RSK3)				
Time after LTP induction (min)	Normality test passed	Equal variance test passed	P-value (One-way ANOVA)	P-value (Kruskal-Wallis test)
3	N			0.168
6	N			0.417
9	N			0.437
12	N			0.653
15	N			0.668
18	N			0.393
21	N			0.437
24	N			0.574
27	N			0.9
30	N			0.728
33	N			0.75
36	N			0.51
39	N			0.665
42	N			0.863
45	N			0.887
48	N			0.895
51	N			0.622
54	N			0.683
57	N			0.705
60	N			0.583
63	N			0.83
66	N			0.871
69	N			0.776
72	N			0.402
75	N			0.349
78	N			0.587
81	Y	Y	0.679	
84	N			0.793
87	N			0.708
90	N			0.64
93	N			0.687
96	N			0.746
99	N			0.591
102	N			0.613
105	N			0.534
108	N			0.496
111	N			0.482
114	N			0.506
117	N			0.215
120	N			0.483

Supplementary Table 11. Statistical analysis of electrophysiology data in Fig. 10a.

Comparison between EGFP and let-7 virus-transduced slices				
Time after LTP induction (min)	Normality Test passed	Equal Variance Test passed	P value (T-test)	P value (Mann-Whitney U Test)
3	Y	Y	0.393	
6	N			0.213
9	Y	Y	0.325	
12	N			0.65
15	Y	Y	0.652	
18	Y	Y	0.865	
21	N			0.91
24	Y	Y	0.543	
27	N			0.213
30	Y	Y	0.68	
33	Y	Y	0.519	
36	Y	Y	0.158	
39	N			0.23
42	Y	Y	0.064	
45	Y	Y	0.125	
48	Y	Y	0.192	
51	Y	Y	0.188	
54	Y	Y	0.068	
57	Y	Y	0.072	
60	Y	Y	0.054	
63	Y	Y	0.074	
66	Y	Y	0.055	
69	Y	Y	0.089	
72	Y	Y	0.037	
75	Y	Y	0.041	
78	Y	Y	0.034	
81	Y	Y	0.076	
84	Y	Y	0.121	
87	Y	Y	0.047	
90	Y	Y	0.021	
93	Y	Y	0.041	
96	Y	Y	0.048	
99	Y	Y	0.091	
102	Y	Y	0.076	
105	Y	Y	0.032	
108	Y	Y	0.046	
111	N			0.046
114	Y	Y	0.028	
117	Y	Y	0.029	
120	Y	Y	0.045	
